# Climate Exposure of US National Parks in a New Era of Change

**DOI:** 10.1371/journal.pone.0101302

**Published:** 2014-07-02

**Authors:** William B. Monahan, Nicholas A. Fisichelli

**Affiliations:** National Park Service, Natural Resource Stewardship & Science, Fort Collins, Colorado, United States of America; University of Vigo, Spain

## Abstract

US national parks are challenged by climate and other forms of broad-scale environmental change that operate beyond administrative boundaries and in some instances are occurring at especially rapid rates. Here, we evaluate the climate change exposure of 289 natural resource parks administered by the US National Park Service (NPS), and ask which are presently (past 10 to 30 years) experiencing extreme (<5^th^ percentile or >95^th^ percentile) climates relative to their 1901–2012 historical range of variability (HRV). We consider parks in a landscape context (including surrounding 30 km) and evaluate both mean and inter-annual variation in 25 biologically relevant climate variables related to temperature, precipitation, frost and wet day frequencies, vapor pressure, cloud cover, and seasonality. We also consider sensitivity of findings to the moving time window of analysis (10, 20, and 30 year windows). Results show that parks are overwhelmingly at the extreme warm end of historical temperature distributions and this is true for several variables (e.g., annual mean temperature, minimum temperature of the coldest month, mean temperature of the warmest quarter). Precipitation and other moisture patterns are geographically more heterogeneous across parks and show greater variation among variables. Across climate variables, recent inter-annual variation is generally well within the range of variability observed since 1901. Moving window size has a measureable effect on these estimates, but parks with extreme climates also tend to exhibit low sensitivity to the time window of analysis. We highlight particular parks that illustrate different extremes and may facilitate understanding responses of park resources to ongoing climate change. We conclude with discussion of how results relate to anticipated future changes in climate, as well as how they can inform NPS and neighboring land management and planning in a new era of change.

## Introduction

Recent scientific reviews of the US National Park Service (NPS) suggest the agency needs to manage its parks for changing climatic and ecological baselines in a landscape context [1]–[3]. These recommendations reiterate and heighten the imperative that it is no longer ecologically viable to manage resources solely within park boundaries, nor feasible to meet objectives based on pre-European settlement conditions. Broad-scale anthropogenic changes impact even the most remote areas with the highest levels of protection (e.g., federally designated wildernesses [4]). Climate change, land use change, pollution, and invasive species are pervasive and have additive and interactive effects on species and ecosystems [5], and they challenge the NPS to work under rapidly shifting conditions at broad spatial scales in order to protect park resources for future generations [6], [7].

Climate change is a principal factor driving the imperative for progressive management aimed at achieving a specified ‘desired future condition’ (DFC) [8], [9]. Magnitudes and rates of modeled future climate change suggest that temperatures in many regions of the globe may shift outside the envelope of historical (1860–2005) variability by mid-century [10], thus requiring DFCs to depart from past observations or records. Furthermore, climate change may already be affecting park natural resources. According to the Intergovernmental Panel on Climate Change (IPCC), globally each of the last three decades was increasingly warmer than any preceding decade since 1850, and – in the northern hemisphere –1983–2012 was possibly the warmest 30-year period of the last 1400 years [11]. In these situations, DFC management confronts existing ‘retrospective’ management practices that have for decades worked to preserve areas and resources within an ‘historical range of variability’ (HRV) [12]. For example, in a coastal park such as Point Reyes National Seashore [13], where the physical shoreline and intertidal wetlands are important resources, park managers may need to consider facilitating the inland migration of both features into new areas, rather than retain HRV by resisting sea level rise. Depending on the proximity of the current shoreline to the park’s boundary, some of this DFC management may necessitate accepting or promoting resource migration beyond areas administered by the NPS. Due in part to logistical dilemmas posed by these possible futures, coupled with their novelty, there is often a desire to – where possible and as supported by current policy – manage under an existing, observed HRV, rather than an entirely new and uncertain set of conditions.

Recommendations to both scale up and consider changing baselines [1]–[3] encourage a thorough analysis of the exposure of individual parks and surrounding landscapes to climate change. Such insights are foundational to understanding which climate drivers are already statistically extreme relative to HRV, and whether ongoing climate change will further exacerbate (vs. alleviate) these conditions. Here, we evaluate the recent climate (past 10–30 years) of each US NPS natural resource park relative to its HRV across the past 112 years (1901–2012). We consider parks in a landscape context (park + surrounding 30 km) and evaluate both mean and inter-annual variation in major climate drivers over multiple climatically and management relevant time periods. Our analyses address three questions relevant to individual park management and NPS planning and policy: (i) relative to 1901–2012 HRV, do recent climate conditions NPS-wide tend to be unusually low or high on certain climate variables, (ii) how sensitive are these statistical distributions to the time window of analysis (10, 20, or 30 years), and (iii) which individual parks are climatically extreme relative to their HRV? We conclude with discussion of how results relate to anticipated future changes in climate and – by extension – how they may inform NPS climate adaptation.

## Materials and Methods

### Study Sites

Our analyses were performed for all natural resource parks considered by the NPS Inventory & Monitoring Program’s landscape dynamics monitoring project, NPScape (*n* = 289), including a 30 km buffer around each park’s boundary [14]. Hereafter these areas of analysis are referred to generally as ‘parks’. The 30 km buffer was selected because of the relatively coarse spatial resolution of the climate data (see below), as well as to approximate the surrounding zone of ecological influence [15]. Natural resource parks range from approximately 14°S to 68°N latitude (spanning the Equator) and 115°W to 65°E longitude (spanning the International Date Line), hence the need to use global climate data as a single, common source for consistent coverage of all parks in this study.

### Climate Data

Gridded climate data were obtained from the Climatic Research Unit (CRU) high-resolution time series version 3.21 (TS 3.21) [16]. CRU TS 3.21 data are globally available at 0.5 decimal degrees (approximately 3000 km^2^ at the Equator and 2000 km^2^ at 70°N latitude) for each month × year, 1901–2012. Although this dataset is spatially coarse, at least relative to the size of some parks (e.g., the smallest park with 30 km buffer encompasses roughly one grid cell), it offers for all NPS geographies the highest spatiotemporal resolution of observed climate over the 20^th^ and 21^st^ centuries. We selected for analysis monthly averages of daily temperature (minimum, maximum, mean; °C), percentage cloud cover, and vapor pressure (haP), as well as total monthly precipitation (mm) and monthly frequencies of frost days (a period of 24 hours in which the minimum temperature falls below 0°C) and wet days (rain days per month). We used the monthly climate variables to calculate a series of biologically meaningful variables, including 19 standard bioclimatic variables [17], as well as six other variables that relate to photosynthetic activity and plant growth [18], [19] (Table 1).

**Table 1:** Biologically relevant variables considered in the analyses of climate change exposure of US national parks.

Code	Name
Bio1	Annual mean temperature
Bio2	Mean diurnal range (mean of monthly (max temp – min temp))
Bio3	Isothermality (Bio2/Bio7)
Bio4	Temperature seasonality (standard deviation)
Bio5	Maximum temperature of the warmest month
Bio6	Minimum temperature of the coldest month
Bio7	Temperature annual range (Bio5– Bio6)
Bio8	Mean temperature of the wettest quarter
Bio9	Mean temperature of the driest quarter
Bio10	Mean temperature of the warmest quarter
Bio11	Mean temperature of the coldest quarter
Bio12	Annual precipitation
Bio13	Precipitation of the wettest month
Bio14	Precipitation of the driest month
Bio15	Precipitation seasonality (coefficient of variation)
Bio16	Precipitation of the wettest quarter
Bio17	Precipitation of the driest quarter
Bio18	Precipitation of the warmest quarter
Bio19	Precipitation of the coldest quarter
Cld1	Mean annual percentage cloud cover
Cld4	Cloud seasonality (standard deviation)
Vap18	Vapor pressure of the warmest quarter
Wet12	Annual number of wet days
Wet18	Number of wet days of the warmest quarter
Frs12	Annual number frost days

Although correlations exist among some of these variables, we include the full suite in our analysis because the relevance of individual climate variables can vary dramatically by park resource (e.g., minimum winter temperature may influence a bird distributional limit, while maximum summer temperature may be a more important driver for particular mammals). As such, the elimination of certain variables due to high statistical correlations would adversely restrict the value of these analyses for park interpretation. Furthermore, the data presented here are percentiles calculated from HRV (see below), not the raw climate variables. Hence, whether variables are correlated is not simply a question of correlation at one point in time; it is a complex question of how sensitive correlations are across the entire 1901–2012 period of analysis, interacting with the moving time window of summarization.

### Statistical Analysis

For each biologically relevant variable, over the entire time series (1901–2012), we used three moving windows (10, 20, and 30 years) to calculate a series of running means and standard deviations (SD). Hereafter we refer to these as ‘moving window means’ and ‘moving window standard deviations’. These statistical distributions are what we use to estimate HRV. For example, with the 10 year window, we calculated the mean and SD for 103 windows (1901–1910, 1902–1911, …, 2003–2012) to create the HRV distribution. The three windows were designed to encompass both near- and long-term management and planning considerations, as well as important climatic periods and cycles. In the NPS, many implementation plans have short horizons (<10 years), while strategic plans forecast out 10 to 20 years [20]. Traditional climate summaries span periods of 30 years [21], while major climate cycles affecting NPS geographies (e.g., Pacific Decadal Oscillation and North Atlantic Oscillation) tend to operate roughly on decadal to multi-decadal scales (e.g., over periods of 10, 20, or 30 years) [22].

For each variable, moving window size, and summary statistic (temporal mean and SD), we calculated the area-weighted mean for each park, based on the spatial intersect of park areas of analysis with the CRU lattice. Using processing logic contained in the NPScape climate grid analysis toolset [23], a resource designed by the NPS Inventory and Monitoring Program to facilitate common statistical analyses of gridded climate data, we then computed using R [24] the percentile of each most recent window (2003–2012, 1993–2012, 1983–2012) on the corresponding distribution of moving window values for each park (i.e., its HRV). In other words, we quantified recent conditions as the percentile of the most recent moving window value relative to the entire distribution of moving window values for that climate variable and window size (e.g., a 90^th^ percentile for the most recent 10 year moving window of annual mean temperature signifies that the value of this climate variable during this time period was greater than 90% of all annual mean temperature 10 year moving window values). Hereafter we refer to these estimates as ‘recent percentiles’.

We then averaged the recent percentiles of the most recent 10, 20, and 30 year moving windows and computed the maximum difference in percentile (max Δ) among windows; this resulted in – for each park, variable, and summary statistic – both an overall measure of recent climate change exposure with respect to HRV, and an estimate of how sensitive that measure was by comparing the different moving window sizes. Note that our measure of variability (moving window SD) does not reveal any information regarding extreme events, such as droughts, heat waves, and storms, which can also impact park resources [25].

**Figure 1:**
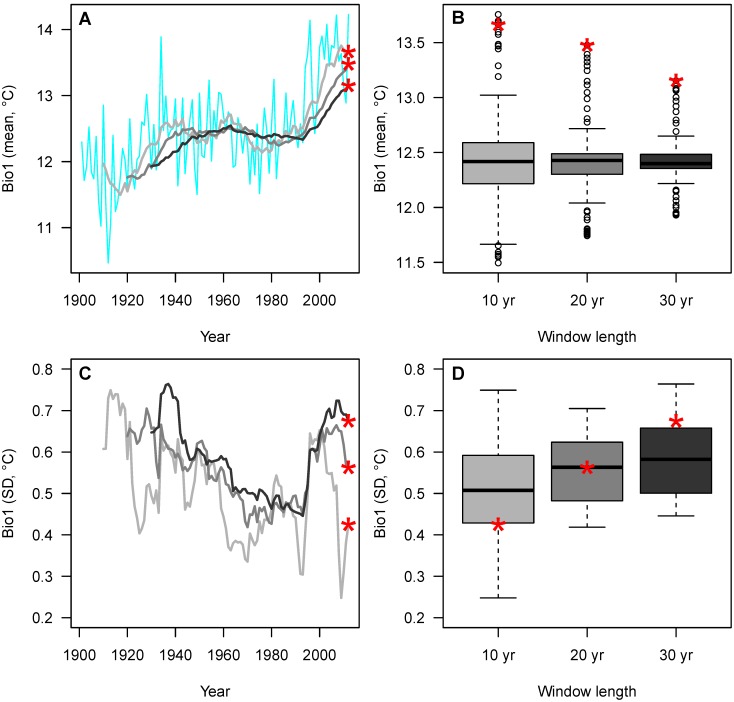
Example moving window time series (used to estimate HRV) and recent percentiles shown for Bio1 (annual mean temperature), Grand Canyon National Park (with 30 km buffer). A, B) Moving window means; C, D) Moving window standard deviations (SD). Three moving windows –10 years (light gray), 20 years (medium gray), and 30 years (dark gray) – are calculated from the annual time series (blue, A). Bio1 values for the most recent windows (2003–2012 (10 yr), 1993–2012 (20 yr), 1983–2012 (30 yr)) are indicated by the red asterisks. Boxes in B and D are the inter-quartile range (median = thick perpendicular line), dashed lines the outer tails (1.5× inter-quartile range), and dots the outliers. Recent percentiles are calculated for the red asterisks shown in B and D.

An example of these steps with annual mean temperature (Bio1) is shown for Grand Canyon National Park (Figure 1). In this instance, the percentiles associated with the most recent mean temperature windows (computed over 10, 20, and 30 years) are all >95% (mean = 99%), showing that recent temperature has been at the extreme warm end of the entire distribution of moving windows (see location of red asterisks in boxplot distributions, Figure 1B). The max Δ for mean temperature is relatively small (2.9%), confirming that recent conditions have been very warm across all three window sizes. The standard deviation of Bio1 (inter-annual variability over 10, 20, and 30 years) shows a more variable pattern among window sizes, with SD percentiles associated with the most recent windows varying between 25 and 80% (red asterisks, Figure 1D). Hence, estimates of recent inter-annual variability in annual mean temperature are sensitive to window size (max Δ = 55%).

Given the number and complexity of variables considered in our analysis, we performed two multivariate analyses in an effort to produce more integrated summaries of recent percentiles. Principal component analyses were used to identify a small set of uncorrelated axes for each combination of summary statistic (temporal mean and SD) and measure (mean percentile and max Δ percentile). We also conducted a threshold-based classification analysis of mean percentiles, on both the moving window mean and SD, to characterize which parks were extreme on their annual, monthly, or quarterly temperature (Bio1, 5, 6, 8–11) or precipitation (Bio12–14, 16–19) variables. In this classification analysis, as well as in other evaluations of individual climate variables, we define ‘extreme’ as either less than the 5^th^ percentile (low) or greater than the 95^th^ percentile (high), and all intervening percentiles as ‘average’. We tallied the total number of temperature and precipitation variables that met either of these conditions and categorized each park as extreme low or high on temperature and precipitation if at least one variable in each class was low or high. Parks were considered ‘mixed’ if they possessed low and high estimates of temperature, or low and high estimates of precipitation. Then, considering only the low and high variables, we calculated the maximum value of their max Δ’s to provide a maximum estimate of sensitivity to window size. Given the thresholds used to define ‘extreme’, the maximum possible value for max Δ is 15%.

For a subset of parks spanning the contiguous US, we conducted another sensitivity analysis using local weather station data to examine the degree to which CRU results differ from those obtained using direct climate observations. For local station data, we identified 18 United States Historical Climatology Network stations located within park boundaries that similarly span the 1901–2012 period of analysis [26]. We repeated the above threshold-based classification analysis and found strong correspondence between datasets. Agreement in classification of extreme warm temperatures based on moving window means occurred at 17 of 18 sites (94%); the station data identified all parks as extreme warm whereas the CRU data found one park that was not extreme. For precipitation moving window means, agreements in classification for extreme wet and extreme dry conditions were 83% and 94%, respectively. Considering extremes of both temperature and precipitation, the CRU and weather station datasets matched in moving window mean classifications at 14 of 18 sites (78%). With moving window standard deviations, agreements in temperature and precipitation classifications were on average 74%. These comparisons suggest that the CRU dataset likely provides reasonable climate estimates for parks in this analysis, especially considering how the two data sources measure climate at such different spatial scales (see discussion).

Lastly, as part of the CRU-based classification analysis, for each climate variable (Bio1, 5, 6, 8–14, 16–19) we used a Monte Carlo simulation to test whether the number of extreme parks was significantly different than random expectations. To conduct this test, we first randomly selected 100000 samples of size 289 from a uniform distribution (min = 0, max = 1) to develop a sampling distribution of the expected number of extreme parks under the null hypothesis (mean = 14.5, SD = 3.7). Using that sampling distribution, we then computed the probability of obtaining the observed number of extreme parks for each climate variable.

## Results

### Climatic Variation across the NPS

**Figure 2:**
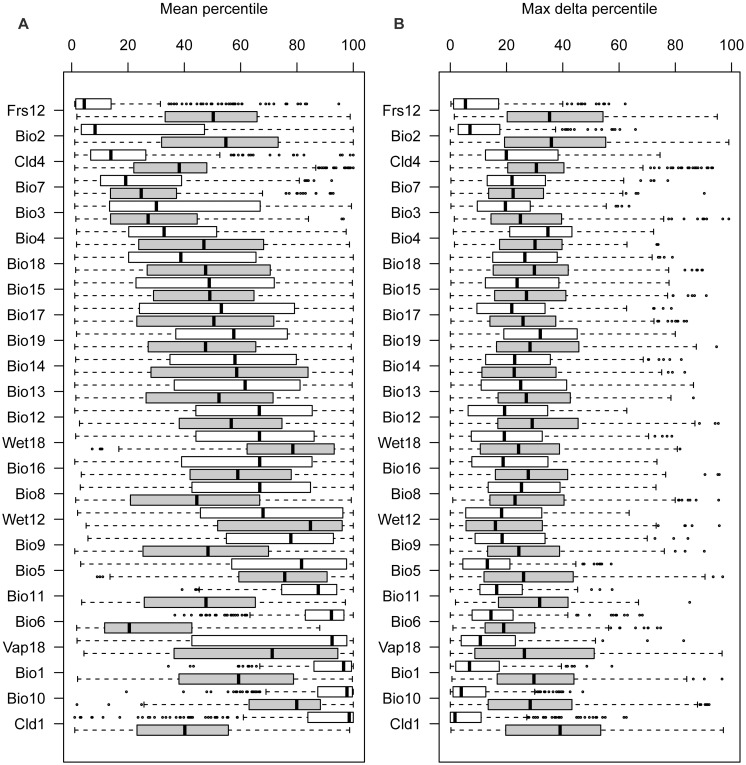
Distribution of recent climate percentiles from each park (with 30 km buffer) for 25 biologically relevant climate variables, showing both moving window means (white boxes) and moving window standard deviation (gray boxes), calculated for three moving windows (10, 20, 30 years). A) Mean percentile across windows; B) Maximum difference in percentile across windows. Boxes are the inter-quartile range (median = thick perpendicular line), dashed lines the outer tails (1.5× inter-quartile range), and dots the outliers. Climate variables are sorted based on median values of mean climate percentiles, thus for a large majority of parks, recent conditions include very low numbers of frost days (Frs12), low diurnal range (Bio2), very warm annual and summer temperatures (Bio1 and 10), and very high cloud cover (Cld1). See Table 1 for definitions of climate variables.

Parks vary considerably in how their recent climates compare to their 1901–2012 HRV, as estimated by mean percentiles for moving window means and standard deviations (Figure 2). For recent mean percentiles associated with moving window means (Figure 2A, white boxes), parks are most broadly distributed on precipitation variables (Bio12–19) and most skewed on temperature and cloud variables (Bio1, 6, 10, 11, Cld1, 4, Frs12). In particular, recent percentiles for moving window means associated with temperature variables tend to be quite high across most parks (e.g., annual mean temperature (Bio1) is higher than the 50^th^ percentile in 99% of parks), while recent percentiles associated with the annual number of frost days (Frs12) are correspondingly quite low (Figure 2A). The mean diurnal temperature range (Bio2) is also low, suggesting a greater warming of nighttime low temperatures than of daytime highs.

For recent mean percentiles associated with moving window standard deviations (Figure 2A, gray boxes), parks are most skewed low on temperature variables (Bio3, 6, 7) and skewed high on the number of wet days (Wet12, 18) and vapor pressure (Vap18). Hence, recent patterns of inter-annual variability tend to be especially low or high on these variables. However, compared to moving window means, percentiles for moving window standard deviations are overall less skewed and more broadly distributed across parks.

### Sensitivity to Moving Window Size

Moving window size has a considerable and consistent effect across most parks and variables on the maximum difference in recent percentile, as evident by inter-quartile ranges often extending to upwards of 30 to 40% (Figure 2B). In other words, evaluating whether a park is presently low or high on its HRV can depend on the time window of analysis. In terms of moving window means, the same temperature and cloud variables with skewed recent mean percentile distributions (Figure 2A, white boxes) also tend to exhibit low maximum differences in recent percentile (Figure 2B, white boxes). Similar patterns are seen with moving window standard deviations (comparing gray boxes between Figures 2A and 2B).

Contrary to the example shown for Grand Canyon National Park in Figure 1, the maximum difference in recent percentile between moving windows is not always based on 10 and 30 year moving windows. Omitting ties (∼10% of the park × variable combinations), 49% of the largest ranges in recent moving window means involve 10 and 30 years, 30% involve 10 and 20 years, and 21% involve 20 and 30 years. For moving window standard deviations, 46% of the largest ranges involve 10 and 30 years, 30% involve 10 and 20 years, and 24% involve 20 and 30 years. Hence, about half the observations do not follow a simple linear trend whereby change in the 10, 20, and 30 year windows is unidirectional.

### Climatically Extreme vs. Average Parks

The mean and maximum difference in recent percentile for all parks and variables are given as a series of appendices for moving window means (Appendix S1: Bio1–6; Appendix S2: Bio7–11; Appendix S3: Bio12–19; Appendix S4: Cld1, 4, Vap18, Wet12, 18, Frs12) and standard deviations (Appendix S5: Bio1–6; Appendix S6: Bio7–11; Appendix S7: Bio12–19; Appendix S8: Cld1, 4, Vap18, Wet12, 18, Frs12).

**Figure 3:**
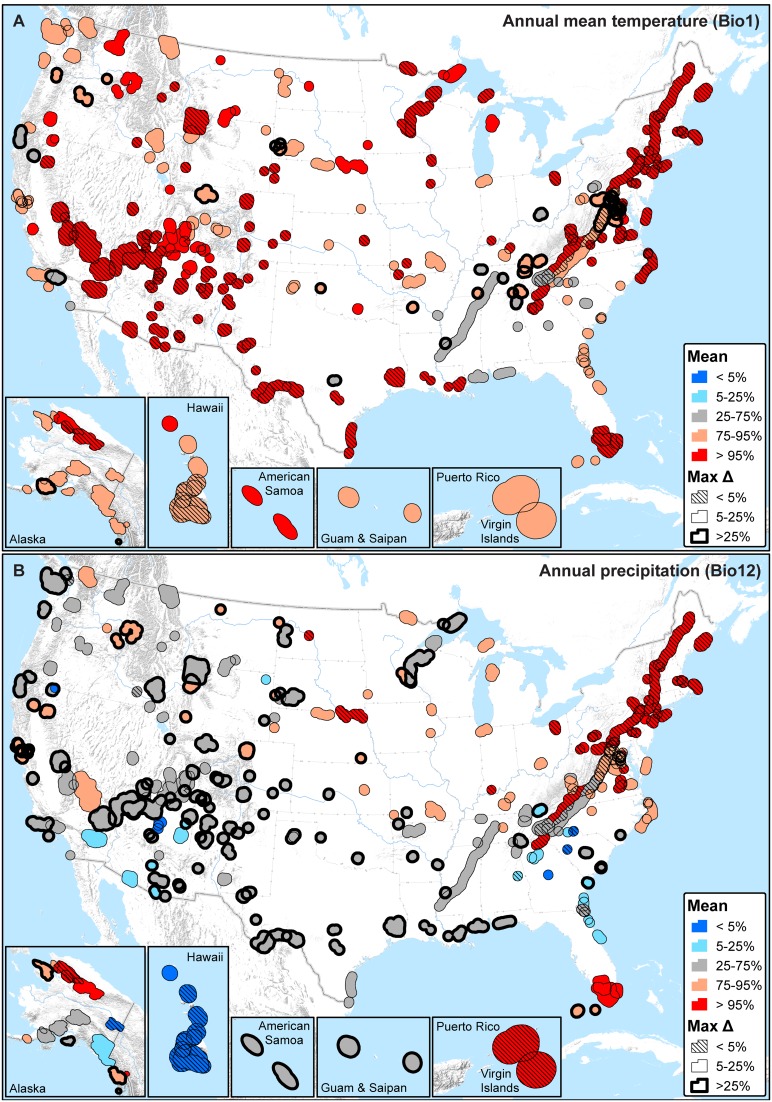
The average (Mean) and maximum difference (MaxΔ) of recent percentiles calculated for moving window means. A) Annual mean temperature (Bio1); B) Annual precipitation (Bio12). Mean values provide an overall measure of recent (past 10, 20, and 30 year windows) climate change exposure with respect to 1901–2012 HRV, while the maximum difference measures sensitivity to moving window size (smaller values are less sensitive).

**Figure 4:**
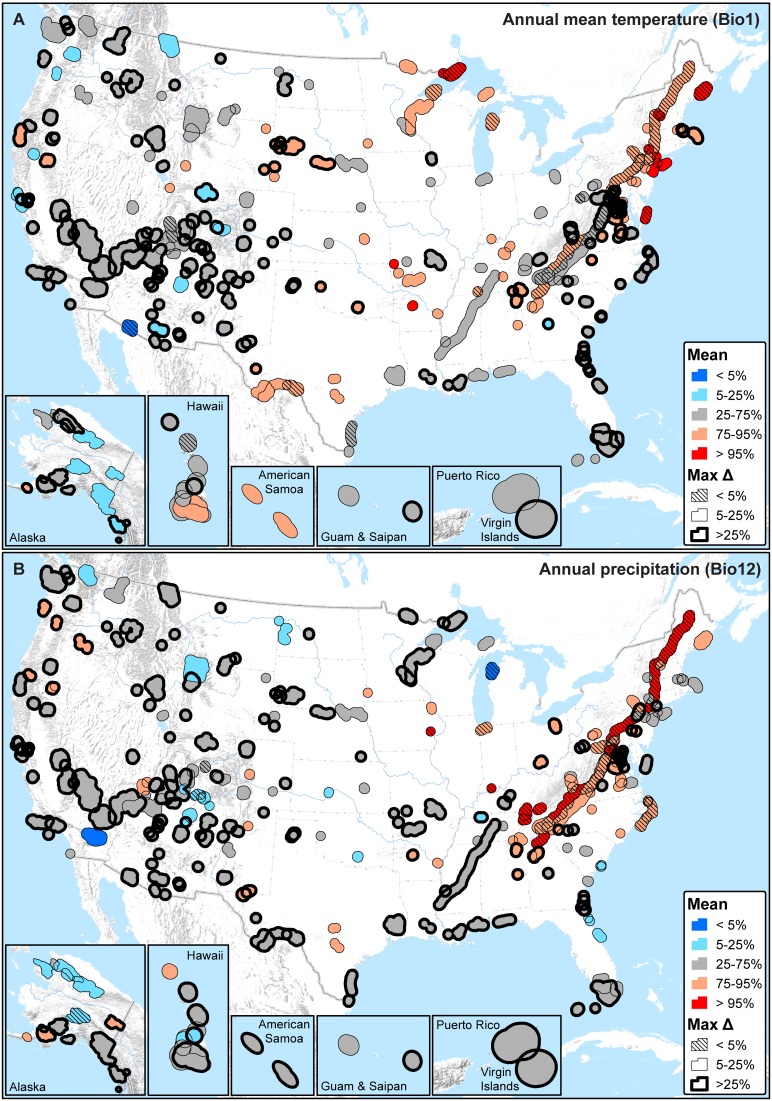
The average (Mean) and maximum difference (MaxΔ) of recent percentiles calculated for moving window standard deviations. A) Annual mean temperature (Bio1); B) Annual precipitation (Bio12). Mean values provide an overall measure of recent (past 10, 20, and 30 year windows) climate change exposure with respect to 1901–2012 HRV, while the maximum difference measures sensitivity to moving window size (smaller values are less sensitive).

Focusing on two common climate variables, annual mean temperature (Bio1) and annual precipitation (Bio12), geographic distributions of recent percentiles are illustrated for moving window means (Figure 3) and standard deviations (Figure 4). Considering moving window means for annual mean temperature (Figure 3A), practically all natural resource parks are warm with respect to their HRV (262 parks (91%) are greater than the 75^th^ percentile; 158 parks (55%) are greater than the 95^th^ percentile), with low sensitivity to moving window size (125 parks (43%) have a maximum difference in percentile that is less than 5%). This general pattern holds for parks from the Pacific Islands to Alaska to the East Coast of the US.

Corresponding results for annual precipitation (Figure 3B) are rather different: parks are heterogeneous with respect to where recent precipitation percentiles fall on their HRV and in many instances are very sensitive to moving window size (i.e., large max Δ). Furthermore, unlike annual mean temperature, most parks have average recent precipitation percentiles: 135 parks (47%) are within the 25^th^ to 75^th^ percentiles and 233 parks (81%) are within the 5^th^ to 95^th^ percentiles. Hence, only 56 parks (19%) are extreme (<5^th^ percentile or >95^th^ percentile) with respect to annual precipitation.

In terms of inter-annual variability, as measured by moving window standard deviations, recent percentiles for both annual mean temperature (Figure 4A) and annual precipitation (Figure 4B) also exhibit complex heterogeneous patterns that are highly sensitive to window size. Most parks have average recent percentiles for inter-annual variability. For annual mean temperature, 176 parks (61%) are within the 25^th^ to 75^th^ percentiles and 275 parks (95%) are within the 5^th^ to 95^th^ percentiles. Annual precipitation is similar: 188 parks (65%) are within the 25^th^ to 75^th^ percentiles and 277 parks (96%) are within the 5^th^ to 95^th^ percentiles. Hence, only about 10 parks are extreme with respect to either annual mean temperature or annual precipitation, and these parks differ between the two variables (Figure 4).

Principal component analyses on the data in Figure 2 reveal further that the percentiles encompass complex correlations that can only be partially captured by a handful of uncorrelated multivariate axes. Principal components 1 and 2 (PC1 and PC2) for the recent mean percentile associated with moving window means explain 38% of the variation (PC1: 21%; PC2: 17%) while those associated with moving window standard deviations explain 31% (PC1: 20%; PC2: 11%). Similarly, PC1 and PC2 for the maximum difference in percentile associated with moving window means explain 26% of the variation (PC1: 15%; PC2: 11%) while those associated with moving window standard deviations explain 21% (PC1: 12%; PC2: 9%). The top two components thus only capture a portion of the information contained by the original 25 variables and suggest strong heterogeneity in climate patterns across parks.

**Figure 5:**
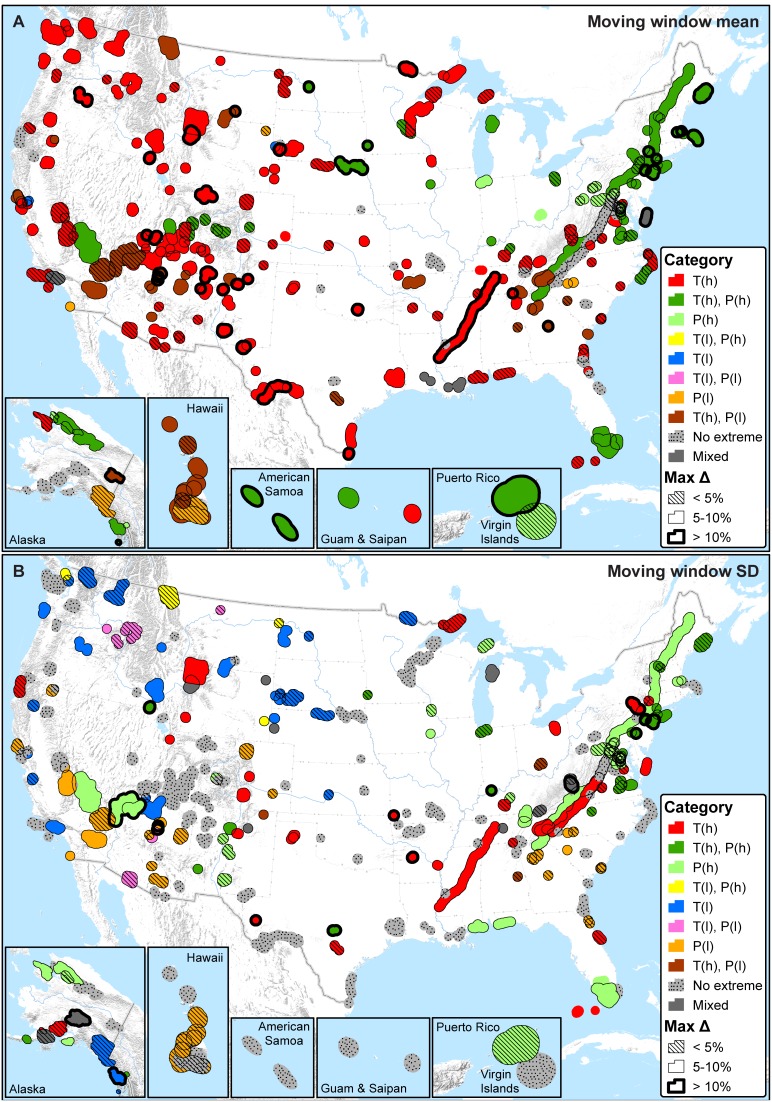
Summary of all temperature (Bio1, 5, 6, 8–11) and precipitation (Bio12–14, 16–19) variables with recent mean percentiles that are either less than the 5^th^ percentile or greater than the 95^th^ percentile (i.e., extreme on HRV). A) Moving window means; B) Moving window standard deviations (SD). T(l) = one or more temperature variables low (<5^th^ percentile; ‘cold’ or decreased inter-annual variability). T(h) = one or more temperature variables high (>95^th^ percentile; ‘warm’ or increased inter-annual variability). P(l) = one or more precipitation variables low (<5^th^ percentile; ‘dry’ or decreased inter-annual variability). P(h) = one or more precipitation variables high (>95^th^ percentile; ‘wet’ or increased inter-annual variability). Parks with both T(l) and T(h) or both P(l) and P(h) are symbolized as ‘mixed’. Parks that fall between the 5^th^ and 95^th^ percentiles on all temperature and precipitation variables are symbolized as ‘no extreme’. The maximum difference (Max Δ) in percentile was calculated only for temperature and precipitation variables that were extreme; maximum values are reported to represent maximum sensitivity. Max Δ is undefined in the case of parks symbolized as ‘no extreme’; parks that fall into all other categories, and with a max Δ of 0, are also shown without outlines.

In the classification analysis, considering extreme climates (<5^th^ percentile or >95^th^ percentile) triggered by any one of the temperature (Bio1, 5, 6, 8–11) or precipitation (Bio12–14, 16–19) variables, most parks are presently dominated by extreme high temperatures (235 parks (81%) are higher than the 95^th^ percentile), and – independent of temperature – some also by either extreme high precipitation (78 parks (27%) are higher than the 95^th^ percentile) or low precipitation (43 parks (15%) are lower than the 5^th^ percentile) (Figure 5A). Excluding the 30 parks (10%) that do not exhibit any extreme percentiles with respect to temperature or precipitation, the maximum difference in percentile across moving windows is less than 10% in 84% of the remaining parks (Figure 5A).

The number of parks that are extreme high or low on each temperature or precipitation variable considered in the classification analysis are reported in Table 2 and summarized below. For all temperature variables (Bio1, 5, 6, 8–11), a significantly large number of parks exhibit extreme high temperatures, while a significantly small number of parks exhibit extreme low temperatures (Table 2). The three most frequent extreme high temperature variables are Bio10 (170 parks, 59%), Bio1 (158 parks, 55%), and Bio6 (102 parks, 35%); numbers sum to more than 234 because parks could be extreme on more than one variable. Extreme low temperature is only documented for two variables (Bio5 and 8) in a total of 3 parks (1%). The number of parks with extreme high precipitation is significantly large for four out of 7 variables, e.g., Bio12 and 16 (44 parks each, 15%). Bio14 and 19 are associated with a significantly small number of parks with extreme low values (8 parks each, 3%). Two of the three ‘mixed’ category parks are explained by concurrent high and low extremes of precipitation. For example, Assateague Island National Seashore in Maryland has experienced extreme warm temperatures (Bio1, 5, 10), extreme wet conditions during the wettest quarter (Bio16), and extreme dry conditions during the driest month (Bio14). The third mixed category park, Santa Monica Mountains National Recreation Area in southern California, has experienced extreme cold conditions for maximum temperature of the warmest month (Bio5), and extreme warm conditions for minimum temperature of the coldest month (Bio6), resulting in an extreme low percentile for temperature annual range (Bio7).

**Table 2:** Monte Carlo simulations identifying significantly small (<9) or large (>20) numbers of parks with extreme climates (recent mean percentiles either <5^th^ percentile or >95^th^ percentile), reported for all temperature (Bio1, 5, 6, 8–11) and precipitation (Bio12–14, 16–19) variables considered in the classification analysis.

	Moving window mean		Moving window SD	
Variable	<5%	>95%	<5%	>95%
Bio1	0****	158****	1***	13
Bio5	2***	93****	0****	40****
Bio6	0****	102****	20	0****
Bio8	1***	39****	10	5**
Bio9	0****	54****	17	9
Bio10	0****	170****	1***	33****
Bio11	0****	58****	4**	1***
Bio12	12	44****	2***	10
Bio13	15	18	3**	10
Bio14	8*	30****	11	32****
Bio16	16	44****	1***	24**
Bio17	17	30****	12	13
Bio18	15	16	10	13
Bio19	8*	13	9	6*

*P<0.05,

**P<0.01,

***P<0.001,

****P<0.0001.

Parks are quite heterogeneous in whether inter-annual variability is high (>95^th^ percentile) or low (<5^th^ percentile) on temperature or precipitation variables (Figure 5B). In terms of temperature, 43 parks (15%) exhibit extreme low inter-annual variability and 69 parks (24%) exhibit extreme high inter-annual variability. For precipitation, independent of temperature, 42 parks (15%) exhibit extreme low inter-annual variability and 76 parks (26%) exhibit extreme high inter-annual variability. Excluding the 108 parks (37%) that are not extreme with respect to either temperature or precipitation inter-annual variability, the maximum difference in percentile across moving windows is less than 10% in 91% of the remaining parks (Figure 5B).

A significantly small number of parks exhibit extreme low temperature inter-annual variability on four variables: Bio5 (0 parks), Bio1 and 10 (1 park each, <1%), and Bio11 (4 parks, 1%; Table 2). For high temperature inter-annual variability, multiple variables exhibit both significantly small and large numbers of extreme parks. For example, fewer parks than expected have high temperature inter-annual variability on Bio6 (0 parks) and Bio8 (5 parks, 2%), while a significantly large number of parks have high inter-annual variability on Bio5 (40 parks, 14%) and Bio10 (33 parks 11%). Patterns of inter-annual variability for precipitation are similar to temperature. A significantly small number of parks exhibit low precipitation inter-annual variability for Bio12 (2 parks, 1%), Bio13 (3 parks, 1%), and Bio16 (1 park, <1%). For high precipitation inter-annual variability, a significantly small number of parks are extreme for Bio19 (6 parks, 2%), while significantly large numbers of parks are extreme for Bio14 (32 parks, 11%) and Bio16 (24 parks, 8%). The 11 ‘mixed’ category parks for inter-annual variability include 8 parks with high and low extremes of temperature and 3 parks with high and low extremes of precipitation.

Finally, we highlight parks from the classification analysis that exemplify particular cases of extreme temperature or precipitation and are supported by maximum differences in percentile that are less than 5%. Parks that exhibit extreme high temperatures and high temperature inter-annual variability include Mammoth Cave National Park in Kentucky and Appomattox Court House National Historical Park in Virginia. Examples of parks that also possess extreme high temperatures but low temperature inter-annual variability include Niobrara National Scenic River in Nebraska and Fort Larned National Historic Site in Kansas. Relative to HRV, pronounced warming characterizes these four parks, but they differ in terms of whether recent extreme warm temperatures have been more or less variable. In terms of precipitation, an example of an extreme wet park with high precipitation inter-annual variability is Chesapeake & Ohio Canal National Historical Park in Washington DC, Maryland, and West Virginia. At the other extreme, Pu’ukohola Heiau National Historic Site in Hawaii is extreme dry with low precipitation inter-annual variability. Parks that have been experiencing extreme warm and dry climates include Kalaupapa National Historical Park in Hawaii, Mojave National Preserve in southern California, and Lake Mead National Recreation Area in Nevada and Arizona. Parks that are extreme warm and wet include Cape Lookout National Seashore in North Carolina, Florissant Fossil Beds National Monument in Colorado, and Delaware Water Gap National Recreation Area in New Jersey and Pennsylvania. These examples represent different categories of extreme climate and may offer insights for understanding how park resources are responding to ongoing changes in climate.

## Discussion

Natural resource parks in the US NPS exhibit complex patterns of exposure to climate change when their recent climates are evaluated relative to their 1901–2012 HRV. However, some regional patterns are clearly identified and corroborated by the literature: parks in the desert southwest are warmer and drier [27]; parks in Hawaii are warmer and drier [28]; parks in the northeast are warmer and wetter [29]; parks in the Midwest are warmer [30]; and parks in the southeast exhibit signs of the ‘warming hole’ [31]. Parks are overwhelmingly at the extreme warm end of historical temperature distributions and this is true for several variables, including annual mean temperature (Bio1), minimum temperature of the coldest month (Bio6), and mean temperature of the warmest quarter (Bio10). Precipitation patterns are more heterogeneous across parks and variables. For example, relative to HRV, precipitation may presently be high during the wettest month (Bio13) and low during the driest month (Bio14) (e.g., Jean Lafitte National Historical Park and Preserve in Louisiana). Climate variability did not show strong or consistent trends, i.e., recent inter-annual variability is generally within the range observed since 1901. Combined, these results suggest that many natural resource parks are already experiencing extreme climatic conditions with respect to their HRV, but these patterns vary across parks, climate variables, summary statistics (temporal mean or SD), and time windows of analysis.

Species within national parks are experiencing extreme climates and measurable plant and animal responses to recent climate change have already been documented [32]–[35]. The complexity of climate change shown here will likely be mirrored by species and other resources responding to different climate drivers. For example, temperate tree species in the Great Lakes region appear to be responding to summer temperature, while white-tailed deer are more sensitive to winter conditions [36], [37]. Additionally, species adapted to more extreme precipitation regimes may show positive responses to warming temperatures, whereas mesic species may show increased stress [38]. These differential responses are likely to cause natural communities to disassemble and novel communities to form [39], [40]. Climate complexity – as well as complex interactions between climate and other global change agents [5] – require that land managers possess tremendous scientific knowledge about the ecosystems under their jurisdiction, and develop coherent and achievable resource management goals.

Hansen et al. [9] identify HRV and DFC as different management philosophies necessitated by the degree to which past, present, and future ecological conditions overlap. Parks like Grand Canyon National Park, with natural resources that are sensitive to temperature (e.g., annual mean temperature, Figure 1), may require DFC management strategies because their temperature trajectories lead to a complete decoupling of past, present, and future conditions. Such a decision, however, should be resource specific, and especially in the case of culturally significant natural resources (e.g., cultural landscapes, seascapes, glaciers, and iconic organisms) may consider resistance as a viable, short-term management realization of DFC. For example, the General Sherman giant sequoia in Sequoia National Park is an iconic organism of cultural significance that – should it be threatened directly or indirectly by climate change through drought or fire – could receive intensive management intervention (i.e., it could be proactively maintained within its HRV). As climate shifts further outside of HRV bounds, resistance strategies will likely become less effective and extremely difficult management decisions – with input from the public and stakeholder groups – will be required. Indeed, because US national parks are for the benefit and enjoyment of the people [41] (the current motto is “Experience Your America”), many park resources will be subject to these decisions requiring compromise solutions that weigh results emerging from climate science against a suite of public values and socioeconomic considerations.

Estimates of future climate are – to varying degrees – uncertain, especially for particular variables considered in our analysis (e.g., cloud cover, vapor pressure) [42], [43]. Some CRU variables (e.g., cloud cover, vapor pressure, number of frost and wet days) are also more uncertain than others (e.g., temperature and precipitation, used to derive bioclimatic variables), owing to a comparably sparse record of observation and the need to initially model and interpolate at a coarser resolution [16]. Nevertheless, even considering just temperature and precipitation, climate projections for the 21^st^ century suggest many park geographies will become warmer and either drier or wetter [44]. Regionally, these predictions generally follow the same patterns and trends seen in our results. In other words, climate change is ongoing, and parks are already experiencing changes that can be documented without having to necessarily look out an additional 50 or 100 years. Importantly, future changes in temperature and precipitation will likely push many parks beyond the limits of their HRV. For example, recent mean temperature of the warmest quarter (Bio10) at Apostle Islands National Lakeshore in Wisconsin has already reached its 100^th^ percentile, meaning that any continued increase in temperature will push the park higher than all warm quarter temperatures it has experienced since 1901. Similarly, many parks are already extreme dry or wet; if these observed extremes are followed by future changes in the same direction, then affected parks will experience precipitation regimes unlike any they have seen in over a century. Furthermore, changes in some climate variables may exacerbate or override shifts in other variables. For example, extreme warm temperatures will cause considerably drier conditions, even if precipitation amounts remain within HRV. Although it is reasonable to expect HRV to increase with time, and indeed there may be real biological, ecological, or cultural reasons to consider longer periods [12], other practical management and planning considerations affect the determination of a meaningful HRV baseline. The year 1901 predates the establishment of the NPS by over a decade [41] and now many parks are transcending all climates they have experienced during their entire tenure. In this sense, 1901–2012 represents a reasonable baseline, and HRV analyses based on it suggest that the NPS is entering a new era of change. Furthermore, given that recent temperatures may be extreme even compared to longer baselines [11], the present analyses likely offer conservative estimates of temperature extremes in parks.

Management and planning decisions will be greatly informed by an overall assessment of vulnerability that integrates the present climate change exposure analyses with others that consider both the sensitivity and adaptive capacity of park resources to climate change [9], [45]–[48]. Beyond climate, additional change agents with additive and interactive effects (e.g., land use, pollution, biological invasions, fire) could also be profitably included as other analyses of exposure [49]. Here, a principal challenge will be developing vulnerability models that can be parameterized at the desired spatial and temporal scales of analysis. Given tradeoffs between the extent and resolution characteristics of scale (e.g., a long time series (extent) may be restricted to a monthly instead of daily time step (resolution)), vulnerability models will be subject to some of the classical model tradeoffs involving realism, precision, and generality [50]. Although there is an unstated desire in vulnerability assessments to maximize realism and precision at the cost of generality (i.e., collect site-level data and develop highly parameterized models that predict ecological complexity), we draw attention to the inability of these models to encompass broad geographic extents and suggest that vulnerability models maximizing generality and realism at the cost of precision stand to play an especially pivotal role in planning and policy.

Our measures of climate change exposure support broad-scale vulnerability assessments in the NPS, but they also help individual parks understand and interpret which of their major climate drivers are beginning to approach the limits of 1901–2012 HRV. Such insights facilitate discussion of how climate change may impact diverse park resources and values (e.g., natural resources, cultural resources, facilities, visitor experience, wilderness character), which are often sensitive to different measures of exposure, but they are not intended to replace more detailed analyses required for site-level management and planning within park boundaries. Many parks are in areas of complex topography and indeed this heterogeneity has important implications for climate adaptation [51], [52]. We suggest that the present analyses may be used to prioritize and select climate variables for more park-specific analyses of meso- and topo-climatic exposure. This ‘coarse filter’ approach is valuable because it allows parks to initiate resource-specific assessments with *a priori* knowledge of how major climate drivers have changed in recent time. For resources influenced by climate variables that are presently well within the bounds of HRV, parks might decide that limited funding should be redirected to study other resources that are sensitive to variables approaching the limits of HRV. Parks might also decide to monitor variables with recent extreme percentiles to assess rates of change and detect when and where they become ‘novel’ with respect to 1901–2010 HRV. Beyond applications related to monitoring and management, results offer park interpreters scientific information that may be translated and communicated to the public.

In approaching the 100-year anniversary of its creation in 2016, the NPS is poised to enter its next century of natural resource stewardship and science. The new century brings new challenges in terms of stewarding park resources in the face of environmental drivers that operate beyond park boundaries. Climate change further challenges us to develop new, ecologically viable desired conditions to guide the preservation of park resources in this new era of change. While such challenges remain paramount, more integrative research and education in the climate and landscape arenas will contribute to solutions.

## Supporting Information

Appendix S1 **Mean percentile and maximum difference in percentile (in parentheses) for moving window means: annual mean temperature (Bio1), mean diurnal range (Bio2), isothermality (Bio3), temperature seasonality (Bio4), maximum temperature of the warmest month (Bio5), and minimum temperature of the coldest month (Bio6).**
(PDF)

Appendix S2 **Mean percentile and maximum difference in percentile (in parentheses) for moving window means: temperature annual range (Bio7), mean temperature of the wettest quarter (Bio8), mean temperature of the driest quarter (Bio9), mean temperature of the warmest quarter (Bio10), and mean temperature of the coldest quarter (Bio11).**
(PDF)

Appendix S3 **Mean percentile and maximum difference in percentile (in parentheses) for moving window means: annual precipitation (Bio12), precipitation of the wettest month (Bio13), precipitation of the driest month (Bio14), precipitation seasonality (Bio15), precipitation of the wettest quarter (Bio16), precipitation of the driest quarter (Bio17), precipitation of the warmest quarter (Bio18), and precipitation of the coldest quarter (Bio19).**
(PDF)

Appendix S4 **Mean percentile and maximum difference in percentile (in parentheses) for moving window means: mean annual percentage cloud cover (Cld1), cloud seasonality (Cld4), vapor pressure of the warmest quarter (Vap18), annual number of wet days (Wet12), number of wet days of the warmest quarter (Wet18), and annual number frost days (Frs12).** NA’s associated with Frs12 indicate that the variable is undefined (i.e., no frost days exist).
(PDF)

Appendix S5 **Mean percentile and maximum difference in percentile (in parentheses) for moving window standard deviations: annual mean temperature (Bio1), mean diurnal range (Bio2), isothermality (Bio3), temperature seasonality (Bio4), maximum temperature of the warmest month (Bio5), and minimum temperature of the coldest month (Bio6).**
(PDF)

Appendix S6 **Mean percentile and maximum difference in percentile (in parentheses) for moving window standard deviations: temperature annual range (Bio7), mean temperature of the wettest quarter (Bio8), mean temperature of the driest quarter (Bio9), mean temperature of the warmest quarter (Bio10), and mean temperature of the coldest quarter (Bio11).**
(PDF)

Appendix S7 **Mean percentile and maximum difference in percentile (in parentheses) for moving window standard deviations: annual precipitation (Bio12), precipitation of the wettest month (Bio13), precipitation of the driest month (Bio14), precipitation seasonality (Bio15), precipitation of the wettest quarter (Bio16), precipitation of the driest quarter (Bio17), precipitation of the warmest quarter (Bio18), and precipitation of the coldest quarter (Bio19).**
(PDF)

Appendix S8 **Mean percentile and maximum difference in percentile (in parentheses) for moving window standard deviations: mean annual percentage cloud cover (Cld1), cloud seasonality (Cld4), vapor pressure of the warmest quarter (Vap18), annual number of wet days (Wet12), number of wet days of the warmest quarter (Wet18), and annual number frost days (Frs12).** NA’s associated with Frs12 indicate that the variable is undefined (i.e., no frost days exist).
(PDF)
