# Supplementary material for: Climate Exposure of US National Parks in a New Era of Change
Source: PLoS One. 2014 Jul 2;9(7):e101302. doi: 10.1371/journal.pone.0101302 (PMC4079655; doi:10.1371/journal.pone.0101302)
Supplement: Appendix S1 — Mean percentile and maximum difference in percentile (in parentheses) for moving window means: annual mean temperature (Bio1), mean diurnal range (Bio2), isothermality (Bio3), temperature seasonality (Bio4), maximum temperature of the warmest month (Bio5), and minimum temperature of the coldest month (Bio6). (PDF) [file pone.0101302.s001.pdf]

Appendix S1. Mean percentile and maximum difference in percentile (in parentheses) for moving window means: annual mean temperature (Bio1), mean diurnal range (Bio2), isothermality (Bio3), temperature seasonality (Bio4), maximum temperature of the warmest month (Bio5), and minimum temperature of the coldest month (Bio6).

Park	Bio1	Bio2	Bio3	Bio4	Bio5	Bio6
Abraham Lincoln Birthplace National Historical Park	99.7 (1)	5.6 (5.1)	9.8 (3.1)	20.1 (40.3)	55.1 (12.5)	89.1 (26.2)
Acadia National Park	100 (0)	29.2 (28.1)	49.4 (49.2)	22.4 (46.4)	98.6 (2.9)	95.5 (13.6)
Agate Fossil Beds National Monument	98 (3.2)	65.1 (39.9)	74.6 (11.5)	38.9 (35.4)	97.7 (6.8)	97.7 (6.8)
Ala Kahakai National Historic Trail	77.8 (0.9)	7.5 (19.5)	15.9 (25)	78.1 (37.7)	67.3 (16.7)	96.2 (6.5)
Alagnak Wild River	80.4 (24.3)	69.2 (11.5)	53.2 (14.7)	49.4 (41.2)	77.9 (32.1)	45.7 (35.7)
Alibates Flint Quarries National Monument	92.8 (21.7)	64.8 (13.3)	88.8 (9)	10 (10.7)	71.6 (17)	97.1 (8.7)
Allegheny Portage Railroad National Historic Site	73.5 (19.2)	1.1 (0.2)	20.1 (30.1)	17.8 (32.8)	37.9 (7)	84.5 (19.6)
American Memorial Park	93.5 (10.8)	83.6 (17.6)	66.9 (12.6)	46.3 (25.7)	75.5 (15.6)	81.1 (3.5)
Amistad National Recreation Area	98.7 (3.9)	98.1 (5.8)	95.2 (5.6)	15.6 (5.2)	96.8 (9.7)	89.6 (19.7)
Anacostia Park	98.6 (4.3)	6 (7.6)	23.9 (27.6)	39.6 (45.3)	81.4 (19.5)	83.8 (17.8)
Aniakchak National Monument and Preserve	79.7 (23.1)	70.6 (21.9)	50.8 (16.4)	60.9 (50)	93.6 (3.5)	40 (45.4)
Antietam National Battlefield	67.6 (34.3)	1.4 (1.2)	19.2 (25.3)	22.3 (47.3)	34.7 (21.2)	68.2 (18.1)
Apostle Islands National Lakeshore	99 (1.9)	2.4 (2.7)	3.1 (6.3)	16.7 (34.7)	74 (41.2)	89.4 (24.3)
Appalachian National Scenic Trail	100 (0)	1.8 (0.9)	21.4 (27.6)	18 (37.6)	70.8 (15)	91.8 (21.4)
Appomattox Court House National Historical Park	98 (4.8)	7 (4.3)	7.3 (6.5)	26 (44.2)	77.9 (37.4)	79.2 (18.6)
Arches National Park	88.4 (17)	99 (2.9)	91.5 (24.3)	32.4 (70.5)	99 (2.9)	82.2 (41.6)
Arkansas Post National Memorial	67.5 (17.4)	4.4 (7.5)	7.1 (8.5)	55 (16.6)	69.1 (9.1)	97.9 (2.8)
Assateague Island National Seashore	100 (0)	1.7 (1.8)	7.3 (3.7)	88.3 (25.5)	100 (0)	87.8 (29.1)
Aztec Ruins National Monument	97.4 (7.8)	54.3 (53.8)	66.3 (20.3)	34.1 (54)	99.7 (1)	91.4 (22.3)
Badlands National Park	79.3 (13.4)	79.8 (15.4)	87.4 (19.7)	28 (49.1)	84.5 (6.7)	98.1 (5.8)
Baltimore-Washington National Parkway	100 (0)	26.2 (30.1)	30 (27.1)	45 (45.5)	94.2 (8.7)	81.9 (16.3)
Bandelier National Monument	99.7 (1)	6.5 (11.4)	37.3 (26)	11.4 (15)	80.1 (25)	94.8 (10.7)
Bent's Old Fort National Historic Site	100 (0)	80.2 (22.8)	80.9 (27.5)	34 (40.9)	97.1 (4.8)	91.9 (14.1)
Bering Land Bridge National Preserve	93.2 (8.6)	21.8 (2.1)	24.6 (7.5)	43.9 (67.3)	84 (9.8)	84.2 (12.5)
Big Bend National Park	100 (0)	83.9 (19.7)	84.8 (15.8)	52.2 (26)	99.4 (1.9)	93.4 (11.5)
Big Cypress National Preserve	99.2 (1.2)	3 (5.7)	20.8 (31.3)	63.2 (37.4)	94.7 (3.9)	87.1 (30.1)
Big Hole National Battlefield	99 (2.2)	25.1 (18.9)	88 (34)	9.4 (21.3)	95.7 (1.8)	97.7 (6.8)
Big South Fork National River and Recreation Area	76 (29.9)	4.5 (5.4)	23.1 (25.2)	20.8 (40.3)	45.2 (21)	90.7 (21.4)
Big Thicket National Preserve	100 (0)	92.4 (22.9)	68 (52)	70.7 (17.9)	70.5 (43.8)	47.6 (11.5)
Bighorn Canyon National Recreation Area	97.6 (5.4)	28.9 (41.9)	69.8 (3.5)	11.3 (14.8)	96.7 (8.7)	98.7 (3.9)
Biscayne National Park	97.9 (1.7)	13.2 (29)	14.9 (17.9)	77.9 (26.2)	97.9 (2)	86 (30.1)
Black Canyon Of The Gunnison National Park	91.7 (11.3)	100 (0)	94.7 (14.6)	33.5 (56.4)	99.7 (1)	74.4 (41.8)
Blue Ridge Parkway	86.8 (20.4)	4.8 (5.4)	10 (8.6)	36.8 (52.7)	53.3 (16.9)	85.5 (35)
Bluestone National Scenic River	98.2 (4.3)	3.4 (4.6)	2.6 (1.7)	65.4 (34.1)	73.1 (18.9)	67.7 (28.8)
Booker T. Washington National Monument	99.6 (1.1)	5.1 (6.3)	5.7 (7.9)	18 (36.7)	44.4 (15.8)	95.3 (8.7)
Boston Harbor Islands National Recreation Area	100 (0)	1.8 (1.4)	19.5 (34.9)	29 (51.2)	100 (0)	93.5 (19.4)

Park	Bio1	Bio2	Bio3	Bio4	Bio5	Bio6
Bryce Canyon National Park	98.4 (4.9)	52 (26.9)	85.8 (37.9)	31.7 (33.2)	99.7 (1)	95.2 (9.7)
Buck Island Reef National Monument	89.2 (17.5)	52.6 (27.2)	43.8 (3.4)	93.1 (11.8)	89.2 (17.5)	93 (14.4)
Buffalo National River	91.9 (16.1)	7.5 (10.9)	10.1 (1.2)	22.7 (45.9)	65.5 (13.8)	96.7 (7.8)
Cabrillo National Monument	73.2 (9.4)	3.7 (7.7)	9.5 (7.1)	24.3 (17.9)	53.5 (15.3)	93.6 (8.6)
Canaveral National Seashore	89 (23.3)	94.5 (9.7)	86.3 (29.1)	65 (16)	88.3 (20.4)	49 (36.7)
Canyon De Chelly National Monument	97.4 (7.8)	35.8 (41.4)	63.8 (22.2)	31 (52.4)	100 (0)	92.4 (15.5)
Canyonlands National Park	91.6 (15.3)	100 (0)	93.5 (19.4)	38.5 (72.3)	99 (2.9)	86.8 (30.1)
Cape Cod National Seashore	100 (0)	80.5 (36.7)	47.6 (58.9)	73.2 (28.6)	100 (0)	93 (16.4)
Cape Hatteras National Seashore	97.3 (4.9)	3.3 (6.7)	7.3 (15.8)	67.3 (41.6)	90.1 (16.1)	83.2 (40.8)
Cape Krusenstern National Monument	94.2 (9.6)	12.1 (4.3)	3.6 (3.4)	36 (64.4)	90.4 (8.4)	81.2 (16.1)
Cape Lookout National Seashore	97.6 (4.3)	1.7 (1.8)	9.9 (17.6)	44.4 (51.1)	88.3 (21.5)	82.8 (39.8)
Capitol Hill Parks	98.6 (4.3)	5.6 (7.6)	23.2 (27.4)	38.9 (45.3)	80.7 (21.7)	83.1 (17.5)
Capitol Reef National Park	97.4 (7.8)	83.5 (14.6)	85.6 (35.9)	37.8 (54.7)	99 (2.9)	92.4 (15.5)
Capulin Volcano National Monument	100 (0)	41.7 (57.4)	54.8 (49.3)	36.9 (57.8)	100 (0)	92.9 (11.2)
Carl Sandburg Home National Historic Site	65.6 (9.7)	9.6 (10.9)	16.7 (8.8)	46.6 (41)	56.8 (8.5)	75.6 (29.7)
Carlsbad Caverns National Park	100 (0)	5.6 (5.5)	28.6 (10.2)	41.7 (37.2)	96.3 (8.7)	96.8 (1.7)
Casa Grande Ruins National Monument	100 (0)	4.1 (4.4)	3.8 (3.4)	89.6 (1.1)	99.7 (1)	98 (4.8)
Castillo De San Marcos National Monument	87.9 (22.3)	98.4 (4.8)	66 (21.3)	67.4 (18.4)	90.1 (17.2)	56.9 (67.3)
Catoctin Mountain Park	75.6 (29.6)	5 (10.6)	32.5 (25.9)	25.7 (43)	49.4 (15.6)	65.6 (13.3)
Cedar Breaks National Monument	99 (2.9)	22.8 (16.8)	66.7 (61)	51.5 (50.6)	99.7 (1)	96.7 (8.7)
Chaco Culture National Historical Park	98.7 (3.9)	8.9 (13.2)	51.2 (31)	17.4 (34.7)	100 (0)	95.3 (11.7)
Channel Islands National Park	81.7 (17.2)	1.1 (0.2)	32.8 (13.5)	1.7 (1.8)	18.6 (4.3)	99.6 (1.2)
Charles Pinckney National Historic Site	89.3 (9.6)	39.5 (58.6)	30 (7.3)	60.9 (32.1)	96.9 (5.4)	58.2 (23.8)
Chattahoochee River National Recreation Area	100 (0)	9.7 (8.3)	22.7 (23.5)	21.8 (41)	96.7 (8.7)	80 (23.4)
Chesapeake and Ohio Canal National Historical Park	77.1 (28)	1.4 (1.2)	11.8 (18.8)	21.7 (44.4)	38.2 (21.2)	78.3 (24.1)
Chickamauga and Chattanooga National Military Park	87.1 (29.4)	4.5 (5.4)	30.5 (41.4)	44.7 (31.8)	73.2 (18.7)	67.9 (21.4)
Chickasaw National Recreation Area	95.2 (14.5)	55.8 (35.9)	83.9 (2.7)	23.6 (30.9)	76.8 (24.4)	88.8 (27.7)
Chiricahua National Monument	100 (0)	2.5 (2.5)	2.1 (2.8)	91.6 (10.4)	100 (0)	98.8 (2.4)
City Of Rocks National Reserve	92 (16.5)	38.7 (19.2)	68.6 (34)	36.9 (43.5)	71.6 (32.2)	96.4 (10.7)
Colonial National Historical Park	100 (0)	3.7 (6.6)	14.2 (14.9)	37.5 (53.7)	97.6 (4.3)	92.5 (19.4)
Colorado National Monument	85.9 (17)	100 (0)	96.1 (11.7)	38.2 (64.9)	99 (2.9)	71.1 (61.9)
Congaree National Park	59.1 (13.2)	32.1 (34.1)	36.5 (4.4)	55.6 (20.5)	44.4 (19.5)	51.3 (22.6)
Coronado National Memorial	100 (0)	20.7 (28.9)	20.8 (30.3)	74.5 (21.1)	99.4 (1.9)	100 (0)
Cowpens National Battlefield	86 (9.6)	52.7 (20.5)	62.2 (7.3)	19.8 (38.1)	62.6 (10.5)	78.9 (40.6)
Crater Lake National Park	95.6 (6.8)	1.1 (0.2)	1.1 (0.2)	43.6 (35.1)	34 (53.9)	96.7 (8.7)
Craters Of The Moon National Monument and Preserve	93.1 (8.3)	47.1 (12.2)	93.5 (19.4)	29.3 (41.5)	70.7 (28.6)	97.3 (6.8)
Cumberland Gap National Historical Park	77.8 (26.5)	4.1 (6.6)	8.6 (9.6)	45.8 (35.2)	40.7 (23.7)	85.9 (16.1)
Cumberland Island National Seashore	91.3 (16.5)	99.6 (1.1)	78.8 (37.4)	71.7 (18.9)	89.1 (16.5)	46.2 (58)
Curecanti National Recreation Area	92.7 (6.8)	100 (0)	92.6 (18.4)	29 (41.3)	99.7 (1)	78.7 (38.1)
Cuyahoga Valley National Park	99 (2.9)	29.9 (17.1)	56.5 (27.4)	20.6 (44.4)	55 (5.4)	69.6 (40.8)

Park	Bio1	Bio2	Bio3	Bio4	Bio5	Bio6
Death Valley National Park	99.4 (1.9)	1.1 (0.2)	11 (17.3)	32.1 (35.6)	96.2 (8.6)	98.3 (3.9)
Delaware Water Gap National Recreation Area	100 (0)	15.2 (6.6)	35.6 (23.7)	18.7 (39.6)	98.4 (4.8)	90.4 (22.3)
Denali National Park and Preserve	93.7 (6.5)	18.7 (20.9)	22.4 (35.4)	51.7 (36.7)	70.1 (34.8)	91.3 (17)
Devils Postpile National Monument	99 (2.9)	1.5 (1.4)	2.2 (0.5)	87.6 (7.2)	95.6 (4.1)	96.1 (6.8)
Devils Tower National Monument	82.6 (21.7)	98 (3.2)	94.9 (7.8)	17 (26.2)	93.4 (7.1)	94.4 (9.8)
Dinosaur National Monument	88.2 (30.1)	63.7 (42.8)	88.8 (25.2)	32.6 (61.6)	91.2 (20.5)	86.1 (32)
Dry Tortugas National Park	86.4 (19.7)	1.1 (0.2)	12.4 (16.4)	78 (33)	61.2 (10.5)	78.8 (49.5)
Ebey's Landing National Historical Reserve	86 (10.2)	7.8 (7)	23.9 (18.6)	27 (20.9)	93.4 (11.7)	94.9 (8.5)
Effigy Mounds National Monument	99.4 (1.9)	7.5 (10.9)	29.8 (17.9)	12.4 (24)	29.5 (47.6)	96.3 (6.8)
Eisenhower National Historic Site	75.9 (21.1)	25.1 (20.6)	43.5 (32.4)	30.4 (41.1)	57.1 (16.5)	60.5 (9.3)
El Malpais National Monument	99.7 (1)	5.2 (4.2)	32.7 (19.6)	15 (21.9)	68.1 (20.6)	96.3 (8.7)
El Morro National Monument	99.7 (1)	6 (4.4)	40.5 (20.4)	16.9 (27.7)	90.7 (10.3)	95.2 (9.7)
Eugene O'Neill National Historic Site	88.1 (8.3)	5.8 (9.5)	18.1 (11.6)	44 (10.9)	16.2 (23)	96.7 (6.7)
Everglades National Park	97.6 (2.7)	4 (8.6)	18.1 (26.2)	71.2 (34.4)	96.4 (1.4)	83.4 (36.9)
Fire Island National Seashore	100 (0)	4.2 (4.6)	17.9 (29.9)	77.8 (40.2)	100 (0)	91.5 (22.3)
Florissant Fossil Beds National Monument	100 (0)	95.9 (8.4)	90.7 (24.3)	30 (26.2)	100 (0)	93.9 (14.6)
Fort Bowie National Historic Site	100 (0)	2.5 (2.5)	2.1 (2.8)	92.4 (12.4)	99.4 (1.9)	98.8 (2.4)
Fort Caroline National Memorial	90.9 (17.5)	100 (0)	67.3 (14)	69.2 (17.9)	89.8 (16.1)	51.4 (63)
Fort Davis National Historic Site	100 (0)	40.9 (3.3)	46.8 (21.4)	66 (26.4)	96.7 (6)	93.1 (16.3)
Fort Donelson National Battlefield	69.8 (32.9)	7.4 (12.2)	17.1 (9.3)	18 (37.6)	52 (26.1)	70.7 (28)
Fort Frederica National Monument	93 (13.6)	100 (0)	77.9 (43.5)	72.7 (20.8)	89.5 (16.5)	47.9 (57.2)
Fort Laramie National Historic Site	98 (3.2)	94 (16.1)	89.7 (22.3)	33.5 (15.2)	98.4 (4.9)	97.7 (6.8)
Fort Larned National Historic Site	89.6 (9.6)	82.7 (13.2)	92.8 (0.7)	11.6 (16.8)	62.5 (35.8)	97.9 (2.8)
Fort Matanzas National Monument	84 (24)	95.6 (13.3)	63.9 (21.1)	66.7 (18.6)	89.7 (18.3)	56.6 (68.3)
Fort Moultrie National Monument	91.8 (10.1)	43.8 (57.9)	28.8 (2.9)	61.3 (29.9)	96.9 (3.1)	57.5 (23.5)
Fort Necessity National Battlefield	86.3 (12.2)	1.7 (1.8)	4.6 (9.8)	19 (39.6)	30.1 (6.7)	83.3 (25.5)
Fort Point National Historic Site	91.2 (14.3)	1.7 (1.8)	12.7 (3.8)	53.9 (11.7)	56.1 (8.6)	97.7 (5.8)
Fort Pulaski National Monument	82.9 (11.7)	91.1 (12)	82.3 (37.2)	69.3 (25.7)	92.2 (13.6)	51 (32.1)
Fort Raleigh National Historic Site	98.6 (2.2)	3.7 (7.7)	7.5 (11.2)	67.7 (41.6)	93.4 (12.9)	85.5 (35)
Fort Sumter National Monument	92.2 (7.9)	41.6 (60.3)	26.8 (8)	62.3 (30.9)	96.9 (5.4)	59 (25)
Fort Union National Monument	100 (0)	5.3 (7.7)	64.5 (24)	17.4 (20.5)	94 (18.1)	97.3 (6.8)
Fort Union Trading Post National Historic Site	85.8 (22.3)	52.9 (24.1)	93.9 (9.7)	13.2 (21.1)	76.6 (15.5)	98.3 (3.2)
Fort Vancouver National Historic Site	82.4 (27.9)	1.1 (0.2)	1.1 (0.2)	67.9 (24.6)	24.7 (34.6)	93.7 (14.5)
Fort Washington Park	98.2 (5.4)	1.4 (1.2)	19.9 (25.7)	39 (43.1)	79.8 (20.3)	85.5 (15.8)
Fossil Butte National Monument	98.7 (3.9)	8.8 (17.3)	30.7 (59.6)	37.4 (49.2)	82.9 (26.5)	97.1 (8.7)
Frederick Douglass National Historic Site	98.6 (4.3)	4.1 (5.1)	23.2 (27.4)	38.9 (45.3)	80.3 (22.7)	83.8 (17.8)
Fredericksburg and Spotsylvania National Military Park	83.4 (26.9)	1.1 (0.2)	7.7 (13.7)	47.4 (47.5)	75.1 (28.7)	85.3 (20.9)
Friendship Hill National Historic Site	93.3 (6.5)	3.4 (5.7)	8.5 (18.5)	24.5 (45.2)	46.3 (8.3)	82.5 (25.5)
Gates Of The Arctic National Park and Preserve	98 (4.9)	31 (5.3)	47.2 (21.7)	43.4 (44.3)	86.9 (23.3)	93 (8.4)
Gateway National Recreation Area	100 (0)	4.1 (6.6)	20.7 (28.4)	47.7 (59.3)	100 (0)	91.4 (21.4)

Park	Bio1	Bio2	Bio3	Bio4	Bio5	Bio6
Gauley River National Recreation Area	99.3 (2.2)	16.5 (2.4)	21 (26.7)	41.4 (45)	76.6 (20.3)	71.4 (31.2)
George Washington Birthplace National Monument	95.8 (8.4)	1.4 (0.9)	12.9 (20.2)	42.5 (45)	87.3 (13.6)	91.5 (22.3)
George Washington Carver National Monument	90 (18.5)	47.2 (44.1)	46.2 (21.1)	19.4 (38.1)	73.1 (20.8)	82.9 (11.6)
George Washington Memorial Parkway	91.1 (19.4)	1.4 (1.2)	20.4 (27.9)	36.5 (41.6)	73.5 (24.3)	82 (18.3)
Gettysburg National Military Park	81.5 (15.2)	28.4 (28.1)	45.2 (31.4)	29.7 (41.3)	58.9 (11.2)	61.9 (7.1)
Gila Cliff Dwellings National Monument	100 (0)	4.8 (6.3)	24.4 (30.3)	23.4 (16.6)	86.6 (21)	99.7 (1)
Glacier Bay National Park and Preserve	88.1 (17.5)	17.2 (8.1)	50.1 (11.1)	16.1 (30.8)	36.6 (39.4)	97.3 (5.8)
Glacier National Park	85.8 (20.2)	39.4 (11.9)	99.4 (1.9)	3.8 (4.2)	42.1 (34.2)	99.7 (1)
Glen Canyon National Recreation Area	97.1 (8.7)	99.4 (1.9)	90.3 (24.3)	32.6 (60.2)	99 (2.9)	92.1 (16.5)
Golden Gate National Recreation Area	88.4 (16.3)	2.4 (3.8)	16.2 (10.9)	55.9 (9.8)	56.7 (7.6)	97 (7.8)
Golden Spike National Historic Site	92.7 (16.5)	5.9 (6.5)	41.1 (54.6)	33.5 (58.6)	92.1 (12.4)	96.3 (8.7)
Grand Canyon National Park	99 (2.9)	7.2 (3.1)	34.3 (29.9)	27.3 (31.4)	99.7 (1)	96.2 (7.8)
Grand Portage National Monument	97.4 (6.8)	1.1 (0.2)	8.3 (21.9)	22.8 (44.9)	69.7 (6.8)	86.2 (34)
Grand Teton National Park	92.5 (10)	40.5 (48.9)	89.9 (19.4)	17.3 (25.4)	91.3 (4)	95.6 (10.7)
Grant-Kohrs Ranch National Historic Site	99 (1.9)	64 (65.9)	83.9 (30.4)	6.6 (15.3)	98.7 (3.9)	99 (2.9)
Great Basin National Park	98.4 (4.9)	46.7 (13.4)	61.8 (30.5)	56.7 (32.6)	98.7 (3.9)	95.7 (6.8)
Great Sand Dunes National Park and Preserve	99.4 (1.9)	86.7 (20.5)	89.4 (16.1)	24 (51.1)	100 (0)	90.8 (20.4)
Great Smoky Mountains National Park	59.4 (11.6)	3.7 (5.6)	6.5 (12.3)	46.9 (40.5)	37.8 (14.6)	88.1 (29.1)
Greenbelt Park	99.6 (1.1)	21.1 (27.9)	28.9 (27.1)	41.4 (46.2)	89.6 (14.8)	81.3 (18.3)
Guadalupe Mountains National Park	100 (0)	19.3 (19.2)	21.5 (20.6)	59.7 (35.9)	94.8 (10.7)	98.9 (0.2)
Guilford Courthouse National Military Park	100 (0)	4.9 (4.4)	14.6 (9.5)	14.9 (24.5)	85.8 (16.4)	95 (9.7)
Gulf Islands National Seashore	73 (22.1)	38.2 (9)	58.2 (5.1)	59.1 (22.2)	81 (10.3)	55.3 (17.1)
Hagerman Fossil Beds National Monument	99.7 (1)	30 (27.8)	73.8 (45.8)	46.3 (37.5)	81.6 (13.2)	97.4 (7.8)
Haleakala National Park	81.9 (8.4)	8.1 (13.4)	12.2 (18.9)	76.7 (19.3)	65.4 (6.5)	98.8 (3.6)
Harpers Ferry National Historical Park	63 (34.9)	1.4 (1.2)	11.8 (19.7)	22 (46.1)	32.1 (21.4)	67.5 (18.1)
Hawaii Volcanoes National Park	76.5 (3.6)	20.9 (33.5)	18.1 (25.8)	80.1 (34.8)	67 (18.6)	90.9 (14)
Herbert Hoover National Historic Site	98.7 (2.9)	7.9 (10.3)	32 (10.3)	9.7 (18.2)	21.3 (26.7)	94.7 (11.7)
Homestead National Monument of America	82.5 (9.9)	88.6 (22.6)	97.9 (5.4)	4.6 (7.4)	10.9 (6.4)	86.5 (11.8)
Hopewell Culture National Historical Park	60.8 (43.3)	3.1 (4.6)	24.8 (31.8)	19 (39.6)	23.4 (16)	80.8 (23.1)
Hopewell Furnace National Historic Site	100 (0)	92.3 (17.4)	80.7 (32.3)	17.7 (36.7)	97.7 (5.8)	83.7 (25.7)
Horseshoe Bend National Military Park	62.6 (24.9)	33 (19.1)	16.7 (20.9)	31.6 (48.3)	55 (26.3)	61.5 (9.3)
Hot Springs National Park	85.2 (34)	5.1 (6.3)	5.3 (7.7)	28.6 (42.1)	59 (14.7)	99.4 (1.9)
Hovenweep National Monument	97.4 (7.8)	100 (0)	93.2 (20.4)	40.5 (61.5)	99.7 (1)	80.7 (32.6)
Hubbell Trading Post National Historic Site	98.1 (5.8)	24.6 (24.8)	50.9 (34)	30.5 (43.2)	100 (0)	92.1 (14.5)
Indiana Dunes National Lakeshore	82.4 (17.4)	3.1 (2.7)	3.9 (2.7)	18.3 (40.5)	15.2 (11.7)	83.3 (34.7)
Isle Royale National Park	97.4 (6.8)	1.1 (0.2)	3.5 (7.5)	29.4 (60.2)	71.6 (16.6)	84.3 (41.7)
Jean Lafitte National Historical Park and Preserve	100 (0)	3.4 (5.6)	10.6 (13.3)	74 (16.5)	99 (1.9)	92.2 (17.3)
Jewel Cave National Monument	68.6 (26.7)	98.7 (2.9)	86 (28.4)	60 (25.6)	93 (8.4)	67.1 (18.1)
John Day Fossil Beds National Monument	87.4 (27.2)	16.1 (17.7)	58.7 (33.5)	13.7 (29.8)	84 (15.9)	95.4 (12.6)
John Muir National Historic Site	90 (10.7)	3.1 (4.7)	24.5 (20.1)	46.8 (13.2)	7 (16.4)	97 (7.8)

Park	Bio1	Bio2	Bio3	Bio4	Bio5	Bio6
Johnstown Flood National Memorial	67.1 (17.4)	1.1 (0.2)	11.9 (23.4)	16.8 (31.8)	34.9 (16.6)	86.3 (19)
Joshua Tree National Park	100 (0)	2.7 (4.7)	4 (3.9)	9.2 (15.2)	54.2 (57.2)	99.4 (1.9)
Kalaupapa National Historical Park	88.1 (7.4)	5.1 (12.3)	12.3 (8.9)	73.8 (19.6)	70.1 (7)	100 (0)
Kaloko-Honokohau National Historical Park	81.1 (6.9)	3.9 (8.7)	13.4 (24.8)	81.9 (23.8)	69.7 (15)	98.5 (2.4)
Katmai National Park and Preserve	80.1 (25.3)	61 (12.5)	43.6 (20.5)	49.1 (40.3)	77.5 (31.9)	42.5 (36.5)
Kenai Fjords National Park	77.5 (22.2)	2.2 (3.3)	1.5 (1.4)	20.2 (25)	8.6 (12.8)	84 (24.8)
Kennesaw Mountain National Battlefield Park	100 (0)	12.2 (11)	21 (17.3)	19 (39.3)	98.7 (3.9)	75.2 (19.3)
Kings Mountain National Military Park	71.2 (6.5)	71.3 (30.3)	73.9 (18.9)	19.1 (38.1)	62.5 (5.3)	63.3 (36.1)
Klondike Gold Rush National Historical Park	88.5 (18.7)	17.5 (20.4)	47.2 (22.3)	13.8 (26)	18.5 (32.5)	93.6 (10.7)
Knife River Indian Villages National Historic Site	95.2 (8.6)	11.5 (24.1)	86.9 (22.5)	10 (13.8)	71.9 (21.4)	99.4 (1.9)
Kobuk Valley National Park	97.2 (4.9)	10.4 (10.5)	7.4 (3.9)	29.4 (51.3)	95.2 (8.6)	92.6 (9.5)
Lake Clark National Park and Preserve	86.9 (13.9)	5.4 (4)	4.7 (1.5)	30.8 (41.6)	27.9 (4.8)	81.7 (30.6)
Lake Mead National Recreation Area	99.7 (1)	3.2 (1.5)	5.1 (2.1)	41.1 (43.6)	99.7 (1)	99.4 (1.9)
Lake Meredith National Recreation Area	93.6 (19.3)	70 (10.3)	90.8 (12.9)	10.4 (10.7)	73.3 (16)	97.1 (8.7)
Lake Roosevelt National Recreation Area	95.2 (8.6)	1.1 (0.2)	71.6 (63.6)	15.1 (28.6)	74.4 (23)	96.8 (9.7)
Lassen Volcanic National Park	97.6 (3.9)	2.2 (2.5)	2.2 (0.5)	67.4 (39.2)	59.7 (53.4)	97.7 (5.8)
Lava Beds National Monument	96.3 (7.8)	1.5 (1.4)	1.5 (1.4)	68.3 (38.8)	54.9 (47.1)	95.6 (8.6)
Lewis and Clark National Historical Park	79.3 (23.6)	13.2 (9.3)	61.7 (32.7)	24 (50)	43.5 (44.6)	95.7 (9.6)
Lincoln Boyhood National Memorial	100 (0)	4.4 (6.6)	2.5 (2.5)	14.7 (30.8)	36.6 (16.4)	94.5 (15.5)
Little Bighorn Battlefield National Monument	95.8 (8.6)	61 (21.4)	79.8 (8)	13 (16.5)	95.4 (11.7)	99 (2.9)
Little River Canyon National Preserve	67 (32)	5 (9.5)	15.2 (14.4)	25.6 (45.9)	46.5 (4.5)	62.2 (12.5)
Lyndon B. Johnson National Historical Park	72.9 (36.3)	63.1 (29.5)	70.2 (31.5)	9.9 (13.9)	49.1 (8.8)	79.5 (35.5)
Mammoth Cave National Park	94 (18.1)	5.6 (5.5)	9.8 (3.4)	23.1 (45.2)	48 (11.3)	86.3 (26.5)
Manassas National Battlefield Park	69.3 (44.2)	1.4 (1.2)	11.9 (20.9)	27.1 (43.3)	53.5 (25.4)	72.9 (17.5)
Manzanar National Historic Site	97.2 (3.1)	1.5 (1.4)	18.8 (13.9)	23.6 (32.6)	87.9 (28.9)	96.8 (3.8)
Marsh-Billings-Rockefeller National Historical Park	100 (0)	14.6 (19)	71.3 (38.2)	12.2 (29.9)	99.7 (1)	97.7 (6.8)
Mary McLeod Bethune Council House National Historic Site	97.8 (6.5)	4.4 (6.6)	23.2 (27.4)	39 (43.1)	80 (22.7)	82.7 (16.3)
Mesa Verde National Park	97.4 (7.8)	94.1 (13.3)	83.5 (17.7)	38.4 (57)	99.7 (1)	88.1 (22)
Minute Man National Historical Park	100 (0)	1.7 (1.8)	32.3 (42.1)	18.1 (35.7)	99.7 (1)	94.5 (16.5)
Mississippi National River and Recreation Area	99.7 (1)	2.4 (2)	16 (25.5)	12.1 (22.1)	72.8 (30.1)	98.3 (2.9)
Missouri National Recreational River	96.6 (6.5)	6.2 (8.4)	58 (27.3)	10.7 (11.9)	21 (23.1)	98.4 (4.9)
Mojave National Preserve	100 (0)	1.1 (0.2)	1.8 (0.9)	29.4 (30.5)	93.4 (13.5)	100 (0)
Monocacy National Battlefield	65.6 (48.6)	2.6 (3.8)	25.5 (31.8)	25.6 (46.1)	41.7 (28.2)	64.4 (14.1)
Montezuma Castle National Monument	99.4 (1.9)	21.4 (6)	46.8 (27)	38 (50.4)	98.9 (2.4)	96.5 (6.8)
Moores Creek National Battlefield	80.9 (19.2)	4.1 (5.6)	11.2 (7.3)	63.1 (30.7)	82.5 (10.4)	70.2 (17.9)
Morristown National Historical Park	100 (0)	23.7 (8.8)	30.1 (19.1)	18.6 (40.5)	100 (0)	91.1 (22.3)
Mount Rainier National Park	88.5 (20.4)	2.1 (2.8)	19.8 (48.7)	36.7 (37.3)	35.3 (52.2)	94.7 (14.6)
Mount Rushmore National Memorial	67.7 (29.1)	99 (2.9)	80.6 (19.1)	59.8 (30.9)	90.8 (5.2)	80.3 (15.9)
Muir Woods National Monument	89.1 (16.3)	1.7 (1.8)	17.7 (13.1)	56.3 (10.8)	57.2 (10)	97.3 (6.8)
Natchez Trace Parkway	64 (24.2)	4.8 (6.3)	11.1 (6.1)	56.2 (22.6)	55 (25.1)	66.3 (13.8)

Park	Bio1	Bio2	Bio3	Bio4	Bio5	Bio6
National Capital Parks-East	97.8 (6.5)	5.6 (7.6)	23.6 (28.6)	38.6 (44.3)	80.3 (22.7)	82.7 (16.3)
National Park of American Samoa	96.8 (9.7)	3.6 (7.6)	4.9 (5.6)	97.5 (2.5)	96.4 (9.7)	96.8 (9.7)
Natural Bridges National Monument	96.8 (9.7)	99.7 (1)	94.5 (16.5)	38.5 (64.7)	99 (2.9)	87.9 (29.1)
Navajo National Monument	97.4 (7.8)	86 (12)	87.1 (29.6)	33.1 (46.9)	99.7 (1)	91.7 (16.5)
New River Gorge National River	98.9 (3.2)	5 (9.5)	4.1 (3.1)	53.8 (37.8)	73.8 (19.8)	68.4 (28.8)
Nez Perce National Historical Park	96.5 (7.5)	5.3 (11)	48.7 (51.1)	23.6 (33.8)	74.3 (16.5)	96.7 (8.7)
Ninety Six National Historic Site	86 (19.5)	14.8 (25.6)	7.4 (8.8)	57.7 (25.6)	82.8 (39.8)	57.5 (17.9)
Niobrara National Scenic River	94.9 (8.6)	5.1 (7.3)	40.5 (19)	13.7 (18.7)	40.8 (51.1)	98.7 (3.9)
Noatak National Preserve	96.4 (6)	8.9 (12.2)	5 (3.9)	29.4 (51.3)	95.9 (6.5)	92.9 (8.5)
North Cascades National Park	90.1 (12.9)	11.4 (21.3)	37.1 (3.9)	18 (28.7)	69.6 (35.8)	96.4 (9.7)
Obed Wild and Scenic River	82.4 (29.9)	3.4 (5.7)	17.3 (22.6)	25.6 (45.2)	45.6 (21.1)	92.4 (18.4)
Ocmulgee National Monument	42.1 (24.9)	97 (5.4)	89.5 (7.6)	60.5 (32.6)	96.4 (8.7)	36.6 (22.2)
Olympic National Park	86 (11.1)	1.1 (0.2)	27.3 (17.4)	13 (27.8)	68 (27.6)	96.7 (6.7)
Oregon Caves National Monument	81.7 (19.7)	3.2 (3.8)	27 (33.9)	69.2 (49.9)	27.9 (30.6)	92.8 (12.4)
Organ Pipe Cactus National Monument	98.7 (3.9)	14 (31.7)	68 (36.5)	26.4 (22.4)	51.8 (17.5)	94.6 (1.2)
Ozark National Scenic Riverways	94.1 (13.5)	29.1 (28.8)	39.8 (19.3)	31.1 (31.2)	79.4 (16.1)	85.9 (21)
Padre Island National Seashore	100 (0)	100 (0)	99.2 (2.4)	25.8 (22.2)	98.4 (4.9)	77.5 (36.8)
Palo Alto Battlefield National Historical Park	100 (0)	85 (5)	96.8 (9.6)	20.5 (15.8)	82.7 (11.5)	87.4 (34.9)
Pea Ridge National Military Park	99.7 (1)	8.8 (13.9)	24.1 (7.7)	21.3 (35.5)	73.2 (17.7)	96.7 (6.8)
Pecos National Historical Park	100 (0)	6.4 (3.5)	45.7 (25.1)	11.8 (13.9)	96.2 (6.3)	96.7 (8.7)
Petersburg National Battlefield	100 (0)	3.5 (2.4)	6.3 (4.6)	35.3 (56.4)	99.3 (2.2)	92.1 (20.4)
Petrified Forest National Park	99 (2.9)	10.7 (4)	56.9 (47.9)	30.2 (36.5)	100 (0)	93 (11.5)
Petroglyph National Monument	100 (0)	3 (5.7)	26 (16)	13.8 (13.9)	41.8 (24)	97.1 (8.7)
Pictured Rocks National Lakeshore	97.6 (3.9)	1.1 (0.2)	7 (15.8)	38.1 (47.9)	41 (20.4)	71.2 (36.5)
Pinnacles National Monument	95.7 (5.1)	8 (9.3)	53.6 (14.4)	33.6 (16.1)	23.8 (27.3)	98.1 (3.6)
Pipe Spring National Monument	99 (2.9)	10.6 (10.3)	63.3 (55.4)	24.5 (28.7)	99.7 (1)	96.5 (6.8)
Pipestone National Monument	95.4 (9.5)	7.1 (12.2)	56.3 (24.2)	8.6 (10.2)	21.2 (29.4)	98 (3.9)
Piscataway Park	97.8 (6.5)	1.4 (1.2)	18.8 (24.5)	39.6 (45)	79.8 (20.3)	85.5 (15.8)
Point Reyes National Seashore	86.4 (21.1)	2.4 (3.8)	20.3 (20.2)	58.8 (12.6)	62.4 (16.2)	96.3 (7.7)
Port Chicago Naval Magazine National Memorial	83.1 (19.7)	3.8 (5.7)	37.2 (25.4)	42.8 (18.3)	3.8 (6.7)	94.6 (11.5)
Presidio of San Francisco	91.2 (14.3)	1.7 (1.8)	12.7 (3.8)	53.9 (11.7)	56.1 (8.6)	97.7 (5.8)
Prince William Forest Park	81.2 (32.6)	1.1 (0.2)	11.1 (19.7)	36.7 (46.9)	69.2 (28.2)	83.1 (17.5)
Pu'uhonua o Honaunau National Historical Park	77.8 (3.2)	18 (33)	15.1 (27.5)	80.1 (34.8)	70.5 (15.9)	92.2 (16.1)
Puukohola Heiau National Historic Site	79.4 (5.2)	1.1 (0.2)	9.8 (16.8)	79.7 (23.4)	70 (14)	96.6 (6.5)
Rainbow Bridge National Monument	97.4 (7.8)	97 (5.7)	86.6 (31)	32.7 (52)	99.4 (1.9)	91.7 (16.5)
Redwood National Park	43.3 (57.5)	77.2 (24)	61.8 (53)	95.7 (10.8)	69.6 (23.2)	54.8 (58.9)
Richmond National Battlefield Park	100 (0)	2.4 (2.7)	2.6 (1.7)	44.3 (42.7)	97.9 (5.4)	93.1 (17.5)
Rio Grande Wild and Scenic River	99.7 (1)	95.7 (10.7)	91 (10.2)	46.3 (23)	98.4 (4.9)	93.5 (11.4)
Rock Creek Park	97.8 (6.5)	6.8 (9.9)	24 (28.6)	38.2 (44.3)	80 (21.7)	82 (17.3)
Rocky Mountain National Park	98.4 (4.9)	81.8 (28.6)	75.4 (24.6)	32.6 (37.1)	98.4 (4.9)	88.7 (21.2)

Park	Bio1	Bio2	Bio3	Bio4	Bio5	Bio6
Roosevelt-Vanderbilt Headquarters	100 (0)	1.1 (0.2)	28.4 (31.5)	38 (30)	98.4 (4.9)	95.1 (13.6)
Russell Cave National Monument	80.9 (32.3)	6 (12.4)	29.9 (34.1)	34.3 (42.3)	58.7 (25.3)	70.2 (22.3)
Sagamore Hill National Historic Site	100 (0)	15.4 (24.3)	30.5 (30.2)	41.6 (59.8)	100 (0)	90.8 (23.3)
Saguaro National Park	100 (0)	4.5 (4.3)	2.8 (1.7)	90.1 (5.1)	92.7 (15.1)	92 (3.9)
Saint Croix National Scenic Riverway	99.7 (1)	2.4 (2)	13.6 (28.4)	9.8 (16.3)	65.7 (28.6)	97.3 (5.8)
Saint-Gaudens National Historic Site	100 (0)	8.8 (19.4)	61.4 (53.2)	13.5 (32.8)	97.7 (6.8)	97.1 (8.7)
Salinas Pueblo Missions National Monument	100 (0)	3.8 (6.7)	30.4 (36.8)	8.7 (10)	53.5 (44.5)	97 (7.8)
San Antonio Missions National Historical Park	100 (0)	56.7 (27)	63.8 (40.6)	30.1 (18.6)	99.7 (1)	98.8 (3.6)
San Juan Island National Historical Park	86.8 (12.4)	12.1 (2.8)	49.4 (34)	17.3 (15.6)	98.6 (3.2)	95.7 (9.6)
Sand Creek Massacre National Historic Site	100 (0)	59.6 (37.4)	86.4 (10.7)	17 (25)	88.5 (10.3)	92.6 (13.1)
Santa Monica Mountains National Recreation Area	34.4 (41.7)	1.1 (0.2)	34.2 (34.1)	1.7 (1.8)	3.2 (3.8)	99.2 (2.4)
Saratoga National Historical Park	100 (0)	1.1 (0.2)	20.2 (33.5)	15 (32.8)	56.3 (15.9)	98.4 (4.9)
Saugus Iron Works National Historic Site	100 (0)	1.1 (0.2)	21 (35.1)	22.1 (44.4)	99.7 (1)	93.5 (19.4)
Scotts Bluff National Monument	99.4 (1.9)	67.5 (31.9)	75.3 (21.5)	24 (31.6)	98.7 (3.9)	97.4 (7.8)
Sequoia and Kings Canyon National Parks	97.9 (4.3)	1.5 (1.4)	10.5 (15.2)	27.1 (35.1)	40 (53.1)	96.5 (4.7)
Shenandoah National Park	79.2 (29.4)	4.4 (6.6)	8.4 (15.2)	20.8 (39.1)	38 (12.1)	84.5 (18.8)
Shiloh National Military Park	78.5 (28.9)	5.1 (7.3)	10.4 (3.4)	48.6 (23.1)	60.9 (16.1)	88 (15.9)
Sitka National Historical Park	81.3 (27.7)	4.5 (3.4)	55.5 (28.9)	19.3 (41.5)	53.8 (19.9)	95 (10.7)
Sleeping Bear Dunes National Lakeshore	97.3 (6)	5.1 (8.5)	25.3 (20.1)	14.8 (26)	28.9 (33.9)	83.7 (24.3)
Stones River National Battlefield	70.1 (32.9)	5.9 (6.1)	18.5 (15.8)	33.8 (37.7)	49.3 (19.6)	54.3 (9.6)
Sunset Crater Volcano National Monument	99 (2.9)	57.4 (19)	87.4 (23)	29.8 (27.6)	96.5 (9.6)	93.1 (13.6)
Tallgrass Prairie National Preserve	99.7 (1)	36.3 (27.5)	86.6 (15.6)	10.5 (16)	65.7 (17.2)	97 (5.8)
Theodore Roosevelt National Park	84.9 (20.2)	72.8 (40.9)	94.5 (10.8)	13.2 (22.1)	81.5 (29.7)	99.7 (1)
Thomas Stone National Historic Site	96.6 (6.5)	1.4 (1.2)	16.7 (26.2)	40.7 (44.1)	83.3 (18.3)	86.7 (19.8)
Timpanogos Cave National Monument	97.1 (8.7)	2.9 (2.8)	3.5 (1.9)	83.9 (34.3)	97.7 (4.9)	96 (9.7)
Timucuan Ecological and Historic Preserve	90.9 (17.5)	99.6 (1.1)	73.8 (18.8)	69.7 (16.7)	89.4 (17.2)	49.1 (59.2)
Tonto National Monument	99.4 (1.9)	8.3 (10.3)	39.9 (31.9)	38.8 (28.3)	99.6 (1.2)	96.5 (4.7)
Tumacacori National Historical Park	100 (0)	1.4 (0.9)	13.2 (19)	67.4 (21.1)	92.4 (15.1)	100 (0)
Tuzigoot National Monument	99.4 (1.9)	16.8 (5.4)	49.6 (32.5)	33.7 (39.7)	99.7 (1)	96.9 (6.8)
Upper Delaware National Scenic and Recreational River	100 (0)	2.8 (4.2)	39.6 (34.3)	20.8 (39.6)	94.8 (10.7)	87.9 (22.1)
Valley Forge National Historical Park	100 (0)	11.9 (6.9)	40.7 (36.2)	19 (40.5)	99.3 (1.1)	91.8 (20.4)
Valor in the Pacific National Monument	96.2 (7.5)	1.1 (0.2)	8.6 (11.2)	83.9 (19.8)	85.5 (25.3)	97 (7.8)
Vicksburg National Military Park	74.2 (30.3)	6.3 (13.4)	12.3 (15.1)	56.4 (27)	58.1 (38.4)	86.7 (25.7)
Virgin Islands National Park	89.2 (17.5)	51.5 (23.1)	47.6 (12)	93.8 (10.8)	89.5 (16.5)	91.3 (17.2)
Voyageurs National Park	99.4 (1.9)	36 (24.2)	77.6 (11.1)	3.7 (6.6)	52.3 (29.7)	93 (15.5)
Walnut Canyon National Monument	99 (2.9)	54.5 (19.5)	79.8 (26.3)	31.7 (34.6)	94.5 (15.7)	93.4 (12.6)
War In The Pacific National Historical Park	94.5 (8.6)	85.1 (16.7)	58.2 (4.9)	35.8 (28.5)	83.8 (22.3)	69.9 (44.9)
Washita Battlefield National Historic Site	76.7 (39.5)	69.8 (51.7)	94.8 (10.8)	10.9 (14.8)	48.9 (36.1)	89.5 (21.7)
Weir Farm National Historic Site	100 (0)	49.4 (21.1)	45.5 (19.5)	23.8 (45.4)	100 (0)	91.8 (22.3)
Whiskeytown National Recreation Area	66.8 (36.6)	2.9 (2.8)	29.5 (33.1)	45.8 (58.5)	14.7 (31)	93.1 (16.3)

Park	Bio1	Bio2	Bio3	Bio4	Bio5	Bio6
White Sands National Monument	100 (0)	10.9 (26.6)	28.2 (27.4)	27.2 (16.3)	54.9 (39.5)	99.7 (1)
Whitman Mission National Historic Site	84.5 (28.4)	22 (28.9)	72.6 (38.5)	21.5 (19.6)	98.3 (2.9)	94.1 (16.5)
Wilson's Creek National Battlefield	95.2 (4.3)	17.2 (19)	29.7 (8.7)	26.4 (42.1)	69.2 (19.1)	87.8 (20.7)
Wind Cave National Park	79.6 (26)	98.7 (2.9)	75.9 (17.9)	59.2 (29.7)	90.4 (4.1)	82.2 (19)
Wolf Trap National Park for the Performing Arts	79.5 (41.2)	1.4 (1.2)	18.2 (27.7)	32.8 (44.5)	64.1 (18.2)	78.8 (17.3)
Wrangell - St Elias National Park and Preserve	86.2 (15.8)	6 (2.9)	59.9 (30.8)	26.2 (37.9)	40.3 (41.1)	94.6 (9.7)
Wright Brothers National Memorial	98.6 (2.2)	3.7 (7.7)	8.1 (6.7)	68.6 (37)	93.4 (12.9)	85.5 (35)
Wupatki National Monument	99 (2.9)	59.6 (17.6)	81.8 (21.2)	32.8 (35.3)	99.3 (1.2)	92.7 (12.5)
Yellowstone National Park	97.5 (3.1)	33.6 (13.8)	87.6 (32.8)	12.2 (13.9)	97.6 (2.7)	96.7 (8.7)
Yosemite National Park	99.4 (1.9)	1.5 (1.4)	1.5 (1.4)	97.3 (3.6)	99.7 (1)	98.6 (2.9)
Yucca House National Monument	97.4 (7.8)	100 (0)	89.8 (24.3)	39.9 (50.7)	99.7 (1)	85.8 (28.8)
Yukon-Charley Rivers National Preserve	94.4 (7.6)	14.8 (10.9)	65.5 (17.2)	24.1 (37.9)	35.9 (42.8)	92.6 (14.3)
Zion National Park	99 (2.9)	15.6 (16.6)	57.1 (51.9)	37.6 (42.5)	99.7 (1)	97 (7.8)
