# Supplementary material for: Climate Exposure of US National Parks in a New Era of Change
Source: PLoS One. 2014 Jul 2;9(7):e101302. doi: 10.1371/journal.pone.0101302 (PMC4079655; doi:10.1371/journal.pone.0101302)
Supplement: Appendix S2 — Mean percentile and maximum difference in percentile (in parentheses) for moving window means: temperature annual range (Bio7), mean temperature of the wettest quarter (Bio8), mean temperature of the driest quarter (Bio9), mean temperature of the warmest quarter (Bio10), and mean temperature of the coldest quarter (Bio11). (PDF) [file pone.0101302.s002.pdf]

Appendix S2. Mean percentile and maximum difference in percentile (in parentheses) for moving window means: temperature annual range (Bio7), mean temperature of the wettest quarter (Bio8), mean temperature of the driest quarter (Bio9), mean temperature of the warmest quarter (Bio10), and mean temperature of the coldest quarter (Bio11).

| Park | Bio7 | Bio8 | Bio9 | Bio10 | Bio11 |
|---|---|---|---|---|---|
| Abraham Lincoln Birthplace National Historical Park | 16.6 (35.7) | 98.1 (3.6) | 82 (15.9) | 97.7 (4.8) | 83.8 (28.9) |
| Acadia National Park | 20.5 (36.7) | 98.6 (4.3) | 35 (69.1) | 100 (0) | 96.8 (9.7) |
| Agate Fossil Beds National Monument | 40.3 (44.6) | 76.3 (32) | 17.4 (14.4) | 99 (2.9) | 89.9 (18.9) |
| Ala Kahakai National Historic Trail | 41.3 (71.3) | 60.7 (43.1) | 96.9 (8.4) | 76.9 (4.7) | 75.7 (14.7) |
| Alagnak Wild River | 57.6 (32.9) | 68.8 (34.5) | 55.7 (33.5) | 86.6 (16.1) | 76 (30.9) |
| Alibates Flint Quarries National Monument | 10.3 (13.9) | 24.7 (43.1) | 86 (29.1) | 72.2 (36.5) | 85.6 (25.4) |
| Allegheny Portage Railroad National Historic Site | 10.7 (21.1) | 46.5 (52.6) | 39.2 (34.8) | 62.4 (8.9) | 78.9 (30.2) |
| American Memorial Park | 60.7 (10.2) | 95.2 (9.7) | 92.6 (14.6) | 89.3 (21.5) | 92.9 (11.7) |
| Amistad National Recreation Area | 66.7 (40.8) | 47.5 (18.3) | 78 (40) | 96.8 (9.7) | 94.8 (13.6) |
| Anacostia Park | 29.4 (12.2) | 31.3 (37.3) | 39.6 (19.4) | 99 (1.9) | 76.6 (27) |
| Aniakchak National Monument and Preserve | 92.3 (8.8) | 40 (22.4) | 34 (17.4) | 88.5 (9) | 71.4 (32.8) |
| Antietam National Battlefield | 14.8 (26) | 30 (33.9) | 71.6 (9.4) | 65.8 (29) | 66.4 (31.2) |
| Apostle Islands National Lakeshore | 15.3 (32.8) | 61.2 (28.6) | 94.5 (16.5) | 100 (0) | 90.6 (18.4) |
| Appalachian National Scenic Trail | 11.4 (23.1) | 81.4 (29.7) | 49.3 (66.8) | 100 (0) | 91.8 (21.4) |
| Appomattox Court House National Historical Park | 28.2 (28.4) | 36.6 (48.6) | 28.5 (65.5) | 99.7 (1) | 83.7 (6.4) |
| Arches National Park | 64.4 (47.5) | 57.4 (52.3) | 89.3 (10.2) | 97.3 (4.3) | 85.7 (31) |
| Arkansas Post National Memorial | 11.9 (8.9) | 61.8 (25.8) | 73 (25.6) | 86.7 (20.4) | 48.8 (10.5) |
| Assateague Island National Seashore | 58.9 (28) | 40.5 (68.8) | 78.6 (33.5) | 100 (0) | 85.4 (32.8) |
| Aztec Ruins National Monument | 36.5 (52.8) | 61 (73.1) | 97.4 (3.8) | 100 (0) | 93.1 (13.6) |
| Badlands National Park | 28.7 (40.4) | 48.2 (33.5) | 28.3 (15.8) | 91.9 (6.3) | 71.8 (42.8) |
| Baltimore-Washington National Parkway | 41.6 (10.9) | 32.9 (40.9) | 21.7 (19.8) | 99 (2.9) | 79.3 (32.8) |
| Bandelier National Monument | 7.3 (11.4) | 21.6 (11.2) | 97 (5.7) | 99.6 (1.2) | 94.4 (10.7) |
| Bent's Old Fort National Historic Site | 37.6 (49.7) | 37.8 (17.7) | 82.4 (28.7) | 98.8 (3.6) | 91 (18.9) |
| Bering Land Bridge National Preserve | 32.5 (20.3) | 98.5 (3.6) | 74.2 (22.6) | 94.4 (7.5) | 88.1 (14) |
| Big Bend National Park | 60.1 (22.2) | 80.5 (25.1) | 99.6 (1.1) | 99.4 (1.9) | 97.4 (7.8) |
| Big Cypress National Preserve | 32.8 (44) | 100 (0) | 79.1 (48.1) | 99.3 (1.2) | 84.9 (29.9) |
| Big Hole National Battlefield | 4.7 (9.6) | 22.4 (34.4) | 44.8 (19.9) | 99.3 (1.1) | 99 (2.9) |
| Big South Fork National River and Recreation Area | 12 (24) | 95.1 (4.4) | 92.4 (18.3) | 69 (24.9) | 58.6 (25) |
| Big Thicket National Preserve | 75.9 (7.7) | 98.9 (0.2) | 75.3 (9.8) | 97.6 (7.2) | 47.2 (8.2) |
| Bighorn Canyon National Recreation Area | 14.9 (20.8) | 8.4 (14.3) | 67.5 (25) | 98.4 (4.9) | 93.5 (8.3) |
| Biscayne National Park | 48.9 (45.1) | 100 (0) | 75.9 (58) | 98.9 (0.2) | 88.6 (22.1) |
| Black Canyon Of The Gunnison National Park | 77.5 (37.6) | 44.3 (26) | 84.3 (28.1) | 100 (0) | 79 (42.3) |
| Blue Ridge Parkway | 13.7 (28.9) | 55.6 (47.6) | 36.5 (49.6) | 87.4 (12) | 60 (14.3) |
| Bluestone National Scenic River | 42.9 (23.1) | 82.3 (24.5) | 45.8 (17.1) | 99 (1.9) | 66.2 (26.8) |
| Booker T. Washington National Monument | 8.1 (14.3) | 39 (34.3) | 59.4 (46.3) | 95.2 (3.1) | 89.9 (7.3) |
| Boston Harbor Islands National Recreation Area | 34.8 (48.3) | 95.4 (9.5) | 39.5 (57.7) | 99.7 (1) | 94.8 (14.6) |

| Park | Bio7 | Bio8 | Bio9 | Bio10 | Bio11 |
|---|---|---|---|---|---|
| Bryce Canyon National Park | 12.9 (18) | 62.4 (60.7) | 99 (1.9) | 99.7 (1) | 95.6 (10.7) |
| Buck Island Reef National Monument | 70.9 (47.1) | 94.6 (9.7) | 77.3 (36.9) | 89.2 (19.4) | 86.9 (24.3) |
| Buffalo National River | 12.1 (21.9) | 12.9 (20.8) | 74.3 (43.5) | 93 (7.6) | 75.3 (29.9) |
| Cabrillo National Monument | 8.5 (14.2) | 69.7 (14.7) | 80.1 (6.1) | 62.1 (10.4) | 76.6 (8.1) |
| Canaveral National Seashore | 80.8 (9.1) | 57.7 (30.1) | 82.8 (39.1) | 87.4 (11.2) | 54.4 (18.5) |
| Canyon De Chelly National Monument | 28.1 (46.4) | 19.7 (9.3) | 93.8 (7.8) | 100 (0) | 93.5 (13.6) |
| Canyonlands National Park | 61.5 (57.8) | 55.1 (52.5) | 77.1 (27.3) | 99.7 (1) | 87.1 (28.1) |
| Cape Cod National Seashore | 74.6 (17.7) | 97.1 (8.6) | 31 (49.4) | 100 (0) | 96.4 (10.7) |
| Cape Hatteras National Seashore | 58.2 (18.3) | 54.1 (19.7) | 72.2 (25.1) | 100 (0) | 81.3 (37.4) |
| Cape Krusenstern National Monument | 30.6 (18.1) | 81 (38.8) | 69.9 (20.8) | 94.7 (7.5) | 88.8 (14.1) |
| Cape Lookout National Seashore | 41.6 (28.2) | 47.3 (17.6) | 77.8 (21) | 100 (0) | 82.4 (38.8) |
| Capitol Hill Parks | 28.7 (11.2) | 30.2 (36.5) | 41.6 (18.5) | 99 (1.9) | 75.9 (26.8) |
| Capitol Reef National Park | 26.4 (53.8) | 76.8 (31.7) | 85.5 (25) | 99.7 (1) | 95.2 (9.7) |
| Capulin Volcano National Monument | 35.8 (54.7) | 99.3 (1.2) | 61.1 (26.3) | 100 (0) | 94.3 (12.5) |
| Carl Sandburg Home National Historic Site | 22.5 (32.3) | 28.7 (40.4) | 80.2 (46.5) | 74.9 (19.2) | 47.6 (17.8) |
| Carlsbad Caverns National Park | 6.7 (3.4) | 100 (0) | 93.4 (17.5) | 100 (0) | 98.9 (2.4) |
| Casa Grande Ruins National Monument | 7.9 (7.6) | 73.9 (15.9) | 99 (1.9) | 99.7 (1) | 97.2 (7.2) |
| Castillo De San Marcos National Monument | 80 (27.9) | 96.5 (9.6) | 51.7 (42.2) | 89.6 (14.8) | 54.6 (33.2) |
| Catoctin Mountain Park | 21.3 (25.8) | 36.1 (31.5) | 77.2 (8.4) | 76.6 (24.4) | 64.3 (34.1) |
| Cedar Breaks National Monument | 10.9 (22.2) | 30.4 (39.4) | 87.2 (19) | 99 (2.9) | 95.6 (10.7) |
| Chaco Culture National Historical Park | 8.9 (16.3) | 72.1 (4.3) | 96.5 (2.7) | 100 (0) | 94.1 (11.7) |
| Channel Islands National Park | 1.1 (0.2) | 84.9 (8.3) | 76.1 (11.9) | 62.4 (9.3) | 85.6 (7.8) |
| Charles Pinckney National Historic Site | 63.2 (36.5) | 50.2 (69.1) | 60.7 (34.9) | 99.3 (1.2) | 56.5 (15.3) |
| Chattahoochee River National Recreation Area | 37.2 (21.8) | 83.1 (24.5) | 98.2 (1.3) | 100 (0) | 84.1 (35.9) |
| Chesapeake and Ohio Canal National Historical Park | 11.7 (24) | 31.6 (38) | 78.1 (29.8) | 71.1 (20.1) | 70.2 (28.3) |
| Chickamauga and Chattanooga National Military Park | 38.6 (32.6) | 96.8 (8.6) | 99.7 (1) | 98.5 (3.6) | 62.2 (7.3) |
| Chickasaw National Recreation Area | 23.3 (23.2) | 65.3 (45.9) | 59.2 (42.4) | 96.8 (9.6) | 82.9 (32.7) |
| Chiricahua National Monument | 35.7 (15.7) | 72.2 (54.7) | 87.3 (17) | 100 (0) | 99.2 (2.4) |
| City Of Rocks National Reserve | 21.1 (26.9) | 45.3 (42.7) | 86.2 (28.1) | 97.6 (3.9) | 77.9 (38.8) |
| Colonial National Historical Park | 27.9 (51.7) | 40.8 (62.3) | 63 (46.5) | 100 (0) | 87.5 (24) |
| Colorado National Monument | 83.2 (26) | 50.4 (47.3) | 94.5 (6.3) | 99.7 (1) | 77.6 (53.2) |
| Congaree National Park | 48.4 (28.7) | 59.1 (13.2) | 58.6 (15.8) | 78.1 (21.9) | 49.4 (9.2) |
| Coronado National Memorial | 29.3 (10.3) | 64.1 (26) | 96.5 (3.7) | 100 (0) | 99.6 (1.2) |
| Cowpens National Battlefield | 21.9 (39.8) | 72.9 (43.7) | 79.5 (40.2) | 71 (20.3) | 70.9 (29) |
| Crater Lake National Park | 5.8 (11.5) | 99 (1.9) | 99.6 (1.1) | 98.9 (3.2) | 96.2 (6.7) |
| Craters Of The Moon National Monument and Preserve | 19.2 (28) | 80.8 (21.8) | 81.1 (47.2) | 77.5 (17.7) | 86 (31.3) |
| Cumberland Gap National Historical Park | 10.4 (21.1) | 96 (9.7) | 92.8 (10.8) | 80.5 (14.6) | 44.3 (15.1) |
| Cumberland Island National Seashore | 86 (9.3) | 48.4 (38.8) | 72.6 (14.7) | 95.8 (5.2) | 59 (45.3) |
| Curecanti National Recreation Area | 64.4 (54.2) | 48.4 (29.6) | 72 (46.6) | 100 (0) | 78.7 (38.1) |
| Cuyahoga Valley National Park | 25.9 (48.3) | 61.8 (43.1) | 80.7 (27.2) | 90.8 (12.2) | 81.3 (37.2) |

| Park | Bio7 | Bio8 | Bio9 | Bio10 | Bio11 |
|---|---|---|---|---|---|
| Death Valley National Park | 6.7 (9.6) | 74.1 (12) | 92.9 (19.3) | 99 (2.9) | 99.2 (2.4) |
| Delaware Water Gap National Recreation Area | 21.6 (38.6) | 47.1 (27.7) | 62.3 (36) | 100 (0) | 91.1 (21.4) |
| Denali National Park and Preserve | 19 (12.2) | 86.2 (16.3) | 70.1 (45.4) | 93.6 (10.7) | 82.1 (21.4) |
| Devils Postpile National Monument | 13.1 (25.1) | 82 (18) | 99.4 (1.9) | 100 (0) | 98.7 (3.9) |
| Devils Tower National Monument | 39.9 (26.7) | 43.3 (9.1) | 12.5 (15.7) | 93.2 (11.7) | 73.5 (20.7) |
| Dinosaur National Monument | 39 (45.4) | 36.9 (70.8) | 51.7 (69.2) | 96.4 (10.7) | 78.5 (40.7) |
| Dry Tortugas National Park | 26.3 (55.8) | 79.2 (51.3) | 33.3 (60) | 97 (3.3) | 70.2 (57.6) |
| Ebey's Landing National Historical Reserve | 9.1 (12.3) | 93 (14.6) | 96.3 (7.8) | 89.8 (11.3) | 92.6 (4.3) |
| Effigy Mounds National Monument | 8.4 (14.3) | 86.1 (11) | 63.2 (28.5) | 93.9 (2.7) | 91.1 (21.4) |
| Eisenhower National Historic Site | 32.3 (8) | 40.3 (36.1) | 74.5 (20.8) | 81.2 (21.5) | 63.2 (32.4) |
| El Malpais National Monument | 6.9 (10.5) | 49.3 (24.6) | 96.3 (7.7) | 100 (0) | 96.3 (8.7) |
| El Morro National Monument | 7.7 (9.4) | 62.4 (10.4) | 98.3 (3.9) | 100 (0) | 95.6 (10.7) |
| Eugene O'Neill National Historic Site | 6.3 (15.4) | 73.2 (12.3) | 94.7 (6.2) | 81.3 (9.3) | 91.9 (12.5) |
| Everglades National Park | 41.4 (45.8) | 99.3 (1.1) | 75.5 (56.1) | 99.3 (1.2) | 82.6 (35.7) |
| Fire Island National Seashore | 70.5 (32.9) | 92.3 (19.4) | 13 (10.8) | 100 (0) | 94.4 (14.6) |
| Florissant Fossil Beds National Monument | 60.9 (33.1) | 88.9 (20.4) | 60.4 (63.8) | 97.1 (4.8) | 94.8 (9.7) |
| Fort Bowie National Historic Site | 35.8 (18.6) | 68.2 (56.9) | 87.5 (22.1) | 100 (0) | 99.2 (2.4) |
| Fort Caroline National Memorial | 83.6 (17.5) | 81.1 (46.5) | 74.4 (15.2) | 91.6 (14.9) | 54.3 (34.4) |
| Fort Davis National Historic Site | 41.9 (31.3) | 93.8 (10.8) | 99 (2.9) | 100 (0) | 97.1 (8.7) |
| Fort Donelson National Battlefield | 20.2 (46.1) | 100 (0) | 58.4 (19.2) | 64.7 (35.1) | 53.4 (4.1) |
| Fort Frederica National Monument | 86 (9.3) | 33.2 (21.3) | 74.8 (26) | 98.2 (0.9) | 61 (41.6) |
| Fort Laramie National Historic Site | 41.5 (41.7) | 77.8 (17.1) | 15.8 (16.7) | 98.1 (5.8) | 92.7 (15.3) |
| Fort Larned National Historic Site | 16.2 (28.4) | 51 (48.1) | 82.7 (26.5) | 70.3 (28.1) | 88.6 (13) |
| Fort Matanzas National Monument | 76.9 (33.9) | 89.1 (24.6) | 48.6 (47.3) | 87.4 (14.5) | 47.5 (26.5) |
| Fort Moultrie National Monument | 65.3 (37.5) | 48.5 (67.2) | 64.6 (32.4) | 99.3 (1.2) | 57.4 (11.4) |
| Fort Necessity National Battlefield | 10.6 (23.1) | 42.7 (39) | 57.7 (51.2) | 76.3 (21.3) | 76.2 (26.3) |
| Fort Point National Historic Site | 7.6 (13.5) | 93.2 (13.5) | 94.5 (8.6) | 87.2 (12.4) | 91.1 (17.4) |
| Fort Pulaski National Monument | 75.4 (26.7) | 27.5 (42.8) | 69.2 (39.4) | 98.6 (1.2) | 55.9 (19.7) |
| Fort Raleigh National Historic Site | 55.2 (21.6) | 65.7 (16.9) | 54.6 (43.2) | 100 (0) | 83.8 (36.9) |
| Fort Sumter National Monument | 64.6 (37.5) | 50.2 (69.1) | 61.4 (34.2) | 99.3 (1.2) | 57.8 (11.4) |
| Fort Union National Monument | 7.2 (10.4) | 92.4 (21.7) | 86.7 (27.9) | 100 (0) | 95.6 (10.7) |
| Fort Union Trading Post National Historic Site | 5.1 (6.6) | 55.4 (33.2) | 38.2 (14.2) | 75 (42.6) | 82.8 (30.1) |
| Fort Vancouver National Historic Site | 5.8 (11.5) | 84.8 (35.7) | 97.3 (4.9) | 94.2 (8.7) | 87.1 (29.9) |
| Fort Washington Park | 18.7 (27.5) | 29.7 (40.1) | 46.5 (19.9) | 99.3 (1.1) | 75.1 (25.6) |
| Fossil Butte National Monument | 16.9 (30.9) | 73 (9.8) | 56.7 (67.1) | 99 (2.9) | 91.1 (18.1) |
| Frederick Douglass National Historic Site | 25.6 (16.6) | 31.3 (36.3) | 44.1 (19.8) | 99.4 (1.9) | 76.2 (26.8) |
| Fredericksburg and Spotsylvania National Military Park | 19.3 (42.5) | 28 (38.2) | 43 (39.6) | 96.8 (6.6) | 66 (21.2) |
| Friendship Hill National Historic Site | 10.6 (23.1) | 31.1 (52.7) | 62.7 (49.3) | 88.3 (17.5) | 78.3 (25) |
| Gates Of The Arctic National Park and Preserve | 10.4 (15.2) | 99.3 (1.1) | 97 (5.7) | 98.9 (3.2) | 90 (17.3) |
| Gateway National Recreation Area | 52.1 (56.7) | 84.1 (25.2) | 41.6 (38.3) | 100 (0) | 91.7 (19.4) |

| Park | Bio7 | Bio8 | Bio9 | Bio10 | Bio11 |
|---|---|---|---|---|---|
| Gauley River National Recreation Area | 32.9 (33.2) | 83.3 (47) | 75 (10.8) | 99.7 (1) | 72.6 (26.3) |
| George Washington Birthplace National Monument | 18.2 (41.3) | 30.1 (49.5) | 55.9 (33) | 99.6 (1.2) | 75.6 (18.3) |
| George Washington Carver National Monument | 31.7 (36) | 32.1 (43) | 62.5 (49.9) | 92.3 (7.7) | 75.5 (22.8) |
| George Washington Memorial Parkway | 27.5 (13) | 29.5 (35.8) | 38.6 (7.2) | 93.8 (12.9) | 71.5 (24.4) |
| Gettysburg National Military Park | 30.3 (14) | 40.2 (39.5) | 75.4 (16.5) | 84.7 (16.3) | 65.5 (36.3) |
| Gila Cliff Dwellings National Monument | 4.5 (5.6) | 50 (13.9) | 75.6 (40.6) | 100 (0) | 99.2 (2.4) |
| Glacier Bay National Park and Preserve | 4.4 (7.5) | 30.1 (34.3) | 26.1 (29.5) | 92 (5.6) | 90.9 (16.3) |
| Glacier National Park | 1.7 (1.8) | 96.9 (4.9) | 6.3 (3.9) | 39.7 (32) | 96.5 (7.5) |
| Glen Canyon National Recreation Area | 43.2 (53.3) | 73.3 (45.4) | 83.6 (20) | 99.7 (1) | 92.1 (16.5) |
| Golden Gate National Recreation Area | 6.1 (12.5) | 89.7 (12.4) | 89.8 (16.5) | 84.8 (16.5) | 90.4 (17.3) |
| Golden Spike National Historic Site | 13.3 (23.2) | 53.8 (49.7) | 97.6 (7.2) | 98.7 (2.9) | 87.3 (22.3) |
| Grand Canyon National Park | 10.5 (16.3) | 91.1 (21.4) | 94.1 (16.5) | 99 (2.9) | 97.3 (6.8) |
| Grand Portage National Monument | 18.7 (39.6) | 87.8 (28.7) | 41.5 (11.4) | 98.7 (3.9) | 86 (28.2) |
| Grand Teton National Park | 11.5 (20) | 84 (23.1) | 95.3 (13.3) | 93.2 (10.8) | 93.9 (9.6) |
| Grant-Kohrs Ranch National Historic Site | 5.9 (14.5) | 52.7 (59.1) | 44.8 (51.4) | 99 (2.9) | 99.4 (1.9) |
| Great Basin National Park | 18 (38.7) | 24.8 (41.6) | 73.2 (28.3) | 98.7 (3.9) | 89.3 (16.5) |
| Great Sand Dunes National Park and Preserve | 37.4 (61.6) | 98.6 (2.2) | 91.7 (0.8) | 100 (0) | 90.1 (22.3) |
| Great Smoky Mountains National Park | 10.4 (20.2) | 97.5 (7.5) | 67 (39) | 75.1 (25.7) | 44 (17.1) |
| Greenbelt Park | 35.2 (13.7) | 32.3 (39) | 31.2 (20.9) | 99.4 (1.9) | 77.7 (29.2) |
| Guadalupe Mountains National Park | 19.4 (12.7) | 100 (0) | 95.1 (14.6) | 100 (0) | 99.2 (2.4) |
| Guilford Courthouse National Military Park | 10.1 (19.2) | 67.9 (35.6) | 40.5 (60.2) | 99.7 (1) | 94.7 (11.7) |
| Gulf Islands National Seashore | 58.2 (16.9) | 90.2 (8.2) | 84.6 (16.2) | 98.9 (2.2) | 45.3 (23.1) |
| Hagerman Fossil Beds National Monument | 16.4 (24.2) | 83.5 (39.8) | 100 (0) | 100 (0) | 96.9 (5.8) |
| Haleakala National Park | 10.6 (14.9) | 79.1 (17.1) | 95.9 (8.4) | 78.7 (9) | 86.2 (7.3) |
| Harpers Ferry National Historical Park | 13.1 (25) | 26.5 (39.6) | 57.1 (7.6) | 58.7 (31.5) | 65.6 (30) |
| Hawaii Volcanoes National Park | 49.2 (72.5) | 59 (46) | 87.7 (24.3) | 77.2 (3.7) | 74.5 (13.7) |
| Herbert Hoover National Historic Site | 7 (13.4) | 75.7 (48.9) | 70.8 (34.8) | 88.4 (16) | 89.7 (23.3) |
| Homestead National Monument of America | 4.1 (5.6) | 6.8 (11.4) | 67.7 (26.1) | 55.5 (25.4) | 83.3 (23.8) |
| Hopewell Culture National Historical Park | 9.8 (17.2) | 16.4 (19.8) | 83.3 (47.2) | 48 (21.6) | 55.9 (10) |
| Hopewell Furnace National Historic Site | 31.1 (22.5) | 49.7 (31.9) | 56.5 (15.4) | 98.3 (3.9) | 89.8 (24.3) |
| Horseshoe Bend National Military Park | 37.8 (24.7) | 82 (22.5) | 98.2 (0.9) | 57.5 (16.2) | 46.4 (5.7) |
| Hot Springs National Park | 7.4 (12.4) | 69.5 (30.3) | 86.5 (32.8) | 89 (27.8) | 71.4 (28.1) |
| Hovenweep National Monument | 80.4 (26.2) | 52.8 (22.8) | 48.3 (42.4) | 100 (0) | 87.6 (30.1) |
| Hubbell Trading Post National Historic Site | 23.7 (27.5) | 36.2 (8.7) | 94.7 (7.3) | 99.4 (1.9) | 93.1 (14.6) |
| Indiana Dunes National Lakeshore | 12.1 (23.1) | 80 (14.8) | 56 (22.3) | 66.9 (10.6) | 69.2 (41.6) |
| Isle Royale National Park | 24.7 (50) | 68 (56.1) | 60.7 (10.2) | 98.4 (4.9) | 85.4 (32) |
| Jean Lafitte National Historical Park and Preserve | 26 (25.7) | 94.7 (4.5) | 90.4 (14.5) | 100 (0) | 58.9 (7.1) |
| Jewel Cave National Monument | 79.3 (2.2) | 3 (5.1) | 14.4 (7.8) | 94.4 (13.6) | 45.2 (20.7) |
| John Day Fossil Beds National Monument | 9.1 (20.3) | 9.5 (7.1) | 89.8 (8) | 85.5 (17.9) | 89.1 (19.1) |
| John Muir National Historic Site | 3.3 (6.7) | 70.1 (17.1) | 94.5 (9.7) | 80.9 (6.7) | 89.3 (15.4) |

| Park | Bio7 | Bio8 | Bio9 | Bio10 | Bio11 |
|---|---|---|---|---|---|
| Johnstown Flood National Memorial | 10 (21.1) | 49.1 (60.8) | 36 (31.3) | 59.8 (1.2) | 79.7 (32.3) |
| Joshua Tree National Park | 4 (8.6) | 68.8 (9.4) | 95.5 (8.6) | 100 (0) | 98.2 (2.9) |
| Kalaupapa National Historical Park | 40.5 (58.8) | 78 (25.3) | 94.6 (8.4) | 80.6 (8.8) | 90.6 (3.1) |
| Kaloko-Honokohau National Historical Park | 39 (67.7) | 61 (51.1) | 96.8 (9.6) | 77.3 (5.7) | 78.9 (19.5) |
| Katmai National Park and Preserve | 58.7 (33.1) | 70.3 (44.9) | 54.4 (37.6) | 85.9 (15.8) | 73.3 (33.8) |
| Kenai Fjords National Park | 13.4 (14.8) | 29.5 (16.5) | 90.6 (16.5) | 60.7 (13.5) | 81.1 (23.8) |
| Kennesaw Mountain National Battlefield Park | 43.3 (30.8) | 78.9 (34.1) | 98.9 (0.2) | 96.9 (7.2) | 77.2 (23.4) |
| Kings Mountain National Military Park | 40.1 (51.8) | 66.8 (17.7) | 79.1 (38.1) | 66.5 (8.3) | 62.4 (30.5) |
| Klondike Gold Rush National Historical Park | 9.8 (15.3) | 35.8 (29.7) | 20.2 (7.6) | 90.6 (6.4) | 88.2 (20.2) |
| Knife River Indian Villages National Historic Site | 3.8 (4.2) | 89.3 (21.9) | 65.3 (7.3) | 95.1 (12.6) | 88.5 (20.4) |
| Kobuk Valley National Park | 17.6 (23.4) | 100 (0) | 77.2 (5.8) | 98.9 (3.2) | 91.3 (14.5) |
| Lake Clark National Park and Preserve | 13.4 (26.8) | 52.8 (24.7) | 72.1 (42.5) | 87.8 (11) | 80.8 (24.3) |
| Lake Mead National Recreation Area | 4.7 (9.6) | 77.7 (21) | 99.4 (1.9) | 99.7 (1) | 98.7 (3.9) |
| Lake Meredith National Recreation Area | 10.9 (15.8) | 25.1 (46.2) | 87 (26.2) | 73.9 (38.5) | 89.1 (18.1) |
| Lake Roosevelt National Recreation Area | 5.4 (10.6) | 57.8 (53) | 100 (0) | 97.2 (7.5) | 94 (12.4) |
| Lassen Volcanic National Park | 14.3 (30) | 69.7 (18.5) | 94.7 (8.7) | 97.5 (0.8) | 97.4 (7.8) |
| Lava Beds National Monument | 9.8 (16.4) | 80.2 (25.2) | 100 (0) | 98.6 (2.2) | 95.8 (5.7) |
| Lewis and Clark National Historical Park | 7.4 (12.2) | 89.1 (29.1) | 92.8 (18.4) | 87.8 (9.8) | 88.3 (14.9) |
| Lincoln Boyhood National Memorial | 8.4 (14.3) | 94.3 (9.6) | 70.6 (19.8) | 89.9 (4.5) | 87.7 (26.7) |
| Little Bighorn Battlefield National Monument | 11.8 (10.2) | 62.5 (14.8) | 43.5 (37.5) | 95.3 (9.7) | 92.1 (10.4) |
| Little River Canyon National Preserve | 17.3 (26.5) | 96.8 (9.7) | 100 (0) | 69.3 (37.8) | 53.1 (1.8) |
| Lyndon B. Johnson National Historical Park | 14.6 (23.3) | 89.8 (5.7) | 60.6 (70) | 58.4 (21.7) | 77.6 (22) |
| Mammoth Cave National Park | 12 (24) | 99.6 (1.2) | 79.4 (24.6) | 84.9 (31.1) | 65.9 (23.2) |
| Manassas National Battlefield Park | 16.9 (27.2) | 30.5 (38.9) | 35.4 (22.1) | 71.8 (26.2) | 63.5 (30.2) |
| Manzanar National Historic Site | 9 (16.4) | 46.5 (43) | 91.3 (13.5) | 98.6 (2.9) | 98.2 (1.3) |
| Marsh-Billings-Rockefeller National Historical Park | 8.4 (16) | 88.2 (31.1) | 47.3 (84.5) | 100 (0) | 97.7 (6.8) |
| Mary McLeod Bethune Council House National Historic Site | 28.7 (11.2) | 29.9 (36.5) | 37.1 (8.8) | 98.3 (3.2) | 74.5 (27.8) |
| Mesa Verde National Park | 53.1 (58.2) | 46.3 (41.2) | 83.4 (16.8) | 100 (0) | 90.1 (22.3) |
| Minute Man National Historical Park | 28 (37.7) | 94.3 (12.8) | 38 (72.8) | 99.7 (1) | 96.4 (10.7) |
| Mississippi National River and Recreation Area | 10.8 (19.2) | 78.3 (35) | 100 (0) | 100 (0) | 93.4 (14.6) |
| Missouri National Recreational River | 7.8 (12.2) | 16.3 (34.2) | 61.1 (25.1) | 71.1 (16.7) | 88.6 (21.4) |
| Mojave National Preserve | 3.7 (7.7) | 81.6 (21.1) | 99.6 (1.1) | 100 (0) | 100 (0) |
| Monocacy National Battlefield | 28.1 (8.3) | 28.5 (35.8) | 54.9 (13.9) | 65.3 (29.9) | 61.2 (38) |
| Montezuma Castle National Monument | 10.5 (19.1) | 53.8 (28.8) | 94.4 (14.6) | 99.4 (1.9) | 97.3 (5.8) |
| Moores Creek National Battlefield | 40.5 (31.3) | 6 (13.4) | 11.5 (15.7) | 99.7 (1) | 54.5 (13.2) |
| Morristown National Historical Park | 32.7 (56.6) | 79.2 (35) | 45.7 (29.7) | 99.7 (1) | 95.7 (10.7) |
| Mount Rainier National Park | 6.8 (13.5) | 81.2 (46.6) | 97.6 (3.9) | 96.5 (8.6) | 92.3 (15.3) |
| Mount Rushmore National Memorial | 74.2 (18.2) | 11.6 (23.6) | 16.5 (10.8) | 94.4 (12.6) | 48.1 (33.3) |
| Muir Woods National Monument | 11.9 (17) | 89.8 (18.1) | 89.2 (18.4) | 85.2 (16.5) | 89.6 (13.1) |
| Natchez Trace Parkway | 27.6 (20.3) | 95.7 (12.9) | 41.4 (35.6) | 77.2 (27.4) | 44.1 (11.5) |

| Park | Bio7 | Bio8 | Bio9 | Bio10 | Bio11 |
|---|---|---|---|---|---|
| National Capital Parks-East | 30.1 (13.1) | 29.5 (35.6) | 37.3 (12.3) | 98.3 (3.2) | 74.5 (27.8) |
| National Park of American Samoa | 57.9 (27.2) | 96.4 (10.7) | 96 (10.7) | 97.1 (8.7) | 96.4 (10.7) |
| Natural Bridges National Monument | 76.4 (30.5) | 31 (15.3) | 46.8 (35.5) | 99.7 (1) | 86.5 (32) |
| Navajo National Monument | 44.6 (55.2) | 51.2 (68.3) | 89.1 (18.1) | 99.4 (1.9) | 92.9 (16.5) |
| New River Gorge National River | 40.9 (25.2) | 81.5 (35.9) | 51.3 (10.8) | 99.3 (1.1) | 69.4 (25.9) |
| Nez Perce National Historical Park | 7.9 (12.3) | 75.2 (19.8) | 84.8 (44.6) | 99.6 (1.1) | 96.7 (8.7) |
| Ninety Six National Historic Site | 52.9 (10.9) | 81.5 (7.4) | 75.5 (7.4) | 98.9 (2.4) | 50.5 (10.4) |
| Niobrara National Scenic River | 7.4 (12.2) | 26.3 (31.3) | 35.5 (10.9) | 78.4 (23.1) | 89.3 (20.4) |
| Noatak National Preserve | 19.9 (16.1) | 100 (0) | 89.2 (17.5) | 98.9 (3.2) | 91.3 (14.5) |
| North Cascades National Park | 6.1 (12.5) | 81.4 (41.5) | 100 (0) | 93.9 (9.7) | 93.2 (11.4) |
| Obed Wild and Scenic River | 9.1 (17.2) | 94 (2) | 88.7 (34) | 82.6 (17) | 52.9 (16) |
| Ocmulgee National Monument | 82.8 (10) | 96.7 (8.6) | 87.1 (19.9) | 71.6 (33.4) | 39.2 (14.2) |
| Olympic National Park | 4.8 (6.6) | 95.7 (9.7) | 92.2 (13.4) | 88.1 (13.5) | 93.8 (6.1) |
| Oregon Caves National Monument | 9.2 (14.5) | 70 (22.7) | 99.6 (1.1) | 98.9 (3.2) | 80.3 (20) |
| Organ Pipe Cactus National Monument | 3.2 (3.8) | 48.3 (61.1) | 84.6 (20.7) | 99 (1.9) | 83.9 (10.7) |
| Ozark National Scenic Riverways | 32.3 (30.3) | 45.1 (32) | 60.3 (53.4) | 92.8 (11.5) | 65.3 (11.6) |
| Padre Island National Seashore | 53.4 (41) | 99.6 (1.2) | 95.4 (8.7) | 97.2 (3.8) | 86.8 (18.5) |
| Palo Alto Battlefield National Historical Park | 42.9 (35.7) | 100 (0) | 90.6 (18.2) | 98.6 (2.9) | 96.1 (11.7) |
| Pea Ridge National Military Park | 12.8 (21.6) | 36 (54.7) | 81.2 (35.9) | 98.4 (4.9) | 85.7 (20.9) |
| Pecos National Historical Park | 5.9 (8.5) | 35.9 (38.7) | 99 (1.9) | 100 (0) | 95.6 (10.7) |
| Petersburg National Battlefield | 42.6 (52.2) | 25.3 (25.3) | 73.2 (42.8) | 99.7 (1) | 92.3 (16.3) |
| Petrified Forest National Park | 16.4 (13.4) | 60.5 (21.7) | 86.5 (23.1) | 99.7 (1) | 93.5 (13.6) |
| Petroglyph National Monument | 6.1 (10.2) | 20.2 (17.3) | 68.5 (39.8) | 96.1 (10.8) | 94.8 (10.7) |
| Pictured Rocks National Lakeshore | 22.7 (37.4) | 5.4 (10.1) | 79.3 (19.2) | 98.7 (3.9) | 83.6 (31.1) |
| Pinnacles National Monument | 3.3 (6.7) | 82.2 (13) | 96.6 (6.5) | 89.6 (12.5) | 96.3 (4.9) |
| Pipe Spring National Monument | 11.1 (18.2) | 81.7 (18.3) | 91.8 (12.9) | 99 (2.9) | 96.3 (8.7) |
| Pipestone National Monument | 8.4 (15.3) | 8.5 (22.2) | 96.8 (9.6) | 49.5 (40.1) | 89.2 (18.4) |
| Piscataway Park | 18.7 (27.5) | 28.7 (40.4) | 44.2 (16.2) | 99.3 (1.1) | 74 (25.3) |
| Point Reyes National Seashore | 13.8 (21.8) | 82.6 (25.8) | 87.1 (21.4) | 84.3 (21.9) | 85 (20.9) |
| Port Chicago Naval Magazine National Memorial | 4 (8.6) | 44.5 (12.5) | 90.9 (15.3) | 64 (15.2) | 78.8 (25.8) |
| Presidio of San Francisco | 7.2 (13.5) | 93.2 (13.5) | 94.5 (8.6) | 87.5 (12.4) | 91.4 (16.4) |
| Prince William Forest Park | 16.9 (28) | 26.4 (33.8) | 32 (10.4) | 85.9 (13.8) | 65.5 (28) |
| Pu'uhonua o Honaunau National Historical Park | 45.1 (77.3) | 57 (59.9) | 100 (0) | 79.8 (5.2) | 75.2 (16.7) |
| Puukohola Heiau National Historic Site | 31.2 (59.3) | 65.3 (30.5) | 97.7 (6) | 77.6 (5.8) | 80 (16.3) |
| Rainbow Bridge National Monument | 38.5 (45.5) | 68.3 (65) | 78.1 (29.6) | 99.7 (1) | 94.5 (11.7) |
| Redwood National Park | 65.5 (49.8) | 32 (31.7) | 79.8 (16.3) | 69.9 (21.5) | 44.1 (50.2) |
| Richmond National Battlefield Park | 28.3 (42.7) | 24.5 (25.3) | 67.5 (43.8) | 99.7 (1) | 90 (16.2) |
| Rio Grande Wild and Scenic River | 58.7 (12.3) | 61.4 (22.2) | 97.4 (7.8) | 98.4 (4.9) | 96.4 (10.7) |
| Rock Creek Park | 31.3 (13.1) | 29.5 (35.6) | 34.5 (12.1) | 97.9 (4.3) | 74.5 (27.8) |
| Rocky Mountain National Park | 51.5 (45.1) | 92.6 (14.1) | 25.3 (44.5) | 98.7 (3.9) | 84.7 (22.9) |

| Park | Bio7 | Bio8 | Bio9 | Bio10 | Bio11 |
|---|---|---|---|---|---|
| Roosevelt-Vanderbilt Headquarters | 18.5 (14.4) | 62.5 (42.8) | 81.9 (30.4) | 100 (0) | 94.4 (14.6) |
| Russell Cave National Monument | 21.1 (40.6) | 96 (10.8) | 90.9 (25.3) | 90.1 (26.5) | 61.8 (4.2) |
| Sagamore Hill National Historic Site | 47.9 (72.4) | 81.2 (39.8) | 31.8 (21.7) | 100 (0) | 94.1 (14.6) |
| Saguaro National Park | 13.2 (0.7) | 88.2 (20.5) | 97 (6.8) | 100 (0) | 97 (5.1) |
| Saint Croix National Scenic Riverway | 10.4 (19.2) | 69.7 (45.2) | 99.7 (1) | 100 (0) | 93.7 (13.6) |
| Saint-Gaudens National Historic Site | 10.7 (14) | 83.7 (38.9) | 47.6 (79.4) | 100 (0) | 97.4 (7.8) |
| Salinas Pueblo Missions National Monument | 4.8 (6.6) | 51 (40.5) | 73.5 (32.2) | 99.6 (1.2) | 97.3 (5.8) |
| San Antonio Missions National Historical Park | 33.5 (33.8) | 81.5 (18.4) | 88.5 (18.7) | 100 (0) | 94 (11) |
| San Juan Island National Historical Park | 9.1 (11.3) | 87.3 (26.2) | 96.4 (9.7) | 91.7 (9) | 92 (6.8) |
| Sand Creek Massacre National Historic Site | 28.8 (39.3) | 43.8 (26.9) | 76 (20.7) | 93.4 (11) | 92.3 (15.1) |
| Santa Monica Mountains National Recreation Area | 1.7 (1.8) | 57 (20.1) | 18.5 (13.5) | 19.4 (19.4) | 61.3 (12.8) |
| Saratoga National Historical Park | 4.4 (6.6) | 85.5 (25) | 77.2 (46) | 100 (0) | 97.1 (7.8) |
| Saugus Iron Works National Historic Site | 29.1 (49) | 99.2 (1.2) | 35.4 (68.5) | 99.7 (1) | 94.5 (15.5) |
| Scotts Bluff National Monument | 28.2 (42.3) | 71.1 (15.5) | 5.8 (7.4) | 99.4 (1.9) | 92.6 (13.1) |
| Sequoia and Kings Canyon National Parks | 9.4 (15.3) | 66.8 (16.9) | 98.5 (1.4) | 99.7 (1) | 98.5 (1.4) |
| Shenandoah National Park | 10.7 (22.1) | 17.1 (32.8) | 21.2 (40.7) | 72.7 (30.3) | 75.1 (25.1) |
| Shiloh National Military Park | 14.7 (20.9) | 99.3 (2.2) | 30.1 (37.7) | 94.2 (11.1) | 51.5 (6.9) |
| Sitka National Historical Park | 6.5 (9.5) | 45.5 (64.6) | 58.6 (13.6) | 87.1 (9.8) | 87.2 (23.1) |
| Sleeping Bear Dunes National Lakeshore | 15 (30.8) | 83.9 (28.1) | 90.1 (18) | 70.3 (39) | 91 (19.4) |
| Stones River National Battlefield | 38.7 (47.5) | 95.8 (9.7) | 53.2 (14.7) | 70.6 (30) | 45.1 (8.6) |
| Sunset Crater Volcano National Monument | 26.9 (39.6) | 65 (36) | 93.2 (20.4) | 99 (2.9) | 94.8 (9.7) |
| Tallgrass Prairie National Preserve | 15.1 (28.4) | 70.1 (23) | 62.7 (15.8) | 77.2 (26.4) | 93 (14.6) |
| Theodore Roosevelt National Park | 4.8 (6.6) | 39.6 (33.6) | 26.6 (7.4) | 82.3 (32.1) | 83.2 (30.1) |
| Thomas Stone National Historic Site | 17.9 (29.7) | 28.4 (48.1) | 44.5 (21) | 99.3 (1.1) | 74 (23.4) |
| Timpanogos Cave National Monument | 15.8 (24) | 74.2 (29.2) | 100 (0) | 99.7 (1) | 91 (18.4) |
| Timucuan Ecological and Historic Preserve | 84.1 (14.2) | 72.9 (56.1) | 74.5 (8.6) | 92.3 (13.8) | 54 (35.4) |
| Tonto National Monument | 11 (13) | 72.6 (14.2) | 83.2 (12.5) | 99.4 (1.9) | 98.1 (2.6) |
| Tumacacori National Historical Park | 3.9 (2.7) | 89.8 (15.7) | 90.8 (14.3) | 100 (0) | 98.8 (2.4) |
| Tuzigoot National Monument | 7.5 (13.4) | 55 (45.5) | 97.4 (7.8) | 99.4 (1.9) | 97.6 (4.9) |
| Upper Delaware National Scenic and Recreational River | 18.3 (29.6) | 54.2 (23.3) | 54.9 (28.1) | 99.7 (1) | 90.8 (22.3) |
| Valley Forge National Historical Park | 20.8 (30.9) | 43.7 (48.4) | 72.6 (33.7) | 100 (0) | 90.8 (22.3) |
| Valor in the Pacific National Monument | 5.4 (11.5) | 81.2 (20.7) | 99 (2.9) | 96.9 (6.5) | 96.5 (4.7) |
| Vicksburg National Military Park | 13.8 (25.7) | 82.1 (26.2) | 66.1 (25.4) | 87.9 (27.9) | 50.7 (5.8) |
| Virgin Islands National Park | 73.1 (40.6) | 95 (10.7) | 79.7 (34) | 89.9 (17.5) | 86.2 (25.2) |
| Voyageurs National Park | 11.5 (19.2) | 80.4 (34) | 90.3 (9.1) | 86 (29.4) | 95.4 (11.7) |
| Walnut Canyon National Monument | 26.5 (39.6) | 55.2 (28.5) | 93.5 (19.4) | 99 (2.9) | 95.4 (7.8) |
| War In The Pacific National Historical Park | 64.5 (10.9) | 96.5 (8.6) | 96.9 (6.5) | 93.5 (11.8) | 94.9 (8.6) |
| Washita Battlefield National Historic Site | 15.2 (25.3) | 21.6 (48.6) | 79.6 (39.8) | 64.3 (47.1) | 74.9 (21.1) |
| Weir Farm National Historic Site | 36.8 (52.7) | 74.5 (38.8) | 19.4 (39.4) | 100 (0) | 95.4 (11.7) |
| Whiskeytown National Recreation Area | 7.4 (16.4) | 46.1 (46.5) | 84.2 (16.9) | 72.6 (39.6) | 89.2 (18.7) |

| Park | Bio7 | Bio8 | Bio9 | Bio10 | Bio11 |
|---|---|---|---|---|---|
| White Sands National Monument | 4.9 (5.6) | 55.3 (43) | 61.7 (30) | 88.8 (33.7) | 96 (12) |
| Whitman Mission National Historic Site | 12.7 (28.9) | 49.6 (32.8) | 99.3 (2.2) | 88.3 (14.8) | 85.2 (14.8) |
| Wilson's Creek National Battlefield | 25.9 (41.7) | 31.1 (8.2) | 77.9 (34.7) | 96.5 (5.8) | 74.6 (22.6) |
| Wind Cave National Park | 68.1 (22.7) | 16.4 (16.3) | 19.2 (19.5) | 95.1 (12.6) | 49.5 (32.3) |
| Wolf Trap National Park for the Performing Arts | 25.3 (13.7) | 28 (28.8) | 35.5 (17.6) | 85.2 (26.5) | 66.4 (31.6) |
| Wrangell - St Elias National Park and Preserve | 6.1 (9.5) | 88.8 (18.7) | 94.7 (15.1) | 92.9 (1.7) | 82.9 (23.6) |
| Wright Brothers National Memorial | 54.8 (21.9) | 64.2 (19.1) | 47.4 (42) | 100 (0) | 84.4 (35.9) |
| Wupatki National Monument | 30 (42.1) | 65 (35.3) | 94.8 (15.5) | 99 (2.9) | 94.1 (11.7) |
| Yellowstone National Park | 11.9 (17.3) | 83 (15.8) | 90.8 (26.5) | 97 (5.8) | 96.9 (5.8) |
| Yosemite National Park | 16.7 (31) | 86 (19.9) | 99.7 (1) | 100 (0) | 99.7 (1) |
| Yucca House National Monument | 61.9 (53.7) | 41.4 (14.9) | 69.9 (32.3) | 100 (0) | 89.8 (23.3) |
| Yukon-Charley Rivers National Preserve | 8.8 (15.1) | 95.7 (10.7) | 93.6 (11.4) | 97.7 (4.9) | 87.7 (17.3) |
| Zion National Park | 10.4 (19.3) | 55.7 (53.5) | 92.3 (4.9) | 99 (2.9) | 96.3 (8.7) |
