# Supplementary material for: Climate Exposure of US National Parks in a New Era of Change
Source: PLoS One. 2014 Jul 2;9(7):e101302. doi: 10.1371/journal.pone.0101302 (PMC4079655; doi:10.1371/journal.pone.0101302)
Supplement: Appendix S3 — Mean percentile and maximum difference in percentile (in parentheses) for moving window means: annual precipitation (Bio12), precipitation of the wettest month (Bio13), precipitation of the driest month (Bio14), precipitation seasonality (Bio15), precipitation of the wettest quarter (Bio16), precipitation of the driest quarter (Bio17), precipitation of the warmest quarter (Bio18), and precipitation of the coldest quarter (Bio19). (PDF) [file pone.0101302.s003.pdf]

Appendix S3. Mean percentile and maximum difference in percentile (in parentheses) for moving window means: annual precipitation (Bio12), precipitation of the wettest month (Bio13), precipitation of the driest month (Bio14), precipitation seasonality (Bio15), precipitation of the wettest quarter (Bio16), precipitation of the driest quarter (Bio17), precipitation of the warmest quarter (Bio18), and precipitation of the coldest quarter (Bio19).

Park	Bio12	Bio13	Bio14	Bio15	Bio16	Bio17	Bio18	Bio19
Abraham Lincoln Birthplace National Historical Park	91.8 (7.9)	88.2 (12.1)	77.8 (37.1)	34.5 (51.5)	89.2 (2.4)	98.5 (2.4)	47.6 (52.4)	70.8 (21.8)
Acadia National Park	98.9 (2.4)	97.1 (8.6)	42.8 (75.8)	80.5 (17.4)	100 (0)	46.8 (45.1)	94.3 (17.2)	28.1 (28.7)
Agate Fossil Beds National Monument	44.2 (20.3)	54.1 (17.7)	30.4 (32.7)	50.9 (16.3)	35.6 (36)	25.4 (20.5)	62.3 (50.5)	6.6 (7.1)
Ala Kahakai National Historic Trail	1.1 (0.2)	3.4 (5.6)	24.2 (24.6)	45.9 (18.7)	2.7 (4.7)	11.2 (13.5)	1.1 (0.2)	33.7 (60)
Alagnak Wild River	66.9 (23.7)	78.4 (42.2)	31.3 (32.6)	77.1 (31.8)	66.3 (20.1)	10 (21.9)	59.5 (20.7)	79.2 (36.3)
Alibates Flint Quarries National Monument	18.5 (19.4)	19 (12.2)	34.3 (53.1)	29.6 (35.3)	5.5 (8.5)	74.9 (40.8)	31.9 (30)	55.8 (76.5)
Allegheny Portage Railroad National Historic Site	99.7 (1)	46.3 (20.8)	98.3 (1.8)	2.8 (1.7)	67.1 (38.3)	99.6 (1.1)	60.4 (46.2)	95.4 (2.8)
American Memorial Park	52.7 (34)	41 (11.1)	87.4 (30.6)	14.4 (9.6)	56 (45.9)	92.2 (7.8)	80.9 (28.2)	77 (20.4)
Amistad National Recreation Area	29.1 (37.9)	33.6 (29.7)	29.5 (43.6)	58.3 (12.5)	36.5 (44.5)	8.8 (6.8)	31.4 (46.4)	29 (43.8)
Anacostia Park	92.1 (2.9)	67.3 (54.3)	64.8 (18.6)	45.9 (40.7)	97.2 (3.8)	69.7 (26.8)	38.3 (32.2)	37.2 (38.7)
Aniakchak National Monument and Preserve	79.1 (21.9)	84.4 (15.4)	68.6 (9)	72.1 (27.9)	73.5 (9)	32.9 (31.4)	67.2 (16.2)	74.9 (29.2)
Antietam National Battlefield	94.2 (7.4)	81.4 (4.6)	77.1 (18)	53.5 (22.1)	89.4 (8.1)	88.4 (12.5)	28.1 (34.8)	74 (25.3)
Apostle Islands National Lakeshore	62.8 (22.8)	14.5 (7.3)	89.3 (13.8)	6.8 (10.2)	35.7 (13.8)	73.1 (23.5)	27.9 (10.9)	60.7 (52.2)
Appalachian National Scenic Trail	98.9 (0.2)	88 (10.4)	97.4 (3.8)	34.5 (51.5)	99.3 (1.2)	97.4 (3.7)	96.6 (6.5)	81.4 (31.9)
Appomattox Court House National Historical Park	76.3 (7.9)	59.6 (33.7)	52.2 (5.4)	43.3 (47.8)	68.7 (28.1)	57.1 (56.3)	34.1 (28.1)	24.3 (32.7)
Arches National Park	50.3 (26.6)	11.3 (17.9)	99.6 (1.1)	2.2 (3.3)	30.8 (15.3)	85.6 (22.5)	31.9 (54.4)	69.5 (31)
Arkansas Post National Memorial	58.2 (34.6)	16.3 (25)	5.8 (1.6)	48.3 (17.7)	13.9 (22.3)	33 (50.6)	26 (26.2)	60.6 (72.1)
Assateague Island National Seashore	92.6 (5.8)	88.9 (3.3)	4.9 (3.2)	85.3 (20.7)	95.7 (10.7)	27.3 (49.3)	37.1 (43.8)	24.9 (16)
Aztec Ruins National Monument	41.6 (51.4)	36 (54.1)	61.5 (62.8)	40.8 (56.5)	48.2 (53.9)	11.1 (15.4)	43.8 (71.8)	64 (35.8)
Badlands National Park	68.8 (48.4)	28.3 (12.5)	93.8 (10.6)	4.8 (5.4)	30.7 (21.2)	95 (9.5)	35.4 (14.9)	73.9 (31.9)
Baltimore-Washington National Parkway	94.9 (2.1)	64.6 (47.7)	71.2 (25.3)	34.5 (37.2)	93 (7.5)	78.9 (24.7)	44.3 (33.7)	52.1 (52.6)
Bandelier National Monument	28.8 (44.5)	2.6 (3.7)	13.5 (26.6)	43.5 (43)	12 (15.6)	16.7 (36.3)	3.9 (8.7)	86.3 (19.9)
Bent's Old Fort National Historic Site	73.5 (41.3)	62.8 (32.2)	71.6 (40.6)	21.4 (21.7)	62.6 (35.8)	69.3 (28.7)	87.3 (15.1)	38.7 (40.4)
Bering Land Bridge National Preserve	88.1 (27.2)	69.6 (32.9)	26.7 (24.4)	25.5 (7.4)	69.3 (33.1)	72.4 (29.5)	65.5 (23.9)	93.8 (7.1)
Big Bend National Park	51.6 (41.8)	56 (30.1)	65.2 (70.4)	77.3 (4.9)	59 (23.5)	47.3 (59.4)	71.3 (23.9)	27.4 (41.1)
Big Cypress National Preserve	96.1 (9.6)	70.4 (45.5)	44.3 (33.8)	51.7 (67.1)	99.6 (1.2)	49.9 (72.3)	99.6 (1.2)	35.1 (60.5)
Big Hole National Battlefield	55.2 (15.8)	37.3 (19)	22.5 (9.3)	49.2 (53.2)	67.4 (29.5)	21.2 (42.9)	30.9 (61.9)	30.7 (44.7)
Big South Fork National River and Recreation Area	59.4 (6.8)	24.2 (34.1)	68.7 (14.5)	33.6 (29)	46.3 (31.6)	68.2 (26.9)	12.4 (23.1)	44.3 (24.6)
Big Thicket National Preserve	71.6 (28)	81.9 (24.8)	44.7 (36.7)	85.6 (21)	74.5 (18.1)	56.3 (40.2)	87.4 (20)	54.9 (42.9)
Bighorn Canyon National Recreation Area	56.5 (15.3)	66.7 (9.2)	48.8 (26.1)	79.9 (22.1)	52.5 (11.6)	22.6 (27.6)	1.8 (2.3)	13 (13.7)
Biscayne National Park	97.7 (6.8)	93.9 (18.4)	49 (23.2)	55.6 (27.7)	97.1 (8.7)	53 (78.6)	100 (0)	53 (80)
Black Canyon Of The Gunnison National Park	61.8 (54.7)	76.8 (32.6)	99.2 (1.2)	28.7 (38.4)	76.5 (25.3)	73.3 (34)	60.2 (50.8)	53 (42.7)
Blue Ridge Parkway	69.7 (21.3)	28.8 (22.3)	70.1 (14)	20.4 (29.9)	41.4 (24.4)	78.1 (30.6)	49.3 (24.6)	46.6 (61.1)
Bluestone National Scenic River	80.3 (13.7)	63.4 (48.9)	89.8 (17.3)	23.5 (25.7)	52.4 (41.4)	93.7 (9.8)	43.9 (22.4)	71.3 (18.1)
Booker T. Washington National Monument	83.1 (14)	57.5 (54.2)	22.1 (14.2)	48.7 (77.8)	68.5 (41.6)	75.2 (39.9)	52.7 (18)	44.4 (67.3)
Boston Harbor Islands National Recreation Area	98.9 (0.2)	95.3 (3.9)	97.3 (5.1)	56 (53.6)	98.9 (0.2)	95.9 (5.3)	94.2 (16.1)	57.6 (25.3)

Park	Bio12	Bio13	Bio14	Bio15	Bio16	Bio17	Bio18	Bio19
Bryce Canyon National Park	54 (5.3)	67.1 (38)	51.1 (82.1)	62.6 (50)	73.2 (27.8)	31.6 (40.4)	34.6 (34.3)	73.4 (61.2)
Buck Island Reef National Monument	99.6 (1.1)	37.6 (58)	64.6 (11.7)	18.3 (24.7)	59.3 (50.7)	99.2 (2.4)	66.1 (46.4)	90.4 (11.2)
Buffalo National River	34.5 (23.6)	27.3 (24.7)	44.8 (59.2)	14.5 (21.8)	18.1 (13.7)	78.4 (41.1)	1.7 (1.8)	58 (57.9)
Cabrillo National Monument	30.8 (9.2)	38.1 (21.7)	19.1 (37.6)	67.7 (45.1)	36.5 (6.6)	3.7 (7.7)	29 (37.4)	46.4 (16)
Canaveral National Seashore	9.5 (10.2)	39.1 (48.9)	52.9 (27.1)	70.6 (60.2)	38.3 (30.5)	34.3 (27.1)	21.1 (29.9)	42.6 (49.8)
Canyon De Chelly National Monument	30.5 (39.8)	44.1 (13.5)	34.9 (39.7)	57.5 (49.8)	22.2 (18.7)	12.9 (21.2)	10.5 (9.1)	92 (7.9)
Canyonlands National Park	66.6 (20.4)	44.8 (40)	99.2 (1.2)	8.5 (19.2)	59.7 (30.9)	81 (32.1)	26.3 (53.7)	83.7 (13.4)
Cape Cod National Seashore	98.9 (0.2)	97.8 (0.5)	98.5 (1.4)	53.6 (26.2)	97.8 (3.3)	93.1 (14.6)	89.2 (29.1)	62.1 (20.5)
Cape Hatteras National Seashore	84.2 (13.1)	84 (23.3)	76.3 (41.4)	66.7 (42.8)	83.8 (21.1)	56.3 (10.7)	63.4 (39.7)	48.4 (30.6)
Cape Krusenstern National Monument	93.6 (11.7)	78.6 (42.7)	63.3 (27.4)	23.2 (3)	79.3 (42.7)	78.1 (18)	86.6 (6.5)	96.1 (4)
Cape Lookout National Seashore	85.5 (13.1)	84.8 (21.2)	98.5 (1.4)	68.8 (40.8)	90.8 (14.3)	67.5 (13.5)	79.1 (25.8)	65.6 (4)
Capitol Hill Parks	92.1 (2.9)	69.8 (50.7)	64.5 (19.6)	47.7 (40.4)	97.2 (3.8)	69.3 (27.9)	38.3 (32.2)	36.5 (37.5)
Capitol Reef National Park	31.7 (10.3)	13.7 (26.6)	95.8 (9.7)	15.4 (41.8)	29.4 (40.1)	79.1 (51.5)	18.6 (11.3)	49 (42.4)
Capulin Volcano National Monument	25.8 (38.5)	41.5 (38.2)	58.1 (49.6)	42 (31.3)	27.3 (41.1)	45.9 (49.7)	39.1 (29.9)	22.1 (35.1)
Carl Sandburg Home National Historic Site	35.7 (22)	7.9 (20.3)	72.7 (6.5)	9.9 (13.6)	18.2 (18.8)	85.2 (9.6)	36.8 (39.7)	38.6 (55.6)
Carlsbad Caverns National Park	47.8 (37.1)	45.5 (31.6)	80.2 (19.7)	51.4 (26)	56.9 (31.2)	48 (38.1)	59.2 (36.4)	68.7 (36)
Casa Grande Ruins National Monument	12.1 (30.3)	11.2 (25.6)	17.8 (17.1)	78.2 (15.4)	19.6 (27.7)	4.7 (9.5)	14.8 (31.5)	36.5 (29.4)
Castillo De San Marcos National Monument	10.6 (15.9)	55.8 (25.8)	42.3 (22.4)	76.8 (41.5)	30.3 (16.1)	24 (31.7)	21.7 (39.1)	41.1 (27.7)
Catoctin Mountain Park	97.5 (2.8)	91.2 (3.5)	98.8 (2.4)	45.4 (19.1)	96.5 (5.8)	97.5 (4.4)	43.4 (38.7)	81.9 (26.7)
Cedar Breaks National Monument	52.2 (27)	78.1 (25.1)	34.9 (42)	76.9 (37.3)	69.5 (47.4)	47.7 (61.5)	42.3 (19.9)	92.1 (21.7)
Chaco Culture National Historical Park	49.5 (60.4)	37.7 (60.5)	40 (57.8)	41.9 (50)	46.3 (61.4)	26.3 (22)	50.9 (79)	68.7 (23.9)
Channel Islands National Park	68.9 (48.6)	63.7 (55.2)	33.7 (62.9)	48.1 (39.5)	72.5 (45.9)	25.6 (35.6)	35.5 (35.5)	72 (34.9)
Charles Pinckney National Historic Site	24.2 (34.7)	11.8 (28.2)	63 (47.9)	7.7 (16.1)	11.7 (17.1)	35.7 (5.5)	6.6 (11.5)	31.2 (39.7)
Chattahoochee River National Recreation Area	13 (14.2)	2.5 (2.8)	87.8 (12.6)	4.8 (6.6)	3.9 (4.7)	60.4 (21.3)	43.8 (49.6)	3.1 (4.7)
Chesapeake and Ohio Canal National Historical Park	96.9 (4.7)	83.1 (8.4)	84.2 (11.7)	32.5 (36.7)	92.7 (13.6)	94.8 (9.7)	37.6 (30.5)	85.4 (23.1)
Chickamauga and Chattanooga National Military Park	50.4 (25.3)	6.7 (13.4)	55.3 (22.2)	13.9 (23.6)	3.8 (4.6)	74.6 (14.6)	46.5 (27)	12.9 (11.9)
Chickasaw National Recreation Area	54.9 (50.4)	27.5 (8.9)	70.8 (21.5)	19.8 (38.6)	36.7 (34.8)	60.3 (62.7)	74.4 (22.2)	64.5 (69.2)
Chiricahua National Monument	41.4 (45.6)	40.2 (42.9)	71.6 (37.9)	49.6 (38.1)	60.1 (28.9)	46.5 (51.2)	41.4 (44.6)	59.7 (35.4)
City Of Rocks National Reserve	57.9 (19.4)	85.3 (20.7)	62.4 (29.4)	79.4 (26.5)	68 (13.8)	47.5 (33.7)	20.2 (24)	29.2 (39.3)
Colonial National Historical Park	94.9 (5.1)	96.8 (1.5)	34.9 (15)	84.3 (2)	97.3 (4.7)	63.8 (29.7)	34.8 (49.4)	47.9 (26.5)
Colorado National Monument	54.6 (23)	36.4 (15)	99.2 (1.2)	12.5 (18)	54.4 (12.6)	70.2 (39)	44.2 (42.5)	58.5 (36.5)
Congaree National Park	35 (27.5)	26.7 (54.5)	92.5 (9.5)	12.1 (17.3)	47.6 (45.8)	47.1 (14.6)	52 (46.9)	46 (32)
Coronado National Memorial	27.4 (54.4)	67.9 (23.6)	34.3 (24.1)	73.2 (48.4)	41 (20.5)	32.3 (31.2)	19.3 (21.4)	36.1 (37.7)
Cowpens National Battlefield	7.6 (18.3)	3.7 (7.7)	86.5 (7.8)	7.6 (7.8)	4.1 (7.7)	74.3 (25.7)	28.5 (44.5)	3.5 (3.6)
Crater Lake National Park	60.8 (20.1)	49 (33.6)	24.8 (10.7)	32.3 (26.1)	62.1 (32.7)	40.3 (44)	14.4 (21.9)	39.9 (25.9)
Craters Of The Moon National Monument and Preserve	72.8 (26.7)	88.4 (17)	71.2 (13.8)	59.4 (36.1)	91 (7.7)	28.7 (28.5)	12.1 (28)	77.4 (29.7)
Cumberland Gap National Historical Park	15.9 (26.2)	12 (4.9)	78.9 (18)	35.3 (25.2)	6.7 (3.4)	54.9 (30)	9 (18.2)	22.6 (8.9)
Cumberland Island National Seashore	22.1 (8.3)	42.5 (13.6)	50.3 (23.3)	72.5 (23.8)	31.2 (5.3)	30.2 (22)	27.3 (19.3)	49.6 (33.7)
Curecanti National Recreation Area	68.8 (48.6)	90.8 (5.2)	98.9 (0.2)	55 (65)	83.3 (15.5)	80 (32.5)	62.5 (33.4)	65.7 (36.7)
Cuyahoga Valley National Park	98.9 (0.2)	94.3 (14.1)	98.9 (0.2)	2.4 (3.8)	97.1 (4.4)	99.3 (1.1)	70.8 (21.2)	100 (0)

Park	Bio12	Bio13	Bio14	Bio15	Bio16	Bio17	Bio18	Bio19
Death Valley National Park	75.9 (12.4)	78.5 (10.2)	52.7 (20.4)	72.1 (26.1)	85.3 (8.8)	59.4 (47.7)	51.6 (27.3)	97 (5.1)
Delaware Water Gap National Recreation Area	99.3 (1.1)	98.9 (0.2)	91.8 (8.2)	85.6 (9)	99.3 (1.1)	94.9 (7.4)	92.2 (15.1)	92.7 (13.3)
Denali National Park and Preserve	25.8 (12.6)	88.4 (17)	60.5 (46.3)	95.3 (11.7)	76.5 (24.4)	7.6 (18.3)	70.5 (38.2)	19.5 (35.5)
Devils Postpile National Monument	50.3 (45.2)	78.9 (47.3)	9.7 (12)	96 (7.5)	63.5 (48.1)	2.6 (3.7)	13.5 (32.7)	73.2 (44.9)
Devils Tower National Monument	12.9 (11.2)	74 (34.6)	9.3 (19.2)	96 (8.7)	39.5 (35.7)	5.1 (6.3)	11.9 (11.6)	4.5 (4.4)
Dinosaur National Monument	67.7 (27.8)	51.3 (50.6)	93.6 (12.4)	4.9 (7.4)	70 (39.3)	93.1 (3.7)	29.6 (70.4)	94 (8.6)
Dry Tortugas National Park	85.1 (35)	18.6 (30.7)	20.6 (5.2)	8.3 (10.3)	75 (54.4)	16.8 (24.9)	70.2 (51.7)	32.4 (50.6)
Ebey's Landing National Historical Reserve	70.4 (4.1)	64.6 (33.3)	24.3 (20.6)	45.7 (25.3)	61.1 (4.6)	24.8 (20.6)	9.6 (3.2)	11.7 (17.1)
Effigy Mounds National Monument	92.4 (8.3)	84.1 (18.6)	76.5 (36.8)	27.7 (50.5)	88.6 (11.2)	88.1 (21.7)	86.6 (20.2)	92.3 (15.7)
Eisenhower National Historic Site	99 (1.9)	92 (5.6)	99.2 (1.2)	29.2 (27.6)	98.2 (2.9)	98.9 (0.2)	51.1 (45.1)	82.8 (24.8)
El Malpais National Monument	46.3 (61.1)	45 (74.9)	14.6 (33.9)	61 (28.8)	45.9 (61.6)	32.3 (17.1)	51.5 (76.1)	58.5 (22.5)
El Morro National Monument	30.8 (58)	44.3 (77.3)	15.5 (32.7)	71 (19.8)	32 (64.1)	19.1 (16.7)	38.2 (66.9)	42.4 (36.8)
Eugene O'Neill National Historic Site	75.1 (39.1)	84.6 (34.7)	43.6 (60.2)	75.4 (44.7)	71.8 (57.8)	47.7 (19.5)	18 (26.5)	77 (50.3)
Everglades National Park	98.1 (5.8)	85.7 (21.9)	41.6 (31.6)	44.5 (58.6)	99.7 (1)	53.5 (75.7)	100 (0)	45.3 (72.5)
Fire Island National Seashore	99.3 (1.1)	96.7 (8.6)	93 (13)	59.7 (25.8)	100 (0)	98.2 (1.3)	96.8 (9.7)	64.3 (27.6)
Florissant Fossil Beds National Monument	49.8 (29.6)	73 (8.4)	96.5 (4.7)	42.1 (35.2)	26.2 (22.7)	77.4 (21.7)	64.7 (36.3)	66.7 (53)
Fort Bowie National Historic Site	42.4 (42.7)	41.1 (46.8)	71.8 (43.3)	46 (38.3)	57.7 (29.8)	45.4 (50)	44.1 (49.9)	62.1 (31.7)
Fort Caroline National Memorial	25.7 (2.8)	70.1 (24)	49.6 (13.7)	81.1 (39.9)	42.1 (6.6)	30.6 (23.5)	38.5 (25.9)	44.7 (36.2)
Fort Davis National Historic Site	46.4 (50)	52.7 (31.6)	78.8 (48.5)	77.1 (8.1)	57.7 (32.4)	40.3 (34.3)	66 (46.8)	33.7 (42.8)
Fort Donelson National Battlefield	59.5 (19.3)	49.2 (42.2)	37.4 (42.3)	42.9 (36.6)	52.4 (20.7)	71.7 (29.9)	46.3 (29.7)	9.9 (5.5)
Fort Frederica National Monument	15.2 (12.7)	16.7 (4.7)	55.2 (19.7)	53.4 (12.4)	9.2 (6.5)	29.7 (21.9)	6 (13.4)	49.6 (32.5)
Fort Laramie National Historic Site	58 (38.8)	30.1 (6.7)	39.6 (37.1)	40.3 (5.1)	23.2 (9.8)	61 (24)	75 (21.7)	21.1 (12)
Fort Larned National Historic Site	69.2 (44.2)	21.1 (42)	84 (24)	1.1 (0.2)	36.2 (48)	60.3 (35.8)	66.6 (35)	79.7 (33.3)
Fort Matanzas National Monument	8.6 (9.9)	55.8 (21)	45.7 (22.9)	77.6 (41.4)	30.1 (17.2)	22.9 (34.9)	22.9 (39.9)	41.7 (22.4)
Fort Moultrie National Monument	23 (32.3)	10.9 (25.6)	60.5 (47.4)	7.3 (14.9)	10.9 (14.7)	34.5 (2.5)	7.9 (14.5)	29.3 (37.3)
Fort Necessity National Battlefield	97 (6.5)	29.4 (20.5)	94.9 (7.5)	3.4 (5.7)	49.7 (18.3)	99.6 (1.1)	43.2 (15.3)	87.4 (1.6)
Fort Point National Historic Site	76.7 (50.5)	86.6 (31)	12.5 (26.6)	85.2 (37.9)	73.6 (58)	23.1 (45)	12.6 (18.5)	79.5 (46.4)
Fort Pulaski National Monument	13.1 (25)	1.1 (0.2)	63.9 (13.8)	5.2 (5.5)	1.4 (0.9)	47.4 (21.1)	3 (5.7)	34.2 (34.1)
Fort Raleigh National Historic Site	80.5 (16.3)	64.8 (51)	45.4 (35.6)	71.9 (51)	81.7 (19.2)	14.3 (22.1)	31.1 (19.2)	37.2 (38.6)
Fort Sumter National Monument	25.8 (29.8)	11.7 (26)	56.7 (51.6)	6.5 (10.3)	12.4 (16.1)	35.6 (4.3)	9.8 (14.4)	31.8 (37.7)
Fort Union National Monument	59.1 (58.6)	28.8 (44.8)	47.8 (34.8)	3.6 (7.6)	32.2 (67.7)	85.3 (21)	39.2 (63.8)	89.2 (21.4)
Fort Union Trading Post National Historic Site	85.4 (25.7)	67.6 (54.3)	92.8 (18.4)	31.3 (45)	87.3 (20.5)	91.7 (15.4)	39.6 (60.7)	70.7 (45.2)
Fort Vancouver National Historic Site	57.1 (18.5)	14.3 (17.8)	37.7 (11.1)	10.5 (9.3)	51.5 (34.1)	24.9 (13.6)	13 (6)	35.8 (27.6)
Fort Washington Park	81.8 (8.9)	62.6 (47)	56.6 (28.2)	49.5 (40.2)	97.5 (2.5)	61.8 (31.4)	32.8 (26.1)	21.2 (20.2)
Fossil Butte National Monument	75.6 (40)	87.6 (15.2)	26.8 (36.1)	71.3 (21.8)	85.8 (18.8)	47.5 (60.3)	29.4 (60.7)	62.8 (69.5)
Frederick Douglass National Historic Site	90.3 (4.1)	66.6 (49.9)	63.4 (19.4)	47.2 (42.6)	96.8 (3.8)	67.8 (26.9)	38.3 (32.2)	30.3 (25.2)
Fredericksburg and Spotsylvania National Military Park	85.9 (7.6)	79.6 (31.4)	71.9 (27.6)	20.3 (28)	94.1 (16.5)	66.5 (37.3)	19.9 (17.2)	46.5 (37.6)
Friendship Hill National Historic Site	95.3 (4.6)	41.6 (1.3)	89 (23.1)	4.4 (6.6)	65.8 (7.5)	100 (0)	34.5 (8.7)	88.4 (7.1)
Gates Of The Arctic National Park and Preserve	96.2 (5.7)	89.9 (21.2)	65.2 (17.8)	75.6 (48.1)	93.9 (13.6)	56.1 (35.5)	91.9 (19.4)	49.6 (45.1)
Gateway National Recreation Area	98.6 (3.2)	96 (8.7)	97.7 (3.6)	39.3 (29.9)	95.1 (11.8)	98.5 (1.4)	87.4 (28.3)	63.2 (21.1)

Park	Bio12	Bio13	Bio14	Bio15	Bio16	Bio17	Bio18	Bio19
Gauley River National Recreation Area	74.7 (15.5)	79.6 (33.5)	75.6 (20.6)	38.9 (25.3)	78.2 (27.7)	88 (17.7)	38.9 (30.1)	64.7 (10.7)
George Washington Birthplace National Monument	95.1 (3.2)	62.4 (43.6)	33.1 (30.6)	28.1 (12.7)	93.8 (9.5)	63.9 (29.5)	24.2 (13.5)	50 (45.6)
George Washington Carver National Monument	86.5 (23.8)	51 (31.8)	64.6 (33.4)	21.2 (15.3)	79.8 (37.1)	77.8 (30.6)	49.5 (30.1)	79.6 (30.4)
George Washington Memorial Parkway	92.7 (4.8)	80.9 (34.3)	66.8 (15.8)	59 (26.6)	96.2 (5.7)	67.5 (33.3)	29.7 (32.1)	39.2 (42.5)
Gettysburg National Military Park	98.7 (2.9)	90.4 (5.2)	99.2 (1.2)	29.2 (25.4)	98.2 (2.9)	98.9 (0.2)	53.6 (43.4)	86.9 (24.1)
Gila Cliff Dwellings National Monument	61.5 (55.6)	84 (42.7)	51.7 (27.8)	67.3 (20.2)	85.3 (29.9)	40.6 (19.2)	78.1 (48.3)	63.1 (23.1)
Glacier Bay National Park and Preserve	79.8 (45.2)	52.1 (50.4)	92.9 (12.6)	22.8 (28)	57.6 (44.6)	70.7 (32.9)	75.1 (45)	98.6 (4.3)
Glacier National Park	30.7 (10.2)	47.8 (27.7)	75.4 (14.9)	63.8 (41.3)	57.4 (16.2)	10.5 (19.5)	41.6 (21.4)	3.4 (5.6)
Glen Canyon National Recreation Area	45.1 (17.6)	49.7 (52.9)	90.9 (15.3)	49.9 (43.8)	61.9 (44.3)	45.8 (62.3)	21.4 (27.9)	58.8 (50.2)
Golden Gate National Recreation Area	78.2 (48.5)	85.3 (34.8)	25.7 (27.6)	83.2 (41.7)	73.3 (59)	21.6 (43.8)	7.4 (12.2)	80.6 (45.4)
Golden Spike National Historic Site	68.5 (10.2)	60.4 (5.8)	84.7 (14.1)	12.1 (16)	71.5 (14.8)	51.7 (33.3)	28.6 (47)	56.8 (69.7)
Grand Canyon National Park	49.9 (42.2)	68.8 (6.9)	13.9 (37.5)	95.7 (12)	75.2 (3.9)	14.8 (32.7)	51.8 (74.6)	53.5 (47.5)
Grand Portage National Monument	51 (36.2)	31 (14.2)	51.3 (14.7)	41.8 (6)	52.1 (16.7)	48.7 (22.6)	51.2 (37.1)	29.6 (47.1)
Grand Teton National Park	82.3 (29.5)	74.8 (9.6)	84.8 (27.6)	14.7 (16.8)	90.1 (23.1)	66.9 (32.3)	12.9 (5.3)	92.2 (18.4)
Grant-Kohrs Ranch National Historic Site	73.3 (14.6)	52 (3.9)	39.6 (46.2)	46.6 (49.2)	78.4 (26)	29.9 (54.6)	57.8 (61.8)	24.6 (42.3)
Great Basin National Park	71.8 (6.3)	50.6 (18)	37.7 (30.4)	21.1 (9.2)	72.7 (11.6)	66.2 (8.6)	42.7 (34.2)	74.3 (26.6)
Great Sand Dunes National Park and Preserve	42 (60.6)	38.4 (30.4)	92.5 (10.4)	15.5 (26.8)	21.4 (26.2)	86.5 (24.2)	36.9 (19.8)	68.8 (71.4)
Great Smoky Mountains National Park	38.6 (21.1)	3.4 (5.7)	60.2 (30.4)	24 (22.5)	2.1 (1.8)	58.3 (22.2)	21.2 (17.3)	27.5 (22)
Greenbelt Park	95.3 (1.5)	69.1 (47.3)	69.8 (26.1)	38.4 (38.7)	95.2 (6.6)	77 (19.9)	44.2 (35.9)	48.9 (55.5)
Guadalupe Mountains National Park	53.5 (37.6)	56.4 (29.3)	84 (13.2)	60.3 (36.5)	63.8 (28.3)	37.3 (31.1)	67.9 (41.6)	62.9 (45.5)
Guilford Courthouse National Military Park	77.9 (8.8)	57.8 (53.3)	25.7 (14.3)	51.4 (53.3)	68.3 (36.5)	51.5 (53.3)	60.7 (37.5)	17.4 (35.4)
Gulf Islands National Seashore	52.4 (62.3)	76.4 (31.1)	20.2 (23.3)	95.4 (11.7)	76.4 (30.2)	44.3 (55.8)	92.6 (13.4)	37.1 (45.4)
Hagerman Fossil Beds National Monument	37.1 (4.8)	38.6 (22.1)	57.8 (8)	43.8 (43.2)	40.6 (7.2)	19.1 (14.1)	5.7 (12.2)	39.9 (36.4)
Haleakala National Park	1.1 (0.2)	34.9 (86.4)	35.2 (27.1)	92.1 (20.5)	24.4 (48.3)	1.4 (0.9)	1.1 (0.2)	44.8 (71)
Harpers Ferry National Historical Park	88.5 (4.7)	69.9 (9.9)	64.1 (13.7)	54.2 (23.3)	83.7 (12.7)	75.6 (35.3)	20.6 (34.7)	59.3 (43.8)
Hawaii Volcanoes National Park	1.1 (0.2)	1.7 (1.8)	18.4 (17.8)	41 (19.1)	1.4 (0.9)	18.8 (13.8)	1.5 (1.4)	31.6 (59.5)
Herbert Hoover National Historic Site	92.4 (7.6)	71.8 (36.4)	99.6 (1.1)	1.1 (0.2)	84.4 (25.9)	99.2 (1.2)	95.4 (7.7)	98.5 (1.4)
Homestead National Monument of America	76.8 (28.9)	55 (45.8)	37.1 (45.5)	14.7 (21.6)	67.3 (58.6)	73 (35.1)	60.3 (33.2)	79.3 (25.7)
Hopewell Culture National Historical Park	85.2 (22.9)	50.5 (55.3)	93 (13.3)	5.8 (8.6)	29.4 (50.3)	100 (0)	10.5 (17.2)	74.2 (30.7)
Hopewell Furnace National Historic Site	99.3 (1.2)	98.9 (0.2)	95.6 (2.5)	75.8 (10.8)	97.1 (5.5)	96.8 (1.5)	80.9 (33.7)	78.8 (16.2)
Horseshoe Bend National Military Park	6.3 (3.9)	1.1 (0.2)	88.4 (26)	6.1 (9.2)	3.5 (3.6)	71 (19.6)	23.2 (30.3)	10 (10.1)
Hot Springs National Park	64.4 (27.1)	39.8 (51.2)	46.9 (58.2)	13 (18.8)	35.1 (29.5)	75.8 (16.5)	14.8 (39.7)	68.4 (57.6)
Hovenweep National Monument	57.6 (28.1)	68.3 (26)	66 (53.5)	66.4 (34.1)	70.9 (28.8)	21.8 (38.8)	30.7 (71.7)	79.8 (42.6)
Hubbell Trading Post National Historic Site	10.6 (26.6)	22.8 (19.4)	20.5 (26.7)	67.4 (49.5)	4.9 (8.6)	10.2 (15.8)	8.7 (10)	82.5 (13.9)
Indiana Dunes National Lakeshore	85.1 (20)	90.7 (5.7)	97.3 (3.6)	16.2 (18.5)	75.3 (21.7)	96 (6.6)	90 (18.3)	89.5 (14.2)
Isle Royale National Park	54 (45)	34.2 (45.2)	39.5 (9.2)	57.8 (12.5)	58.6 (16.3)	34.6 (24.4)	42.8 (43.5)	33.4 (46.8)
Jean Lafitte National Historical Park and Preserve	44.1 (59.9)	90.8 (9.3)	12.6 (22.6)	99.4 (1.9)	79.3 (22.4)	1.4 (1.2)	96.9 (6.5)	13.8 (31.5)
Jewel Cave National Monument	77 (39.2)	61.5 (59.8)	87.5 (30.4)	8.2 (10.9)	53.8 (40.5)	86 (10.7)	46.7 (25.2)	77.1 (23.9)
John Day Fossil Beds National Monument	72.1 (13.4)	52.9 (17)	15.9 (33.2)	36.6 (47.7)	75.4 (32.4)	8.2 (14.6)	7.8 (19.4)	75.8 (32.2)
John Muir National Historic Site	80.3 (34.6)	87.5 (26)	51.4 (70.6)	76.4 (41)	73.2 (56)	46.9 (19.5)	24.1 (26.8)	79.2 (47.4)

Park	Bio12	Bio13	Bio14	Bio15	Bio16	Bio17	Bio18	Bio19
Johnstown Flood National Memorial	99.7 (1)	38.4 (30)	98.3 (3.9)	2.8 (1.7)	55.4 (44.7)	100 (0)	60.7 (47.2)	95 (3.9)
Joshua Tree National Park	6.5 (12.5)	3.3 (6.7)	45.7 (5.7)	79.7 (20.6)	12.1 (4.9)	14.7 (6.1)	5 (1.5)	27.9 (22)
Kalaupapa National Historical Park	2.1 (2.8)	21.6 (56.1)	43.1 (27.1)	78.5 (31.4)	13.1 (34.7)	2.1 (2.8)	1.4 (0.9)	34.8 (78.7)
Kaloko-Honokohau National Historical Park	1.1 (0.2)	3.4 (5.6)	40.7 (13.4)	49.8 (21.4)	2.4 (3.8)	14.4 (7.6)	1.1 (0.2)	38.4 (59.3)
Katmai National Park and Preserve	61.1 (18.4)	88.8 (23.4)	46.2 (39.1)	82.5 (32.8)	68.6 (15.3)	23.6 (16.2)	59.4 (13.1)	69.7 (22.8)
Kenai Fjords National Park	55.6 (35.1)	81.1 (32.6)	69.2 (31.2)	63.6 (16.4)	62.9 (24.1)	41.4 (24.3)	42.7 (9.8)	85 (10.7)
Kennesaw Mountain National Battlefield Park	12.3 (24.5)	3.4 (5.7)	79.3 (30.7)	6.5 (11.4)	4.3 (4.9)	58.9 (23.3)	45.6 (53.8)	2.7 (4.7)
Kings Mountain National Military Park	3.1 (4.7)	2.1 (1.8)	82.4 (11.7)	9.9 (10.3)	3 (5.7)	30.3 (45.2)	20.4 (41)	2.4 (3.8)
Klondike Gold Rush National Historical Park	96.8 (9.7)	65.3 (57)	42.4 (26.7)	40.1 (51.1)	78.5 (46.5)	76.7 (23.5)	94.9 (7.8)	83.4 (22.4)
Knife River Indian Villages National Historic Site	99.3 (1.2)	74.4 (44.6)	74.8 (43.2)	11.6 (21.2)	89.1 (18.1)	93.1 (12.5)	89.7 (21.4)	94.5 (9.6)
Kobuk Valley National Park	100 (0)	82.8 (40.8)	77.7 (15.6)	18.2 (4.8)	91.5 (23.3)	91.2 (13.2)	89.3 (22.1)	98.9 (2.2)
Lake Clark National Park and Preserve	42.9 (15.1)	83 (25.5)	61.6 (30)	73.9 (25.5)	45.5 (18.8)	12 (19.3)	38.8 (10.2)	69.1 (27.3)
Lake Mead National Recreation Area	39.7 (38.2)	73.1 (1.7)	15.4 (41.1)	99.7 (1)	78.5 (3.3)	2.4 (3.8)	12.6 (21.1)	72 (12.5)
Lake Meredith National Recreation Area	25.9 (31.2)	21.4 (12.7)	43.3 (38.2)	24.4 (28.5)	9 (4.4)	76.1 (42.8)	33.2 (24.8)	64.3 (63.8)
Lake Roosevelt National Recreation Area	70.8 (20.3)	42.5 (7.3)	65.6 (13)	21.6 (5.8)	67.2 (23.9)	34.4 (10.9)	34 (37.2)	53.7 (30.5)
Lassen Volcanic National Park	83.2 (35.9)	87.1 (20.2)	23.6 (29.8)	83.8 (36.9)	84.1 (36.9)	26.5 (39.2)	29 (29.3)	86 (23.1)
Lava Beds National Monument	65.4 (53)	62.6 (55.2)	32.9 (48.6)	44 (67.9)	62.5 (51.4)	13.4 (13.7)	7.9 (20.7)	61.5 (57.7)
Lewis and Clark National Historical Park	59 (33.9)	40.1 (34.7)	30.6 (15.2)	37.1 (23)	51.4 (45.8)	12.1 (13.4)	5.5 (7.4)	50.9 (40.2)
Lincoln Boyhood National Memorial	98.1 (3.6)	98.9 (0.2)	43.4 (29.9)	49.2 (23.3)	95.9 (7.7)	84.9 (17.4)	53.5 (17.9)	68.5 (20.6)
Little Bighorn Battlefield National Monument	60.5 (13.2)	71.6 (31.7)	42.7 (30.2)	84.9 (21.9)	66.2 (23.9)	6.1 (1.4)	7.7 (11.6)	3.4 (5.7)
Little River Canyon National Preserve	58.4 (19.9)	6.1 (11.4)	58 (32.8)	10.8 (19.9)	7.6 (2)	83.1 (24.6)	42 (22.4)	39.4 (39.1)
Lyndon B. Johnson National Historical Park	61 (31)	71.1 (17.4)	40.6 (51.9)	77.3 (33.5)	65.9 (39.2)	23.6 (28.6)	65.8 (18)	57.5 (35.1)
Mammoth Cave National Park	75.8 (5.1)	72.1 (26.3)	69.7 (35.3)	35.6 (54.2)	66.3 (9.7)	94 (5.5)	38.5 (55.6)	33.7 (23.2)
Manassas National Battlefield Park	75.9 (15.4)	73.6 (6.8)	72.9 (24.6)	60.6 (22.5)	86.6 (9.8)	56.7 (47.8)	14.6 (31.8)	39.3 (45.2)
Manzanar National Historic Site	65.3 (12.6)	91.2 (9.3)	25.8 (51)	71.9 (48.3)	79 (15.4)	28.4 (18.1)	35.5 (28.2)	90.3 (9)
Marsh-Billings-Rockefeller National Historical Park	100 (0)	99.6 (1.2)	98.9 (0.2)	56.7 (21.1)	100 (0)	99.3 (1.2)	100 (0)	89.5 (16.9)
Mary McLeod Bethune Council House National Historic Site	92.9 (4.9)	72.6 (51.5)	65.6 (18.9)	52.6 (32.8)	96.5 (4.7)	70.4 (29.8)	37.1 (34.6)	38.1 (42.3)
Mesa Verde National Park	47.3 (48.5)	41.8 (61.6)	70.8 (43.7)	49 (53.8)	53.7 (38.3)	15.3 (24.2)	39.8 (70.8)	60.9 (70.8)
Minute Man National Historical Park	98.9 (0.2)	95 (6.2)	89.2 (11)	62.8 (53)	98.9 (0.2)	93.7 (7)	98.9 (2.2)	73.2 (24.4)
Mississippi National River and Recreation Area	82.4 (28.2)	98.7 (3.9)	94.4 (14.6)	63.5 (47.9)	94.5 (16.5)	50.7 (34.2)	78.1 (47.6)	79 (14.8)
Missouri National Recreational River	97.8 (0.5)	96.8 (1.5)	95.8 (10.8)	54.9 (18.3)	97.9 (2)	84.8 (23.1)	72.2 (16.5)	67.7 (51.5)
Mojave National Preserve	43.3 (33)	63.1 (29.3)	51 (43.9)	85.3 (34.8)	75.5 (7.7)	6.9 (17.4)	4.2 (3)	82.7 (10.9)
Monocacy National Battlefield	93.5 (5.2)	87.1 (1.9)	79.2 (2.3)	59.5 (9.7)	89.3 (5.1)	83.8 (24.5)	29.2 (36.2)	65.2 (37.8)
Montezuma Castle National Monument	45.1 (40.3)	52.5 (23.4)	5.1 (7.3)	96.3 (7.2)	70.2 (11.6)	3.7 (5.6)	71.4 (63.4)	69.3 (37.7)
Moores Creek National Battlefield	36.7 (33.9)	60.4 (66.4)	72.6 (24.7)	52.5 (54.8)	50.7 (73.5)	39.8 (19.3)	14.7 (19.7)	22.2 (38.3)
Morristown National Historical Park	95.4 (8.5)	96.4 (7.6)	87.2 (30.1)	70.5 (21.8)	95.7 (8.5)	92.1 (4.9)	86.2 (34.5)	74.9 (20.2)
Mount Rainier National Park	67.1 (21.1)	24.2 (12)	25.2 (26.7)	9.4 (11.2)	49.2 (19.2)	35.1 (9.6)	27.4 (4.8)	24.6 (7.7)
Mount Rushmore National Memorial	56.3 (38.4)	34.9 (48)	60.6 (58.7)	16.1 (19.7)	35.6 (45.8)	53.5 (18)	33.8 (35.2)	27.3 (26.7)
Muir Woods National Monument	76.7 (48.4)	87.2 (29)	14.8 (32.7)	84.2 (38.8)	73.3 (59)	22 (45)	8.5 (12.2)	79.5 (46.4)
Natchez Trace Parkway	67.6 (15.7)	16.7 (27)	70 (31.8)	5.6 (12.4)	22.5 (48.8)	82.8 (17.9)	93.7 (6.2)	36.9 (50.8)

Park	Bio12	Bio13	Bio14	Bio15	Bio16	Bio17	Bio18	Bio19
National Capital Parks-East	93.9 (2.7)	73.4 (51.4)	65.2 (19.8)	50.4 (33.2)	97.2 (3.8)	70.8 (29.8)	38.3 (32.2)	40 (44.9)
National Park of American Samoa	60.3 (58.5)	95.8 (7.2)	55.6 (78)	84.1 (15.8)	96 (9.7)	52 (49.5)	28.6 (21.8)	54.4 (32.3)
Natural Bridges National Monument	62.8 (25.6)	72.4 (27.7)	82.9 (27.2)	63.9 (37.7)	72.8 (22)	45.6 (72.5)	20.9 (41.5)	84.2 (20.9)
Navajo National Monument	48.5 (33.5)	51.3 (30.3)	49.4 (54.9)	68.7 (28.8)	66.3 (13.8)	21 (27.4)	33.4 (51.5)	80.9 (22.6)
New River Gorge National River	76.9 (17.6)	71.3 (41.4)	82.9 (15.8)	26.2 (21.3)	66.2 (45)	89.4 (13.3)	43.9 (24.7)	69.9 (12.1)
Nez Perce National Historical Park	75.8 (44.6)	56.7 (49.5)	26.9 (51.5)	46 (37.4)	87.9 (21.7)	25.8 (29.1)	14.4 (32.8)	57.6 (65.1)
Ninety Six National Historic Site	2.1 (2.8)	2.1 (2.8)	72.8 (4.8)	16.9 (17.3)	2.4 (3.8)	3.8 (3.4)	6.3 (6.5)	1.7 (1.8)
Niobrara National Scenic River	82.3 (15.6)	77.8 (20.5)	74.2 (20.1)	54.4 (13.7)	82.1 (31.3)	52.3 (25)	69.4 (28.8)	50.1 (32.8)
Noatak National Preserve	99.7 (1)	85.1 (34)	74.6 (10.8)	20.5 (4.1)	92.2 (21.4)	93.1 (10)	88.5 (19.9)	98.5 (3.2)
North Cascades National Park	81.3 (17.1)	52 (8.1)	14.9 (15.2)	26.6 (38.2)	70.8 (18.3)	37.2 (16.7)	38.9 (34.8)	51.9 (13.6)
Obed Wild and Scenic River	61.4 (6.2)	12 (8.3)	62.3 (23.2)	15.6 (17.9)	34.2 (24.2)	70.5 (32.7)	15.3 (21.9)	49.5 (22.4)
Ocmulgee National Monument	4.6 (10.6)	1.1 (0.2)	69.8 (56.4)	10.4 (10.4)	5.5 (8.5)	9.4 (9)	53.7 (30)	23 (11.6)
Olympic National Park	61.6 (38.6)	47.6 (34.1)	39.8 (28.2)	35.4 (17.6)	55.5 (37.3)	20 (17.4)	11.6 (16.3)	28 (30.2)
Oregon Caves National Monument	48.4 (31)	56.8 (47.6)	30 (25.7)	60.7 (53.3)	47.7 (30.8)	23.1 (23.8)	11.4 (17.6)	53.1 (35.6)
Organ Pipe Cactus National Monument	7.7 (15.1)	8.7 (9.2)	72.3 (2.7)	74.8 (29.7)	9.1 (14.8)	6.5 (8.3)	6.3 (13.4)	11.9 (17.2)
Ozark National Scenic Riverways	89 (12.9)	64.5 (40.8)	69 (50.2)	12.6 (29.6)	80.7 (20.2)	90.7 (21.1)	40 (38)	72.3 (47.4)
Padre Island National Seashore	59.5 (10.7)	63.1 (30.4)	52.1 (56.9)	51.1 (65.1)	72.6 (23.2)	16 (34)	75.6 (20.1)	57.7 (46.6)
Palo Alto Battlefield National Historical Park	54.8 (16.2)	66.4 (28.1)	56.1 (37.7)	65.6 (60.2)	68.3 (8.2)	28.5 (53.9)	68.1 (31)	59.8 (35.3)
Pea Ridge National Military Park	40.4 (36.2)	28.7 (12.5)	55.1 (57.2)	29 (29.7)	19.2 (25.7)	57 (49.4)	8.2 (12.4)	54.9 (71.4)
Pecos National Historical Park	51 (62.7)	24.6 (46)	9 (16.4)	16.9 (14.2)	23.7 (52)	63.5 (59.2)	23.4 (53.2)	90.2 (19.2)
Petersburg National Battlefield	98.5 (1.4)	91.7 (11)	52.6 (22.9)	62.1 (11.2)	97.7 (6)	86.8 (29.1)	40 (33.5)	57.8 (16.9)
Petrified Forest National Park	7.9 (20.7)	15.1 (22.6)	6.1 (13.4)	86.1 (18.2)	4.5 (7.4)	4.9 (8.6)	12.6 (19.7)	19.4 (16)
Petroglyph National Monument	61.5 (44.6)	46.3 (60.4)	9.8 (25.4)	26.8 (25.7)	52.8 (44.4)	62.2 (48)	58.4 (58.1)	79 (20.5)
Pictured Rocks National Lakeshore	75.8 (24.2)	70.9 (50.8)	79.8 (15.4)	44 (33.4)	68 (31.9)	49.8 (59.4)	29.1 (23.4)	74.1 (33.6)
Pinnacles National Monument	68.6 (50.3)	74.7 (51.2)	18.7 (11.6)	54.5 (33)	65.1 (60.9)	38.8 (7.8)	34.3 (37.2)	73.1 (36.5)
Pipe Spring National Monument	74.2 (9.5)	82.2 (15.7)	20.1 (51.3)	74.2 (51.3)	82.3 (15.5)	65.3 (42.3)	67.9 (55.6)	92.6 (18.2)
Pipestone National Monument	93.9 (9.2)	91.1 (22.1)	98.6 (2.2)	28.5 (18.3)	94.7 (12.5)	98.5 (2.4)	63.1 (21.2)	96 (12)
Piscataway Park	82.6 (7.8)	62.1 (52.8)	56.2 (30.4)	47.6 (41.7)	97.1 (2.8)	63 (33.5)	30.6 (25.4)	22.8 (27.4)
Point Reyes National Seashore	78.7 (49.5)	83.4 (41.6)	32.5 (16.7)	78 (56.3)	72.9 (60)	20.6 (45)	3.3 (6.7)	80.9 (44.5)
Port Chicago Naval Magazine National Memorial	79.3 (35.5)	85.9 (30.9)	46.4 (68.6)	71.9 (36.5)	73.2 (56)	51.9 (8.8)	32.5 (13.1)	78.5 (49.3)
Presidio of San Francisco	76.7 (50.5)	86.6 (31)	12.5 (26.6)	85.2 (37.9)	73.6 (58)	23.1 (45)	12.6 (18.5)	79.5 (46.4)
Prince William Forest Park	83.7 (6.4)	69.2 (20.5)	69.5 (31.9)	44.7 (34.5)	92.1 (11.3)	62.6 (41.1)	16.2 (28.7)	37.7 (41.1)
Pu'uhonua o Honaunau National Historical Park	1.1 (0.2)	1.1 (0.2)	26.6 (18.4)	48.9 (17.2)	1.1 (0.2)	18.8 (15.4)	1.1 (0.2)	33.8 (55.9)
Puukohola Heiau National Historic Site	1.1 (0.2)	24.2 (49.2)	19.4 (10)	81.2 (25.8)	13.6 (33.9)	1.5 (1.4)	1.1 (0.2)	43.8 (67.8)
Rainbow Bridge National Monument	42.2 (27.5)	47.9 (39.8)	55.4 (45.4)	72.3 (29.3)	64.9 (27.5)	25.8 (36)	30.5 (50.1)	46.9 (61)
Redwood National Park	58.6 (36)	80.5 (40.7)	65.2 (52.6)	67.2 (45.5)	61.1 (41.5)	25.7 (21.3)	12.2 (18.5)	71.6 (41.4)
Richmond National Battlefield Park	98.5 (1.4)	92.1 (10.6)	45.5 (22.6)	61.3 (11.4)	98.9 (2.4)	80.5 (24.1)	39.4 (31.3)	56.9 (18.8)
Rio Grande Wild and Scenic River	44.4 (53.2)	45.4 (42.9)	50.8 (57.4)	83.2 (11.4)	54.4 (36.4)	21.6 (33.7)	57.6 (34.5)	26 (39.7)
Rock Creek Park	94.2 (1.6)	76.2 (44)	66.6 (19.1)	51.1 (34)	96.5 (4.7)	71.9 (29.7)	37.9 (31.2)	43 (48.8)
Rocky Mountain National Park	76.3 (28)	92.4 (13.5)	89 (24)	56.7 (77)	91.7 (0.8)	87.2 (12.7)	94.7 (6.2)	13 (26.5)

Park	Bio12	Bio13	Bio14	Bio15	Bio16	Bio17	Bio18	Bio19
Roosevelt-Vanderbilt Headquarters	99.3 (1.1)	88.2 (18.8)	79.5 (40.7)	73.9 (12.8)	98.2 (2.3)	97.2 (0.8)	96.8 (5.2)	98.9 (0.2)
Russell Cave National Monument	67.2 (24.7)	5.1 (8.5)	55.7 (18.1)	10.9 (16.8)	8 (12.8)	85.2 (14.8)	57.7 (11)	41.6 (30.7)
Sagamore Hill National Historic Site	98.2 (4.3)	91.1 (16.9)	90.1 (15.7)	53.8 (21.4)	96.1 (7.4)	98.2 (2.3)	93.9 (13.8)	69 (12.6)
Saguaro National Park	27.4 (35.1)	81.6 (25.7)	27.2 (2.3)	95.7 (10.8)	52.2 (24.3)	9.1 (5.6)	44.4 (39.1)	34.6 (32.3)
Saint Croix National Scenic Riverway	69.1 (47.6)	69.3 (52.9)	91.4 (21.4)	45 (35.7)	86.8 (32)	62.5 (31.2)	54.8 (59.6)	68.9 (50.2)
Saint-Gaudens National Historic Site	99.7 (1)	99.6 (1.2)	98.6 (0.9)	53 (27.9)	100 (0)	99.3 (1.2)	100 (0)	89 (19.3)
Salinas Pueblo Missions National Monument	35.2 (52.2)	30.2 (61.7)	15.4 (26.6)	46.9 (32.6)	32.8 (56.4)	31.7 (20.8)	43.9 (74)	29.5 (37.2)
San Antonio Missions National Historical Park	50.9 (31.3)	62.7 (4.4)	1.4 (0.9)	98.5 (3.6)	63.3 (15.3)	1.8 (1.3)	46.2 (21.8)	55.4 (58.4)
San Juan Island National Historical Park	58.7 (36.6)	62.5 (43.8)	26.8 (20.5)	38 (18.5)	52.7 (23.4)	24 (18.9)	15.2 (8.7)	11.6 (6.7)
Sand Creek Massacre National Historic Site	71.2 (45.5)	56.4 (44.1)	81.3 (34.3)	13.4 (6.4)	63.3 (55.7)	53.8 (55.4)	81.2 (26.8)	38 (55.5)
Santa Monica Mountains National Recreation Area	43.8 (15.9)	55.3 (53.8)	57.5 (74.2)	46.9 (40.9)	59.6 (43.1)	63 (55.7)	32.7 (55.4)	71.8 (35.7)
Saratoga National Historical Park	99.7 (1)	99.3 (1.2)	97.8 (2.3)	79.2 (4.1)	99.7 (1)	97.9 (2.8)	98.6 (0.9)	97.9 (3.9)
Saugus Iron Works National Historic Site	98.9 (0.2)	94.6 (6.7)	95.1 (7.5)	57 (61.1)	98.9 (0.2)	93.9 (8.9)	96 (10.8)	61.4 (19)
Scotts Bluff National Monument	84.4 (9.6)	59.8 (28.8)	47.6 (48.6)	36.5 (23.9)	50.1 (13.9)	75.1 (4.3)	81.3 (32)	24.6 (14.9)
Sequoia and Kings Canyon National Parks	57.8 (21.4)	80.1 (40.4)	8.4 (16.3)	73.3 (15.5)	69.6 (30.4)	5.8 (9.5)	19.8 (22.1)	82.5 (22.7)
Shenandoah National Park	84.7 (4.6)	59.7 (44.3)	80.7 (20.7)	35.3 (64.6)	67.1 (31)	84.4 (29.1)	29.2 (17.4)	76.6 (36.7)
Shiloh National Military Park	72.9 (14.2)	28 (20.7)	82.2 (19.9)	3.3 (6.7)	38 (23.2)	92 (8.8)	89.2 (9.3)	33.8 (30.7)
Sitka National Historical Park	75.2 (55.5)	16.5 (39.3)	100 (0)	4 (8.6)	24.7 (35.8)	99.7 (1)	72 (38.5)	98.6 (3.2)
Sleeping Bear Dunes National Lakeshore	87.8 (23.5)	31.8 (17.6)	90 (18.1)	2.5 (2.5)	42.4 (43.9)	97 (2.9)	75.1 (44.5)	90.5 (6.8)
Stones River National Battlefield	75.5 (15.2)	55.7 (43.1)	75 (12.9)	10.5 (9.1)	37.5 (37.2)	87.9 (11.1)	78.5 (21)	37.9 (26.3)
Sunset Crater Volcano National Monument	5 (11.5)	19.1 (27.5)	4.1 (7.7)	96 (9.7)	72 (21.6)	4 (8.6)	18.4 (30.8)	25.1 (60.9)
Tallgrass Prairie National Preserve	94.1 (6.1)	70.3 (31.9)	52.8 (43.1)	9.1 (18.2)	89.3 (15.1)	70.6 (29.8)	92.6 (6.4)	79 (32)
Theodore Roosevelt National Park	73 (39.6)	65.6 (34)	87.5 (24.3)	35.8 (42.5)	66.7 (29.7)	57.1 (38.7)	12.6 (33.4)	50.5 (65)
Thomas Stone National Historic Site	91.1 (6.9)	58.9 (49.4)	49.9 (36.2)	37.9 (40.5)	95.1 (7.7)	64.4 (35.7)	29 (21.7)	38.6 (45.7)
Timpanogos Cave National Monument	56.9 (28.6)	69.7 (14.8)	67.2 (45)	53.6 (66.7)	66.6 (12.7)	39.2 (58.8)	13.3 (33.9)	49.1 (52.1)
Timucuan Ecological and Historic Preserve	26.1 (3.1)	71.6 (21.3)	49.7 (17.8)	81.1 (38.8)	41.8 (6.6)	31.7 (23.5)	40.4 (23.5)	45.6 (33.3)
Tonto National Monument	34.4 (23.2)	45.6 (15.8)	9 (12.2)	80.9 (20.6)	49.7 (17.1)	9.6 (6.2)	51.7 (41.9)	57.8 (38.8)
Tumacacori National Historical Park	14.8 (35.2)	78.1 (11.2)	27.3 (16.6)	94.4 (16.9)	35.7 (26.6)	15.5 (27.2)	17.4 (21.9)	24.5 (30.3)
Tuzigoot National Monument	37.9 (30.2)	44.4 (22.3)	2.5 (2.8)	98.2 (2.9)	72.9 (16.3)	2.7 (4.7)	63.4 (52.7)	51.4 (55.8)
Upper Delaware National Scenic and Recreational River	99.7 (1)	97.9 (2.8)	99.3 (1.1)	78.6 (23.6)	99.3 (1.1)	98 (4.9)	95.8 (6.5)	96.8 (4.9)
Valley Forge National Historical Park	98.6 (1.2)	99.3 (1.2)	92.5 (9.4)	73.1 (25.2)	96.8 (6.6)	94.6 (4)	69.4 (44.8)	78.8 (5.8)
Valor in the Pacific National Monument	4 (8.6)	5.4 (9.5)	1.4 (0.9)	96.8 (9.7)	10 (22.1)	1.1 (0.2)	1.4 (0.9)	25.4 (40.5)
Vicksburg National Military Park	60.5 (28.6)	27.6 (47.6)	43.4 (50.8)	15.6 (34.5)	28.7 (60.2)	80.4 (21.7)	74.5 (28.5)	44.6 (43.2)
Virgin Islands National Park	99.3 (1.1)	47.9 (57.9)	36.9 (35.6)	20.5 (25.3)	91.2 (15.3)	100 (0)	64.4 (44.2)	92.9 (4.9)
Voyageurs National Park	94.4 (13.5)	57.6 (47.7)	52.3 (42.9)	34.7 (47.6)	82.5 (41.7)	46.4 (47.6)	68.4 (57.8)	55.2 (40.1)
Walnut Canyon National Monument	5 (11.5)	17 (15.4)	4.4 (8.6)	96 (9.7)	69.8 (25.2)	4 (8.6)	19.1 (32.7)	24.1 (57)
War In The Pacific National Historical Park	58.1 (33.5)	48.5 (13.4)	100 (0)	20 (17.5)	74.9 (17.8)	95.4 (7.2)	99.2 (2.4)	70.4 (15.8)
Washita Battlefield National Historic Site	60.1 (55.4)	23.9 (36.8)	32.2 (1.7)	13.5 (22.1)	26.1 (39.5)	83.6 (22.6)	71.7 (39.6)	72.6 (55.1)
Weir Farm National Historic Site	98.6 (1.2)	88.8 (11.5)	82.8 (29.1)	70.4 (3.4)	97.5 (3.1)	97.8 (3.3)	98.6 (1.2)	85.9 (9.5)
Whiskeytown National Recreation Area	82.5 (36.9)	89.1 (24.8)	24.7 (28.8)	66.2 (68)	81.4 (46.5)	29.6 (25.2)	16.1 (30.1)	57.1 (50.8)

Park	Bio12	Bio13	Bio14	Bio15	Bio16	Bio17	Bio18	Bio19
White Sands National Monument	63.2 (24.2)	77.7 (14.6)	74.1 (18.1)	67.5 (45.7)	67.4 (9.8)	53.1 (52.5)	72.5 (23.9)	55.6 (51.4)
Whitman Mission National Historic Site	81.3 (19.6)	61.6 (5.5)	25.8 (21.5)	25.8 (29)	87.8 (18.1)	20 (28.4)	15.1 (32.3)	70.4 (26.2)
Wilson's Creek National Battlefield	73.6 (32.1)	61.4 (33.4)	42.8 (63.6)	26.5 (10.6)	58.8 (49.2)	74.7 (34.3)	9.4 (2.2)	72.6 (46.4)
Wind Cave National Park	64.5 (22.9)	19.9 (32.1)	80.8 (27.7)	9 (10)	39 (48.9)	72.1 (7.4)	42.7 (22)	62.8 (25.1)
Wolf Trap National Park for the Performing Arts	90.6 (2.2)	83 (14.7)	71.7 (21)	67.3 (16.4)	92.6 (9.5)	66.9 (39.6)	23.4 (37.7)	41.5 (46.6)
Wrangell - St Elias National Park and Preserve	10.3 (23)	28.3 (31.8)	2.8 (3.8)	94.5 (7.3)	18.7 (33)	5.1 (8.5)	24 (45.4)	48.1 (36.7)
Wright Brothers National Memorial	79.8 (17.3)	64.3 (47.5)	41.8 (38.3)	76.1 (44.6)	82.1 (19.2)	13.9 (20.9)	30.5 (19.9)	35.5 (40.4)
Wupatki National Monument	5 (11.5)	15.9 (27.2)	3.8 (5.7)	96.3 (8.7)	66 (17.7)	4.3 (9.6)	19.4 (26.8)	30.1 (70.6)
Yellowstone National Park	65.4 (32.2)	59.8 (18)	72.4 (37.6)	19.5 (17.1)	78.5 (39.1)	39.5 (14.4)	20.5 (31.8)	73.6 (43.4)
Yosemite National Park	37.9 (49.3)	65.2 (75)	10.7 (19.7)	90.1 (18.2)	47.9 (60.8)	8.3 (21.9)	10.5 (25.4)	56.3 (49.8)
Yucca House National Monument	52 (40.5)	56.8 (35.1)	66.9 (46.7)	61.4 (40.2)	65.8 (32.2)	16.7 (26.6)	26.9 (61)	73.5 (60.2)
Yukon-Charley Rivers National Preserve	2.9 (3.7)	19.3 (15.9)	31.4 (34.1)	96.4 (0.7)	2.6 (3.7)	24.1 (22.8)	2.6 (3.7)	15.9 (20.9)
Zion National Park	59.1 (29.3)	89.3 (14.8)	21.1 (53.3)	94.2 (9.6)	79.6 (16.5)	38.5 (62.5)	41 (42.7)	92.4 (22.9)
