# Supplementary material for: Climate Exposure of US National Parks in a New Era of Change
Source: PLoS One. 2014 Jul 2;9(7):e101302. doi: 10.1371/journal.pone.0101302 (PMC4079655; doi:10.1371/journal.pone.0101302)
Supplement: Appendix S4 — Mean percentile and maximum difference in percentile (in parentheses) for moving window means: mean annual percentage cloud cover (Cld1), cloud seasonality (Cld4), vapor pressure of the warmest quarter (Vap18), annual number of wet days (Wet12), number of wet days of the warmest quarter (Wet18), and annual number frost days (Frs12). NA’s associated with Frs12 indicate that the variable is undefined (i.e., no frost days exist). (PDF) [file pone.0101302.s004.pdf]

Appendix S4. Mean percentile and maximum difference in percentile (in parentheses) for moving window means: mean annual percentage cloud cover (Cld1), cloud seasonality (Cld4), vapor pressure of the warmest quarter (Vap18), annual number of wet days (Wet12), number of wet days of the warmest quarter (Wet18), and annual number frost days (Frs12). NA's associated with Frs12 indicate that the variable is undefined (i.e., no frost days exist).

Park	Cld1	Cld4	Vap18	Wet12	Wet18	Frs12
Abraham Lincoln Birthplace National Historical Park	99.6 (1.1)	19.7 (24.6)	35.4 (48.8)	74.5 (7.2)	62 (5.4)	1.4 (0.9)
Acadia National Park	87.7 (12.5)	9.4 (24.2)	100 (0)	86.3 (15.5)	98.6 (4.3)	1.1 (0.2)
Agate Fossil Beds National Monument	92.6 (2.7)	47.4 (57.7)	93.4 (16.4)	65.7 (20.8)	84.9 (7.4)	8.5 (12)
Ala Kahakai National Historic Trail	100 (0)	1.1 (0.2)	77.3 (7.9)	30.3 (24.7)	24.7 (29.1)	42.4 (18.4)
Alagnak Wild River	51.2 (23.7)	16.7 (31.7)	93.1 (3.6)	86.5 (30.2)	92.6 (12.4)	11.6 (7.9)
Alibates Flint Quarries National Monument	72 (9.1)	22.3 (37.9)	97.1 (8.7)	31.8 (57.5)	67.6 (23.7)	12.3 (13.2)
Allegheny Portage Railroad National Historic Site	100 (0)	6 (13.5)	46.6 (51)	87.5 (11.5)	91.6 (4)	1.1 (0.2)
American Memorial Park	1.1 (0.2)	11.5 (31.6)	89.3 (21.5)	73.6 (36.8)	96.5 (5.8)	NA (NA)
Amistad National Recreation Area	41.7 (23.1)	63.6 (64.6)	90.8 (22.9)	60.3 (12.1)	62.7 (10.7)	11.7 (22.8)
Anacostia Park	100 (0)	7.7 (17.4)	21.1 (27.7)	96.7 (0.7)	72 (51.5)	1.4 (1.2)
Aniakchak National Monument and Preserve	32.3 (13.4)	28.7 (38.7)	83.9 (9.8)	94.5 (10.9)	58.2 (3.3)	19.2 (13.2)
Antietam National Battlefield	100 (0)	8.4 (19.3)	22.7 (23.8)	99.4 (1.9)	78.4 (32.5)	8.6 (22.7)
Apostle Islands National Lakeshore	100 (0)	4.3 (9.6)	96.8 (9.7)	33.8 (18.7)	47.9 (17.1)	1.4 (0.9)
Appalachian National Scenic Trail	100 (0)	4.3 (9.6)	61.3 (40.2)	97.3 (4.9)	98.2 (2.3)	1.1 (0.2)
Appomattox Court House National Historical Park	100 (0)	9.2 (15.2)	11.3 (16.2)	97.4 (6.8)	94.2 (8.8)	3.4 (4.6)
Arches National Park	41.4 (46.4)	39.8 (70.5)	96 (9.7)	68.1 (18.6)	54.2 (61.5)	45.9 (20.6)
Arkansas Post National Memorial	100 (0)	13.9 (23.7)	97 (7.8)	4.4 (8.6)	10.1 (27.1)	3.5 (3.6)
Assateague Island National Seashore	100 (0)	5.9 (14.5)	20.3 (27.7)	100 (0)	97.1 (7.5)	1.8 (0.9)
Aztec Ruins National Monument	68.9 (33.1)	16.9 (43.6)	99.7 (1)	51 (31.5)	55.4 (55.9)	10.7 (22.1)
Badlands National Park	98.5 (1.4)	24.2 (45)	89.8 (26)	82.6 (16.6)	93.3 (8.8)	70.1 (46.4)
Baltimore-Washington National Parkway	100 (0)	8 (18.3)	21.4 (26.7)	97.1 (1.7)	72.7 (50.3)	2.2 (3.3)
Bandelier National Monument	96 (2.5)	18.8 (49.4)	99 (2.9)	37.4 (54.6)	45.5 (78.8)	3 (5.7)
Bent's Old Fort National Historic Site	76.2 (18.1)	30.1 (61.2)	97.1 (8.7)	46.8 (55.5)	79.9 (30.1)	5.6 (7.3)
Bering Land Bridge National Preserve	33.5 (24)	36.5 (21.3)	95.8 (6.3)	43.7 (19)	63.4 (41.3)	1.8 (1.3)
Big Bend National Park	17.1 (21.1)	82.5 (11.9)	91.7 (18.1)	61.3 (11.4)	70.5 (17.6)	5.4 (8.3)
Big Cypress National Preserve	78.1 (29.6)	96.1 (11.7)	96.1 (3)	94.5 (16.5)	97.7 (5.8)	34.5 (46.9)
Big Hole National Battlefield	99.3 (1.2)	29.9 (60.7)	3.5 (4.7)	34.9 (12.6)	24.5 (32.7)	1.8 (2.3)
Big South Fork National River and Recreation Area	99.6 (1.1)	19.7 (23.5)	57 (34.6)	80.9 (14.4)	85.2 (10.7)	1.7 (1.8)
Big Thicket National Preserve	85 (24.1)	6.1 (12.5)	100 (0)	50.6 (31.8)	72.2 (25.2)	60.5 (11.3)
Bighorn Canyon National Recreation Area	74.7 (37.6)	42.6 (49.2)	51.3 (42.2)	5.7 (10.4)	14.1 (25.9)	7.4 (16)
Biscayne National Park	58.9 (52.1)	96.1 (11.7)	96.7 (0.7)	95.1 (14.6)	98.1 (5.8)	NA (NA)
Black Canyon Of The Gunnison National Park	47.5 (36.2)	33.5 (74.6)	97.7 (5.8)	67.6 (34.8)	73.1 (50.8)	83 (8.8)
Blue Ridge Parkway	99.2 (1.2)	10.8 (17.3)	10.2 (14.7)	92.6 (14.3)	95.4 (5.1)	1.4 (1.2)
Bluestone National Scenic River	99.2 (1.2)	18.3 (24.4)	1.9 (2.6)	97.7 (4.9)	96.4 (4.5)	1.4 (1.2)
Booker T. Washington National Monument	100 (0)	10.1 (13.2)	2.7 (5.1)	96 (9.7)	95.7 (5.1)	1.8 (2.3)
Boston Harbor Islands National Recreation Area	99.6 (1.1)	13.9 (32.7)	95.4 (10.7)	97.3 (3.6)	99.2 (1.2)	1.1 (0.2)

Park	Cld1	Cld4	Vap18	Wet12	Wet18	Frs12
Bryce Canyon National Park	83 (3.7)	43.3 (41)	99 (1.9)	54.9 (23.8)	52.6 (12.1)	5.9 (4.2)
Buck Island Reef National Monument	64.9 (61.7)	100 (0)	100 (0)	98.2 (0.9)	86.1 (28.2)	NA (NA)
Buffalo National River	98.5 (2.4)	17.1 (27.5)	97.3 (6.8)	28.9 (34.2)	31 (29.5)	3.1 (4.6)
Cabrillo National Monument	100 (0)	4.3 (9.6)	100 (0)	41.2 (26.5)	47.3 (14.8)	7.5 (10.6)
Canaveral National Seashore	7.3 (15.3)	95.4 (12.6)	90.3 (9.5)	80 (41)	93.8 (8.3)	68.9 (27.1)
Canyon De Chelly National Monument	69.6 (33.1)	7.3 (17.4)	96.9 (5.8)	42.4 (27.9)	38.3 (18.1)	10.9 (14.8)
Canyonlands National Park	37.1 (55.1)	49.4 (71.2)	96.3 (8.7)	60.3 (12.9)	49.2 (51.6)	26.2 (29.7)
Cape Cod National Seashore	98.6 (4.3)	12.6 (32.7)	92.7 (16.5)	97.3 (3.6)	99.6 (1.1)	1.7 (1.8)
Cape Hatteras National Seashore	100 (0)	7.5 (12.3)	18.9 (17.8)	100 (0)	77.1 (30.4)	16.3 (35.7)
Cape Krusenstern National Monument	51.5 (27.2)	14.2 (4.5)	95.8 (8.6)	42.1 (36.1)	62.9 (44.5)	1.8 (1.3)
Cape Lookout National Seashore	100 (0)	7.1 (13.4)	18.8 (13.4)	97.5 (6.5)	80.7 (27.2)	13.7 (27)
Capitol Hill Parks	100 (0)	7.7 (17.4)	21.1 (27.7)	96.7 (0.7)	72 (51.5)	1.4 (1.2)
Capitol Reef National Park	60.9 (30.3)	60.8 (42.3)	96.9 (6.8)	61.1 (10.1)	50.4 (14.8)	9.9 (15.1)
Capulin Volcano National Monument	81.7 (21.7)	32.9 (72)	96.8 (9.7)	34.4 (60.9)	65.7 (52.5)	2.4 (3.8)
Carl Sandburg Home National Historic Site	98.9 (2.2)	14.4 (25.9)	29.6 (28.1)	85.3 (13.7)	94.7 (3.9)	14.1 (16.8)
Carlsbad Caverns National Park	50.9 (10.5)	32.5 (74.6)	99.4 (1.9)	38.9 (44.9)	75.4 (35)	2.1 (2.8)
Casa Grande Ruins National Monument	97.7 (3.6)	4.3 (9.6)	97.9 (2)	38 (16.8)	35.6 (11.6)	2.6 (1.7)
Castillo De San Marcos National Monument	3.3 (6.7)	64.5 (31.2)	65.4 (24.7)	62.5 (43.1)	72.5 (10.4)	53.3 (51.9)
Catoctin Mountain Park	100 (0)	10.1 (22.2)	24.4 (22.1)	99 (2.9)	86.5 (17.6)	11.5 (31.3)
Cedar Breaks National Monument	84.9 (21.2)	46.9 (52.1)	99.7 (1)	55 (25.6)	52.2 (16.2)	5.3 (3.2)
Chaco Culture National Historical Park	94.6 (1.7)	8 (19.3)	100 (0)	44.5 (30.8)	45.5 (50.7)	4 (8.6)
Channel Islands National Park	100 (0)	5.4 (9.5)	100 (0)	59.4 (6.2)	61.1 (25.2)	1.1 (0.2)
Charles Pinckney National Historic Site	98.2 (0.9)	6.1 (12.5)	14.5 (13.7)	57 (34.9)	83.8 (28.8)	46 (17.3)
Chattahoochee River National Recreation Area	99.3 (2.2)	6.9 (12.5)	44.2 (20.8)	62.9 (35.1)	28.3 (53.3)	5.8 (7.5)
Chesapeake and Ohio Canal National Historical Park	100 (0)	6 (13.5)	22.7 (23.8)	99.7 (1)	80.6 (30.8)	1.8 (2.3)
Chickamauga and Chattanooga National Military Park	99.6 (1.1)	13.5 (20.9)	47.3 (33.7)	58.3 (35.6)	56.7 (12.2)	24.2 (25)
Chickasaw National Recreation Area	83.9 (20.4)	17.9 (29.9)	99.7 (1)	32.7 (63.6)	69.3 (28.6)	12.3 (23.4)
Chiricahua National Monument	87.4 (22.9)	4.3 (9.6)	100 (0)	19.4 (50.9)	28.5 (66.2)	1.1 (0.2)
City Of Rocks National Reserve	98.5 (1.4)	57.4 (57.8)	79.1 (37.2)	89.4 (15.3)	20.3 (13.6)	10.2 (16.3)
Colonial National Historical Park	100 (0)	6.7 (13.4)	21.1 (19.4)	99.3 (1.2)	84.4 (24.3)	2.1 (2)
Colorado National Monument	51.7 (31.1)	27.5 (53.2)	96 (9.7)	75.2 (28)	66.7 (70.4)	94.9 (14.5)
Congaree National Park	98.2 (4.3)	5.4 (10.6)	14.1 (14.7)	67.5 (38)	87.7 (25.5)	56.5 (23.9)
Coronado National Memorial	97 (5.4)	4.6 (10.6)	99.4 (1.9)	15.8 (42.3)	24.4 (48.8)	1.1 (0.2)
Cowpens National Battlefield	98.1 (3.2)	12.1 (21.2)	20.9 (23.1)	83.6 (16.5)	94 (3.9)	22.8 (35.3)
Crater Lake National Park	100 (0)	17.6 (29.7)	99.4 (1.9)	98.7 (3.9)	45.4 (25.6)	1.4 (1.2)
Craters Of The Moon National Monument and Preserve	97.4 (1.7)	48.7 (72.2)	50.6 (32.6)	87.1 (16.3)	19.8 (22.1)	14.7 (9.8)
Cumberland Gap National Historical Park	99.2 (1.2)	18.6 (25.7)	47 (47.8)	82.3 (13.5)	91.8 (1.5)	1.4 (0.9)
Cumberland Island National Seashore	27.8 (5.5)	21.2 (49.1)	48.9 (24.6)	50.1 (38.6)	56.4 (5.3)	57.8 (38.6)
Curecanti National Recreation Area	51.4 (34)	31.2 (69.7)	97.3 (6.8)	66.6 (37.7)	79.7 (34.2)	47.9 (54.6)
Cuyahoga Valley National Park	99.3 (2.2)	9.8 (10.2)	90.6 (17.2)	83.6 (3)	88.9 (13.9)	2.4 (2)

Park	Cld1	Cld4	Vap18	Wet12	Wet18	Frs12
Death Valley National Park	100 (0)	25.9 (46.4)	100 (0)	48.9 (26.2)	50.6 (22.1)	1.1 (0.2)
Delaware Water Gap National Recreation Area	100 (0)	3.3 (6.7)	66.6 (37.5)	99 (2.2)	99 (2.2)	1.1 (0.2)
Denali National Park and Preserve	55.3 (15.2)	6.4 (11.2)	100 (0)	43.5 (21.9)	66.9 (30.9)	1.4 (0.9)
Devils Postpile National Monument	99.6 (1.2)	15.1 (18.5)	100 (0)	48.2 (24.6)	44.9 (12.7)	1.4 (0.9)
Devils Tower National Monument	41.4 (45)	51.1 (73)	82 (38.1)	10.7 (12.7)	48 (42.1)	80.3 (22.2)
Dinosaur National Monument	64.3 (3.5)	29.6 (54.7)	93.7 (16.5)	62.3 (48.2)	51.6 (70.5)	53.2 (54.5)
Dry Tortugas National Park	93.2 (9.3)	96.1 (11.7)	97.1 (1.7)	94.8 (15.5)	97.7 (5.8)	NA (NA)
Ebey's Landing National Historical Reserve	98.9 (2.2)	13.9 (31.6)	13.7 (29.2)	11.2 (15.6)	11.1 (19.2)	15 (11)
Effigy Mounds National Monument	99.2 (1.2)	15.6 (20.7)	94.5 (16.5)	91.5 (18.3)	97.4 (3.7)	1.4 (0.9)
Eisenhower National Historic Site	100 (0)	12.8 (27.9)	26.5 (20.4)	97.9 (1.7)	92.8 (10.1)	28.4 (41.6)
El Malpais National Monument	97.5 (1.3)	5 (11.5)	98.4 (4.9)	39.8 (32.3)	36.4 (45.2)	3 (5.7)
El Morro National Monument	96.7 (0.7)	4.6 (10.6)	97.3 (5.8)	39.7 (31.1)	34.3 (26.6)	3.3 (6.7)
Eugene O'Neill National Historic Site	99.6 (1.2)	13.8 (18)	99.2 (1.2)	74.3 (35.8)	27.7 (16.6)	12.1 (20.1)
Everglades National Park	78 (25.4)	96.1 (11.7)	96.1 (0.7)	94.8 (15.5)	98.1 (5.8)	27.8 (54.9)
Fire Island National Seashore	100 (0)	5.1 (9.6)	47.1 (23.1)	99.3 (2.2)	98.9 (3.2)	1.1 (0.2)
Florissant Fossil Beds National Monument	83.6 (15.4)	27.6 (59)	97 (7.8)	66.5 (25.1)	84.8 (24.8)	49.5 (18.3)
Fort Bowie National Historic Site	94.5 (13.3)	4.3 (9.6)	99.7 (1)	19 (49.7)	25.8 (61.2)	1.1 (0.2)
Fort Caroline National Memorial	7.1 (5.9)	34.1 (58.8)	59.5 (20.4)	53.7 (38.6)	60.8 (7.1)	55.8 (43.2)
Fort Davis National Historic Site	27.2 (19.4)	52.6 (47)	100 (0)	58 (18.9)	71.1 (22.9)	3.7 (7.7)
Fort Donelson National Battlefield	98.9 (2.2)	16.9 (21.8)	44.2 (51.6)	81.3 (25.6)	70.8 (26.9)	10.2 (11.3)
Fort Frederica National Monument	38.5 (2.9)	16.2 (40.6)	37.1 (16.8)	51.6 (38.8)	56.2 (6.8)	58.1 (39.8)
Fort Laramie National Historic Site	77.2 (11)	56.6 (60.4)	93.8 (17.5)	57.4 (22.8)	73.6 (24.4)	3.7 (2.9)
Fort Larned National Historic Site	92.5 (1.8)	15.7 (32.1)	94.5 (16.5)	82.6 (34.2)	97 (5.8)	5.7 (12.8)
Fort Matanzas National Monument	3 (5.7)	80.1 (15.3)	70.1 (25.4)	64.3 (44.8)	75.4 (7.5)	57.2 (52.9)
Fort Moultrie National Monument	98.2 (0.9)	6.4 (13.5)	14.8 (12.7)	57 (34.9)	83.5 (29.8)	46.7 (18.2)
Fort Necessity National Battlefield	100 (0)	5.8 (11.5)	30.2 (22)	97.4 (6.5)	95.6 (4.5)	1.1 (0.2)
Fort Point National Historic Site	99.2 (1.2)	14.4 (18.9)	98.9 (0.2)	78.2 (35.5)	36.7 (25.7)	5.4 (10.4)
Fort Pulaski National Monument	82.1 (9.1)	14.2 (36.8)	23.9 (16)	57.3 (41)	73.6 (26.6)	52 (28.7)
Fort Raleigh National Historic Site	100 (0)	7.5 (12.3)	15.5 (22.2)	100 (0)	79 (33.4)	12.4 (24)
Fort Sumter National Monument	97.9 (1.7)	6.4 (13.5)	14.8 (12.7)	57 (34.9)	83.5 (29.8)	44.5 (18.7)
Fort Union National Monument	93.3 (1.7)	36.3 (66.6)	97.7 (6.8)	29.1 (61.4)	44.3 (77.1)	2.1 (2.8)
Fort Union Trading Post National Historic Site	85.2 (21.7)	3.7 (6.6)	96 (10.7)	13.5 (15.6)	51.2 (49.3)	57.6 (34.8)
Fort Vancouver National Historic Site	100 (0)	18.7 (42.3)	85.9 (6.6)	96.2 (7.8)	55.5 (10.6)	8.1 (14.3)
Fort Washington Park	100 (0)	7.1 (15.4)	20.7 (27.7)	96.1 (0.7)	70.1 (55.4)	1.4 (1.2)
Fossil Butte National Monument	84.9 (13.2)	36.6 (58.4)	78.8 (38.2)	81.3 (24.4)	1.4 (0.9)	2.4 (3.8)
Frederick Douglass National Historic Site	100 (0)	7.4 (16.4)	20.7 (27.7)	96.7 (0.7)	71.3 (51.8)	1.4 (1.2)
Fredericksburg and Spotsylvania National Military Park	100 (0)	7.1 (15.4)	25.6 (15.1)	97.5 (3.9)	72.4 (49.4)	1.4 (1.2)
Friendship Hill National Historic Site	100 (0)	6.8 (13.5)	29.1 (20.6)	100 (0)	97.1 (1.7)	1.4 (0.9)
Gates Of The Arctic National Park and Preserve	49.5 (20.4)	3.8 (4.6)	100 (0)	47.6 (27.5)	72.1 (31.3)	1.1 (0.2)
Gateway National Recreation Area	100 (0)	4.3 (9.6)	27.2 (19.1)	99.3 (2.2)	99.3 (2.2)	1.1 (0.2)

Park	Cld1	Cld4	Vap18	Wet12	Wet18	Frs12
Gauley River National Recreation Area	99.2 (1.2)	14.9 (16.6)	8.5 (15.2)	98.6 (2.2)	96.4 (4.5)	1.4 (1.2)
George Washington Birthplace National Monument	100 (0)	7.7 (17.4)	27.1 (16.3)	96.7 (2.9)	70.7 (57.3)	1.1 (0.2)
George Washington Carver National Monument	98.2 (1.4)	12.4 (15.3)	98.7 (3.9)	73.3 (25.4)	71.2 (12.2)	4.3 (8.5)
George Washington Memorial Parkway	100 (0)	8 (18.3)	20.3 (26.5)	96.4 (0.7)	72 (50.6)	2.2 (3.3)
Gettysburg National Military Park	100 (0)	12.8 (27.9)	26.9 (20.4)	98.3 (2.9)	94 (6.5)	25.4 (36.2)
Gila Cliff Dwellings National Monument	98.5 (2.4)	5.6 (13.5)	99.4 (1.9)	37.7 (52.7)	43.6 (75.4)	1.1 (0.2)
Glacier Bay National Park and Preserve	66.8 (10.6)	31.5 (26.2)	91 (8.5)	84.2 (34.5)	100 (0)	10.4 (2.2)
Glacier National Park	78.4 (11.4)	24 (20)	22.6 (19.4)	38.1 (33.8)	53.2 (54)	67 (17.4)
Glen Canyon National Recreation Area	43 (38.8)	57.7 (40.6)	98.4 (4.9)	52.8 (18.3)	44.4 (22.3)	16.8 (20.9)
Golden Gate National Recreation Area	99.6 (1.2)	14.7 (19.9)	98.9 (0.2)	78.6 (36.5)	20.2 (26)	5.8 (9.2)
Golden Spike National Historic Site	99.3 (1.2)	62.9 (55.6)	92.2 (17.5)	94.3 (5.6)	10.1 (9.1)	8.5 (13.1)
Grand Canyon National Park	92.8 (11.2)	33.8 (47.6)	99.4 (1.9)	45.5 (26.8)	45.9 (17.7)	3.1 (4.6)
Grand Portage National Monument	100 (0)	4.6 (10.6)	97.1 (8.7)	24 (10.5)	41.6 (31.6)	1.7 (1.8)
Grand Teton National Park	98.5 (3.6)	16.8 (32.1)	69.7 (43.8)	65.4 (5.5)	21.3 (35.6)	26 (45.4)
Grant-Kohrs Ranch National Historic Site	98.9 (2.4)	40.7 (59.3)	21.3 (34.4)	35.8 (7.7)	33.9 (46.4)	1.1 (0.2)
Great Basin National Park	85.4 (27.9)	78.7 (43.4)	100 (0)	72.9 (26.4)	74.1 (31.7)	16.4 (8.8)
Great Sand Dunes National Park and Preserve	71.7 (30.3)	33.2 (72.4)	97.3 (6.8)	53.6 (44.4)	71.4 (53.2)	11.2 (29)
Great Smoky Mountains National Park	99.2 (1.2)	13.1 (20)	48.1 (38.5)	84.2 (15.7)	93.7 (4.2)	2.1 (1.7)
Greenbelt Park	100 (0)	7.7 (17.4)	21.4 (26.7)	97.1 (1.7)	72.7 (50.3)	2.2 (3.3)
Guadalupe Mountains National Park	48.5 (8.9)	32.5 (72.4)	99.7 (1)	40 (44.9)	75 (33.8)	1.1 (0.2)
Guilford Courthouse National Military Park	98.9 (2.2)	8.6 (13.3)	1.9 (2.6)	88.7 (13.4)	95.8 (3.4)	1.8 (2.3)
Gulf Islands National Seashore	99.2 (1.2)	19.3 (29.8)	63.3 (22.8)	16.7 (35.2)	54.6 (48.1)	54.8 (20.9)
Hagerman Fossil Beds National Monument	96.7 (2.9)	35.6 (47.2)	20 (2.1)	86.6 (23.3)	5 (11)	1.1 (0.2)
Haleakala National Park	100 (0)	1.1 (0.2)	78.9 (12.5)	8.3 (7.1)	12.5 (8.4)	NA (NA)
Harpers Ferry National Historical Park	100 (0)	7.1 (15.4)	22.4 (23.8)	99.7 (1)	73.5 (40.2)	8.6 (21.4)
Hawaii Volcanoes National Park	100 (0)	2.2 (0.5)	79.2 (8.4)	38.2 (24.9)	31.8 (26.9)	42.4 (18.4)
Herbert Hoover National Historic Site	98.9 (0.2)	19.7 (27.3)	91.9 (24.3)	98.5 (2.4)	97.4 (3.7)	1.7 (1.8)
Homestead National Monument of America	96.7 (0.7)	15.9 (30.7)	93.4 (18.4)	97.2 (0.8)	97.8 (0.5)	29.1 (22.8)
Hopewell Culture National Historical Park	99.2 (1.2)	22 (25.2)	7 (13)	98.5 (3.2)	91.7 (12.5)	3.4 (6.2)
Hopewell Furnace National Historic Site	100 (0)	5.3 (12.5)	78 (35.7)	99 (2.2)	96.8 (8.6)	1.1 (0.2)
Horseshoe Bend National Military Park	99.3 (2.2)	6.2 (11.5)	42.2 (10.3)	37.7 (42.8)	19 (31.5)	40.8 (3)
Hot Springs National Park	98.9 (2.2)	12.8 (23.9)	99.3 (1.2)	4.8 (8.6)	9.2 (24.2)	1.7 (1.8)
Hovenweep National Monument	34.9 (54.1)	26.3 (55.4)	98.4 (4.9)	51.5 (27.4)	51.7 (51.4)	42.2 (62.2)
Hubbell Trading Post National Historic Site	85.3 (13.2)	6 (13.5)	96.9 (5.8)	38.8 (17)	38.2 (15.7)	9 (7.8)
Indiana Dunes National Lakeshore	99.2 (1.2)	12.2 (21.3)	62.3 (83)	75.2 (15.6)	73.4 (21.3)	1.4 (0.9)
Isle Royale National Park	100 (0)	4.6 (10.6)	97.1 (8.7)	29.9 (6)	49 (26.3)	1.7 (1.8)
Jean Lafitte National Historical Park and Preserve	98.9 (2.2)	13.9 (24.1)	100 (0)	23.2 (48.4)	58.3 (67.2)	9.9 (12.3)
Jewel Cave National Monument	70.8 (27.6)	51.1 (64.8)	85.1 (34.5)	45.7 (34.7)	50.9 (17.7)	76.5 (23.4)
John Day Fossil Beds National Monument	99.3 (1.2)	21.7 (41.5)	62.2 (14.5)	96.6 (7.8)	19.3 (19.2)	11.6 (15.6)
John Muir National Historic Site	99.6 (1.2)	14.1 (19.1)	99.2 (1.2)	75.6 (35.8)	24.4 (19.8)	8.4 (18.3)

Park	Cld1	Cld4	Vap18	Wet12	Wet18	Frs12
Johnstown Flood National Memorial	100 (0)	5.7 (12.5)	43.8 (50.7)	84.5 (7.4)	89.7 (6.5)	2.2 (3.3)
Joshua Tree National Park	100 (0)	4.6 (10.6)	100 (0)	46.9 (26.5)	50.7 (26)	1.1 (0.2)
Kalaupapa National Historical Park	100 (0)	1.1 (0.2)	78.5 (10.3)	2.1 (1.7)	2.1 (2.8)	NA (NA)
Kaloko-Honokohau National Historical Park	100 (0)	1.1 (0.2)	78.5 (9.3)	22.3 (13.1)	13.4 (13.7)	42.4 (18.4)
Katmai National Park and Preserve	72.9 (6.6)	42.3 (40.3)	93.4 (4.6)	75.9 (47.4)	94.1 (6.5)	9.3 (3.2)
Kenai Fjords National Park	87 (17.8)	37 (41.1)	93.1 (2.6)	59 (24.2)	79.8 (43.4)	9.4 (8.2)
Kennesaw Mountain National Battlefield Park	99.3 (2.2)	7.2 (13.5)	46.7 (27.2)	52.4 (31.8)	18.2 (33.9)	11.2 (17.2)
Kings Mountain National Military Park	98.1 (3.2)	9.4 (15.3)	13.2 (19.5)	81.6 (21.4)	92.7 (6.1)	49.9 (28.9)
Klondike Gold Rush National Historical Park	65.5 (14.3)	47.1 (17.7)	96.1 (10.7)	78.2 (45.3)	100 (0)	15.7 (13.2)
Knife River Indian Villages National Historic Site	87.6 (15.6)	2.7 (3.6)	95.8 (12.6)	98.6 (2.9)	98.9 (0.2)	2.1 (2.8)
Kobuk Valley National Park	55.5 (39.5)	2.8 (3.8)	94.9 (7.8)	56.6 (26.6)	80.3 (4.2)	1.4 (0.9)
Lake Clark National Park and Preserve	91 (1.9)	29.1 (49.1)	97 (3.8)	73.9 (51.9)	84.6 (29.9)	7.1 (1.3)
Lake Mead National Recreation Area	97.1 (1.7)	38.1 (57.6)	99.7 (1)	47.2 (29.6)	45.8 (23.5)	1.1 (0.2)
Lake Meredith National Recreation Area	72.6 (9)	22.9 (39.9)	97.1 (8.7)	31.1 (57.5)	67.3 (22.7)	12.3 (13.2)
Lake Roosevelt National Recreation Area	96.1 (6.3)	4.8 (6.6)	38.5 (5.9)	80.8 (20.3)	52.4 (32.8)	3.9 (7.4)
Lassen Volcanic National Park	99.6 (1.2)	27.8 (23.9)	99.6 (1.1)	98.3 (3.9)	42.5 (36.5)	1.7 (1.8)
Lava Beds National Monument	99.6 (1.2)	26.7 (24.1)	99.7 (1)	99.7 (1)	40.3 (33.3)	1.4 (0.9)
Lewis and Clark National Historical Park	95.2 (14.5)	22.1 (53.3)	93.9 (12.5)	98.7 (3.9)	60.6 (9.5)	10.8 (18.9)
Lincoln Boyhood National Memorial	99.6 (1.1)	15.3 (20.1)	41.5 (70.1)	80.8 (27.3)	80.3 (25.9)	1.4 (0.9)
Little Bighorn Battlefield National Monument	55.2 (49.5)	49.2 (47.2)	62.5 (44.3)	4.4 (7.5)	18.9 (31.5)	13.9 (16.4)
Little River Canyon National Preserve	99.3 (2.2)	8.6 (15.3)	42.7 (18.6)	47.2 (36.2)	37.6 (26.8)	35.4 (16.9)
Lyndon B. Johnson National Historical Park	69.1 (22)	18.8 (38.3)	81 (54.2)	60 (13.1)	70.6 (20.4)	28.2 (4.6)
Mammoth Cave National Park	99.6 (1.1)	17.9 (23.7)	43.1 (51.4)	69.6 (20.7)	64 (21)	1.4 (0.9)
Manassas National Battlefield Park	100 (0)	6.1 (12.5)	19.9 (26.5)	97.9 (3.9)	73.2 (48.2)	3.6 (6.3)
Manzanar National Historic Site	100 (0)	13.7 (18.5)	100 (0)	49 (24.2)	50.7 (17.1)	1.7 (1.8)
Marsh-Billings-Rockefeller National Historical Park	98.5 (3.6)	3 (5.7)	92.7 (7.1)	98.6 (1.2)	99.2 (1.2)	1.1 (0.2)
Mary McLeod Bethune Council House National Historic Site	100 (0)	8 (18.3)	21.1 (27.7)	96.7 (0.7)	72 (51.5)	2.2 (3.3)
Mesa Verde National Park	42.1 (44.9)	20.9 (46.6)	98.7 (3.9)	52.7 (29.6)	57.3 (59.8)	17.5 (20.9)
Minute Man National Historical Park	100 (0)	13.8 (31.5)	92.3 (16.5)	97.3 (3.6)	99.2 (1.2)	1.1 (0.2)
Mississippi National River and Recreation Area	99.2 (1.2)	7.5 (10.6)	92.5 (19.4)	88.5 (23.7)	98.5 (1.4)	1.1 (0.2)
Missouri National Recreational River	98.9 (0.2)	11.9 (23.1)	95.4 (12.6)	99.2 (1.2)	98.5 (1.4)	1.4 (0.9)
Mojave National Preserve	99.6 (1.2)	19.7 (39.3)	100 (0)	48.2 (28.4)	44.9 (26)	1.1 (0.2)
Monocacy National Battlefield	100 (0)	8.4 (19.3)	21.4 (26.7)	99 (2.9)	79 (35.6)	16.8 (37.7)
Montezuma Castle National Monument	94.5 (2.9)	7.3 (17.4)	99 (2.9)	40.7 (21.2)	39.8 (13.2)	3.8 (5.7)
Moores Creek National Battlefield	100 (0)	5.4 (11.5)	7 (6.8)	72.2 (33.3)	88.5 (15.1)	36.4 (9.5)
Morristown National Historical Park	100 (0)	3.7 (7.7)	43.8 (23.4)	99.3 (2.2)	99 (2.2)	1.1 (0.2)
Mount Rainier National Park	100 (0)	16 (35.1)	13.6 (24.2)	98.1 (5.8)	63.7 (13)	7.6 (11.7)
Mount Rushmore National Memorial	88.2 (8.1)	45.9 (58.4)	85.4 (33.5)	50.9 (39)	58.1 (14.7)	81.3 (18)
Muir Woods National Monument	99.6 (1.2)	14.4 (18.9)	98.9 (0.2)	78.2 (35.5)	24.8 (27.3)	5.4 (10.4)
Natchez Trace Parkway	98.9 (2.2)	10.6 (20.2)	57.5 (27.2)	25.1 (31)	48.1 (18.3)	31.5 (9.2)

Park	Cld1	Cld4	Vap18	Wet12	Wet18	Frs12
National Capital Parks-East	100 (0)	8 (18.3)	21.1 (27.7)	96.7 (0.7)	72.3 (51.5)	2.2 (3.3)
National Park of American Samoa	100 (0)	98.9 (0.2)	96.3 (5.6)	24.9 (46.2)	39.9 (5)	NA (NA)
Natural Bridges National Monument	25.2 (51.8)	46.8 (65)	98.1 (5.8)	52.2 (23.1)	47.4 (42.2)	39.2 (56.4)
Navajo National Monument	61.5 (37.2)	26.9 (47.7)	99 (2.9)	44.9 (20.8)	39.3 (17.6)	13.6 (21.6)
New River Gorge National River	99.2 (1.2)	17.2 (22.5)	3 (5.1)	98.6 (2.2)	96.4 (4.5)	1.4 (1.2)
Nez Perce National Historical Park	99.6 (1.2)	6.9 (9.4)	12 (10.4)	83 (16.7)	26.9 (40.4)	4.3 (6.7)
Ninety Six National Historic Site	98.2 (4.3)	6.5 (12.5)	22.9 (18.5)	75 (26.7)	85.3 (29.6)	37.3 (11.8)
Niobrara National Scenic River	98.9 (0.2)	12.6 (19.2)	94.1 (14.5)	99.2 (1.2)	98.9 (0.2)	1.1 (0.2)
Noatak National Preserve	55.3 (33.2)	3.3 (3.7)	96.2 (7.5)	57 (29.4)	80.3 (10.4)	1.4 (0.9)
North Cascades National Park	98.2 (4.3)	9.7 (17.1)	14.1 (25.8)	66.1 (34.4)	51.5 (26)	13.9 (12.2)
Obed Wild and Scenic River	100 (0)	18.2 (23.6)	59.3 (27.8)	80.2 (15.4)	87 (9.8)	1.7 (1.8)
Ocmulgee National Monument	77.5 (17.3)	5.4 (10.6)	41.7 (16.3)	46.8 (44.7)	20.3 (28.9)	76.1 (18.2)
Olympic National Park	90.9 (22.9)	21 (44.5)	53.5 (11.5)	95.8 (12.6)	46.4 (23.7)	8.7 (7.8)
Oregon Caves National Monument	100 (0)	25 (23.2)	98 (3.9)	98.7 (3.9)	55.8 (6.6)	9.9 (7.1)
Organ Pipe Cactus National Monument	98.5 (2.4)	4.3 (9.6)	95.7 (10.8)	40.8 (28.6)	34.8 (31)	6 (6.5)
Ozark National Scenic Riverways	98.2 (1.4)	21.6 (30)	82.7 (25.4)	88 (18.7)	83.7 (25)	1.8 (0.9)
Padre Island National Seashore	38.6 (47.9)	60.6 (42.2)	94.8 (15.7)	68.9 (17.5)	80.8 (16)	14.7 (40)
Palo Alto Battlefield National Historical Park	42.5 (45.6)	73.7 (35.8)	95.6 (13.3)	78.8 (23.6)	85 (13.7)	13.5 (36.3)
Pea Ridge National Military Park	98.2 (1.4)	13.7 (17)	99.7 (1)	48.9 (32.1)	52.3 (29.1)	2.1 (2)
Pecos National Historical Park	96.3 (2.9)	23.9 (53.8)	99 (2.9)	27.6 (61.4)	36.6 (70.1)	2.4 (3.8)
Petersburg National Battlefield	100 (0)	7.8 (14.4)	25.1 (9.7)	96.3 (8.7)	90.7 (19.7)	1.4 (1.2)
Petrified Forest National Park	94.9 (4)	4.3 (9.6)	96.9 (5.8)	38 (14.6)	36.4 (15.5)	6.7 (3.9)
Petroglyph National Monument	97.8 (0.5)	15.8 (41.6)	99.4 (1.9)	36.3 (53.4)	40.2 (72.8)	2.1 (2.8)
Pictured Rocks National Lakeshore	100 (0)	2.1 (1.8)	96.1 (11.7)	45.8 (11.8)	59.1 (4.2)	1.7 (1.8)
Pinnacles National Monument	99.6 (1.2)	9.9 (14.3)	100 (0)	64.9 (39.2)	29.8 (42.6)	7 (8.4)
Pipe Spring National Monument	86.2 (14.7)	47.3 (40.5)	98.7 (3.9)	48.7 (29.5)	49 (15.6)	4.9 (3.2)
Pipestone National Monument	98.6 (1.2)	9.8 (16.4)	96.1 (11.7)	99.6 (1.2)	98.5 (1.4)	1.1 (0.2)
Piscataway Park	100 (0)	7.1 (15.4)	21.4 (26.7)	96.1 (0.7)	69.7 (56.6)	1.4 (1.2)
Point Reyes National Seashore	99.6 (1.2)	15.4 (19.9)	98.9 (0.2)	82.7 (32.5)	9.3 (10.9)	6.1 (9.2)
Port Chicago Naval Magazine National Memorial	99.6 (1.2)	14.5 (20.2)	100 (0)	76.7 (33.4)	15.8 (13.7)	11.1 (17.2)
Presidio of San Francisco	99.2 (1.2)	14.4 (18.9)	98.9 (0.2)	78.2 (35.5)	36.7 (25.7)	5.4 (10.4)
Prince William Forest Park	100 (0)	6.7 (14.5)	23.3 (20.9)	96.4 (0.7)	70.8 (54.2)	1.4 (1.2)
Pu'uhonua o Honaunau National Historical Park	100 (0)	2.2 (0.5)	77.3 (7.9)	30.3 (24.7)	23.9 (27.9)	42.4 (18.4)
Puukohola Heiau National Historic Site	100 (0)	2.2 (0.5)	76.7 (8.9)	22.3 (13.1)	19 (21.4)	42.4 (18.4)
Rainbow Bridge National Monument	62.1 (33.9)	59.2 (22.3)	98.4 (4.9)	49.2 (23.4)	44.2 (16.6)	11.5 (18.9)
Redwood National Park	100 (0)	27.4 (24.9)	97.7 (5.8)	98.7 (3.9)	63.1 (9.5)	83.5 (11.2)
Richmond National Battlefield Park	100 (0)	7.5 (13.4)	27.6 (10.2)	96.5 (6.8)	85.9 (22.2)	1.4 (1.2)
Rio Grande Wild and Scenic River	23.6 (15.2)	78.8 (20.2)	91.1 (18.1)	60.6 (11.2)	64.7 (14.3)	6.8 (11.2)
Rock Creek Park	100 (0)	8 (18.3)	21.1 (27.7)	96.7 (0.7)	72.3 (51.5)	2.2 (3.3)
Rocky Mountain National Park	92.2 (3.7)	27.7 (58.1)	94.4 (15.5)	67.9 (27.5)	86.8 (22.8)	10.4 (11)

Park	Cld1	Cld4	Vap18	Wet12	Wet18	Frs12
Roosevelt-Vanderbilt Headquarters	100 (0)	2.7 (4.7)	54.5 (21.4)	99.3 (2.2)	99.6 (1.1)	1.1 (0.2)
Russell Cave National Monument	99.3 (2.2)	12.5 (20.1)	44 (30.2)	51.7 (41.9)	44.1 (10.8)	27 (23.3)
Sagamore Hill National Historic Site	100 (0)	4 (8.6)	37.1 (25.6)	99.3 (2.2)	99.3 (2.2)	1.7 (1.8)
Saguaro National Park	98.8 (2.4)	4.6 (10.6)	98.1 (5.8)	21.4 (59.2)	11.6 (25.6)	2.2 (2.6)
Saint Croix National Scenic Riverway	99.6 (1.1)	4.7 (9.6)	96.1 (11.7)	60 (48.6)	80.5 (7)	1.1 (0.2)
Saint-Gaudens National Historic Site	100 (0)	3 (5.7)	93.4 (6.1)	98.6 (1.2)	99.2 (1.2)	1.1 (0.2)
Salinas Pueblo Missions National Monument	87.2 (17.6)	18.1 (33.6)	99.4 (1.9)	31.5 (59.5)	40.3 (75.4)	2.1 (2.8)
San Antonio Missions National Historical Park	29.4 (12.6)	46.4 (52.1)	83.1 (50.6)	60.3 (12.1)	72.6 (22.1)	1.4 (1.2)
San Juan Island National Historical Park	93.6 (8.7)	17.8 (37.3)	21.9 (31.3)	29.7 (43.6)	20.2 (6.9)	13.6 (10)
Sand Creek Massacre National Historic Site	87 (10)	22.3 (47.4)	96.8 (9.7)	58.1 (40.4)	88.1 (18)	7 (7.8)
Santa Monica Mountains National Recreation Area	100 (0)	4.3 (9.6)	100 (0)	48.5 (17.6)	56.6 (11.7)	15.5 (13)
Saratoga National Historical Park	100 (0)	2.1 (2.8)	96.4 (7.6)	98.2 (4.3)	99.6 (1.2)	1.1 (0.2)
Saugus Iron Works National Historic Site	100 (0)	13.5 (31.5)	95.7 (9.7)	97.9 (1.7)	99.2 (1.2)	1.1 (0.2)
Scotts Bluff National Monument	97 (2.9)	36.5 (63.6)	94.1 (16.5)	71.5 (21.6)	91.8 (10)	2.7 (5.1)
Sequoia and Kings Canyon National Parks	100 (0)	13.3 (19.7)	100 (0)	48.6 (25.3)	49.7 (17.2)	1.7 (1.8)
Shenandoah National Park	100 (0)	5.7 (12.5)	19.2 (24.3)	99 (2.9)	88.4 (14.9)	3.1 (3.1)
Shiloh National Military Park	98.9 (2.2)	14.8 (24.8)	48.2 (37.7)	38.1 (23.9)	48.6 (9.2)	3.1 (3.1)
Sitka National Historical Park	63.3 (6.4)	18.1 (22.9)	91.7 (9.7)	96.8 (3.4)	98.3 (3.9)	13.9 (12.9)
Sleeping Bear Dunes National Lakeshore	99.6 (1.2)	1.7 (1.8)	95.5 (13.6)	53 (21.1)	61.6 (10.1)	1.7 (1.8)
Stones River National Battlefield	99.2 (1.2)	16.5 (21.8)	46.3 (37.6)	59.3 (42)	60.4 (35.9)	31.4 (26.6)
Sunset Crater Volcano National Monument	88.6 (7.7)	8 (19.3)	97.3 (6.8)	40.8 (18.2)	36.9 (4.1)	7.1 (3.4)
Tallgrass Prairie National Preserve	97.8 (0.5)	16.2 (28)	95.1 (14.6)	95.5 (3.6)	89.5 (17.7)	1.4 (0.9)
Theodore Roosevelt National Park	84 (25.3)	4.4 (6.6)	95.4 (12.6)	15.7 (19)	65.9 (49.4)	60.4 (34.8)
Thomas Stone National Historic Site	100 (0)	7.7 (17.4)	22.4 (23.8)	95.7 (0.9)	69.7 (56.6)	1.4 (1.2)
Timpanogos Cave National Monument	99.3 (1.2)	70.4 (54.9)	94.6 (12.6)	93.3 (8.5)	5.2 (6.6)	3.7 (7.7)
Timucuan Ecological and Historic Preserve	11.1 (6.3)	30.8 (61.7)	56.3 (22.1)	51.7 (42.5)	60.1 (9.2)	59.2 (38.4)
Tonto National Monument	96.7 (2.9)	4.3 (9.6)	90.7 (24.1)	37.3 (14.2)	32.5 (4.8)	4.6 (4.6)
Tumacacori National Historical Park	99.2 (2.4)	4.6 (10.6)	94.4 (10.8)	14.6 (38.7)	2.5 (2.5)	1.1 (0.2)
Tuzigoot National Monument	94.5 (2.9)	9.3 (23.2)	98.7 (2.9)	41.4 (21.1)	40.4 (15.1)	2.7 (4.7)
Upper Delaware National Scenic and Recreational River	100 (0)	2.4 (3.8)	77.5 (33.9)	96.1 (9.7)	99 (2.2)	1.1 (0.2)
Valley Forge National Historical Park	100 (0)	5 (11.5)	33.9 (25.4)	99.3 (1.1)	96.1 (10.8)	1.1 (0.2)
Valor in the Pacific National Monument	100 (0)	25 (21.2)	90.9 (8)	10.2 (14.3)	1.4 (0.9)	1.1 (0.2)
Vicksburg National Military Park	99.6 (1.1)	14.8 (25.9)	88.7 (28.9)	3.4 (5.7)	21.9 (55.2)	4.7 (7.5)
Virgin Islands National Park	63.9 (62.6)	100 (0)	100 (0)	99.3 (1.1)	80.8 (36.6)	NA (NA)
Voyageurs National Park	83.9 (16.2)	1.4 (0.9)	96.8 (9.7)	70.9 (19.2)	70.5 (8.2)	4.1 (3.1)
Walnut Canyon National Monument	91.8 (1.5)	7.6 (18.3)	97 (5.7)	40.4 (18.2)	36.9 (4.1)	6.4 (3.5)
War In The Pacific National Historical Park	1.1 (0.2)	18.8 (48.3)	100 (0)	73 (33.9)	87.6 (37.3)	NA (NA)
Washita Battlefield National Historic Site	53.7 (27.2)	18.2 (30.9)	97.7 (6.8)	41.2 (59.9)	81.8 (24.1)	8.3 (14.3)
Weir Farm National Historic Site	100 (0)	3.7 (7.7)	47 (20.2)	99.3 (2.2)	99.3 (2.2)	1.1 (0.2)
Whiskeytown National Recreation Area	100 (0)	24.1 (16.4)	99.3 (1.1)	98.7 (3.9)	52.5 (13.4)	15.1 (28.7)

Park	Cld1	Cld4	Vap18	Wet12	Wet18	Frs12
White Sands National Monument	64.6 (17.2)	25.9 (44.8)	99.7 (1)	34.6 (54.6)	61.6 (59.3)	1.1 (0.2)
Whitman Mission National Historic Site	99.6 (1.2)	6.6 (9.5)	36 (9.6)	96.6 (7.8)	32.5 (35.8)	19.4 (28.9)
Wilson's Creek National Battlefield	98.2 (1.4)	19.3 (24.2)	97.4 (7.8)	77.7 (14.7)	77.6 (11.1)	3.6 (6.3)
Wind Cave National Park	91.6 (3.9)	47 (58.2)	86.9 (33.7)	53.9 (37.1)	59.1 (12.4)	71.8 (23.3)
Wolf Trap National Park for the Performing Arts	100 (0)	8 (18.3)	20.3 (26.5)	96.4 (0.7)	71.2 (53)	3.6 (7.6)
Wrangell - St Elias National Park and Preserve	74.5 (28)	22 (13.7)	89.5 (8)	38.3 (18.9)	64.9 (30.7)	1.8 (1.3)
Wright Brothers National Memorial	100 (0)	7.5 (12.3)	15.9 (23.4)	100 (0)	78.5 (34.4)	12.1 (23.1)
Wupatki National Monument	83.8 (8.9)	10.9 (28.1)	98.4 (4.9)	40.8 (18.2)	37.7 (6.6)	7.5 (2.3)
Yellowstone National Park	98.5 (3.6)	13.1 (25.8)	62.8 (51.6)	58.8 (2.4)	27 (44.3)	12.3 (7.8)
Yosemite National Park	99.6 (1.2)	15.8 (18.5)	100 (0)	53.7 (21.7)	29.4 (21)	1.1 (0.2)
Yucca House National Monument	39.8 (50.7)	22.3 (48.6)	98.7 (3.9)	50.8 (28.4)	52.8 (52.3)	30.9 (47.9)
Yukon-Charley Rivers National Preserve	58.8 (38.5)	5.7 (11.4)	97 (5.8)	29.2 (15.5)	18.5 (29)	1.1 (0.2)
Zion National Park	87.2 (17.6)	48.4 (50.9)	99.7 (1)	51.4 (27)	52.9 (17.2)	4.9 (4.4)
