# Supplementary material for: Climate Exposure of US National Parks in a New Era of Change
Source: PLoS One. 2014 Jul 2;9(7):e101302. doi: 10.1371/journal.pone.0101302 (PMC4079655; doi:10.1371/journal.pone.0101302)
Supplement: Appendix S5 — Mean percentile and maximum difference in percentile (in parentheses) for moving window standard deviations: annual mean temperature (Bio1), mean diurnal range (Bio2), isothermality (Bio3), temperature seasonality (Bio4), maximum temperature of the warmest month (Bio5), and minimum temperature of the coldest month (Bio6). (PDF) [file pone.0101302.s005.pdf]

Appendix S5. Mean percentile and maximum difference in percentile (in parentheses) for moving window standard deviations: annual mean temperature (Bio1), mean diurnal range (Bio2), isothermality (Bio3), temperature seasonality (Bio4), maximum temperature of the warmest month (Bio5), and minimum temperature of the coldest month (Bio6).

Park	Bio1	Bio2	Bio3	Bio4	Bio5	Bio6
Abraham Lincoln Birthplace National Historical Park	79.6 (15.1)	56.1 (23.1)	18.3 (35.2)	71 (35.2)	93.8 (12.3)	11.1 (10.2)
Acadia National Park	99.6 (1.1)	5.7 (14.1)	4.4 (8.5)	27.5 (24.1)	33.6 (30.4)	80.5 (43.1)
Agate Fossil Beds National Monument	84.7 (25.5)	82.7 (33.6)	14.6 (18.8)	23.8 (24)	94 (3.9)	4.3 (9.6)
Ala Kahakai National Historic Trail	74.6 (19.8)	79 (33.7)	38.4 (30.4)	69.5 (20.5)	86.4 (12.8)	57.7 (70.4)
Alagnak Wild River	71.1 (38.8)	12.4 (8.4)	63.5 (27)	44.7 (3.2)	99.6 (1.2)	80.2 (17.5)
Alibates Flint Quarries National Monument	70.1 (25.4)	49.5 (39.3)	12 (16.5)	85.1 (5.2)	96.3 (5.8)	6 (4.4)
Allegheny Portage Railroad National Historic Site	37.5 (11.5)	28.4 (53.4)	42.8 (13.9)	65.9 (18.6)	91.3 (2.9)	34.8 (21.2)
American Memorial Park	52.2 (37.1)	64.7 (31.9)	59.2 (13.7)	50 (25.3)	64.4 (36.6)	53.6 (7.3)
Amistad National Recreation Area	94.2 (2.4)	85.9 (23.1)	3.7 (4.4)	33.8 (13.1)	74.6 (38.3)	8.7 (14.8)
Anacostia Park	82.4 (28.9)	58 (48.5)	44.6 (13.9)	68.2 (34.1)	92 (16.5)	18.9 (15.6)
Aniakchak National Monument and Preserve	79.1 (32.5)	23.7 (40.9)	76.2 (23.5)	66.8 (28.8)	98.5 (2.4)	83.7 (8.8)
Antietam National Battlefield	62.9 (53.8)	42.4 (73.2)	50.4 (15.3)	54.4 (45)	47.1 (65.4)	14.2 (13.7)
Apostle Islands National Lakeshore	92.8 (2)	22.4 (59.2)	22.2 (56.8)	50.7 (48.6)	85 (34.4)	36.3 (19.9)
Appalachian National Scenic Trail	90.8 (2.2)	9.9 (17.3)	16.5 (10.3)	86.6 (12.7)	90.2 (16.5)	55.4 (24.9)
Appomattox Court House National Historical Park	78.6 (53.2)	100 (0)	21.1 (27.5)	61 (30.8)	99.4 (1.9)	21.4 (14.9)
Arches National Park	31.1 (32.6)	30.3 (38.9)	26 (21)	20.8 (29.1)	77.4 (40.1)	26 (25.8)
Arkansas Post National Memorial	79.6 (17.2)	59.2 (26.5)	9.1 (4)	50.2 (60.4)	70.7 (13.6)	18.7 (20.9)
Assateague Island National Seashore	97.3 (4.3)	73.7 (9.8)	27.2 (10.3)	60.6 (46.3)	99 (2.9)	19.8 (15.4)
Aztec Ruins National Monument	71.3 (30.3)	35.5 (66.8)	11.5 (18.9)	25 (36.8)	54.1 (38.5)	14 (30.4)
Badlands National Park	83.2 (29.9)	67.5 (21.5)	2.1 (2.8)	15.1 (21.9)	84.9 (11.5)	3.1 (3.6)
Baltimore-Washington National Parkway	85.3 (0.6)	72.4 (39.4)	49.9 (22.6)	70 (37.7)	99 (2.9)	18.5 (16.6)
Bandelier National Monument	46.9 (68.5)	57.8 (32)	44.7 (57.5)	43.8 (40.8)	72.7 (17.9)	21.3 (30.8)
Bent's Old Fort National Historic Site	55.1 (56)	39.6 (26.2)	12.6 (14.8)	40.5 (19.8)	78.7 (23.3)	13.8 (25.3)
Bering Land Bridge National Preserve	72 (18)	24.4 (7.7)	64.7 (28.1)	42.2 (49.4)	77.3 (34.4)	68 (33)
Big Bend National Park	86.9 (24.7)	94.5 (4.3)	4.7 (9.8)	30.8 (40.1)	93.9 (5.8)	5.6 (12.4)
Big Cypress National Preserve	45 (47.8)	14.2 (29.3)	30.4 (38.3)	78.2 (38.6)	59.8 (25.2)	68.4 (14.3)
Big Hole National Battlefield	49 (6.2)	16.3 (15.8)	30.9 (73.2)	9.4 (6.1)	84.3 (32.1)	4.9 (7.7)
Big South Fork National River and Recreation Area	52.2 (22.6)	77 (37.2)	39.9 (64)	61.1 (47.1)	86.6 (23.5)	8.4 (5.2)
Big Thicket National Preserve	48.8 (20.4)	32 (63.1)	34.7 (27.8)	54 (73.9)	55.9 (21.8)	17.2 (26.6)
Bighorn Canyon National Recreation Area	40.9 (19.2)	33 (20.6)	9 (15.9)	20.8 (10.7)	94.6 (9.7)	4.5 (5.4)
Biscayne National Park	64.2 (47)	3 (5.7)	31.9 (25.1)	76.4 (37.8)	31.8 (22.7)	76.4 (7.1)
Black Canyon Of The Gunnison National Park	24.9 (34.8)	85.6 (37.9)	40.8 (38.3)	29.3 (37.1)	76.4 (42.3)	46.8 (28.6)
Blue Ridge Parkway	71.1 (24.2)	71.7 (51.7)	21.3 (20.4)	74.2 (30.6)	95.3 (0.6)	24.2 (22.2)
Bluestone National Scenic River	51.9 (42.7)	27.6 (38)	25.6 (36.3)	61.6 (51)	94.3 (2.7)	20.5 (16.8)
Booker T. Washington National Monument	64.4 (26.2)	90.1 (22.6)	23.7 (23.9)	68.1 (35.9)	71.2 (32.9)	34.1 (32.8)
Boston Harbor Islands National Recreation Area	81.2 (25)	15.4 (38.8)	11.7 (27)	73 (27.7)	70.1 (46.6)	53 (29.9)

Park	Bio1	Bio2	Bio3	Bio4	Bio5	Bio6
Bryce Canyon National Park	43.3 (27)	71.7 (52.8)	54.5 (38.1)	2.8 (1.7)	48.6 (87.2)	18.7 (26.6)
Buck Island Reef National Monument	34.1 (25.5)	22.2 (34.5)	23.5 (22.1)	63.8 (33.2)	11.1 (11.7)	20 (12.3)
Buffalo National River	80.6 (20.5)	18 (39.1)	20.3 (35.4)	53.4 (48.7)	57.8 (46.1)	16.6 (32.3)
Cabrillo National Monument	27 (33.9)	46.8 (66.1)	83.3 (15)	80.4 (44.4)	86.7 (8.8)	26.3 (57)
Canaveral National Seashore	30.5 (26.9)	26 (55.9)	40.6 (60.1)	76.1 (45.8)	31.3 (21.4)	54.1 (24.1)
Canyon De Chelly National Monument	40 (34.8)	64.2 (58.1)	47.8 (36.2)	12.6 (12.7)	63.4 (69.5)	12 (29)
Canyonlands National Park	26.3 (36.7)	41.2 (76.7)	18.6 (22.6)	14 (20.2)	76.1 (44)	21.7 (30.1)
Cape Cod National Seashore	85.1 (33)	66 (46.4)	24.8 (20.9)	37.2 (11.3)	79.9 (39.8)	67.4 (49.2)
Cape Hatteras National Seashore	64.9 (29.3)	80.7 (21.2)	14.3 (25.1)	54.6 (47.1)	88.8 (30.1)	15.1 (29.2)
Cape Krusenstern National Monument	64.9 (11)	28.7 (18.4)	65.8 (23.7)	46.9 (49.8)	91 (11.5)	60.5 (56.3)
Cape Lookout National Seashore	59.2 (29.6)	85.4 (23.6)	9.9 (20.3)	54.9 (48.3)	80 (29.4)	18.8 (31.4)
Capitol Hill Parks	81.4 (30.9)	57 (49.2)	44.6 (13.9)	67.4 (33.9)	91.7 (17.5)	19.2 (14.5)
Capitol Reef National Park	47 (3)	12.4 (19)	12.1 (21.6)	6.1 (5.8)	67.7 (67.8)	12.1 (23.2)
Capulin Volcano National Monument	63.1 (59.3)	68.1 (4.7)	5.1 (7.4)	83.1 (20.4)	97.9 (5.4)	20.1 (25.6)
Carl Sandburg Home National Historic Site	73.4 (20.8)	36.3 (78.2)	21.8 (28.1)	77.5 (26.9)	92.6 (4.9)	17.8 (8.9)
Carlsbad Caverns National Park	57.5 (67.5)	72.9 (25.4)	4.3 (1.8)	71.2 (44)	74.3 (33)	17.3 (21)
Casa Grande Ruins National Monument	33.4 (71.3)	68.8 (58.3)	68.2 (17)	8.6 (11.4)	44.3 (56.3)	12.3 (15.8)
Castillo De San Marcos National Monument	32.2 (46.7)	75.9 (67.9)	17.7 (46.4)	76 (39.7)	39.4 (28)	59.7 (27.6)
Catoctin Mountain Park	59.3 (54.3)	70.8 (36.2)	58.1 (21.6)	56 (37.6)	63.9 (42.6)	13.8 (14.7)
Cedar Breaks National Monument	26.9 (25.9)	93.3 (18.3)	72.4 (39.9)	3.5 (1.9)	52.6 (77.3)	8.3 (20.3)
Chaco Culture National Historical Park	47.4 (42.2)	26.2 (37.1)	12.9 (20.2)	24.4 (29.6)	65 (32.9)	9.3 (23.2)
Channel Islands National Park	30.4 (57.3)	42.5 (74.4)	59.9 (30)	37.9 (29.4)	46.1 (55.9)	59.5 (30.8)
Charles Pinckney National Historic Site	35.5 (67.6)	73.3 (18.5)	18 (36.6)	73.7 (34.9)	44.1 (18.8)	41.7 (46.1)
Chattahoochee River National Recreation Area	77.5 (24.4)	38.1 (33.8)	5.8 (8.6)	79.6 (28.1)	87.5 (9.3)	15.7 (16)
Chesapeake and Ohio Canal National Historical Park	61.8 (44.3)	37.3 (90.1)	52.1 (34.7)	55.6 (23.4)	62.8 (53.2)	16.9 (14.6)
Chickamauga and Chattanooga National Military Park	79.8 (37.6)	61 (63.7)	18.7 (31.7)	70.8 (33.7)	72.3 (32.9)	9.5 (12.4)
Chickasaw National Recreation Area	91.6 (25.2)	36.6 (40.5)	45.2 (11.8)	33.6 (34.8)	47.9 (33.7)	17.5 (19)
Chiricahua National Monument	56.3 (80.6)	47.3 (71)	26.6 (52.5)	39.7 (12.1)	59 (76.8)	9.3 (8.3)
City Of Rocks National Reserve	65.6 (11.8)	62.3 (47.2)	37.9 (15.7)	25.9 (43.6)	95.7 (11.7)	49.3 (21.7)
Colonial National Historical Park	66.4 (39.8)	37.8 (58.7)	10.9 (21.2)	62.1 (44.2)	98.1 (5.8)	34.5 (40.8)
Colorado National Monument	33.9 (53.5)	51.4 (62.7)	44.2 (27)	34.3 (56.4)	75.7 (41)	33.1 (21.2)
Congaree National Park	58.5 (43.1)	33.1 (36.1)	11.3 (26)	77.5 (26.4)	78 (7.2)	30 (21.5)
Coronado National Memorial	43.2 (86.4)	56.1 (16.3)	11.3 (7.1)	21.6 (8.1)	38.5 (46.1)	20.8 (20.2)
Cowpens National Battlefield	57.5 (34.4)	66.2 (42.3)	22.3 (21)	77.5 (26.9)	88.2 (10.7)	18.8 (7.8)
Crater Lake National Park	26.9 (36.8)	29.7 (68.9)	23.7 (52.9)	16.4 (21.9)	31 (44.5)	42.9 (27)
Craters Of The Moon National Monument and Preserve	69.1 (38.9)	38.1 (51.7)	49 (45.8)	24.3 (36.6)	91.8 (16.4)	37.4 (21.4)
Cumberland Gap National Historical Park	73.2 (41.5)	72.8 (32.1)	8.9 (12.4)	69.4 (43.8)	99.6 (1.2)	11.7 (12.7)
Cumberland Island National Seashore	20.9 (43.8)	71.9 (24.8)	10.5 (27.1)	73.6 (37)	62.1 (25.7)	42.7 (47.2)
Curecanti National Recreation Area	23.6 (23.8)	80.3 (40.8)	29.5 (52.2)	24.2 (35.1)	72.7 (38.2)	32.1 (25.6)
Cuyahoga Valley National Park	72.9 (15.5)	42.4 (38.3)	50.6 (12)	77.1 (14.6)	96.6 (5.8)	50.4 (28.8)

Park	Bio1	Bio2	Bio3	Bio4	Bio5	Bio6
Death Valley National Park	39 (32.5)	66 (90.3)	51.3 (77.6)	1.7 (1.8)	52.2 (66.2)	64.8 (46.2)
Delaware Water Gap National Recreation Area	84.6 (10)	2.9 (2.8)	18.6 (26.5)	92.6 (12.2)	94.5 (15.5)	33.4 (12.2)
Denali National Park and Preserve	7.9 (10.4)	77 (18.3)	61.4 (52.5)	28.6 (48.7)	88.8 (32.5)	23.2 (34.1)
Devils Postpile National Monument	41.8 (42.8)	45 (66.1)	56.2 (83.1)	16.1 (19.7)	58.4 (42.1)	68.8 (34.4)
Devils Tower National Monument	78.5 (18.7)	71.3 (38.6)	8.8 (19.4)	17.9 (28.6)	96.6 (5.8)	3.1 (3.6)
Dinosaur National Monument	23.3 (40.1)	27.4 (53.9)	24 (16.4)	13.2 (17.6)	75.6 (40.6)	17.8 (42.5)
Dry Tortugas National Park	74.2 (23.8)	67 (33.8)	55.6 (38.7)	78.4 (31.8)	86.8 (17.3)	71.2 (12.8)
Ebey's Landing National Historical Reserve	34.1 (29.7)	24.9 (45.7)	5.9 (14.7)	54.4 (46.5)	35.2 (14)	5.4 (10.4)
Effigy Mounds National Monument	64.1 (27.1)	35.6 (63.2)	5.8 (13.4)	57.7 (42.8)	87 (9.5)	26.1 (18)
Eisenhower National Historic Site	62.8 (42.4)	80.2 (28.2)	68.5 (30)	59.3 (34.4)	76.7 (39.1)	14.8 (17.6)
El Malpais National Monument	35 (50.3)	64.9 (19.9)	48.8 (20.2)	28.7 (19.6)	71.3 (20.2)	8 (18.3)
El Morro National Monument	42.8 (64.9)	69 (19.3)	43.8 (17.7)	20.4 (17.8)	68.9 (26.1)	8.4 (19.3)
Eugene O'Neill National Historic Site	15.7 (29.6)	28.1 (49.8)	12 (3.5)	12.1 (23.2)	28.5 (24.2)	40.8 (4.5)
Everglades National Park	55.8 (49.1)	4.1 (4.4)	26 (35.2)	78.2 (38.8)	75.8 (36.2)	72.7 (12.4)
Fire Island National Seashore	95.4 (6.2)	51.3 (39.5)	18.2 (21.2)	88.7 (14.5)	94.8 (14.6)	37.5 (6.1)
Florissant Fossil Beds National Monument	53.3 (42.9)	62.9 (29.9)	16.2 (19.9)	35.9 (32.2)	95.5 (8.6)	6.3 (14.5)
Fort Bowie National Historic Site	53.5 (81.6)	53.2 (73.4)	34 (68.2)	39.7 (12.1)	54.5 (81.7)	9.6 (7.2)
Fort Caroline National Memorial	27.5 (54.6)	79.4 (52.3)	10.5 (28.1)	75 (36.8)	60 (20.6)	50.3 (35.5)
Fort Davis National Historic Site	80.1 (34.4)	80.1 (26)	1.5 (1.4)	34.2 (45.5)	96.4 (10.8)	8.9 (13.2)
Fort Donelson National Battlefield	74.4 (13)	28 (38.3)	32 (49.4)	55.9 (59.2)	71.3 (28.2)	8.2 (2.5)
Fort Frederica National Monument	27.2 (52.5)	87.6 (9)	10.3 (24.1)	73.6 (37)	56.1 (29.6)	43.4 (45)
Fort Laramie National Historic Site	80.6 (30.3)	38.4 (21.2)	19.5 (25.5)	21.6 (24.4)	94.1 (7.4)	6.4 (13.5)
Fort Larned National Historic Site	55.4 (22.9)	52.8 (52.1)	23.4 (43.2)	25.3 (30.8)	84.7 (26.2)	4.8 (4.3)
Fort Matanzas National Monument	32.9 (46.7)	74.5 (69.7)	25.2 (46.9)	76 (39.7)	30.9 (21.9)	62.9 (23.2)
Fort Moultrie National Monument	35.5 (67.6)	75.5 (21.8)	17.6 (36.6)	73.7 (34.9)	43.6 (27.9)	43.2 (47.4)
Fort Necessity National Battlefield	33.2 (13.6)	63.4 (79.1)	54.3 (62.4)	67.7 (20.1)	88.5 (28.2)	29.1 (2)
Fort Point National Historic Site	18 (23.1)	28 (76)	7.2 (3.9)	21.3 (34.6)	55.9 (33.3)	45.1 (16.7)
Fort Pulaski National Monument	45.5 (38.2)	58.1 (48.1)	9.9 (25.1)	72.5 (38.5)	39.4 (20.1)	36.2 (44.2)
Fort Raleigh National Historic Site	62.4 (38.2)	75.3 (31.4)	16.7 (31)	55 (55.8)	89.8 (28.2)	16.2 (28.5)
Fort Sumter National Monument	35.9 (66.5)	74.5 (24.8)	19.2 (36.6)	73.3 (34.9)	44 (27.9)	42.8 (45)
Fort Union National Monument	58.2 (74.1)	61.3 (69)	27.2 (19.8)	52.5 (20.3)	71.5 (18.3)	12.5 (21.8)
Fort Union Trading Post National Historic Site	38.6 (23.9)	82.9 (35.4)	13.2 (32.7)	23.5 (16.3)	91.3 (8)	13.3 (9.3)
Fort Vancouver National Historic Site	15.5 (18.6)	42.4 (93.2)	16.1 (13.8)	38.9 (33.5)	28.9 (16)	8.3 (20.3)
Fort Washington Park	83.3 (33)	50.3 (43.9)	41 (14.4)	65.5 (29.1)	90.7 (20.4)	20.6 (15.4)
Fossil Butte National Monument	88 (24.3)	17.2 (44.7)	16.6 (10.7)	25.8 (50.6)	93 (13.6)	20.5 (22.5)
Frederick Douglass National Historic Site	83.1 (28.9)	53.6 (46.5)	43.9 (15.9)	67.4 (33.9)	91.7 (17.5)	18.9 (15.6)
Fredericksburg and Spotsylvania National Military Park	78.3 (55.1)	53.1 (42.4)	34.1 (41.8)	63.7 (34.5)	75.4 (45.8)	23.7 (9.4)
Friendship Hill National Historic Site	44.1 (1.6)	65.3 (72.1)	56.9 (53.4)	69.3 (20.1)	93.7 (15.5)	34 (9.7)
Gates Of The Arctic National Park and Preserve	9.9 (11.2)	35.7 (12.8)	50.5 (40.8)	40.9 (50)	48.8 (58.6)	51.8 (56.2)
Gateway National Recreation Area	97.2 (7.2)	7.2 (8)	4.1 (4.4)	92.4 (11.7)	96.1 (10.7)	40.4 (6.7)

Park	Bio1	Bio2	Bio3	Bio4	Bio5	Bio6
Gauley River National Recreation Area	40 (33.4)	26.3 (30.9)	29.7 (27.7)	72.1 (34.1)	99.7 (1)	18.9 (13.5)
George Washington Birthplace National Monument	86 (19.5)	50.8 (47.3)	32.4 (33.8)	64.8 (41.3)	99 (2.9)	23.4 (17.2)
George Washington Carver National Monument	95.5 (13.6)	52.8 (4)	31.1 (19.7)	39.6 (53.9)	62.6 (54.6)	7.5 (7.9)
George Washington Memorial Parkway	78.3 (44)	55.7 (65.4)	43.2 (13.9)	63.3 (27.8)	88.7 (25.2)	19.7 (12.4)
Gettysburg National Military Park	68.5 (30.2)	79.2 (30.1)	67.5 (24.2)	61.6 (38)	83.8 (29.1)	14.8 (17.6)
Gila Cliff Dwellings National Monument	51.8 (65.1)	63.6 (73.4)	48 (87.9)	37.5 (3.9)	62.5 (52.6)	15.3 (23.2)
Glacier Bay National Park and Preserve	20.6 (45.4)	37.3 (10.2)	21.9 (39.6)	8.6 (20.6)	81.3 (29.6)	3.7 (5.6)
Glacier National Park	21.9 (20.2)	19.3 (13.5)	28.9 (66)	15.5 (13.2)	91.6 (7.2)	2.1 (2)
Glen Canyon National Recreation Area	36.6 (9.7)	66.4 (69.6)	27 (47.4)	4.7 (1.5)	60.7 (82.8)	13.8 (26.2)
Golden Gate National Recreation Area	16.1 (23.5)	20.5 (54.3)	3.5 (2.4)	26.3 (34.4)	51.9 (42.6)	46.3 (26.8)
Golden Spike National Historic Site	67.7 (37.3)	35.4 (80.9)	34.9 (43)	30.1 (40.2)	94.5 (15.5)	36.9 (30.4)
Grand Canyon National Park	51.4 (54.3)	100 (0)	71.5 (46.5)	2.8 (1.7)	52.5 (84.8)	6.3 (14.5)
Grand Portage National Monument	98.7 (2.9)	80 (20.7)	33.1 (74.8)	61 (44)	94.6 (8.7)	47 (33.9)
Grand Teton National Park	73.2 (19.8)	11.1 (6.1)	48.2 (46)	34.8 (50)	97.1 (7.8)	14.7 (10.6)
Grant-Kohrs Ranch National Historic Site	71.7 (31.2)	56.9 (69)	30.2 (61.4)	7.6 (12.3)	88.1 (17.5)	8.2 (4.3)
Great Basin National Park	54.7 (6.3)	75.3 (22.2)	69.4 (15)	18.3 (19.9)	74.4 (28.6)	36.7 (49.7)
Great Sand Dunes National Park and Preserve	33.4 (62.3)	99 (2.9)	15.4 (19)	47.4 (73.7)	97.1 (7.8)	44.4 (46.2)
Great Smoky Mountains National Park	62.1 (5.9)	71.2 (49.5)	13.2 (16.2)	69.2 (40.2)	96.5 (6.8)	11.9 (19.7)
Greenbelt Park	83.1 (9.3)	66.4 (42.4)	46.5 (16.6)	69.2 (35.3)	96.9 (4.9)	18.5 (16.6)
Guadalupe Mountains National Park	62.6 (75.3)	71.8 (10.4)	3.3 (3.7)	71.9 (42.8)	77.9 (21)	14.5 (17.4)
Guilford Courthouse National Military Park	76.9 (44.4)	76.8 (26.7)	22.9 (14)	85.8 (12.7)	81 (4)	32.1 (35.8)
Gulf Islands National Seashore	68.3 (22.7)	43.3 (13.1)	40.6 (63.4)	66.2 (53.2)	94.1 (16.5)	39 (51.6)
Hagerman Fossil Beds National Monument	59.3 (45.5)	31.8 (57.4)	30.8 (69.6)	22.3 (32)	78.7 (54.4)	21.6 (30.4)
Haleakala National Park	51 (8)	53.4 (28.6)	38.4 (45.1)	50.9 (39.8)	56.6 (11.8)	25.3 (35.6)
Harpers Ferry National Historical Park	65.7 (64.4)	39.1 (81.4)	45.7 (16.4)	51.3 (51.2)	40 (66.7)	17.4 (12.5)
Hawaii Volcanoes National Park	75.6 (18.9)	83.4 (28.2)	42.9 (30.3)	70.3 (19.4)	88.1 (12)	59.8 (70.3)
Herbert Hoover National Historic Site	71.8 (22.9)	77.4 (24.6)	2.5 (2.5)	60.4 (35.2)	77.9 (29.8)	30.3 (36.1)
Homestead National Monument of America	56.4 (20.9)	43.9 (51.9)	21.7 (32.4)	15.5 (21.9)	22.7 (46.9)	14.5 (15.9)
Hopewell Culture National Historical Park	69.6 (21.8)	38.2 (65)	29.6 (36.3)	65 (35.3)	99.4 (1.9)	24.8 (11.7)
Hopewell Furnace National Historic Site	82.8 (9.8)	54.7 (24)	63 (37.8)	87.2 (13.6)	98.1 (5.8)	22.2 (17.4)
Horseshoe Bend National Military Park	76.8 (12)	38.1 (44.5)	12.2 (15.3)	73 (42.6)	81.6 (32.5)	22.7 (34.4)
Hot Springs National Park	96.1 (11.7)	71.5 (55)	13.7 (23.5)	48.5 (52.5)	61.2 (8.8)	25.1 (60.2)
Hovenweep National Monument	25.8 (50.5)	70.1 (50.8)	33.8 (26.4)	27.8 (35.4)	74.9 (60.7)	23.2 (51.7)
Hubbell Trading Post National Historic Site	28.7 (31.7)	78.1 (42.2)	47.1 (45.1)	6.4 (7.1)	69.5 (68)	12.4 (29)
Indiana Dunes National Lakeshore	67.2 (16.4)	11.5 (13.9)	23.1 (31)	61.1 (44.9)	97.8 (3.3)	32.2 (17.6)
Isle Royale National Park	99.7 (1)	91.9 (13.6)	33.6 (78.7)	69.7 (44.8)	97.3 (4.9)	59.8 (49.5)
Jean Lafitte National Historical Park and Preserve	58.5 (70.9)	38.1 (73)	17.6 (35.6)	49 (73.4)	69.5 (60.2)	33.1 (42)
Jewel Cave National Monument	78.8 (32.5)	58.2 (15.6)	14.5 (16.3)	24 (20.4)	85.2 (23.4)	4.4 (7.5)
John Day Fossil Beds National Monument	34.8 (18.8)	33.9 (35.9)	5.9 (4.6)	14.7 (19.5)	44.6 (64)	3.8 (4.6)
John Muir National Historic Site	21.2 (38.2)	19.3 (41.1)	3.1 (3.6)	9.7 (17.1)	23.6 (28.2)	41.2 (9.1)

Park	Bio1	Bio2	Bio3	Bio4	Bio5	Bio6
Johnstown Flood National Memorial	30.7 (13.5)	29.3 (57.1)	39.1 (15.9)	63.6 (13.8)	83.6 (7.9)	34 (20.8)
Joshua Tree National Park	35.5 (60.4)	52.3 (88.7)	28.2 (14.9)	10.8 (18.9)	66.4 (65.3)	14.3 (20.9)
Kalaupapa National Historical Park	40.4 (3.4)	75.3 (41.3)	12.4 (22.1)	63 (31.9)	61.7 (29.4)	55.8 (68.9)
Kaloko-Honokohau National Historical Park	72.6 (20.5)	92.2 (23.3)	49.6 (42.5)	71.3 (17.5)	88.9 (18.2)	60.3 (73.6)
Katmai National Park and Preserve	74.8 (38.6)	30.5 (43.6)	70.2 (21.3)	43.9 (4)	100 (0)	80.8 (13.2)
Kenai Fjords National Park	40.9 (58.1)	77.6 (47.4)	28.4 (19.5)	26.1 (49.1)	85.4 (8.4)	30.2 (37.7)
Kennesaw Mountain National Battlefield Park	89.6 (22.6)	35.3 (34.9)	5.8 (8.6)	78.9 (28.1)	90.5 (9.2)	15.1 (18.5)
Kings Mountain National Military Park	52.5 (24.5)	81.5 (20.2)	25.7 (15.9)	77.4 (29.3)	86 (11.8)	35.1 (18.4)
Klondike Gold Rush National Historical Park	15.5 (27.9)	49.2 (33.5)	36.2 (32.8)	5 (11)	66.4 (48.9)	5.7 (10.4)
Knife River Indian Villages National Historic Site	55.4 (6.3)	77.2 (48.9)	13.8 (21.8)	23.8 (19.9)	87.2 (16.6)	5.9 (8.5)
Kobuk Valley National Park	33.2 (35.6)	21.4 (20.4)	48.9 (44.9)	37.1 (44.7)	71.2 (34.6)	62.9 (50.4)
Lake Clark National Park and Preserve	48.1 (79.3)	36.2 (29.7)	43.5 (25.7)	34.4 (59.3)	100 (0)	57.7 (16.8)
Lake Mead National Recreation Area	55.4 (75.8)	81.9 (44.7)	55.2 (24.1)	4 (2.5)	57.7 (90.6)	20.4 (48.3)
Lake Meredith National Recreation Area	73.9 (30.7)	49.5 (39.3)	11.6 (16.5)	81.7 (11.2)	95.9 (7.5)	6 (4.4)
Lake Roosevelt National Recreation Area	27.2 (30.2)	2.2 (2.6)	2.5 (3.1)	18.1 (35.5)	46.4 (13.7)	2.1 (1.8)
Lassen Volcanic National Park	81.2 (42.5)	67.1 (62.4)	84.1 (38.6)	50.5 (31.2)	72.6 (69.6)	48 (15.8)
Lava Beds National Monument	41.7 (34.7)	62.4 (90.1)	68.4 (90.2)	19.8 (13.1)	54.6 (76.9)	45.2 (27.5)
Lewis and Clark National Historical Park	38.5 (29.7)	50.4 (87.4)	7.1 (11.1)	26.7 (13.7)	9.1 (9)	15.9 (20.9)
Lincoln Boyhood National Memorial	81.8 (21.2)	63.6 (51.9)	23.1 (35.8)	63.5 (38.4)	100 (0)	14.8 (7.4)
Little Bighorn Battlefield National Monument	36 (16.7)	10.4 (10.5)	2.1 (2.8)	12.8 (18)	90.8 (13.9)	5.4 (10.4)
Little River Canyon National Preserve	83 (35.7)	31.9 (41.9)	21.5 (28.1)	73.7 (34.2)	72.2 (17)	8.4 (10)
Lyndon B. Johnson National Historical Park	86.9 (16.1)	63.1 (48.4)	16.1 (12.1)	30.9 (56.6)	81.9 (34.2)	13.1 (25.8)
Mammoth Cave National Park	84.5 (6.4)	87.8 (18.9)	31.9 (56.7)	69.5 (32.2)	99.6 (1.2)	9.3 (11.9)
Manassas National Battlefield Park	76.9 (54.9)	42.1 (74.9)	39.2 (13.4)	58.2 (46.4)	59.1 (69.5)	23.3 (9.3)
Manzanar National Historic Site	53.3 (53.8)	43.3 (67.4)	60.7 (96.9)	6.4 (10.5)	64 (56.6)	77.5 (24.6)
Marsh-Billings-Rockefeller National Historical Park	97.5 (1.3)	2.3 (3.8)	46.5 (30.5)	72.2 (26.9)	70.6 (41.7)	82.4 (22.1)
Mary McLeod Bethune Council House National Historic Site	79.5 (37.9)	57.1 (53.8)	44.2 (13.9)	66.7 (32.7)	90.7 (20.4)	19.2 (14.5)
Mesa Verde National Park	38.4 (48.1)	36.1 (40)	26 (14.7)	24.5 (34.4)	77.5 (43.5)	24.3 (45.9)
Minute Man National Historical Park	93.7 (9.8)	1.1 (0.2)	14.4 (12.9)	86.2 (24.5)	72.2 (43.7)	66.2 (39.6)
Mississippi National River and Recreation Area	74.8 (11.8)	26.1 (67.9)	20.9 (56.8)	26.5 (36.8)	73 (23.1)	29.7 (17.7)
Missouri National Recreational River	72 (18)	56.5 (49.7)	23.4 (34)	32.5 (24.5)	80.9 (34.2)	16.6 (16.9)
Mojave National Preserve	38.4 (72)	61.8 (95.4)	45.6 (65.9)	2.1 (1.8)	46 (64.3)	23.4 (60.4)
Monocacy National Battlefield	65.7 (62.2)	63 (67)	47.8 (13.7)	49.5 (33.1)	52.5 (64.7)	18.6 (9.4)
Montezuma Castle National Monument	44.9 (84.1)	58.6 (29.4)	42.7 (58.7)	6.1 (5.8)	41.1 (67.9)	4.7 (9.6)
Moores Creek National Battlefield	25.6 (48)	85.2 (21)	34.4 (39.6)	63.3 (43.8)	74.9 (31.7)	20.4 (29.4)
Morristown National Historical Park	93.9 (6.3)	5.5 (9.6)	18.1 (17.3)	91.2 (13.1)	95.8 (11.7)	37.5 (11.6)
Mount Rainier National Park	8.6 (14.4)	6.3 (9.6)	13.5 (20.5)	29.7 (27.4)	21.6 (33)	5.1 (8.5)
Mount Rushmore National Memorial	76.6 (32.7)	89.1 (15.1)	6.1 (10.2)	21.6 (24)	86 (20.9)	3.8 (4.6)
Muir Woods National Monument	19.7 (27.6)	22.9 (62.8)	3.9 (2.4)	24.4 (39.7)	55.2 (41.9)	44.9 (25.9)
Natchez Trace Parkway	52 (18.2)	62.5 (49.5)	30.5 (36.1)	54.2 (56.8)	74.1 (23.5)	22 (29.7)

Park	Bio1	Bio2	Bio3	Bio4	Bio5	Bio6
National Capital Parks-East	80.8 (34)	57.9 (55)	44.2 (13.9)	66.7 (32.7)	91.3 (18.4)	19.2 (14.5)
National Park of American Samoa	80.5 (16.8)	70.5 (17.9)	95.9 (6.6)	98.6 (2.2)	87.1 (9.3)	57.9 (14.6)
Natural Bridges National Monument	25.1 (30.9)	58.1 (73.4)	30.5 (52.2)	13.8 (15.5)	70.5 (65.8)	17.2 (39.3)
Navajo National Monument	50.9 (15.3)	95 (15.1)	46.4 (64.5)	5.2 (4.4)	58.5 (93.5)	18.7 (36.9)
New River Gorge National River	42.6 (41.2)	26.4 (34.4)	25.9 (36.2)	64.3 (45.2)	96.9 (5.4)	18.8 (14.5)
Nez Perce National Historical Park	27.4 (25.9)	5 (8.9)	12.5 (31.5)	9.4 (9.3)	64.6 (41.9)	2.4 (2.7)
Ninety Six National Historic Site	64 (47.5)	83.4 (25.5)	10.7 (23.1)	77.4 (31.8)	85.3 (8.8)	26.8 (2.9)
Niobrara National Scenic River	83.1 (31.7)	78.5 (16.6)	9.6 (13.5)	15.1 (24.7)	87.6 (25.5)	7.2 (4.4)
Noatak National Preserve	37.5 (37.2)	24.2 (23)	55.8 (44.2)	38.1 (42.8)	73.9 (28.6)	56.3 (54)
North Cascades National Park	28.8 (7.2)	1.4 (1.2)	6.8 (10)	22.4 (25.6)	24 (9.3)	3.5 (3.7)
Obed Wild and Scenic River	62.4 (13.4)	81 (28.9)	39.5 (61.1)	65.3 (41.7)	82.1 (29.6)	12.1 (9.1)
Ocmulgee National Monument	20.5 (27.3)	74.5 (12.1)	11.7 (24.9)	72.1 (37.5)	73.9 (32.5)	21.1 (27.5)
Olympic National Park	31.3 (16.7)	79.7 (43.7)	13.9 (18.6)	29.7 (30.1)	10 (1.1)	15.8 (23.4)
Oregon Caves National Monument	31.1 (32)	41.5 (99)	53.5 (63.3)	88.9 (21.6)	24.8 (51.1)	52.3 (27)
Organ Pipe Cactus National Monument	2.1 (1.7)	56.1 (76.3)	49.2 (21.6)	2.1 (2.8)	64 (45)	12.5 (20.9)
Ozark National Scenic Riverways	69 (35.6)	20.7 (29.3)	23.8 (54.9)	52.8 (53.8)	31.1 (61.4)	20.3 (35.9)
Padre Island National Seashore	61.7 (2.4)	88 (4)	19.1 (7.6)	13 (18.2)	39.3 (62.7)	13.4 (25.8)
Palo Alto Battlefield National Historical Park	59.1 (13.5)	46.4 (5.3)	27.1 (33.1)	13.9 (23.3)	26.6 (47.8)	31.8 (56.2)
Pea Ridge National Military Park	89 (16.4)	34.9 (46)	29.9 (13.3)	38 (52.8)	64 (54.6)	8.4 (14)
Pecos National Historical Park	44 (83.6)	67.4 (69.2)	26.1 (37.1)	46.7 (18.4)	68.2 (12.6)	7.8 (11.5)
Petersburg National Battlefield	74.2 (38.8)	50 (64.6)	17.5 (23.4)	63.6 (32.3)	98.4 (4.9)	33.8 (50)
Petrified Forest National Park	14.6 (16.1)	98.8 (2.4)	59.9 (48.6)	8.1 (9.3)	62.6 (55.7)	10.1 (21.2)
Petroglyph National Monument	35.1 (32.8)	56.2 (66.9)	44.8 (62.8)	31.5 (20.3)	69.1 (29.3)	5.4 (10.6)
Pictured Rocks National Lakeshore	89.8 (14.4)	81.1 (27.3)	41 (89.8)	70.3 (58.1)	88.8 (24.7)	53 (13.1)
Pinnacles National Monument	54.2 (37.7)	22.2 (35.6)	64 (34.9)	29.1 (24.8)	60 (23)	61.3 (30)
Pipe Spring National Monument	52.3 (38.1)	100 (0)	75.6 (40)	3.9 (3)	61.8 (75.5)	9.3 (23.2)
Pipestone National Monument	77.1 (22.5)	46.2 (57.9)	34.8 (41.1)	50.2 (29.8)	80.9 (16.1)	26.6 (21.6)
Piscataway Park	82.9 (34)	54.2 (52.1)	41.3 (13.5)	65.8 (28)	90.7 (20.4)	21 (14.3)
Point Reyes National Seashore	16.1 (22.5)	20.2 (52.3)	4.2 (3)	32.7 (41.3)	46.3 (45.8)	45.6 (23.7)
Port Chicago Naval Magazine National Memorial	24.5 (45.7)	18.7 (35.2)	3.5 (3.6)	10.5 (20.6)	16.9 (21.4)	45.4 (17.8)
Presidio of San Francisco	18.4 (21.9)	28 (76)	6.8 (2.8)	20.6 (34.6)	55.5 (33.3)	44.4 (16.8)
Prince William Forest Park	77.2 (53.9)	46.7 (73.2)	37.1 (22.5)	63.9 (39.2)	69.6 (51.3)	24.9 (11.8)
Pu'uhonua o Honaunau National Historical Park	73 (24.6)	90.2 (28.2)	49.7 (26.2)	70.6 (17.2)	86.7 (11.8)	60.2 (74.8)
Puukohola Heiau National Historic Site	67.9 (31.4)	92 (17.5)	52.9 (56)	72.4 (17)	94.8 (15.5)	45.6 (65.9)
Rainbow Bridge National Monument	53.9 (14.5)	84.7 (32.3)	35.8 (60)	3.9 (3)	60.1 (86)	14.6 (31.6)
Redwood National Park	83.6 (42.5)	47.4 (83.6)	63.7 (23.5)	90.4 (11.7)	59 (86)	65.3 (12.9)
Richmond National Battlefield Park	78.3 (44.7)	56 (53.2)	18.2 (30.3)	60.8 (40.2)	98.4 (4.9)	31.7 (43.9)
Rio Grande Wild and Scenic River	89.5 (16.9)	91.1 (17.3)	1.5 (1.4)	24.6 (34.3)	83.8 (23.1)	5.7 (11.4)
Rock Creek Park	79.4 (36)	60.3 (62)	44.2 (13.9)	66.7 (32.7)	91.3 (18.4)	19.2 (14.5)
Rocky Mountain National Park	55.2 (13)	85.3 (32)	32.3 (20.2)	24.4 (20.4)	91.8 (11.4)	8.6 (19.2)

Park	Bio1	Bio2	Bio3	Bio4	Bio5	Bio6
Roosevelt-Vanderbilt Headquarters	96.7 (0.7)	64.1 (97.8)	7.9 (4.9)	86.4 (9.9)	91.7 (19.4)	54.8 (35.5)
Russell Cave National Monument	82 (29.2)	61.2 (59.2)	29.3 (39.2)	62 (51.3)	75.9 (32.5)	6.8 (5.5)
Sagamore Hill National Historic Site	95.1 (8.6)	9.5 (14.8)	4.4 (6.6)	88 (8.9)	93.1 (16.5)	44.3 (16.4)
Saguaro National Park	24.3 (50.2)	6.9 (7.3)	36.2 (26.9)	19.6 (12.4)	52 (60.4)	17.2 (30.4)
Saint Croix National Scenic Riverway	80.7 (12.5)	23 (61.7)	23.1 (60.4)	28.6 (49.2)	72.3 (35.7)	36.4 (19.8)
Saint-Gaudens National Historic Site	96.4 (3.4)	10.6 (26.7)	37 (12)	71.7 (19.4)	70.9 (40.6)	80.5 (20.6)
Salinas Pueblo Missions National Monument	49.3 (77.4)	76.4 (50.3)	28.4 (42.4)	36.9 (10.7)	77.1 (29.9)	19 (42.3)
San Antonio Missions National Historical Park	81.7 (8.1)	90.8 (17.1)	5.6 (7.6)	29.4 (38.4)	82.4 (39.8)	10 (20)
San Juan Island National Historical Park	49.6 (25.3)	32.4 (66.3)	8.9 (8.6)	41.5 (35)	28.6 (21.3)	7.2 (12.4)
Sand Creek Massacre National Historic Site	37.7 (32.4)	48.3 (27.7)	15 (13.9)	44.5 (12.8)	89.1 (15.1)	9.4 (16.8)
Santa Monica Mountains National Recreation Area	55.1 (96.6)	65.6 (99)	64.2 (55.7)	27.6 (44.7)	75.9 (57)	41.5 (20.8)
Saratoga National Historical Park	88.5 (12)	53.7 (79.7)	21.4 (14.5)	80.6 (11.9)	77.1 (30.4)	69.6 (39.3)
Saugus Iron Works National Historic Site	81.8 (28.2)	4.3 (9.9)	11.2 (23.4)	80.6 (18.8)	69.7 (44.4)	53 (42.9)
Scotts Bluff National Monument	83.5 (16.7)	59.9 (4.1)	17.8 (33.5)	19.1 (24.3)	96.5 (7.5)	6.7 (15.4)
Sequoia and Kings Canyon National Parks	59.3 (60.3)	55.1 (76.8)	65.4 (99)	5.5 (4.6)	66.6 (37.3)	88.1 (17.4)
Shenandoah National Park	73.4 (48.8)	82.7 (34.4)	37.6 (21.3)	46.3 (32.2)	62.9 (56.1)	25.5 (26.7)
Shiloh National Military Park	80 (3.1)	50.4 (65.3)	41.9 (58.6)	56 (47.6)	72.7 (32.9)	14 (13.1)
Sitka National Historical Park	34.3 (54.4)	41.3 (17.7)	17.5 (30.6)	9 (17)	64.8 (30.7)	1.8 (0.9)
Sleeping Bear Dunes National Lakeshore	80.4 (3.1)	87.7 (22.6)	32 (60.5)	71.3 (45.8)	97.6 (3.9)	46.7 (20.3)
Stones River National Battlefield	86.8 (5.3)	82.3 (35.1)	44.8 (54.8)	62.5 (45.2)	85 (18.5)	10.9 (10.3)
Sunset Crater Volcano National Monument	52.7 (61.5)	91.8 (16.1)	73.5 (50.6)	2.8 (2.7)	41.7 (83.6)	5 (11.5)
Tallgrass Prairie National Preserve	78.6 (6.9)	59.4 (32.9)	11.2 (8)	25.3 (28.7)	76.1 (47)	8.8 (5.3)
Theodore Roosevelt National Park	38.4 (39)	77.8 (47.3)	6.8 (13.4)	18.7 (19.2)	90.4 (5.7)	4.8 (5.6)
Thomas Stone National Historic Site	82.9 (33)	55.4 (55.2)	35.1 (20)	66.7 (32.7)	91.3 (18.4)	22.2 (13.2)
Timpanogos Cave National Monument	86.7 (9)	19.5 (49.5)	41.5 (57.8)	45.9 (59.6)	79.7 (33.8)	36.8 (40.4)
Timucuan Ecological and Historic Preserve	26.7 (53.4)	79.6 (48.3)	10.5 (28.1)	74.3 (35.8)	61.2 (22.6)	48.9 (35.3)
Tonto National Monument	42.7 (60)	88.8 (11.4)	96.5 (7.2)	13.7 (19)	57.1 (63.3)	22.5 (31.6)
Tumacacori National Historical Park	25.8 (64.2)	31.1 (29)	51.8 (43.9)	18.3 (3.3)	44.7 (48)	26 (35.8)
Tuzigoot National Monument	38.1 (83.1)	76.8 (33)	46.5 (61.3)	5 (3.9)	41.8 (77.6)	5.7 (12.5)
Upper Delaware National Scenic and Recreational River	91.6 (4.7)	3.4 (4.1)	18.8 (24.8)	92.6 (12.2)	95.1 (13.6)	52.4 (23)
Valley Forge National Historical Park	85 (8.7)	23.3 (17.3)	41.7 (28.9)	85.5 (8.6)	95.8 (12.6)	25.3 (13.9)
Valor in the Pacific National Monument	32.3 (44.1)	53.2 (75.6)	61.3 (75.9)	22.4 (6)	45.3 (55.1)	45.5 (44.5)
Vicksburg National Military Park	49.1 (9.1)	32.2 (5.9)	20.2 (31.7)	50.1 (62.8)	71 (25.9)	17.5 (21.3)
Virgin Islands National Park	28.1 (19.4)	1.4 (1.2)	8.5 (3.5)	61.9 (30.5)	13.6 (20.7)	17.4 (14.1)
Voyageurs National Park	83.6 (13.4)	4.3 (9.9)	19.5 (23.7)	31.8 (46.4)	73.5 (56.6)	54.5 (28.8)
Walnut Canyon National Monument	51.5 (77)	86.7 (20.5)	68.6 (56.6)	2.8 (2.7)	39.7 (77.6)	5 (11.5)
War In The Pacific National Historical Park	63.4 (18.2)	49.4 (20.4)	60.7 (14.3)	53.4 (21.2)	69.1 (27.1)	50.1 (7.6)
Washita Battlefield National Historic Site	85.9 (35)	56.5 (27.2)	11.8 (7)	38.6 (33)	66.1 (55.1)	6 (4.4)
Weir Farm National Historic Site	96.3 (5.5)	1.1 (0.2)	11.4 (18.8)	89.8 (8.1)	91 (19.4)	47.1 (20.6)
Whiskeytown National Recreation Area	20.6 (11.5)	29.4 (83.3)	20.5 (40.1)	15.7 (12.5)	47.3 (79.5)	68.9 (18.9)

Park	Bio1	Bio2	Bio3	Bio4	Bio5	Bio6
White Sands National Monument	46.9 (90.3)	72.3 (56.8)	22.9 (22.4)	64.7 (35.7)	82.7 (28.2)	18.7 (40)
Whitman Mission National Historic Site	38.3 (25.1)	47.4 (4.2)	4.3 (1.6)	12.6 (17.5)	46.2 (29.4)	2.5 (2.8)
Wilson's Creek National Battlefield	73.3 (16.9)	73.3 (28.2)	28.6 (38.8)	39.5 (56.9)	73.3 (33.9)	7.7 (4.8)
Wind Cave National Park	77.4 (31.5)	71.4 (9)	6.4 (11.2)	21.5 (20.6)	84.5 (23.6)	3.7 (5.6)
Wolf Trap National Park for the Performing Arts	74.3 (53.7)	54.6 (73.9)	41 (11.4)	57.9 (27.1)	68.4 (48.4)	22.7 (13.9)
Wrangell - St Elias National Park and Preserve	12.3 (17.1)	30.9 (29.4)	4.6 (10.6)	21.5 (38.5)	90.5 (9.2)	3.4 (4.6)
Wright Brothers National Memorial	65.3 (37.5)	76 (31.4)	15.8 (31.9)	54.3 (56.1)	89.8 (28.2)	16.2 (28.5)
Wupatki National Monument	56.8 (55.5)	98.5 (2.4)	75.2 (51.8)	2.8 (2.7)	47.9 (96.9)	6.7 (15.4)
Yellowstone National Park	51.7 (22)	15.3 (18.7)	35 (37.7)	26.4 (26.6)	95.7 (9.7)	8.8 (16.2)
Yosemite National Park	42.2 (46.2)	53.4 (80.4)	67.7 (91.2)	24 (30.8)	59.3 (58.2)	68.3 (31.9)
Yucca House National Monument	33.3 (62.2)	51 (47.4)	23.8 (26.8)	26.7 (34.4)	79.9 (48.3)	20.7 (42.5)
Yukon-Charley Rivers National Preserve	6.5 (9.5)	3 (5.7)	20 (24.5)	24 (19.7)	43 (13.2)	9.6 (16.2)
Zion National Park	49.9 (32.8)	100 (0)	75.9 (31.3)	3.5 (1.9)	62.2 (74.1)	9.3 (22.2)
