# Supplementary material for: Climate Exposure of US National Parks in a New Era of Change
Source: PLoS One. 2014 Jul 2;9(7):e101302. doi: 10.1371/journal.pone.0101302 (PMC4079655; doi:10.1371/journal.pone.0101302)
Supplement: Appendix S6 — Mean percentile and maximum difference in percentile (in parentheses) for moving window standard deviations: temperature annual range (Bio7), mean temperature of the wettest quarter (Bio8), mean temperature of the driest quarter (Bio9), mean temperature of the warmest quarter (Bio10), and mean temperature of the coldest quarter (Bio11). (PDF) [file pone.0101302.s006.pdf]

Appendix S6. Mean percentile and maximum difference in percentile (in parentheses) for moving window standard deviations: temperature annual range (Bio7), mean temperature of the wettest quarter (Bio8), mean temperature of the driest quarter (Bio9), mean temperature of the warmest quarter (Bio10), and mean temperature of the coldest quarter (Bio11).

Park	Bio7	Bio8	Bio9	Bio10	Bio11
Abraham Lincoln Birthplace National Historical Park	22.5 (22.9)	21.4 (47.2)	68.4 (17.7)	91.2 (7)	55.9 (48.9)
Acadia National Park	14.7 (15.7)	59.4 (47)	86.3 (11.1)	41.2 (53.6)	57.5 (55.6)
Agate Fossil Beds National Monument	9.9 (14.7)	80.9 (24.2)	2.1 (2.8)	93.8 (13.5)	26.4 (12.8)
Ala Kahakai National Historic Trail	86.7 (30.1)	77 (57.6)	27.8 (37.9)	82.8 (39.3)	62.9 (38.9)
Alagnak Wild River	79.9 (10.4)	66.5 (13.3)	11.6 (5.2)	99.6 (1.2)	53.5 (38.3)
Alibates Flint Quarries National Monument	29.6 (39.3)	88.8 (22.2)	82.3 (17.3)	99.6 (1.1)	16.8 (19.7)
Allegheny Portage Railroad National Historic Site	35.7 (33.9)	61.1 (14.1)	22 (2.5)	72.9 (5.8)	55.5 (41.5)
American Memorial Park	63.2 (32.4)	57.6 (18.4)	43.6 (55.8)	57.5 (33)	48.1 (42.9)
Amistad National Recreation Area	17.2 (40.1)	78 (14.1)	32.6 (47.7)	89.8 (14.1)	32.7 (32.6)
Anacostia Park	24.7 (8.5)	18.8 (16.3)	74.7 (26.2)	87.5 (30.8)	70.5 (34.2)
Aniakchak National Monument and Preserve	92.1 (10.6)	79.4 (32.8)	9.4 (8.1)	81.7 (50.6)	76.8 (47.4)
Antietam National Battlefield	20.1 (20.4)	22.4 (33)	57.4 (53.3)	65.1 (56.9)	59.2 (38.7)
Apostle Islands National Lakeshore	14.4 (32.9)	40.8 (41.7)	73.6 (7.8)	54.1 (43.1)	72.5 (12.1)
Appalachian National Scenic Trail	36.1 (36.3)	22.4 (40.5)	42 (19.3)	93.4 (5.1)	84.8 (24.8)
Appomattox Court House National Historical Park	44.2 (17.5)	22.4 (38.5)	77.9 (39.7)	79.4 (55.1)	59.4 (52.2)
Arches National Park	30.3 (22.9)	36.3 (76.7)	63.1 (24.2)	87.1 (20.4)	24.7 (8.6)
Arkansas Post National Memorial	28.8 (12.2)	64 (43)	27.8 (16.7)	96.2 (7.2)	43.6 (50.3)
Assateague Island National Seashore	21.9 (18.9)	16.4 (11.8)	96.9 (8.4)	97 (5.7)	75.5 (34.2)
Aztec Ruins National Monument	6.9 (16.3)	47 (77.5)	13.5 (24)	86.6 (27.5)	34.4 (58.5)
Badlands National Park	1.1 (0.2)	47.2 (31.7)	5.6 (7.3)	89.1 (16.2)	23.4 (17.1)
Baltimore-Washington National Parkway	25.5 (11.7)	23.1 (20.6)	70.2 (32.2)	95.9 (6.6)	70.8 (34.2)
Bandelier National Monument	34.5 (45.2)	86.5 (14.3)	58.7 (42.3)	96 (8.7)	37 (63)
Bent's Old Fort National Historic Site	34.9 (39.1)	37 (17.7)	29.7 (15.8)	81.9 (14.6)	18.1 (16.7)
Bering Land Bridge National Preserve	57 (37.3)	72.2 (43.8)	43.2 (35.9)	87.3 (6.3)	53.7 (12)
Big Bend National Park	36.4 (33.4)	48.4 (19.2)	24.1 (23.9)	82.1 (44.1)	14.7 (15.8)
Big Cypress National Preserve	54.2 (47.3)	35.8 (68.4)	54 (37.5)	79.4 (12.3)	73.4 (42.1)
Big Hole National Battlefield	8.1 (10)	72 (21.6)	53.8 (22.6)	81.4 (40.6)	19.5 (23.3)
Big South Fork National River and Recreation Area	23.2 (21.9)	24.1 (8.6)	46.7 (40.2)	77.7 (32.5)	47.8 (44.6)
Big Thicket National Preserve	32.4 (56.4)	14.5 (25)	86.3 (24.6)	91.6 (5.9)	43.6 (55.2)
Bighorn Canyon National Recreation Area	6.9 (13.7)	39.6 (15.7)	87.1 (21.1)	73 (5.4)	12.7 (5.9)
Biscayne National Park	55.4 (33.8)	31.8 (33.8)	47.9 (46.3)	77.4 (29.5)	76.1 (36.6)
Black Canyon Of The Gunnison National Park	57.6 (28.1)	47.1 (84.5)	56.5 (45.5)	91.3 (20.3)	20.4 (13.1)
Blue Ridge Parkway	35 (22.2)	52.4 (53.5)	45.9 (33.4)	96.1 (8.7)	66.2 (53.5)
Bluestone National Scenic River	25.6 (33.1)	6 (8.7)	65.8 (36.5)	75.2 (51.3)	54.3 (63.8)
Booker T. Washington National Monument	28.4 (21.3)	50.6 (40.5)	49.6 (50.3)	72.1 (17.9)	74.2 (40.5)
Boston Harbor Islands National Recreation Area	29.7 (38.5)	56 (1.3)	86.2 (28.3)	57.7 (41.8)	63.4 (30.2)

Park	Bio7	Bio8	Bio9	Bio10	Bio11
Bryce Canyon National Park	11 (17.1)	39.1 (69.2)	23.8 (24.7)	62.3 (68.5)	19.8 (17.4)
Buck Island Reef National Monument	16 (7.6)	46.8 (6.7)	19.6 (32.2)	49.9 (39)	29.2 (18.1)
Buffalo National River	22.6 (16.3)	67 (51.5)	64.7 (36)	61.2 (51.5)	54.4 (42.1)
Cabrillo National Monument	83 (21.4)	11.1 (22)	29.6 (16.2)	75.9 (27.7)	19.5 (47.3)
Canaveral National Seashore	36.7 (25.8)	98.6 (1.2)	69.9 (50.3)	64 (13.1)	70.3 (37.8)
Canyon De Chelly National Monument	13.8 (16.1)	80.1 (15.6)	35.5 (42.9)	70.9 (67)	22.6 (38.9)
Canyonlands National Park	23.8 (24.3)	40.1 (81.4)	61.2 (32.9)	83.2 (29.7)	13.5 (14)
Cape Cod National Seashore	23.4 (27.6)	78.6 (19.1)	92.8 (18.4)	71.5 (43.5)	67.5 (44.3)
Cape Hatteras National Seashore	16.9 (8.1)	36.2 (12.8)	94.7 (7.5)	58.1 (50.6)	59.2 (59.3)
Cape Krusenstern National Monument	50.3 (47.9)	53.1 (21.6)	46.3 (47.3)	91.6 (11.6)	55 (15.9)
Cape Lookout National Seashore	14.9 (6.6)	28.3 (11.4)	92.2 (7.9)	48.9 (44.9)	61.6 (55.2)
Capitol Hill Parks	23.6 (9.5)	18.8 (16.3)	76.3 (22.6)	87.2 (31.8)	70.8 (34.2)
Capitol Reef National Park	10.6 (13.3)	21.5 (30.1)	77.8 (9.2)	85.9 (25.3)	16.1 (19.8)
Capulin Volcano National Monument	67.8 (41)	20.9 (37.2)	5 (10.6)	99.6 (1.1)	23.1 (32.5)
Carl Sandburg Home National Historic Site	33 (23.3)	67.4 (30.4)	35.2 (20.8)	94.1 (14.5)	68.4 (41.2)
Carlsbad Caverns National Park	50.1 (62.5)	49.4 (54)	82.8 (40.8)	85 (24.1)	27.2 (12.3)
Casa Grande Ruins National Monument	8.4 (10.4)	85.8 (14.5)	61.1 (22.4)	40.5 (90.6)	33.9 (32.9)
Castillo De San Marcos National Monument	48.7 (17.4)	36.4 (52.5)	50.2 (10.8)	83.2 (12.3)	69.6 (37.8)
Catoctin Mountain Park	13.8 (19.9)	17.7 (35.6)	68.9 (57.6)	66.8 (52.5)	58 (38.5)
Cedar Breaks National Monument	13.6 (16.2)	51.6 (50)	19.7 (21.9)	69.5 (67.8)	19.9 (20.6)
Chaco Culture National Historical Park	8.8 (17.3)	32.6 (33.3)	10.9 (26)	85.3 (29.6)	29.1 (53.5)
Channel Islands National Park	60.5 (37.3)	8.1 (15.3)	56.5 (27.6)	37.1 (88.7)	18.3 (8.1)
Charles Pinckney National Historic Site	27.8 (32.4)	68.4 (57.1)	71.2 (27.3)	88.1 (18.5)	64.5 (39.7)
Chattahoochee River National Recreation Area	20 (18.6)	69.7 (34.3)	7.5 (14.8)	90.2 (22.9)	62.3 (52.5)
Chesapeake and Ohio Canal National Historical Park	22.6 (16.3)	35.3 (44.7)	63.9 (48.1)	73.7 (45.9)	60.3 (48.4)
Chickamauga and Chattanooga National Military Park	13.5 (23.1)	90 (20.4)	40.7 (15.1)	94.8 (5.5)	51.2 (58.7)
Chickasaw National Recreation Area	29.7 (37.4)	91.1 (21.1)	57.7 (44.1)	87.6 (19.5)	35.2 (45.6)
Chiricahua National Monument	7.9 (15.8)	57.1 (23.8)	55.6 (38.6)	71.1 (53.4)	69.9 (19.1)
City Of Rocks National Reserve	49.1 (9.3)	5.1 (6.6)	80.9 (30.8)	80.4 (45.6)	55.4 (40.9)
Colonial National Historical Park	48.9 (14.9)	32.1 (34.9)	96.8 (3.8)	91.9 (9.4)	63.3 (61.8)
Colorado National Monument	45.7 (35.4)	34.7 (61)	45.2 (31.3)	94.2 (17.5)	39 (24)
Congaree National Park	27.3 (15.3)	69.7 (13.6)	57.6 (8.9)	88.7 (25.3)	63.7 (34.8)
Coronado National Memorial	6.1 (10.5)	76.6 (13.7)	66.2 (55.7)	45.6 (91)	62.1 (17.4)
Cowpens National Battlefield	40.1 (15.5)	53.2 (36.9)	37 (42)	88.3 (16.1)	68.7 (44.1)
Crater Lake National Park	37.2 (28.2)	29.8 (8.2)	11.9 (9.4)	29.6 (54.7)	46.8 (25.1)
Craters Of The Moon National Monument and Preserve	45.4 (26.3)	4.4 (7.5)	52.7 (69.4)	85.8 (36.8)	45.3 (33.3)
Cumberland Gap National Historical Park	41.9 (12.8)	10.8 (19.2)	52.8 (36.5)	98.6 (1.2)	56.7 (38.2)
Cumberland Island National Seashore	30.8 (39.4)	75.2 (18.7)	69.2 (12.2)	82.2 (18)	65.2 (40.9)
Curecanti National Recreation Area	37.9 (37.7)	46.5 (82.4)	65.4 (34.6)	87.8 (21.3)	15 (14.3)
Cuyahoga Valley National Park	19.9 (15.6)	37.9 (63.9)	100 (0)	95.5 (7.5)	79.5 (22.1)

Park	Bio7	Bio8	Bio9	Bio10	Bio11
Death Valley National Park	44.2 (34)	26.8 (34.5)	31.8 (37.5)	36.9 (40.9)	27.4 (21.1)
Delaware Water Gap National Recreation Area	25.6 (34.7)	66.4 (28.5)	52.1 (27.6)	91 (11.2)	92.3 (6)
Denali National Park and Preserve	25 (41.4)	76.5 (26.4)	38.3 (16.4)	95.7 (10.8)	4.6 (7.5)
Devils Postpile National Monument	44 (31.2)	19 (32.7)	42.9 (56.5)	28.5 (34.2)	22.2 (12.9)
Devils Tower National Monument	1.4 (0.9)	24.7 (34)	10.5 (7.6)	85.8 (8.8)	36.9 (2.8)
Dinosaur National Monument	10.2 (15.7)	31.6 (38.5)	92.8 (10.8)	81.3 (15.9)	11.1 (10.5)
Dry Tortugas National Park	63.5 (48.6)	83.9 (26.5)	88.9 (9.1)	100 (0)	76.3 (31.8)
Ebey's Landing National Historical Reserve	21.9 (21.8)	11.1 (19.2)	2.1 (2.8)	61.6 (36.7)	36.3 (37.2)
Effigy Mounds National Monument	11.4 (27.9)	36.9 (24.5)	9.6 (12.8)	68.9 (31.5)	70.1 (6)
Eisenhower National Historic Site	11.3 (20.7)	16.6 (34.8)	77.2 (45.4)	76.1 (33.8)	57.8 (37.4)
El Malpais National Monument	28.3 (30)	75.5 (22.8)	40.5 (15.2)	83.2 (32.3)	11.9 (20.3)
El Morro National Monument	20.6 (5.4)	61.9 (42.5)	26.8 (18.9)	81 (39.1)	11.7 (17.4)
Eugene O'Neill National Historic Site	14.2 (26.5)	4 (8.6)	56.6 (80)	24.8 (6.2)	30.8 (11.5)
Everglades National Park	58.4 (40.8)	46.4 (64)	59 (43.6)	84.8 (11.8)	74.2 (40.7)
Fire Island National Seashore	41.5 (40.4)	71.1 (6.3)	80.3 (19.3)	98.6 (1.2)	66.4 (38.7)
Florissant Fossil Beds National Monument	7.7 (15.3)	45 (57.3)	41.7 (47)	98 (4.9)	21.7 (12.1)
Fort Bowie National Historic Site	10.3 (23)	55.1 (27.6)	54.9 (23.1)	64.9 (61.2)	68.7 (23.7)
Fort Caroline National Memorial	37.9 (41.9)	50.9 (41.4)	61 (9.1)	85.1 (24.9)	68.5 (40.2)
Fort Davis National Historic Site	31.6 (41)	21.2 (26.2)	97.7 (5.8)	67.1 (75.3)	15.9 (16.6)
Fort Donelson National Battlefield	21.8 (8)	41.1 (46.3)	62.6 (4.8)	62.7 (42.1)	44.9 (56.4)
Fort Frederica National Monument	33 (29.8)	77.2 (25.8)	75.4 (24.1)	87 (13.1)	65.6 (40.7)
Fort Laramie National Historic Site	10.8 (15.2)	85.4 (10.8)	4.9 (7.7)	93.7 (15.9)	22.6 (8.9)
Fort Larned National Historic Site	15.4 (16.7)	73.4 (22.4)	66.2 (19.9)	57.9 (44)	24 (21)
Fort Matanzas National Monument	51.1 (16.3)	46 (71.8)	52.7 (8.9)	80.1 (15.3)	69.6 (37.8)
Fort Moultrie National Monument	29.8 (40.5)	69.7 (55.1)	72.2 (24.4)	87.7 (15.2)	65.7 (37.3)
Fort Necessity National Battlefield	37.4 (5.8)	81.7 (38.4)	40.8 (25.4)	84.1 (27.5)	51.9 (32.9)
Fort Point National Historic Site	12 (26.8)	8.8 (17.1)	14.6 (16.6)	50.5 (13.4)	29.4 (13.7)
Fort Pulaski National Monument	25.9 (32.7)	86.6 (17.5)	87.1 (1.8)	93.7 (18.1)	61.6 (42.3)
Fort Raleigh National Historic Site	21.1 (12.6)	23.6 (11)	60.4 (44)	58.4 (63)	60.1 (60.4)
Fort Sumter National Monument	30.3 (33.7)	70.3 (52.2)	71.8 (24.4)	87.3 (14.2)	65.3 (38.5)
Fort Union National Monument	25.7 (29.1)	82.8 (11.8)	4.1 (7.4)	99.6 (1.1)	26.3 (26.2)
Fort Union Trading Post National Historic Site	10.4 (24.2)	2.2 (2.5)	17.4 (9.3)	69.3 (35)	25.8 (11.8)
Fort Vancouver National Historic Site	40.8 (27.9)	30 (47.5)	26.4 (26.6)	33.8 (2.9)	27.2 (21)
Fort Washington Park	22.2 (8.4)	20.2 (15.4)	82.6 (15.1)	86.2 (33.7)	70.5 (33.1)
Fossil Butte National Monument	20.5 (12.4)	5.3 (11.4)	70 (83.5)	96.8 (9.7)	42.8 (63.9)
Frederick Douglass National Historic Site	23.3 (9.5)	18.5 (15.2)	77.4 (21.4)	87.5 (30.8)	71.2 (33)
Fredericksburg and Spotsylvania National Military Park	23.8 (12)	9.7 (8.3)	80.5 (21.4)	79.2 (40.6)	68.8 (37.5)
Friendship Hill National Historic Site	41 (3.3)	79.3 (21.9)	50 (30.6)	83.8 (27.9)	67.7 (40.2)
Gates Of The Arctic National Park and Preserve	47.8 (66.8)	54.6 (36.7)	20.5 (16.8)	55.4 (64.7)	15.1 (22.1)
Gateway National Recreation Area	35.1 (41.7)	50.6 (47.7)	83.1 (25.9)	98.2 (1.4)	75 (28.8)

Park	Bio7	Bio8	Bio9	Bio10	Bio11
Gauley River National Recreation Area	30.5 (24.5)	1.4 (1.2)	98.2 (2.3)	91.7 (10.6)	56.8 (64.6)
George Washington Birthplace National Monument	33.7 (4.5)	21.7 (4.7)	90.1 (13.6)	93.4 (15.3)	66.7 (44)
George Washington Carver National Monument	20.4 (24.5)	42.3 (62.7)	48.4 (63.8)	63.7 (43.8)	55.9 (59)
George Washington Memorial Parkway	24.3 (8.3)	17.5 (18.1)	77.2 (25.7)	82.1 (30.9)	68.6 (35.8)
Gettysburg National Military Park	10.9 (20.7)	22.4 (35.3)	70.9 (53.1)	78.2 (32.3)	60.3 (38.4)
Gila Cliff Dwellings National Monument	18.5 (26.5)	96.8 (9.7)	16.9 (12)	84.7 (23.3)	29.8 (33.6)
Glacier Bay National Park and Preserve	5.1 (6.3)	90.1 (5.1)	68.9 (17.8)	75.5 (30.1)	4.6 (10.6)
Glacier National Park	4.1 (4.1)	1.4 (0.9)	94.4 (14.6)	86.7 (30.9)	5.6 (8.5)
Glen Canyon National Recreation Area	9.9 (14.6)	37.8 (74.8)	56.7 (22.9)	71.7 (59.8)	13.4 (21.3)
Golden Gate National Recreation Area	13.9 (28.2)	19.4 (21.9)	13.8 (15.5)	53.3 (12)	38 (10.1)
Golden Spike National Historic Site	42.1 (24.2)	11.7 (12.4)	28.8 (72.8)	84.5 (40.7)	42.9 (35.9)
Grand Canyon National Park	5.2 (7.6)	34 (70.2)	3.7 (6.6)	60.5 (89.1)	24.4 (30.6)
Grand Portage National Monument	25.2 (52.7)	20.5 (27.3)	28.4 (48.1)	82.5 (30.8)	75.6 (19.3)
Grand Teton National Park	38.8 (26.1)	2.7 (4.7)	53.1 (90.3)	96.4 (9.7)	66.7 (51.5)
Grant-Kohrs Ranch National Historic Site	3.3 (2.4)	60.4 (24.6)	57.7 (29)	84.3 (34.6)	25.5 (21.6)
Great Basin National Park	28.5 (32.2)	31.8 (18.1)	42.8 (11.6)	82.6 (35.4)	33.8 (18.4)
Great Sand Dunes National Park and Preserve	76.2 (24.8)	14.7 (13.9)	66.2 (18)	97.1 (7.8)	29 (58.5)
Great Smoky Mountains National Park	41.2 (36.6)	63.8 (26)	45.5 (71.4)	98.5 (1.4)	56.1 (46.6)
Greenbelt Park	25.1 (11.7)	22.6 (19.6)	71.2 (32.2)	93 (15.3)	70.8 (34.2)
Guadalupe Mountains National Park	44.7 (61)	42.8 (19.9)	60.4 (35.6)	85.4 (28.9)	31.7 (12.1)
Guilford Courthouse National Military Park	38.4 (11.4)	35 (24.2)	64 (43.1)	97.6 (2)	84.1 (30.2)
Gulf Islands National Seashore	44.9 (58.2)	13.9 (11.7)	72.6 (35.6)	85.9 (14.7)	62.4 (47.9)
Hagerman Fossil Beds National Monument	27.5 (17.2)	21.6 (21.4)	24.7 (28.3)	71 (78.6)	44 (63.5)
Haleakala National Park	27.7 (33.3)	43.5 (13.9)	55.1 (76.1)	57.7 (20.5)	27.9 (42.3)
Harpers Ferry National Historical Park	26.4 (12.1)	21 (24.8)	51 (56.7)	66 (62.2)	58.4 (41.1)
Hawaii Volcanoes National Park	91.8 (22.3)	77.9 (54.6)	25.9 (18.9)	84.5 (38.8)	64 (38.7)
Herbert Hoover National Historic Site	9 (20.7)	47.8 (71.3)	30.6 (5.1)	55.1 (41)	77.6 (13.9)
Homestead National Monument of America	7.5 (19.5)	73.8 (39.4)	39.8 (44.3)	28.7 (28.9)	46.6 (22.9)
Hopewell Culture National Historical Park	42.4 (36.5)	28.4 (36.7)	100 (0)	93.3 (13.8)	74.3 (26.7)
Hopewell Furnace National Historic Site	27.5 (38.5)	13.2 (24.8)	75.8 (34.7)	85.5 (16.3)	81.6 (12.4)
Horseshoe Bend National Military Park	10.7 (21.9)	99.3 (2.2)	14.2 (34.3)	87.2 (16.8)	49.3 (64)
Hot Springs National Park	27 (24.7)	91 (19.7)	49.2 (47)	92.6 (15.1)	46.5 (43.5)
Hovenweep National Monument	19.8 (18.3)	53.4 (69.3)	52.7 (30.2)	73.8 (60)	13.5 (33.8)
Hubbell Trading Post National Historic Site	15.4 (19)	72.4 (20.6)	7.4 (12.6)	69.8 (67.8)	9.3 (17.4)
Indiana Dunes National Lakeshore	7.9 (20.7)	59.6 (47.6)	8.9 (17.4)	89.8 (24.7)	68.6 (27.6)
Isle Royale National Park	17 (34.9)	28.2 (29.5)	44.4 (40.7)	84.7 (22.2)	86.6 (24.8)
Jean Lafitte National Historical Park and Preserve	30.7 (52.7)	15.6 (22.8)	68 (21.5)	85.9 (25.8)	41.1 (62.1)
Jewel Cave National Monument	4.8 (4)	72.2 (53.2)	5 (6.7)	89.7 (18.3)	29.9 (16.6)
John Day Fossil Beds National Monument	2.5 (2.8)	4.2 (6.7)	13.5 (16.1)	45.9 (23.1)	18 (12.4)
John Muir National Historic Site	15.1 (30.1)	6.8 (10.2)	8.5 (7)	25.9 (5.3)	37.7 (21.5)

Park	Bio7	Bio8	Bio9	Bio10	Bio11
Johnstown Flood National Memorial	36 (33)	50.5 (20.5)	31.4 (9.3)	68.4 (4.7)	52.4 (38.1)
Joshua Tree National Park	25.4 (34.3)	41.5 (15.9)	55.6 (60.3)	72 (57.8)	19.7 (20.5)
Kalaupapa National Historical Park	77.3 (66)	52 (71.8)	45.2 (60.4)	58.2 (37.4)	25.8 (7.2)
Kaloko-Honokohau National Historical Park	86.8 (31.8)	60.1 (95.4)	38.7 (40.4)	84 (33)	53.7 (52.3)
Katmai National Park and Preserve	80.2 (12.6)	55.1 (13.1)	1.1 (0.2)	98.8 (3.6)	56.8 (44.9)
Kenai Fjords National Park	31 (25.2)	79.1 (22.9)	12.4 (23.8)	86.2 (25.8)	29.7 (32.3)
Kennesaw Mountain National Battlefield Park	17 (22.1)	65.2 (39.4)	10.9 (14.5)	90.7 (20.5)	61.2 (55.2)
Kings Mountain National Military Park	44.9 (22.1)	68.8 (41.8)	35.9 (21.2)	89.3 (18.3)	78.7 (28.4)
Klondike Gold Rush National Historical Park	6 (13.4)	63.8 (29.9)	97.1 (8.6)	82 (24.3)	6.5 (9.4)
Knife River Indian Villages National Historic Site	8.7 (23.1)	20.3 (7.4)	3.8 (4.6)	60.3 (37.3)	37.1 (6.1)
Kobuk Valley National Park	60.7 (50.6)	54.8 (11.9)	26.2 (38.2)	73.3 (16.3)	35.1 (19.1)
Lake Clark National Park and Preserve	43.8 (46.9)	78.3 (15.2)	6.5 (9.5)	99.6 (1.2)	22.1 (31.5)
Lake Mead National Recreation Area	7.7 (11.1)	49.3 (44.2)	10.2 (9.2)	59.2 (92)	26.2 (46.4)
Lake Meredith National Recreation Area	28.8 (38.3)	85.7 (28.2)	79.6 (19.5)	99.6 (1.1)	16.8 (19.7)
Lake Roosevelt National Recreation Area	2.1 (1.8)	10.9 (9.2)	2.3 (3.8)	54.2 (25.2)	19.3 (13.5)
Lassen Volcanic National Park	55.6 (51.3)	11.7 (12.4)	43.7 (25.7)	79.5 (48.1)	53.7 (56.9)
Lava Beds National Monument	35.8 (28.2)	11.7 (23.2)	26.2 (59.7)	59.6 (73.9)	45.3 (50.2)
Lewis and Clark National Historical Park	20.3 (36.3)	8.8 (15.1)	4 (8.6)	25.9 (4.7)	32.4 (29.3)
Lincoln Boyhood National Memorial	39.9 (26.7)	59.9 (14.5)	74.6 (6.1)	96.9 (4.6)	58.7 (39.8)
Little Bighorn Battlefield National Monument	1.8 (0.9)	44.4 (18.8)	80.6 (16.5)	66.1 (14.8)	6.8 (11.4)
Little River Canyon National Preserve	10.3 (24.1)	81 (40.1)	25.1 (31)	94.3 (8.7)	54.5 (46.9)
Lyndon B. Johnson National Historical Park	21.3 (34.1)	58.4 (31.7)	39.4 (65.7)	95.2 (5.3)	26.2 (26.3)
Mammoth Cave National Park	23.7 (9.8)	16.3 (34.4)	48.7 (38.8)	91.9 (7)	54.7 (40.9)
Manassas National Battlefield Park	25.7 (6.6)	8.4 (9)	77.8 (29.9)	75.1 (45)	66.4 (36.3)
Manzanar National Historic Site	37.6 (27.9)	12.6 (23)	15 (18.8)	65 (55.1)	16.2 (27.2)
Marsh-Billings-Rockefeller National Historical Park	56.1 (55.3)	23.6 (36.5)	43.8 (71.5)	53.8 (33.6)	87.4 (22.9)
Mary McLeod Bethune Council House National Historic Site	24.4 (9.5)	19.9 (19.5)	75.9 (23.5)	85 (31.3)	70.1 (34.4)
Mesa Verde National Park	13.6 (19.7)	43.1 (87.4)	38.2 (45.3)	85.9 (39.8)	14.7 (38.6)
Minute Man National Historical Park	43.8 (57.1)	30 (23.7)	83.2 (27.6)	69.6 (43.2)	79.3 (16)
Mississippi National River and Recreation Area	12.6 (32.8)	27.2 (30.6)	82.3 (14.2)	74.7 (35.3)	61.8 (10.4)
Missouri National Recreational River	8.5 (19.4)	16 (21.8)	56.5 (24.8)	58.2 (37)	53.4 (2.4)
Mojave National Preserve	9.6 (21.8)	50.7 (54.5)	33.8 (51.8)	63 (77.2)	11.2 (13.6)
Monocacy National Battlefield	23.8 (13.8)	15.2 (27.4)	64.2 (53.9)	67.8 (50.6)	58.5 (38.9)
Montezuma Castle National Monument	8.1 (16.3)	51.1 (74.2)	47.6 (42.7)	63.9 (91.5)	12.2 (31.9)
Moores Creek National Battlefield	7.1 (12.2)	85.7 (31.1)	51.5 (36)	82.4 (38.7)	58.5 (49.3)
Morristown National Historical Park	29 (40.5)	32.8 (28.9)	77.8 (32.5)	92.3 (16.3)	85.6 (5)
Mount Rainier National Park	28.3 (20.8)	27 (42.5)	32.9 (42.6)	34.9 (15)	21.9 (18.3)
Mount Rushmore National Memorial	1.4 (0.9)	79.3 (3)	2.4 (2.7)	88.4 (12.5)	29.6 (13.9)
Muir Woods National Monument	13.9 (28.2)	12.8 (17.3)	14.6 (16.6)	50.8 (10.2)	34.4 (9.9)
Natchez Trace Parkway	22.4 (24.6)	95.5 (5.6)	63.8 (21.8)	78 (34.9)	44.3 (59.3)

Park	Bio7	Bio8	Bio9	Bio10	Bio11
National Capital Parks-East	24.7 (9.5)	19.5 (18.5)	75.2 (25.7)	85.8 (32.5)	70.5 (33.2)
National Park of American Samoa	91.7 (4)	87 (3.8)	84.3 (12.7)	91.7 (4.9)	69.5 (47.9)
Natural Bridges National Monument	14.9 (14.6)	60.7 (67.2)	54.1 (29.4)	72.8 (60)	13.7 (20.1)
Navajo National Monument	16 (21.7)	40.3 (75.3)	13.3 (30.8)	65.1 (75.1)	21.5 (29.6)
New River Gorge National River	27 (30.9)	4.2 (7.5)	83.9 (17)	77.8 (40.7)	51 (66.9)
Nez Perce National Historical Park	2.6 (3.7)	28.5 (19.7)	25.3 (67.7)	85.5 (37.8)	10.6 (4.5)
Ninety Six National Historic Site	45.4 (10.7)	15.4 (19.9)	24.9 (19.7)	90.2 (23.1)	61 (48.1)
Niobrara National Scenic River	6.2 (9.8)	22.4 (19.3)	3.8 (4.2)	84.6 (20)	40.9 (10.2)
Noatak National Preserve	55.6 (47.9)	58.5 (8.9)	31.8 (44)	76.3 (12.4)	42.8 (8.3)
North Cascades National Park	7.6 (9)	34 (57.2)	13.8 (25)	57.7 (8.9)	28.9 (14)
Obed Wild and Scenic River	32.1 (4.8)	58.8 (6.6)	31.4 (48.2)	79.8 (24.1)	51.3 (45.2)
Ocmulgee National Monument	32.6 (29.5)	82.3 (44.7)	34.9 (36.5)	66.6 (24.3)	49.4 (53.3)
Olympic National Park	21.7 (43.5)	12.6 (13.5)	17.7 (27.5)	32.6 (23.5)	33.3 (32.5)
Oregon Caves National Monument	59.3 (54.4)	13.9 (32.8)	36.6 (61.7)	29.5 (43.8)	94.1 (6.2)
Organ Pipe Cactus National Monument	13.9 (25.8)	62.3 (39.2)	91 (18.9)	26.4 (35.9)	35.5 (18.4)
Ozark National Scenic Riverways	27.1 (16)	63 (31)	22.5 (37)	51 (47.8)	57.1 (49.9)
Padre Island National Seashore	6.5 (4.1)	31.8 (30.6)	51.1 (50.8)	43.8 (39)	16.4 (17.9)
Palo Alto Battlefield National Historical Park	9.2 (5.2)	56.7 (20.2)	31.8 (26.9)	26 (26.7)	27.3 (31.1)
Pea Ridge National Military Park	19.7 (12.3)	44.5 (1.5)	73.3 (27.7)	68.3 (44.3)	56.4 (55.5)
Pecos National Historical Park	18.1 (24.5)	98.1 (5.8)	52.4 (32.4)	99.6 (1.1)	32.8 (25.7)
Petersburg National Battlefield	49.2 (24)	58.2 (55)	75 (13.2)	82.4 (40.3)	67.2 (60.6)
Petrified Forest National Park	15.1 (18)	59.7 (12.4)	14.4 (27.7)	65.2 (70)	7.2 (13.5)
Petroglyph National Monument	18.9 (20.6)	92.2 (18.4)	6.8 (11.4)	93.7 (14.6)	29.5 (18.3)
Pictured Rocks National Lakeshore	13.8 (31.5)	76.8 (20)	77.2 (26)	67.7 (31)	86.7 (1.8)
Pinnacles National Monument	51.4 (47.5)	2.8 (4.2)	35.2 (31)	72.4 (30.7)	38.5 (13)
Pipe Spring National Monument	14.1 (19.8)	71.4 (39)	2.7 (3.6)	63.9 (74.1)	18.9 (25.6)
Pipestone National Monument	10.7 (29.1)	45.2 (44.1)	100 (0)	73.9 (42.9)	63.2 (8.3)
Piscataway Park	23.2 (7.3)	19.9 (14.4)	83.4 (13.7)	86.2 (33.7)	70.5 (33.1)
Point Reyes National Seashore	17.6 (31.3)	20.9 (22.1)	18.8 (23.8)	47.9 (5.3)	42.6 (2.7)
Port Chicago Naval Magazine National Memorial	21.5 (34.8)	7.8 (11.2)	6.5 (5.8)	13.2 (12)	43 (28.5)
Presidio of San Francisco	12 (26.8)	8.8 (17.1)	14.6 (16.6)	50.5 (13.4)	29.4 (13.7)
Prince William Forest Park	24.7 (7.3)	12.3 (5.3)	81.7 (19.5)	76.7 (40.1)	68 (33.7)
Pu'uhonua o Honaunau National Historical Park	92.9 (21.4)	64.5 (81.1)	34.5 (25.6)	87.6 (33)	53.3 (55.2)
Puukohola Heiau National Historic Site	81.3 (47.6)	79.7 (44.9)	42.4 (63.1)	75.5 (35)	59.4 (43.4)
Rainbow Bridge National Monument	14.5 (20.8)	25.6 (54.4)	31.1 (39.5)	66.5 (65.1)	22.3 (37.6)
Redwood National Park	82.2 (32.3)	45 (20.1)	28.7 (28.2)	62.4 (80.5)	97.2 (3.1)
Richmond National Battlefield Park	43.7 (9.6)	45.4 (35.5)	74.1 (8.6)	84.9 (38.6)	68.4 (53.4)
Rio Grande Wild and Scenic River	23.9 (42.1)	70 (17.9)	46.6 (29)	86.3 (25.3)	15.4 (2.5)
Rock Creek Park	25.1 (10.7)	19.9 (19.5)	74.4 (26.7)	85.8 (32.5)	70.1 (34.4)
Rocky Mountain National Park	15.3 (14.6)	6.8 (10.4)	40.1 (61)	79.9 (20.4)	27.1 (17.4)

Park	Bio7	Bio8	Bio9	Bio10	Bio11
Roosevelt-Vanderbilt Headquarters	37.8 (56)	71.6 (46)	39.8 (22.2)	82.9 (22.3)	91.1 (7.4)
Russell Cave National Monument	9.3 (21.2)	90.7 (21.1)	39.5 (45.3)	98.2 (1.4)	45.3 (54.2)
Sagamore Hill National Historic Site	33.2 (42.7)	83.1 (41)	78.8 (22.4)	98.1 (3.2)	72.4 (29.5)
Saguaro National Park	5.9 (14.5)	30.8 (1.2)	57.4 (32.4)	34.2 (65)	47.7 (39.3)
Saint Croix National Scenic Riverway	13 (32.8)	46 (57.4)	29.5 (21.2)	71.9 (36.1)	62.1 (14.8)
Saint-Gaudens National Historic Site	60.9 (61.3)	39.5 (56.6)	47 (61.3)	44.6 (52.6)	84.2 (20.2)
Salinas Pueblo Missions National Monument	34.9 (23.5)	55.1 (52.9)	15.8 (40.2)	94.3 (9.7)	42.7 (85.1)
San Antonio Missions National Historical Park	19.4 (27.8)	66.8 (58.9)	48.8 (62.6)	98.8 (2.4)	25.1 (34.4)
San Juan Island National Historical Park	25.8 (23)	13.2 (24.6)	4 (8.6)	66.7 (28.5)	38.3 (33)
Sand Creek Massacre National Historic Site	26 (29.7)	60.3 (2.3)	31.7 (10.3)	64.5 (19.8)	36.5 (24.4)
Santa Monica Mountains National Recreation Area	76.1 (53.2)	7.4 (7.2)	62 (48.9)	59.8 (87.9)	3.6 (6.3)
Saratoga National Historical Park	43.4 (60.5)	22.7 (37.4)	42.5 (15.1)	64.7 (19.1)	77.6 (34.6)
Saugus Iron Works National Historic Site	31.1 (36.8)	42.2 (9.3)	85.4 (24.1)	62.1 (49)	63.6 (36.7)
Scotts Bluff National Monument	10.3 (24.1)	93.8 (8.4)	3.7 (7.7)	95.7 (9.9)	21.3 (26.8)
Sequoia and Kings Canyon National Parks	46.4 (34.3)	12.1 (14.2)	9.4 (12.5)	53.3 (61)	20.9 (26.3)
Shenandoah National Park	32.9 (16.3)	77.3 (46.2)	37.5 (44.2)	70 (62.4)	63.4 (41.9)
Shiloh National Military Park	21.1 (24.1)	81.1 (43.2)	69.2 (14.3)	79.1 (33.2)	49.7 (48.7)
Sitka National Historical Park	2.7 (4.7)	86.6 (6.6)	74.6 (10.4)	69.6 (27.1)	8.5 (22.2)
Sleeping Bear Dunes National Lakeshore	16 (25.7)	8.2 (21.3)	60.2 (37.3)	68.3 (56.9)	75.2 (13.8)
Stones River National Battlefield	20.3 (16.4)	74.3 (25.8)	69.2 (23.3)	81.4 (28.4)	54 (43.1)
Sunset Crater Volcano National Monument	8.5 (13.9)	53.8 (80)	39.8 (30.5)	66 (80.6)	9.6 (19.3)
Tallgrass Prairie National Preserve	25.2 (9.6)	56 (28.5)	8.2 (11.2)	52.6 (43.3)	41.6 (28.6)
Theodore Roosevelt National Park	8.4 (18.2)	3.7 (1.9)	3.1 (3.1)	61.5 (31.9)	22.8 (15.1)
Thomas Stone National Historic Site	26.4 (10.8)	16.2 (6.8)	85 (12.7)	87.2 (31.8)	69.2 (36.4)
Timpanogos Cave National Monument	31.5 (27.2)	39.7 (35.5)	25.8 (70.1)	96.7 (8.7)	55.4 (49.9)
Timucuan Ecological and Historic Preserve	35 (44.3)	65.8 (22.4)	62.4 (8.1)	80.9 (21.6)	68.5 (40.2)
Tonto National Monument	13.4 (17.3)	89.6 (1.1)	58 (17)	44.5 (81)	33.9 (44)
Tumacacori National Historical Park	18.8 (24.8)	36.4 (18.4)	82.5 (17.5)	42.1 (65)	46.5 (31.8)
Tuzigoot National Monument	7.2 (12.4)	40.5 (78.7)	45.6 (35.3)	62.8 (91.3)	9.8 (26.1)
Upper Delaware National Scenic and Recreational River	35.6 (46.9)	58.4 (22.9)	35.4 (12.5)	92.2 (6.4)	95 (5.7)
Valley Forge National Historical Park	27.8 (36.1)	33.9 (30.9)	96 (6.6)	89.3 (20.9)	74.4 (25.5)
Valor in the Pacific National Monument	43.7 (90.3)	17.1 (30.4)	24.5 (53.9)	50.9 (47.9)	45.7 (55)
Vicksburg National Military Park	34.9 (15)	84.4 (20.8)	51 (26.7)	93.9 (10.1)	40.1 (61.9)
Virgin Islands National Park	11.9 (9.3)	62.4 (31.4)	22.6 (24.4)	38.3 (50.6)	26.6 (14.1)
Voyageurs National Park	13.7 (32.8)	19.5 (27.8)	3.8 (4.6)	59.2 (83.6)	59.2 (13.7)
Walnut Canyon National Monument	8.5 (13.9)	60.9 (69.6)	52.7 (37.2)	67.1 (80.9)	8.1 (17.4)
War In The Pacific National Historical Park	57.9 (26.2)	55.4 (14.3)	30.7 (25.4)	57.7 (29.1)	57.1 (22.5)
Washita Battlefield National Historic Site	22.3 (27.8)	94.4 (8.5)	58.7 (41.1)	89.8 (15.9)	10.2 (5.2)
Weir Farm National Historic Site	24.5 (36.7)	64 (74.2)	52.8 (14.1)	94.2 (11)	86.8 (7.4)
Whiskeytown National Recreation Area	54.3 (66.1)	10.5 (26)	13.6 (19.5)	1.9 (2.6)	54 (24.4)

Park	Bio7	Bio8	Bio9	Bio10	Bio11
White Sands National Monument	26.1 (18)	91.5 (18.3)	8.2 (13.3)	86.5 (28)	50.1 (53.5)
Whitman Mission National Historic Site	1.8 (1.4)	8.6 (5.5)	12.9 (20.7)	56.7 (14)	4.1 (5.1)
Wilson's Creek National Battlefield	21.8 (9.1)	67.7 (5.4)	59.3 (48.5)	62.1 (41)	54 (56.6)
Wind Cave National Park	1.7 (1.8)	86.1 (12)	3.1 (4.6)	87.5 (18.3)	29.8 (19.7)
Wolf Trap National Park for the Performing Arts	24.6 (7.3)	16.3 (23.3)	76.6 (24.8)	75.1 (44)	67.9 (35.1)
Wrangell - St Elias National Park and Preserve	3.4 (5.6)	45.6 (7.4)	54.7 (50.2)	56.6 (41.3)	15.3 (4)
Wright Brothers National Memorial	21.8 (13.6)	23.9 (11.9)	56.5 (42.7)	62.8 (59)	61.5 (58.2)
Wupatki National Monument	9.2 (12.7)	55 (74.6)	11.2 (15.7)	65 (82.6)	13.8 (31.9)
Yellowstone National Park	26.1 (28.6)	5.8 (7.5)	80.1 (45.2)	95.6 (8.5)	32.9 (17.7)
Yosemite National Park	42.6 (22.2)	9.4 (21.3)	50.1 (58.4)	37 (53)	22.9 (11.1)
Yucca House National Monument	15.1 (18.2)	44.3 (85.2)	34.1 (41.8)	81.8 (48.5)	14.9 (36.7)
Yukon-Charley Rivers National Preserve	12.4 (21)	10.8 (12.1)	12.6 (18.2)	25.7 (22.8)	16.9 (13.5)
Zion National Park	12.6 (23)	40.1 (40.2)	9.3 (11.2)	68.6 (73.6)	21.4 (19.4)
