# Supplementary material for: Climate Exposure of US National Parks in a New Era of Change
Source: PLoS One. 2014 Jul 2;9(7):e101302. doi: 10.1371/journal.pone.0101302 (PMC4079655; doi:10.1371/journal.pone.0101302)
Supplement: Appendix S7 — Mean percentile and maximum difference in percentile (in parentheses) for moving window standard deviations: annual precipitation (Bio12), precipitation of the wettest month (Bio13), precipitation of the driest month (Bio14), precipitation seasonality (Bio15), precipitation of the wettest quarter (Bio16), precipitation of the driest quarter (Bio17), precipitation of the warmest quarter (Bio18), and precipitation of the coldest quarter (Bio19). (PDF) [file pone.0101302.s007.pdf]

Appendix S7. Mean percentile and maximum difference in percentile (in parentheses) for moving window standard deviations: annual precipitation (Bio12), precipitation of the wettest month (Bio13), precipitation of the driest month (Bio14), precipitation seasonality (Bio15), precipitation of the wettest quarter (Bio16), precipitation of the driest quarter (Bio17), precipitation of the warmest quarter (Bio18), and precipitation of the coldest quarter (Bio19).

Park	Bio12	Bio13	Bio14	Bio15	Bio16	Bio17	Bio18	Bio19
Abraham Lincoln Birthplace National Historical Park	64.4 (44.5)	79.9 (20.1)	76.4 (21.2)	69.1 (68.5)	70.1 (15.8)	98.8 (2.4)	20.1 (38.6)	11.8 (16.4)
Acadia National Park	92.3 (17.4)	61.7 (24)	84.1 (34.4)	83.2 (9.6)	95.8 (2.6)	84.6 (20.9)	98.6 (2.9)	24.4 (50.1)
Agate Fossil Beds National Monument	62.8 (16.9)	71 (19.8)	62.5 (11.1)	6.2 (6.8)	64.2 (30.7)	50.8 (52.4)	73 (32.9)	16.9 (16.4)
Ala Kahakai National Historic Trail	28.9 (23.9)	37.7 (43.2)	1.1 (0.2)	52.5 (84.5)	50.9 (41.7)	1.7 (1.8)	12 (24.2)	49.8 (21.9)
Alagnak Wild River	86.6 (27.2)	85.6 (21)	26.8 (28.1)	76.1 (9.8)	54.3 (28.8)	42.6 (11.4)	42.3 (33.3)	78.2 (44.1)
Alibates Flint Quarries National Monument	48.4 (50.9)	57.7 (51.2)	60.7 (62.1)	56.3 (49.2)	51.8 (54.6)	76.5 (35.6)	88.4 (5.9)	28.8 (30.9)
Allegheny Portage Railroad National Historic Site	87.8 (20.7)	52.1 (14.1)	81 (25.9)	29 (9.7)	61.4 (7)	12.5 (22.5)	50.4 (33.8)	40.7 (52.4)
American Memorial Park	42 (27.4)	60.4 (36.3)	44.9 (47)	9.5 (17.2)	45.1 (25)	43 (30)	55.6 (21.1)	26.9 (18.9)
Amistad National Recreation Area	70.5 (34.8)	12.3 (27.9)	15.8 (10.6)	49 (73.6)	27.4 (56.9)	29.5 (29.4)	43.2 (53.1)	39.7 (57.1)
Anacostia Park	61.1 (45.4)	20.6 (34.5)	98.5 (1.4)	60.4 (26.1)	54 (48.6)	55.3 (38.1)	17.9 (16.3)	16.9 (17.4)
Aniakchak National Monument and Preserve	92.6 (22.3)	84.7 (26.2)	57.8 (13.4)	88 (10.4)	95.4 (9.5)	74.2 (18.7)	23 (26.5)	71.4 (41.5)
Antietam National Battlefield	94.6 (1.7)	39.2 (56.9)	98.3 (1.8)	37.5 (37.5)	83.1 (19.3)	87 (13.8)	57.4 (14.2)	34.7 (29)
Apostle Islands National Lakeshore	30.9 (11.1)	26.1 (4.5)	76.6 (52)	33 (17.8)	54.2 (36.9)	81.3 (42.7)	48.2 (36.4)	65.9 (62.9)
Appalachian National Scenic Trail	97.1 (2.8)	64 (14.5)	94.9 (11.1)	16.2 (12.9)	97.6 (3.8)	91.3 (11.1)	96.6 (7.8)	20.1 (31)
Appomattox Court House National Historical Park	68 (32.9)	62.1 (33.7)	84.9 (11.8)	50.3 (52.8)	72.8 (35.3)	39.8 (27.7)	20.4 (21.9)	60.3 (62.7)
Arches National Park	28.4 (20.7)	25.7 (42.1)	64.2 (38.8)	59.9 (24.9)	42 (47.7)	14.8 (19.8)	8.3 (12.8)	60.9 (30.9)
Arkansas Post National Memorial	90 (19)	64.8 (30.3)	71.8 (59.2)	33.6 (47.8)	70.7 (31.6)	59.7 (48.9)	15.9 (38.7)	18.5 (24.8)
Assateague Island National Seashore	73.7 (33.7)	47.2 (63.5)	11.8 (18.8)	35.7 (54.4)	90.1 (19.5)	83.5 (21.9)	82 (20.6)	28.4 (31.3)
Aztec Ruins National Monument	13.2 (17.1)	43.5 (60.1)	30.4 (31.2)	63.3 (17.8)	16.8 (23.2)	36 (27.2)	60 (89.5)	78.3 (25.7)
Badlands National Park	69.9 (25.6)	49 (51.8)	87.9 (15.9)	10.6 (23.8)	73.3 (5.8)	86.9 (22)	64.4 (60.4)	42.9 (16.2)
Baltimore-Washington National Parkway	59.7 (35.3)	20.7 (33.3)	98.2 (1.3)	47.1 (37.3)	54 (34.4)	59.1 (41)	12.9 (16.8)	18.8 (15.9)
Bandelier National Monument	56 (42.1)	99 (2.9)	23.2 (66.5)	48.7 (32.3)	90.4 (17.2)	49.6 (74.2)	97.1 (1.5)	84.8 (19)
Bent's Old Fort National Historic Site	55.8 (12)	68.4 (20.7)	65.6 (31.5)	58.5 (36.7)	75.7 (23.1)	50.3 (23.1)	75.1 (4.4)	2.5 (2.8)
Bering Land Bridge National Preserve	14.4 (11.7)	25.4 (21.7)	80 (12)	64.7 (27.5)	63.8 (29.7)	97.6 (5.4)	53.1 (21)	90.5 (15.7)
Big Bend National Park	52.7 (54.3)	10.4 (24.2)	71.9 (56.1)	68.5 (24.3)	40.7 (57.9)	71.8 (3.2)	81.9 (35)	15.2 (33.9)
Big Cypress National Preserve	70.7 (21.3)	27.7 (18.4)	45.2 (65.9)	29.2 (40.2)	100 (0)	53.3 (83.1)	64.5 (11.9)	57.5 (84.3)
Big Hole National Battlefield	63.2 (42.1)	37 (47.9)	68.4 (27.1)	44.9 (43.9)	73.3 (29.3)	45.8 (18.7)	73.8 (68)	80.1 (14.9)
Big South Fork National River and Recreation Area	98.9 (0.2)	65.6 (30.7)	91.5 (7)	75.4 (14.7)	95.6 (7.5)	98.9 (2.4)	39.6 (45.3)	18.4 (16.5)
Big Thicket National Preserve	60.8 (34.5)	36 (24.4)	57.9 (27.9)	44.1 (79.2)	67.5 (33.8)	65.5 (39.8)	79 (27.9)	49.7 (76.8)
Bighorn Canyon National Recreation Area	70.1 (63.1)	63.1 (59.2)	67.7 (50.1)	24.4 (24.9)	70.6 (50.6)	23.3 (14.2)	63.1 (51.6)	13.3 (13.8)
Biscayne National Park	54.5 (23.9)	11.6 (16.5)	54.1 (43)	17.5 (27.2)	40 (44.8)	58.1 (80.7)	79.9 (32.7)	69.4 (76.7)
Black Canyon Of The Gunnison National Park	37.2 (30)	65.8 (23.9)	49 (43.6)	93 (17.5)	99.4 (1.9)	15.1 (9.8)	55.5 (45.2)	74 (55.2)
Blue Ridge Parkway	92.3 (12)	85.1 (21.9)	53.5 (23.3)	27.2 (10.2)	93.5 (14.1)	30.5 (7.8)	84.2 (10.8)	16.7 (18.8)
Bluestone National Scenic River	90.7 (13.2)	91.7 (15.4)	90.9 (20.3)	61.4 (86.9)	81.1 (16.6)	80.2 (30.2)	62.9 (14)	13.8 (13.6)
Booker T. Washington National Monument	92.9 (6.7)	28.4 (8.3)	68.9 (22.7)	19.4 (10.4)	87.1 (16.5)	58.5 (8.1)	58.3 (24.1)	59 (69.2)
Boston Harbor Islands National Recreation Area	47 (17.3)	56.8 (22.9)	88.1 (27.2)	79 (40.3)	70.9 (21.8)	94 (15.5)	85.2 (31.1)	41.2 (25.1)

Park	Bio12	Bio13	Bio14	Bio15	Bio16	Bio17	Bio18	Bio19
Bryce Canyon National Park	67.4 (40.2)	69.9 (43.9)	7.6 (14.7)	47.1 (55.2)	84 (24.7)	23.5 (5.1)	76.2 (63.1)	68.4 (19.2)
Buck Island Reef National Monument	61.3 (33.7)	15.7 (25.4)	93.8 (11.8)	18.2 (49.5)	53.1 (39.3)	15.8 (21.4)	49.1 (22.6)	22.2 (30.1)
Buffalo National River	43.2 (53.6)	57.1 (56.6)	64.3 (49.1)	44.7 (33.4)	47.3 (60.6)	5.5 (7.3)	14.5 (25)	17.1 (48.4)
Cabrillo National Monument	55 (7.4)	87.1 (20.7)	18.8 (36.7)	92.7 (15.1)	86.4 (14.8)	4.4 (8.6)	87.9 (20.3)	89.9 (11.1)
Canaveral National Seashore	18.2 (5.8)	25.8 (50.3)	62.6 (22.5)	57 (18.2)	27.4 (16.9)	16.9 (23.5)	26.2 (28.7)	35.6 (63.6)
Canyon De Chelly National Monument	17 (12.5)	31.1 (13.7)	21.4 (32.3)	27.1 (34.1)	22.6 (21.4)	11 (15.9)	37.8 (34.1)	88.6 (24.2)
Canyonlands National Park	29.1 (14.5)	35 (54.7)	49.7 (32.5)	69.1 (12.3)	40.8 (72.4)	30.7 (37.5)	8.3 (11.1)	69.1 (23.2)
Cape Cod National Seashore	46 (15)	57.2 (39.6)	70.3 (17.7)	76.3 (43.8)	62.2 (12)	74.2 (30.8)	84.9 (8.6)	35.5 (25.8)
Cape Hatteras National Seashore	91.9 (3.7)	69.2 (67.6)	81.7 (19)	41.9 (15)	85.9 (34.4)	70.1 (19.2)	38.5 (23.5)	58.6 (63.9)
Cape Krusenstern National Monument	26.2 (8.4)	48.3 (55.3)	90.9 (15.2)	37.1 (34.7)	67.6 (10.5)	90.2 (16.1)	61.1 (13.8)	89 (17.2)
Cape Lookout National Seashore	91 (1)	79.8 (29.6)	80.4 (23.3)	60.7 (12.4)	73.8 (14.2)	78.9 (18.2)	46.2 (18.1)	46.4 (43.1)
Capitol Hill Parks	61.8 (45.2)	21 (35.5)	98.1 (2.6)	60.7 (26.1)	54 (48.6)	55.3 (38.1)	19.3 (13.9)	17.2 (17.4)
Capitol Reef National Park	52.7 (39.8)	61.9 (64.3)	28.2 (14.2)	50.8 (22.7)	42.4 (57.6)	9.7 (11.1)	15.1 (37.8)	73.5 (18.2)
Capulin Volcano National Monument	31.8 (41.6)	50.6 (48.9)	40.1 (39.9)	29.4 (40.4)	39.4 (61.7)	4.2 (6.7)	48.7 (50.7)	20.5 (48.5)
Carl Sandburg Home National Historic Site	76.4 (28.7)	88.9 (15.4)	27.5 (12.6)	41.4 (15.8)	94.9 (2.1)	24.1 (25.3)	91.9 (4.2)	14.7 (19.7)
Carlsbad Caverns National Park	78.6 (47.9)	37.6 (41.7)	87.9 (4.3)	42.7 (31.3)	64.7 (21.9)	82.5 (19.6)	72.9 (37.2)	56.7 (42.9)
Casa Grande Ruins National Monument	31.8 (87)	26 (44.3)	11.6 (13.7)	56.1 (37.4)	28 (42.6)	1.1 (0.2)	22.8 (65.3)	50.8 (11.3)
Castillo De San Marcos National Monument	5.9 (14.5)	67.6 (54.1)	46.8 (16.6)	93.9 (9.6)	42.1 (66.1)	30.8 (46.9)	2.4 (3.8)	34.7 (39.5)
Catoctin Mountain Park	85.6 (13.8)	44.1 (59.6)	96.2 (7.8)	30 (35.2)	62.4 (22.3)	80.6 (30.5)	46.9 (32.2)	36.4 (34.8)
Cedar Breaks National Monument	92.4 (8.7)	57.4 (37.4)	28.3 (70.3)	53.1 (40.8)	75.3 (39.2)	61.2 (28.8)	84 (27.6)	53.8 (49.2)
Chaco Culture National Historical Park	26.4 (55.6)	61.1 (86.4)	22 (28.6)	55.9 (63.3)	39 (58.5)	19.7 (20.6)	60.6 (83.4)	76.9 (23.5)
Channel Islands National Park	55.1 (30.6)	70 (62.4)	25.8 (42.3)	59.3 (25.6)	50.1 (39)	8.7 (6.8)	27 (31.2)	51.8 (20.4)
Charles Pinckney National Historic Site	21.6 (23.3)	5.9 (8.7)	63 (36.4)	26.6 (30.8)	5.5 (7.5)	10.5 (12.4)	2.6 (3.8)	53.3 (87.4)
Chattahoochee River National Recreation Area	77.2 (26.2)	53.4 (61.1)	42.2 (32.8)	32.9 (53.4)	85.4 (18.6)	27.4 (6.3)	54.9 (56)	1.4 (0.9)
Chesapeake and Ohio Canal National Historical Park	89.2 (0.2)	19.7 (20.9)	98.3 (1.8)	20.9 (23.5)	69.4 (28)	72.3 (34.8)	41.8 (20.4)	27 (34.2)
Chickamauga and Chattanooga National Military Park	82.9 (17.1)	25.3 (54.6)	82.9 (33)	21.9 (28.2)	59.1 (31.9)	60.1 (61)	43.7 (30.1)	10.9 (13.2)
Chickasaw National Recreation Area	10.9 (17.4)	5.4 (9.5)	59.4 (36.2)	8.1 (14.3)	19.9 (25.8)	38.6 (38.1)	76.5 (6.4)	54.7 (87)
Chiricahua National Monument	35.8 (79.8)	49.2 (48.3)	65.2 (40.1)	78 (23.1)	51.3 (40.1)	38.5 (55.3)	14.5 (25)	70.8 (24.2)
City Of Rocks National Reserve	69.1 (37.9)	83.6 (27.2)	20.3 (56.8)	64.6 (48.6)	98 (3.2)	20.3 (28.1)	8.6 (11.9)	70.8 (26.6)
Colonial National Historical Park	74.1 (21.3)	84.5 (13.3)	83.9 (11.3)	91.5 (22.2)	97.8 (2.6)	71.4 (24.6)	93.3 (6.7)	58.5 (58.6)
Colorado National Monument	62.1 (3.2)	26.9 (29.2)	76.9 (25.5)	56.3 (10.3)	78.5 (29.1)	34 (12.6)	23.8 (22.9)	58.8 (35.8)
Congaree National Park	41.4 (11.4)	15.6 (25.7)	83.5 (20)	38.1 (14.7)	42.9 (37.4)	43.6 (11.6)	63.1 (70.7)	32.3 (35.5)
Coronado National Memorial	48.1 (75.7)	68.2 (31.5)	33.6 (27.2)	82 (13.4)	39.9 (21.6)	41 (64.2)	68.1 (46.7)	57.6 (18.4)
Cowpens National Battlefield	65.2 (31)	58.2 (44.2)	64.5 (48.6)	56.3 (40.9)	75.2 (22.8)	19.5 (26.1)	70.5 (27.1)	4.4 (5.6)
Crater Lake National Park	70.3 (62.3)	25.1 (14.7)	84.5 (16.8)	8.4 (15.3)	53.1 (29)	67.6 (27.1)	36.8 (29.6)	55.5 (25.9)
Craters Of The Moon National Monument and Preserve	63.4 (48.6)	48.8 (28.2)	21.7 (30.5)	4.1 (7.7)	71.7 (21.3)	15.5 (26.8)	25.4 (49.1)	76.2 (34.7)
Cumberland Gap National Historical Park	98.9 (0.2)	2.5 (4.4)	82.4 (20.2)	54.4 (50.9)	98.9 (0.2)	99.6 (1.2)	44.3 (44.8)	12.6 (27.9)
Cumberland Island National Seashore	31.8 (26.7)	59.6 (78)	51.4 (14.6)	93.7 (5.1)	50 (55.5)	55.6 (33.3)	34.5 (27.6)	48.9 (63.5)
Curecanti National Recreation Area	42 (18.5)	76.9 (24.3)	63.2 (28)	95.8 (12.6)	94.6 (12.2)	11.5 (2.2)	37.6 (36.8)	89.6 (19)
Cuyahoga Valley National Park	83.5 (29.1)	41.3 (50)	82.4 (22.9)	10.7 (20.3)	66 (20.8)	46.3 (17.8)	9.4 (16.4)	59.1 (28.5)

Park	Bio12	Bio13	Bio14	Bio15	Bio16	Bio17	Bio18	Bio19
Death Valley National Park	70.7 (32.4)	85 (31.1)	71.2 (39.3)	100 (0)	94.4 (12.6)	47.5 (26)	53 (37.9)	96.3 (6.6)
Delaware Water Gap National Recreation Area	69.8 (30.2)	64.1 (47.9)	44.1 (59.1)	16.3 (24.8)	85.1 (16.6)	86.2 (28.2)	75.1 (34.1)	48.2 (25.3)
Denali National Park and Preserve	5.2 (4.4)	58.6 (25.6)	50.1 (19.6)	85.5 (14.8)	40.2 (27.9)	57.2 (54.2)	38.2 (43.8)	66.4 (61.2)
Devils Postpile National Monument	52.2 (48.4)	9.6 (4.6)	6.8 (8.7)	23.5 (16.5)	16.2 (12.9)	3 (5.7)	31.3 (63.6)	20.3 (7.9)
Devils Tower National Monument	51.8 (15.1)	76.9 (24.5)	16.9 (39.1)	29.6 (31.4)	55 (44.4)	65.9 (60.9)	26.8 (20.9)	48.1 (62.3)
Dinosaur National Monument	63.4 (59.3)	63.3 (29.2)	66.8 (14.7)	31.6 (35.4)	68.1 (30.3)	51.5 (67.9)	7.8 (8.9)	41.3 (28.4)
Dry Tortugas National Park	51.1 (44.1)	59.9 (37.9)	39.5 (29)	76.6 (23.5)	92.1 (12)	17.8 (9.4)	77.3 (22.6)	47.9 (85.8)
Ebey's Landing National Historical Reserve	53.6 (56.7)	83.1 (3.8)	11.6 (10.4)	79.2 (34)	84.5 (17.5)	27.7 (36.3)	41.5 (15.9)	49.9 (41.5)
Effigy Mounds National Monument	76.1 (17.5)	99.6 (1.2)	51.8 (44.6)	54.4 (61.6)	100 (0)	35.4 (49.7)	99.6 (1.1)	53.3 (26.9)
Eisenhower National Historic Site	79.7 (22.4)	44.9 (58.6)	95.5 (8.7)	35.2 (38.7)	47.9 (24.6)	69.3 (52.7)	31.8 (32.4)	37.5 (37.5)
El Malpais National Monument	38.9 (94.2)	76.6 (66)	28.8 (83.4)	65 (16.4)	57.9 (95.6)	50.5 (40.2)	78.9 (41.8)	70 (16.7)
El Morro National Monument	35.4 (88.6)	76.5 (43.5)	23.1 (65.2)	68.5 (2.9)	52.2 (95.1)	17.3 (22.9)	67.3 (57.1)	65.2 (12.6)
Eugene O'Neill National Historic Site	32.1 (52.2)	14.1 (34)	46.9 (69.8)	59 (17.4)	6 (7.7)	64.5 (12.2)	29.4 (32.6)	29 (49.8)
Everglades National Park	58.2 (28)	20.9 (16.6)	52.5 (52.7)	24.5 (33.5)	97.6 (5.4)	56.4 (80.1)	75.1 (15.3)	65.1 (86.4)
Fire Island National Seashore	55.4 (15.5)	79.7 (14.4)	86.9 (25.8)	24.3 (52.2)	69.2 (32.3)	80.3 (21.8)	97.6 (3.9)	22.7 (22.7)
Florissant Fossil Beds National Monument	88.4 (7.1)	76.3 (7.7)	50.3 (25.4)	3.4 (5.7)	54.3 (43.9)	55.3 (26.4)	51.2 (40.8)	22.5 (15.9)
Fort Bowie National Historic Site	45.6 (73.6)	53.6 (55.3)	67.2 (35.3)	79.7 (16.1)	70.3 (29.5)	39.9 (62.1)	22.2 (30.6)	66 (21.8)
Fort Caroline National Memorial	29.3 (27)	92.9 (18.1)	41.1 (13.6)	95.6 (5.1)	59.4 (62.1)	52.1 (44.2)	30.6 (47.4)	38 (50.7)
Fort Davis National Historic Site	34.9 (50.9)	14.7 (22.7)	75.3 (14.3)	48.6 (50.5)	28.7 (58.6)	80.3 (16.2)	59.3 (63.6)	31.3 (34.5)
Fort Donelson National Battlefield	22.7 (47.8)	22.7 (38.4)	88.6 (32)	90.8 (23.1)	20.2 (35.8)	84 (17.3)	46.2 (13.5)	17.4 (22.2)
Fort Frederica National Monument	30.9 (28.3)	35.6 (66.6)	75.5 (3.5)	98.3 (3.9)	39.5 (47.9)	63.7 (14.1)	28.9 (14.7)	55.6 (72.5)
Fort Laramie National Historic Site	64 (34.3)	58 (0.4)	55.2 (35.1)	17 (12.5)	28.6 (41.1)	96.5 (2.7)	39.5 (38.3)	40 (4.6)
Fort Larned National Historic Site	19.8 (22.4)	67.5 (52.8)	39.7 (27.5)	15.3 (12.9)	39.8 (41.1)	34.3 (32)	45.8 (39.1)	51.8 (21.9)
Fort Matanzas National Monument	7.2 (18.3)	66.1 (56.6)	49.3 (9.1)	89.6 (20.4)	32.6 (59.8)	26.1 (38.5)	1.7 (1.8)	35.5 (42.8)
Fort Moultrie National Monument	22 (24.5)	5.1 (6.3)	62.1 (34.4)	28.1 (33.2)	6.2 (7.5)	12.5 (13.2)	2.6 (3.8)	50.6 (82.6)
Fort Necessity National Battlefield	64.4 (56.2)	11.1 (20)	76.1 (33)	9.5 (5.1)	28.9 (37)	33.7 (60.9)	14.1 (15.8)	57.7 (73.1)
Fort Point National Historic Site	35.4 (50)	33.6 (66.5)	5.4 (12.2)	55 (35.4)	9.8 (13)	44.1 (81)	17.9 (29.9)	42.2 (68.6)
Fort Pulaski National Monument	38.5 (16.7)	10.7 (20.5)	57.4 (30.5)	49 (58.5)	16.4 (10.5)	68.8 (5.3)	25.6 (23.3)	56.4 (94.7)
Fort Raleigh National Historic Site	92.6 (3.9)	62.1 (68)	78.5 (28.9)	38.2 (38.1)	84.3 (32.1)	65.5 (22.1)	31.1 (39.6)	64.6 (62)
Fort Sumter National Monument	22.3 (23.6)	5.1 (5.1)	61.8 (48.5)	23.8 (34.6)	6.2 (7.5)	14.6 (13.9)	3 (2.9)	51.7 (81.7)
Fort Union National Monument	76.4 (16.3)	71.5 (58.8)	41.7 (34.7)	23.8 (39.3)	41.6 (21.4)	84.7 (14.4)	51.5 (6.9)	83.3 (25)
Fort Union Trading Post National Historic Site	13.1 (20.9)	30.9 (53.6)	67.1 (4.8)	82.6 (24.6)	17.9 (30)	82.7 (21.6)	24.3 (17.5)	97 (4.8)
Fort Vancouver National Historic Site	79.1 (36.2)	52.3 (22.1)	37.9 (24.1)	51.3 (59.9)	80 (20.7)	55.8 (26.3)	49.6 (13.9)	58.4 (67.7)
Fort Washington Park	59.2 (57.3)	20.3 (33.7)	97 (2.9)	62.7 (28)	49.7 (62.5)	52.8 (36.6)	23 (18.3)	16.2 (15.2)
Fossil Butte National Monument	56.8 (65.4)	81 (11.2)	12 (13.3)	30.8 (30.7)	75.2 (18.1)	12.8 (17.1)	57.6 (85.9)	39.3 (38)
Frederick Douglass National Historic Site	60.7 (47.9)	20.6 (35.7)	98.2 (1.4)	61.1 (27.2)	53.4 (51.9)	53.1 (38.8)	19.6 (13.9)	16.9 (17.4)
Fredericksburg and Spotsylvania National Military Park	80 (28.4)	32.4 (27.5)	91.6 (4.4)	44.9 (27.1)	62.7 (51)	84.5 (14.4)	40.8 (41.2)	56.3 (64.7)
Friendship Hill National Historic Site	52.7 (32.5)	13.7 (22.9)	49.3 (40.4)	11.1 (5.7)	20.4 (35)	35.3 (68.2)	27.8 (14)	66 (51.1)
Gates Of The Arctic National Park and Preserve	18.1 (17.3)	6.3 (13.4)	85.6 (22.4)	2.4 (2)	13.7 (7.6)	60.7 (52.7)	13.9 (4.7)	73.8 (12.2)
Gateway National Recreation Area	60.1 (5.8)	90.3 (11.8)	99.3 (1.2)	67.8 (21.1)	47.2 (50.5)	98.1 (5.8)	67.3 (31.9)	17.8 (31.6)

Park	Bio12	Bio13	Bio14	Bio15	Bio16	Bio17	Bio18	Bio19
Gauley River National Recreation Area	74.7 (13.8)	47.2 (31.7)	92.5 (11.2)	63.6 (60.1)	91.3 (16)	72.8 (36.3)	42.8 (55.5)	13.3 (9.3)
George Washington Birthplace National Monument	70.3 (37.3)	36.6 (44.2)	90.4 (8.6)	52.4 (26.9)	67.8 (41.8)	64.8 (27.8)	38.5 (18.7)	53.3 (67.3)
George Washington Carver National Monument	59.4 (56.7)	62.1 (42.7)	46.2 (27.9)	81.1 (44)	79.4 (8.1)	40.9 (37.7)	39 (37.4)	46 (43.4)
George Washington Memorial Parkway	75.7 (29.3)	20.2 (35.7)	97.7 (3.8)	62.5 (17)	61.8 (39.4)	62.4 (29)	29.3 (18.4)	19 (19.4)
Gettysburg National Military Park	81.7 (17.5)	45.6 (58.4)	95.5 (8.7)	36.7 (37.3)	50.1 (23.1)	68.3 (52.7)	30.4 (31.7)	37.9 (37.5)
Gila Cliff Dwellings National Monument	38.2 (50.2)	91.6 (5)	41 (22.5)	78.9 (3)	94.5 (4.3)	50.9 (31.7)	95.6 (2.8)	82 (6.9)
Glacier Bay National Park and Preserve	43.3 (66.9)	37.5 (35.5)	85.1 (14.1)	74.5 (15.3)	48.1 (61.5)	90 (18.3)	20.4 (20)	76 (41.3)
Glacier National Park	53.3 (37.1)	99.2 (1.2)	27.5 (38.4)	51.3 (44.2)	54.7 (32.4)	12.8 (28.1)	75.3 (50)	22.9 (33.9)
Glen Canyon National Recreation Area	43.4 (50.4)	56.5 (43.9)	33.6 (12.7)	56.5 (7.2)	53.5 (57.5)	14.2 (26.7)	22 (46.8)	75.7 (28.3)
Golden Gate National Recreation Area	41 (39.3)	37.7 (71.6)	11.6 (23)	51.1 (32)	11.4 (18.8)	41.7 (73.7)	14.9 (25.3)	42.3 (70.7)
Golden Spike National Historic Site	62.3 (20.7)	73.8 (21.1)	39.5 (41.8)	16.6 (19.8)	89.8 (12.3)	24.5 (63.1)	30 (67)	55.6 (33)
Grand Canyon National Park	56.7 (9)	84.8 (24.1)	9.1 (24.3)	60.2 (79.2)	74.6 (18.7)	37.7 (70.8)	88.3 (34)	72.2 (17)
Grand Portage National Monument	42.5 (33.9)	16.5 (27.2)	61.6 (54.8)	58.1 (45.5)	26.9 (30)	63.1 (16.8)	54.5 (61.2)	38.4 (50)
Grand Teton National Park	47.8 (54.8)	69.9 (30)	26.2 (61.2)	46 (47.6)	72.9 (46.2)	13.9 (26.5)	62.6 (44.7)	51.5 (77.3)
Grant-Kohrs Ranch National Historic Site	34.2 (46)	18.6 (31.6)	92.6 (15.1)	29.1 (42.3)	42 (48.2)	30.8 (15.7)	64.1 (87.9)	84.6 (38.6)
Great Basin National Park	52.7 (42.1)	71.4 (14.6)	11.7 (15.6)	5.2 (8.5)	78 (5.9)	29 (29.3)	50.5 (49.9)	43.2 (10.5)
Great Sand Dunes National Park and Preserve	46.5 (11.5)	91.5 (15.9)	60.2 (0)	24.5 (49.9)	45.7 (46)	68.9 (32.6)	36.1 (41.6)	19.6 (7.6)
Great Smoky Mountains National Park	89.9 (16.5)	44.9 (7.7)	61 (12)	26.8 (52)	70.5 (27.3)	91.9 (16.1)	53.5 (43.6)	5.2 (4.4)
Greenbelt Park	58.7 (41.1)	21.3 (34.5)	98.2 (1.3)	53.8 (28.1)	54.3 (35.4)	56.2 (41.3)	13.3 (19)	18.4 (19.4)
Guadalupe Mountains National Park	78.6 (46.8)	48.7 (45.7)	92.5 (2.6)	50.2 (50.5)	78.6 (27.1)	77.4 (10.1)	73.9 (30.9)	57.3 (59.5)
Guilford Courthouse National Military Park	86 (8.8)	67.9 (34.8)	38.2 (75.2)	36.5 (47.9)	97.3 (4.9)	34.5 (57.1)	77.8 (22.5)	46.1 (65.7)
Gulf Islands National Seashore	41.4 (49.4)	74.2 (46.1)	28.3 (28.6)	82.7 (27)	97 (6.8)	24.3 (22.2)	100 (0)	51.8 (76.6)
Hagerman Fossil Beds National Monument	59 (26.9)	39.3 (26.1)	15.8 (31.6)	6.9 (17.4)	38.1 (24.8)	5 (11)	6.5 (13.4)	42.4 (42.7)
Haleakala National Park	43.4 (37.4)	89.1 (4.5)	1.4 (0.9)	99.2 (2.4)	40.7 (23.3)	6 (12.4)	13.8 (36.3)	70.2 (22.5)
Harpers Ferry National Historical Park	96.8 (1.9)	40.4 (61.8)	98.9 (0.2)	44.7 (36.8)	91.2 (17.3)	87.7 (11.5)	61.6 (26.8)	34.9 (25.6)
Hawaii Volcanoes National Park	32.8 (26.1)	27.1 (36.5)	7.3 (14.7)	36.5 (91)	49.6 (38.9)	6.4 (4.4)	16.5 (11.5)	45.8 (22.1)
Herbert Hoover National Historic Site	100 (0)	100 (0)	29.1 (25.2)	28.1 (25.2)	98.1 (5.8)	36.2 (8.9)	97.4 (7.8)	30.2 (7.7)
Homestead National Monument of America	71.2 (35.5)	37.1 (24.2)	45 (25.5)	19.7 (29.1)	59.3 (53.7)	18.9 (21.7)	52 (13.3)	37.4 (14.3)
Hopewell Culture National Historical Park	78.8 (42.9)	46.7 (54)	71.6 (23)	36.5 (22.6)	74.2 (40.8)	65.2 (71.9)	4.9 (3.2)	37.8 (60.8)
Hopewell Furnace National Historic Site	87 (25)	90.8 (13.9)	92.5 (10.6)	51.2 (22.4)	98 (3.2)	76 (35.7)	93.8 (10.6)	35.2 (52.5)
Horseshoe Bend National Military Park	80.2 (33.5)	26.6 (47.4)	36.5 (41.2)	32.6 (21.3)	55.3 (76.6)	44.7 (72.4)	91 (14.4)	1.1 (0.2)
Hot Springs National Park	87.2 (27.9)	93.5 (13.1)	66.5 (58)	79.6 (21.4)	78.1 (30.6)	51.7 (46.8)	6.6 (6.3)	14.9 (15.1)
Hovenweep National Monument	9.8 (4.5)	7.6 (5.9)	29.9 (24.3)	41.2 (48.6)	29.2 (34.1)	28.6 (37)	27.9 (48.6)	75.4 (27.4)
Hubbell Trading Post National Historic Site	19.8 (36.5)	6.9 (7.3)	7.3 (11.4)	36.9 (28.5)	20.6 (25.5)	11.5 (31.6)	43.9 (24.9)	82.1 (28.4)
Indiana Dunes National Lakeshore	79.2 (4.4)	100 (0)	14.4 (22.1)	37.9 (52.3)	98.4 (4.9)	65.4 (40.8)	80.5 (41.9)	70.3 (19.1)
Isle Royale National Park	64.2 (37.7)	18.9 (36.7)	65.1 (47.4)	78.6 (34.3)	35.6 (36.8)	83.1 (13.5)	67.3 (61.4)	42.9 (39.5)
Jean Lafitte National Historical Park and Preserve	37.3 (86.3)	78.7 (28.1)	16.1 (30.4)	95.1 (14.6)	80.6 (12.7)	16.7 (20.3)	89.7 (13.1)	31.9 (59.2)
Jewel Cave National Monument	62.3 (37.3)	61 (29.2)	92.9 (8.2)	16.6 (35.7)	61.2 (37.5)	82.5 (35.8)	39.1 (49.3)	43.7 (41.3)
John Day Fossil Beds National Monument	78.4 (40)	39 (23.1)	5.4 (10.1)	37.3 (50.1)	51.1 (51.1)	29.4 (42.6)	9.7 (22.2)	65.4 (13.6)
John Muir National Historic Site	36.1 (46.6)	23.7 (61.7)	49 (63.4)	55.5 (31.8)	13.8 (24.6)	69.8 (27.6)	38.7 (29.3)	44.9 (61.5)

Park	Bio12	Bio13	Bio14	Bio15	Bio16	Bio17	Bio18	Bio19
Johnstown Flood National Memorial	86.5 (24.5)	54.8 (11.6)	79 (33)	42.6 (19)	48.5 (13.7)	14 (24.5)	37 (39)	36.4 (49.2)
Joshua Tree National Park	4.5 (7.4)	45.2 (61.1)	60.7 (23)	100 (0)	44.7 (58.4)	30.8 (20.3)	20.6 (3.5)	62.2 (28.8)
Kalaupapa National Historical Park	46.2 (34.8)	72.1 (26.9)	55.6 (59.4)	55.6 (48.3)	56.5 (31.4)	15.4 (41.6)	39.3 (87.7)	72.9 (45.7)
Kaloko-Honokohau National Historical Park	15.7 (21.2)	10.2 (25)	8.2 (4.7)	33.6 (63.6)	39.3 (39.8)	1.7 (1.8)	4.3 (9.9)	52.8 (25.6)
Katmai National Park and Preserve	81.8 (43.5)	94.3 (5)	23.9 (27.9)	83.1 (2.5)	55 (25.6)	51 (41.5)	32.7 (27.5)	57.6 (11.6)
Kenai Fjords National Park	52.1 (25.3)	96.7 (7.5)	58 (47.7)	51.7 (19.9)	44 (32.5)	33.7 (17.7)	65.1 (32.4)	72.2 (15.5)
Kennesaw Mountain National Battlefield Park	90.4 (14.7)	50.2 (60.4)	36.9 (42)	28.2 (42.8)	89.1 (20.7)	49.7 (25.5)	47.8 (77.6)	1.7 (1.8)
Kings Mountain National Military Park	58.4 (31.4)	19.1 (23.4)	60 (53.7)	48.8 (40.5)	67.8 (18.6)	17 (29.9)	48.7 (39.8)	3.3 (2.4)
Klondike Gold Rush National Historical Park	57.8 (62.3)	60.6 (32.6)	99.7 (1)	71.4 (28.8)	62.8 (42.9)	99.7 (1)	41 (15.1)	95.9 (6.8)
Knife River Indian Villages National Historic Site	59.7 (56.6)	51.1 (19.1)	86 (27)	60.3 (15.2)	61.5 (12.6)	57.5 (48.4)	67 (21.8)	52.3 (31.6)
Kobuk Valley National Park	22.8 (16.9)	16.3 (25.5)	94.7 (11)	13.3 (19)	39.9 (28.1)	76 (19.8)	23.3 (5.5)	99.3 (1.2)
Lake Clark National Park and Preserve	48.8 (30.6)	89.8 (27.7)	43.3 (29.3)	84.3 (7.6)	36.5 (20.6)	44.4 (58.2)	71.8 (36.3)	85.3 (24.7)
Lake Mead National Recreation Area	68 (27.8)	95.5 (13.6)	15.5 (42.4)	61.4 (73.3)	86.3 (13.9)	16.6 (22.2)	91.3 (18.4)	80.4 (29.2)
Lake Meredith National Recreation Area	48.4 (50.9)	56.6 (51.2)	70.6 (49.5)	56.6 (50.2)	51.8 (45.1)	87.7 (24.1)	89.4 (5)	43.1 (46.5)
Lake Roosevelt National Recreation Area	48.3 (16.8)	59.6 (26.9)	66.9 (12.7)	41 (56.4)	56.1 (25.6)	50.1 (13.5)	43.2 (35.2)	43.2 (44.9)
Lassen Volcanic National Park	58.5 (41.2)	38.9 (36.5)	22.5 (28.6)	6.4 (12.4)	37.3 (27.2)	27.2 (39.7)	38.6 (27.7)	48.5 (56.5)
Lava Beds National Monument	77.8 (30.2)	64.5 (20.3)	45.3 (58.2)	3.4 (4.6)	60 (23.5)	5.5 (5.4)	1.4 (0.9)	64.4 (55.4)
Lewis and Clark National Historical Park	80.7 (42.7)	51.3 (17)	43.3 (8.4)	92.9 (11.1)	90.2 (16)	58 (31.5)	57.1 (44.1)	61.7 (60.8)
Lincoln Boyhood National Memorial	99.3 (2.2)	96.1 (11.7)	72.6 (34.8)	58.5 (15)	100 (0)	96.3 (8.6)	44.8 (34)	27.2 (52)
Little Bighorn Battlefield National Monument	37.9 (72.6)	40.9 (78.4)	13.9 (18.9)	5.8 (9.5)	41.4 (64.2)	11.1 (13.9)	30.1 (5.2)	7.6 (15.8)
Little River Canyon National Preserve	83.1 (25.2)	32.9 (72.9)	82.9 (33.3)	21.5 (40.3)	44.1 (69.8)	67.8 (54.7)	26.5 (24.2)	8.2 (5.3)
Lyndon B. Johnson National Historical Park	94.1 (5.5)	95.6 (12)	38.9 (74.4)	15.7 (23.5)	89.2 (22.1)	27.3 (37.3)	96.9 (6.3)	87.1 (21.4)
Mammoth Cave National Park	35.9 (57.6)	65.6 (39.4)	74.7 (20.8)	82.4 (39.3)	37.2 (47.8)	85.8 (20.1)	8.2 (21.3)	10.1 (14.6)
Manassas National Battlefield Park	96.1 (2.4)	35.8 (52.3)	96.9 (6.3)	54.2 (28.7)	77.9 (26.3)	72.5 (26.6)	47.6 (38.3)	34.5 (29.1)
Manzanar National Historic Site	44.9 (52.4)	48.1 (13.2)	22.2 (49.3)	100 (0)	39.8 (19.5)	66.2 (18.4)	25.2 (20)	56.6 (29.8)
Marsh-Billings-Rockefeller National Historical Park	95.8 (4.6)	93.2 (9)	95.9 (4.1)	59.8 (68.5)	95.3 (9.6)	84.5 (18.6)	96.9 (4.7)	60.4 (38.2)
Mary McLeod Bethune Council House National Historic Site	65.1 (42.3)	21 (35.5)	98.1 (2.6)	60.6 (21.8)	54.8 (48.6)	58 (32.1)	21.7 (17.1)	17.2 (17.4)
Mesa Verde National Park	10.3 (5.1)	6.1 (10.5)	31 (38.4)	39.7 (59.6)	28.5 (29.8)	28.4 (21.3)	59.4 (89.7)	73.4 (23.5)
Minute Man National Historical Park	53.2 (13.2)	66.2 (22.2)	82.5 (26.5)	72.8 (41.5)	91.8 (10.6)	97.3 (6)	94.2 (8.6)	49.9 (35.8)
Mississippi National River and Recreation Area	43.8 (28)	49 (38.4)	85.3 (20.9)	16.3 (16.8)	61.9 (37.8)	12.1 (18)	59.5 (53.3)	19.2 (29.4)
Missouri National Recreational River	31.8 (5.9)	37.7 (1.8)	80.3 (6.2)	1.1 (0.2)	56.6 (8.8)	61.8 (84)	84.5 (31.1)	43.1 (31.9)
Mojave National Preserve	40.5 (30.3)	86.8 (24.8)	53.8 (51.2)	61.7 (16.4)	90.6 (6.6)	2.1 (2.8)	48.5 (26.4)	90.5 (16.5)
Monocacy National Battlefield	96.3 (4.9)	39.2 (56.9)	97.8 (2.5)	47.3 (41)	74.6 (17.7)	84.8 (20.1)	59.2 (23.7)	30.4 (25.3)
Montezuma Castle National Monument	26.8 (28.8)	31.3 (20.7)	3.1 (3.6)	39.8 (45)	50.3 (24.5)	6.5 (4.9)	66.9 (55.9)	76.1 (25.9)
Moores Creek National Battlefield	77.4 (16.3)	75.1 (51.2)	20.9 (14.2)	73.8 (42.3)	75.1 (42.2)	13.6 (14.5)	44.8 (45.1)	34.6 (54.5)
Morristown National Historical Park	66 (18.1)	39.7 (8)	97.5 (2.5)	26.7 (24)	49 (58.1)	95.1 (13.6)	51.4 (46.5)	26.9 (57.3)
Mount Rainier National Park	48.3 (58.2)	57.2 (6)	41.5 (22.4)	68.2 (68.1)	83.5 (27.7)	88.1 (12.6)	68.6 (6.6)	43.9 (54.3)
Mount Rushmore National Memorial	65.7 (32.3)	70.6 (40.9)	90.2 (11.2)	16 (32.5)	75.7 (20.8)	75 (43.4)	42 (60)	15.7 (12.1)
Muir Woods National Monument	38.2 (45.7)	35.8 (67.9)	6.2 (14.6)	51 (33.2)	10.5 (14.2)	42.9 (77.3)	15.2 (25.3)	43.9 (65.7)
Natchez Trace Parkway	57.7 (48.3)	24.9 (53.1)	85.3 (23.7)	50.4 (76.4)	29.5 (62.9)	67.2 (49.3)	65 (46.4)	10.6 (27.8)

Park	Bio12	Bio13	Bio14	Bio15	Bio16	Bio17	Bio18	Bio19
National Capital Parks-East	64.4 (43.5)	21 (35.5)	98.1 (2.6)	60 (21.8)	56.2 (43.3)	58 (33.1)	19.7 (15)	17.2 (17.4)
National Park of American Samoa	73.1 (46.7)	88 (30.1)	37.2 (38.4)	83.5 (39.8)	60.1 (45.8)	36.5 (25)	90.2 (23.4)	31.7 (44.5)
Natural Bridges National Monument	19.2 (10.3)	26.3 (12)	40.7 (29.2)	47.3 (30.9)	27.6 (41.1)	17 (33)	8.6 (14.1)	74.3 (26.4)
Navajo National Monument	47.7 (38.1)	53 (40.5)	31.2 (10.2)	16.8 (27.4)	64.2 (59.4)	11.6 (13.2)	34.2 (51.5)	75.7 (28.1)
New River Gorge National River	84.5 (8.8)	76.9 (26.5)	95.1 (13.6)	67.4 (77.3)	86.1 (12.7)	83.5 (23.5)	48.7 (40.3)	15.2 (10.8)
Nez Perce National Historical Park	30.2 (39.3)	4.5 (4.3)	43.3 (48.6)	6.2 (11.5)	34.7 (49.1)	74.7 (33.9)	56.3 (71.7)	43.1 (51.5)
Ninety Six National Historic Site	72.1 (19.4)	81.8 (10.9)	23.2 (37.5)	42 (21.4)	74.9 (19.6)	4.9 (7.7)	63.6 (53.7)	7 (13)
Niobrara National Scenic River	45.6 (37.5)	17.3 (38.1)	71.8 (17.9)	10.5 (6.8)	27.7 (48.4)	29.2 (34.9)	78.3 (7.9)	28.7 (33.7)
Noatak National Preserve	21.5 (21.9)	16 (22.2)	91.9 (15.2)	14.2 (22.3)	35.2 (21.7)	84.2 (3.8)	31.1 (7.5)	96.4 (6.3)
North Cascades National Park	5.9 (6.2)	24.5 (47.8)	19.5 (24.8)	63.4 (42.3)	57.7 (28.9)	33.8 (15.8)	44.9 (69.8)	50.8 (16)
Obed Wild and Scenic River	99.3 (1.2)	55.5 (36.2)	74.9 (19.2)	25 (40.3)	95.2 (6.6)	98.1 (2.6)	40.8 (21.6)	16.9 (15.6)
Ocmulgee National Monument	53.3 (42.7)	42.3 (19.3)	85.4 (22.4)	40.8 (39.1)	56.9 (41.3)	7.1 (5.9)	79.3 (32.8)	2.1 (2)
Olympic National Park	66.9 (57.9)	71.4 (12.4)	23.2 (14.6)	96.1 (3.7)	83.7 (22.7)	64.9 (64.9)	60.4 (38.3)	71 (40.9)
Oregon Caves National Monument	86.4 (31.1)	78.4 (37.2)	50.5 (39.8)	70.7 (61.7)	74.7 (43)	17.5 (19)	18 (42.4)	80.8 (35.2)
Organ Pipe Cactus National Monument	66.1 (64.8)	25.2 (29.7)	79.7 (15.8)	31.4 (33.8)	56.6 (76.5)	3.5 (3.6)	38.8 (77.6)	44 (15.8)
Ozark National Scenic Riverways	32.8 (34.1)	69.1 (14.6)	82.2 (23.8)	15.7 (33.6)	36.2 (59.8)	8.1 (15.1)	10.6 (15.3)	26.1 (32.5)
Padre Island National Seashore	26.5 (43.6)	55 (46.6)	65.6 (42.2)	69.6 (14.5)	48.9 (36.4)	72.2 (43.5)	58.2 (35.7)	30.2 (26.4)
Palo Alto Battlefield National Historical Park	46.3 (57.2)	72.4 (37.4)	63.6 (13.6)	98.9 (2.2)	57.8 (27.3)	74.1 (39.8)	66.7 (39.6)	8.4 (19.3)
Pea Ridge National Military Park	52.9 (58.3)	53.2 (21)	66.7 (41.6)	76.3 (21.6)	59.5 (29.5)	25.3 (45.7)	19.3 (42.3)	30.2 (66)
Pecos National Historical Park	67 (26.8)	54.1 (26.6)	20.5 (42.6)	42.1 (19.8)	78.3 (33.5)	92.5 (10.4)	91.5 (22.3)	85.5 (14.4)
Petersburg National Battlefield	84.4 (13.9)	87.2 (8.4)	88.2 (1.8)	36.6 (50.7)	96.4 (1.4)	50.3 (44.1)	54.2 (28)	28.5 (14.8)
Petrified Forest National Park	35.4 (95.3)	25.5 (22.6)	3 (4.9)	85.2 (27.9)	37.3 (90.6)	12.3 (32.7)	43.1 (63.2)	42.8 (34.3)
Petroglyph National Monument	71 (61.2)	84.5 (38.8)	29.6 (77.5)	42.5 (63.7)	84.1 (35.7)	94.7 (2.8)	95.8 (4.6)	73.8 (9.4)
Pictured Rocks National Lakeshore	40.3 (24.4)	73.2 (17.5)	99.3 (2.2)	65.5 (43.1)	17 (24.1)	78.6 (36.3)	68.8 (31.5)	69.4 (32.5)
Pinnacles National Monument	31 (26.7)	31.2 (41.6)	17 (21)	47.9 (12.4)	21.3 (14.6)	18 (29.2)	37.8 (41.6)	32.6 (20)
Pipe Spring National Monument	60.9 (18.4)	59.9 (40.4)	7.4 (17.1)	43.2 (74.9)	71.5 (15)	60.5 (27.6)	77.9 (55)	67.3 (14.9)
Pipestone National Monument	76.8 (17)	45.1 (28.5)	57 (49.1)	2.2 (3.3)	84.9 (20.9)	20.6 (34.2)	99 (2.9)	40.3 (35.5)
Piscataway Park	61.8 (54.9)	19.7 (30.8)	96.6 (4.1)	60.6 (23.7)	49.1 (63.4)	53.5 (36.6)	24.2 (16.9)	16.5 (15.2)
Point Reyes National Seashore	46.1 (37.4)	44.3 (75.2)	10.9 (21.8)	32.6 (20.5)	13 (22.4)	48 (78.8)	4.9 (4.5)	43 (68.8)
Port Chicago Naval Magazine National Memorial	37.5 (45.6)	26.4 (66.5)	46.4 (68.6)	55.4 (25.1)	18.2 (26.5)	80.2 (7.1)	44.8 (10.5)	46.8 (64.9)
Presidio of San Francisco	35.4 (50)	33.6 (66.5)	5.4 (12.2)	55 (35.4)	9.8 (13)	44.1 (81)	18.2 (30.9)	42.2 (68.6)
Prince William Forest Park	91.9 (5)	25.9 (36.4)	96.2 (6.3)	56.6 (17.5)	65.6 (41.1)	70.9 (21.8)	40.9 (31.4)	33.1 (39.3)
Pu'uhonua o Honaunau National Historical Park	27.5 (23.8)	22.7 (10.5)	1.1 (0.2)	50.3 (67.9)	46.3 (28.1)	4.1 (6.6)	16.9 (31.5)	43.4 (8.8)
Puukohola Heiau National Historic Site	23.7 (29.4)	60.4 (23.9)	1.1 (0.2)	58 (42.3)	57.3 (35.5)	2.7 (3.6)	3.8 (5.7)	68.1 (10.6)
Rainbow Bridge National Monument	51.1 (43.9)	44.5 (37.3)	27.7 (4.3)	45.4 (5.5)	62.1 (57.5)	12.7 (24.8)	33 (53.9)	72.9 (35.6)
Redwood National Park	74 (35)	75.6 (39.1)	58.6 (50.6)	41.6 (33.7)	65.6 (50.1)	19.9 (27.7)	45.5 (40)	80.2 (38.4)
Richmond National Battlefield Park	79.5 (22.6)	76.9 (25)	88.6 (1)	37 (33.1)	97.4 (4.3)	54.5 (46)	58.1 (30.4)	30.7 (18.2)
Rio Grande Wild and Scenic River	50.6 (50.5)	7 (16.4)	44.3 (37.2)	77.3 (4.9)	25.9 (43.5)	60.5 (23.8)	78.7 (35.4)	20.3 (47.1)
Rock Creek Park	67.3 (41.1)	21.3 (36.4)	98.1 (2.6)	61.1 (19.4)	57.3 (40)	59.4 (31.9)	19.7 (15)	18 (19.5)
Rocky Mountain National Park	60.5 (41.7)	86.4 (40.8)	48.3 (23.3)	20.6 (25)	78.9 (37.6)	28.3 (51.8)	64.1 (30.3)	3.2 (3.8)

Park	Bio12	Bio13	Bio14	Bio15	Bio16	Bio17	Bio18	Bio19
Roosevelt-Vanderbilt Headquarters	40.2 (22.8)	13 (4.9)	22.8 (44.9)	2.7 (5.1)	17.1 (24.1)	64.1 (38.3)	48.6 (38.3)	56.4 (41.4)
Russell Cave National Monument	86.3 (12.8)	38.9 (51.2)	90.7 (21.1)	34.9 (26.4)	56.5 (23.1)	61.7 (34.7)	40.1 (28.5)	17.2 (8.6)
Sagamore Hill National Historic Site	57.4 (21.8)	78.3 (19.3)	99.3 (1.2)	65.7 (45.7)	56.4 (34.7)	91.6 (12.4)	78.8 (21.8)	35 (40.9)
Saguaro National Park	41.3 (78.7)	89.6 (21.5)	18.1 (16.3)	88 (4.9)	67.4 (49.1)	2.8 (4.2)	70.4 (41.4)	56.3 (9.4)
Saint Croix National Scenic Riverway	40.5 (36.8)	21.3 (6)	82.3 (28.4)	9.8 (15.8)	50.9 (3.2)	22.2 (25.8)	64.4 (17.4)	24.9 (15.1)
Saint-Gaudens National Historic Site	95 (10.6)	90.9 (17)	97 (2.9)	63.8 (75.9)	98.3 (1.8)	89.7 (6.7)	95 (12.6)	62.4 (39.9)
Salinas Pueblo Missions National Monument	46.1 (19.8)	70.2 (68.9)	21.1 (43.9)	63.9 (17.9)	76.6 (37.1)	55.1 (45.4)	98 (4.9)	55.2 (26.6)
San Antonio Missions National Historical Park	85.6 (15.5)	69.7 (24.2)	7.5 (19.5)	50.5 (26.9)	86.4 (29.4)	40.2 (40.8)	75.9 (41.9)	74.3 (52.4)
San Juan Island National Historical Park	48 (48.1)	95.1 (4.4)	12.6 (14.4)	95.3 (3.9)	89 (15)	41.7 (16.3)	46.2 (20.6)	72.1 (45.5)
Sand Creek Massacre National Historic Site	44.6 (25.7)	90.7 (14.6)	77.8 (53.6)	77.9 (27.4)	80.7 (22.2)	39.8 (20.1)	72.7 (43)	8.1 (13.4)
Santa Monica Mountains National Recreation Area	44.4 (39.5)	78.8 (43.2)	57.5 (74.2)	51.7 (28.2)	61.3 (19.4)	51.6 (45.9)	36.1 (50.4)	62.8 (1.7)
Saratoga National Historical Park	70.5 (16.6)	77.9 (32.5)	35.2 (20.8)	58.6 (63.6)	44.1 (39.5)	74.1 (30.8)	91.8 (20.2)	75.2 (36.6)
Saugus Iron Works National Historic Site	49.2 (19.5)	63.5 (15.8)	86.9 (32)	80.2 (29.2)	83.9 (5.5)	95.6 (7.2)	94.6 (12.6)	46.1 (22.8)
Scotts Bluff National Monument	42 (49.4)	49 (42.2)	63.7 (16.8)	31 (45)	50.6 (58.9)	51 (62.1)	21.3 (44.4)	14.5 (15.5)
Sequoia and Kings Canyon National Parks	41.3 (53.4)	30.8 (29.5)	4.4 (6.6)	78 (20.1)	28.6 (4.7)	15.1 (27.9)	31.4 (31.2)	27.2 (10.4)
Shenandoah National Park	91.1 (8.6)	76.9 (24.8)	90.5 (16.5)	40.4 (26.8)	88.7 (16.2)	58.6 (30.4)	49 (30.7)	68.1 (53.1)
Shiloh National Military Park	32.7 (17.2)	11.2 (23.4)	89.9 (9.1)	47.7 (40.8)	5.6 (6.6)	75.9 (39.8)	77.5 (25)	6.2 (14.6)
Sitka National Historical Park	31 (48.7)	18.4 (20.9)	85.2 (25.8)	52.5 (35.6)	8.5 (17.1)	91 (18.4)	9.3 (19.9)	60.9 (30.3)
Sleeping Bear Dunes National Lakeshore	2.8 (2.7)	21.9 (24.1)	65.4 (79)	49.5 (60.2)	3.4 (6.2)	90.5 (15.5)	25.4 (8.8)	97.1 (5.5)
Stones River National Battlefield	71.7 (20.3)	70.6 (22.4)	95.9 (4.4)	85.1 (10.8)	59.5 (37.1)	87.6 (9)	60.6 (51.8)	4.5 (8.6)
Sunset Crater Volcano National Monument	51.3 (52.6)	31.5 (15.4)	4.2 (6.7)	61.6 (68.1)	60.6 (30.8)	10.2 (14.1)	82.2 (44.2)	84.4 (31.3)
Tallgrass Prairie National Preserve	32.1 (20)	48.5 (36.4)	51.1 (51.3)	11.6 (15.3)	50.5 (26)	40.2 (26.1)	8.2 (5.3)	53.2 (18.3)
Theodore Roosevelt National Park	10.2 (24)	67.5 (20.8)	68 (18.4)	74.7 (44)	43 (53.7)	50.4 (33.3)	38.1 (31.2)	52.5 (25.8)
Thomas Stone National Historic Site	59.6 (57.3)	16.5 (25.7)	95.5 (5.3)	56.1 (16.2)	44.9 (63.6)	60.2 (34.6)	29.1 (13.8)	28.3 (33.7)
Timpanogos Cave National Monument	34.6 (54.4)	58.5 (19.6)	41 (63.3)	2.7 (4.7)	43.5 (15.4)	22.7 (64.1)	17.1 (46)	38.6 (48.3)
Timucuan Ecological and Historic Preserve	31.2 (31.8)	92.1 (19.3)	40.5 (28.8)	95.5 (7.7)	59 (64.6)	53.7 (36.9)	38.7 (49.6)	39.9 (51.9)
Tonto National Monument	31.8 (46.4)	64.3 (25.9)	7 (12.2)	84 (16.5)	51.9 (20.7)	23.1 (25.2)	54.4 (59.1)	72.1 (18.3)
Tumacacori National Historical Park	58 (84.8)	75.4 (35.7)	30.3 (26.1)	80.8 (5.2)	76.7 (47.2)	18 (22.4)	72 (47.1)	52 (9.3)
Tuzigoot National Monument	25.6 (34.1)	25.5 (21.5)	2.1 (2.8)	48.5 (56.8)	54.4 (27)	11.2 (15.3)	62.8 (62.9)	80.1 (31)
Upper Delaware National Scenic and Recreational River	81.2 (24.5)	56.4 (22.3)	20.1 (48.1)	14.7 (20.7)	77.6 (13.9)	66.8 (9.8)	72.8 (53.8)	55.6 (41.2)
Valley Forge National Historical Park	78.6 (27.4)	90.3 (19.9)	87.7 (23.6)	34.9 (33.9)	96.4 (6.3)	68.2 (39.8)	76 (28.6)	20.4 (31.3)
Valor in the Pacific National Monument	79.4 (15.6)	27.7 (16.3)	7.8 (8.9)	85.7 (10.8)	55.3 (30.1)	14.5 (13.1)	44.9 (73)	53.6 (5.7)
Vicksburg National Military Park	73.6 (25.2)	50.4 (45.5)	48.2 (44.6)	44.1 (50.6)	38.6 (42.1)	14.4 (17)	52.2 (30.4)	7.2 (4.4)
Virgin Islands National Park	69.5 (39.7)	17.8 (14.3)	98.7 (3.9)	15.7 (41.1)	68.8 (29.2)	23.7 (30.1)	54.8 (50.4)	36.9 (18.1)
Voyageurs National Park	74.4 (33)	71.9 (24.6)	94.5 (15.7)	60.6 (33.6)	71.7 (34)	92.7 (11)	54.5 (31.5)	83.2 (24.5)
Walnut Canyon National Monument	50.8 (56.7)	32.8 (14.3)	4.1 (7.7)	64.3 (66.7)	65.6 (27.2)	5.5 (10.9)	82.8 (40.3)	81.8 (35.1)
War In The Pacific National Historical Park	56 (21.1)	83.9 (32)	78.9 (10)	37.5 (38.2)	84.8 (8.6)	57.9 (14.3)	81.9 (5.9)	18.9 (9)
Washita Battlefield National Historic Site	30.4 (5.6)	40.3 (50.7)	10.1 (9.3)	21.2 (15.3)	61.4 (63.9)	77.9 (35.2)	78.8 (33.1)	48.1 (60.6)
Weir Farm National Historic Site	46.9 (39.1)	62.2 (8)	62 (50.6)	36.3 (47.9)	49.8 (26)	96.3 (8.7)	68.6 (44)	61.1 (17.3)
Whiskeytown National Recreation Area	65.4 (33.3)	58.3 (52.2)	17.8 (22.7)	13.9 (8.4)	48.4 (43.6)	33.2 (26.1)	10.2 (16.2)	51.4 (62.9)

Park	Bio12	Bio13	Bio14	Bio15	Bio16	Bio17	Bio18	Bio19
White Sands National Monument	36.6 (58.9)	83.1 (21.2)	84.1 (25)	70.4 (26.4)	98.6 (4.3)	72.5 (30.1)	85.6 (24.3)	47.5 (75.8)
Whitman Mission National Historic Site	49.4 (48.1)	1.5 (1.4)	34.3 (48.3)	5.9 (14.5)	40.2 (65.7)	28.1 (31.4)	25 (18.1)	51.3 (49.6)
Wilson's Creek National Battlefield	46.7 (63.4)	85.7 (22.1)	59.3 (44.3)	23.4 (23.1)	60.3 (28.7)	26.1 (48.6)	17.9 (30.4)	49.8 (36.8)
Wind Cave National Park	68.7 (29.4)	55.1 (42.9)	88.3 (23.8)	15.8 (29.6)	68.2 (13.8)	79.2 (44.6)	45.1 (51.1)	13.5 (11.8)
Wolf Trap National Park for the Performing Arts	88.8 (12.2)	25.7 (32.3)	97.3 (5.1)	62.5 (16.2)	69.6 (29.2)	68.2 (26.6)	42.9 (34.8)	21.5 (16.6)
Wrangell - St Elias National Park and Preserve	33.7 (68.5)	19.3 (27)	27.4 (40.4)	6 (12.4)	27 (50.3)	34.5 (53)	46.4 (35.8)	50.4 (19.6)
Wright Brothers National Memorial	93 (3.9)	62.5 (68)	76.5 (35.7)	38 (39.3)	87.8 (24.6)	69.5 (24.5)	35.6 (37.3)	62.1 (63.7)
Wupatki National Monument	43.3 (49)	27 (26.3)	4.2 (6.7)	29.1 (30.5)	53.8 (32.9)	9.1 (14.7)	73.5 (61.6)	80.6 (35.3)
Yellowstone National Park	21.3 (15.5)	47.5 (32.4)	8.3 (11.8)	16.3 (16.8)	62 (42.7)	22.2 (26.6)	68.4 (48.6)	51.8 (38.4)
Yosemite National Park	57.3 (47.5)	9.2 (6.5)	8.5 (13.1)	15.7 (19.2)	15.9 (6.7)	17.4 (29.3)	16.1 (31.8)	16.3 (9.9)
Yucca House National Monument	11.4 (7)	10.9 (9.3)	46.1 (44.4)	41.9 (50.7)	36.3 (36.8)	31.1 (28.3)	56.5 (87.6)	76.5 (25.9)
Yukon-Charley Rivers National Preserve	80.6 (28.9)	70.1 (19.2)	71.7 (13.6)	79.4 (24.5)	73.6 (18.7)	53.4 (10.5)	75.3 (15.5)	64.2 (60.8)
Zion National Park	87.6 (22.1)	73.5 (38.8)	14.6 (37.6)	49 (86)	74.8 (12.1)	49.1 (27.6)	92.2 (23.3)	65.8 (25.3)
