# Supplementary material for: Climate Exposure of US National Parks in a New Era of Change
Source: PLoS One. 2014 Jul 2;9(7):e101302. doi: 10.1371/journal.pone.0101302 (PMC4079655; doi:10.1371/journal.pone.0101302)
Supplement: Appendix S8 — Mean percentile and maximum difference in percentile (in parentheses) for moving window standard deviations: mean annual percentage cloud cover (Cld1), cloud seasonality (Cld4), vapor pressure of the warmest quarter (Vap18), annual number of wet days (Wet12), number of wet days of the warmest quarter (Wet18), and annual number frost days (Frs12). NA’s associated with Frs12 indicate that the variable is undefined (i.e., no frost days exist). (PDF) [file pone.0101302.s008.pdf]

Appendix S8. Mean percentile and maximum difference in percentile (in parentheses) for moving window standard deviations: mean annual percentage cloud cover (Cld1), cloud seasonality (Cld4), vapor pressure of the warmest quarter (Vap18), annual number of wet days (Wet12), number of wet days of the warmest quarter (Wet18), and annual number frost days (Frs12). NA's associated with Frs12 indicate that the variable is undefined (i.e., no frost days exist).

Park	Cld1	Cld4	Vap18	Wet12	Wet18	Frs12
Abraham Lincoln Birthplace National Historical Park	75.6 (68)	50.4 (43.9)	99.3 (2.2)	91 (15.7)	95.4 (1.8)	81.7 (50.5)
Acadia National Park	3.9 (6.3)	53.8 (91.3)	32.7 (63)	18.1 (34)	27.2 (32.1)	97.7 (4.9)
Agate Fossil Beds National Monument	52.2 (37.3)	28.5 (35.8)	74.7 (44.8)	97.7 (6)	98.4 (4.9)	41.7 (56.1)
Ala Kahakai National Historic Trail	98.1 (5.8)	98.3 (3.9)	40.6 (44.3)	16.1 (8.7)	10.1 (17)	42.4 (18.4)
Alagnak Wild River	5.4 (10.6)	14.5 (24.9)	13.7 (27)	88.3 (35)	65.3 (13.1)	50.3 (16.6)
Alibates Flint Quarries National Monument	29.2 (45.6)	87.7 (27.2)	30.9 (50.2)	83.3 (24.6)	65.8 (3.4)	37.9 (59)
Allegheny Portage Railroad National Historic Site	38.3 (50.4)	42.5 (57)	100 (0)	90.5 (15.7)	84.8 (21.7)	53.4 (34.9)
American Memorial Park	98.7 (3.9)	93.9 (18.3)	54.3 (10.6)	40 (35)	81.8 (21.4)	NA (NA)
Amistad National Recreation Area	52.3 (40.3)	90.6 (16.8)	73 (51.9)	99 (2.2)	85.1 (32.9)	30.7 (20.2)
Anacostia Park	42.7 (50.1)	39.3 (30.4)	97.1 (8.7)	96 (5.6)	57.3 (80.7)	65.4 (24.7)
Aniakchak National Monument and Preserve	2.3 (3.8)	15.8 (28.8)	24.4 (50.3)	91.9 (24.3)	35.9 (32.9)	73.8 (21.3)
Antietam National Battlefield	44.1 (49.8)	46.8 (35.5)	97.4 (7.8)	96.7 (2.4)	68.4 (70.1)	57.6 (68.3)
Apostle Islands National Lakeshore	12.9 (9.3)	10.2 (15.8)	87.2 (19.1)	96.1 (3.7)	90.1 (27.7)	77.3 (67)
Appalachian National Scenic Trail	40.5 (59.7)	41.7 (35.6)	96.4 (0.7)	98.6 (2.9)	96.1 (11.7)	78.2 (32.8)
Appomattox Court House National Historical Park	74.6 (51)	33.5 (36.1)	96.4 (8.7)	96.7 (2.9)	85.8 (25.7)	68.7 (25.9)
Arches National Park	14.7 (16.2)	19.5 (6.9)	20 (28.4)	73 (36.2)	96.1 (11.7)	60.5 (59.3)
Arkansas Post National Memorial	43.7 (26.7)	16.9 (39.6)	98.2 (4.3)	88.1 (9.9)	81.6 (21.4)	38.7 (10.7)
Assateague Island National Seashore	44.8 (30.2)	9.7 (12.2)	97.7 (6.8)	99.4 (1.9)	90.2 (28.2)	62.5 (33.8)
Aztec Ruins National Monument	15.5 (30.7)	14.9 (14.7)	27.2 (68.2)	45.5 (39.1)	93.1 (18.4)	51.5 (64.6)
Badlands National Park	66.9 (53.4)	22.2 (14.9)	85.8 (34.9)	99.4 (1.9)	69.3 (39.9)	78.8 (16.1)
Baltimore-Washington National Parkway	41.7 (48.9)	39 (29.4)	97.4 (7.8)	95.6 (5.6)	57.6 (78.8)	62.3 (19.9)
Bandelier National Monument	24.2 (27.1)	53.6 (50.7)	32.3 (81.7)	74.3 (63.1)	93.3 (12.4)	56.8 (65.9)
Bent's Old Fort National Historic Site	34.7 (52.1)	74.3 (40.2)	52 (72.8)	88.7 (27.1)	80.3 (10.5)	25.5 (23.4)
Bering Land Bridge National Preserve	1.1 (0.2)	21.9 (28.5)	71 (27.8)	56.1 (32.5)	72.7 (41.4)	64.1 (48.8)
Big Bend National Park	74 (34.6)	90 (14.1)	52.3 (44.9)	86.8 (24.2)	52 (28.6)	42.2 (52.2)
Big Cypress National Preserve	72.7 (64.7)	89.4 (14.1)	83.2 (15.6)	48.5 (31.4)	62.3 (36.3)	31 (54.2)
Big Hole National Battlefield	47.3 (40)	61.5 (20.4)	88.2 (13)	26.6 (33.9)	96.4 (10.7)	14 (26.3)
Big South Fork National River and Recreation Area	80.7 (51.5)	50.9 (48.8)	99.3 (1.1)	94.4 (8.7)	99.7 (1)	54.8 (48.3)
Big Thicket National Preserve	25.6 (10.8)	11.5 (20)	90.4 (25.8)	93.3 (12.8)	70.2 (22.2)	43.4 (48.5)
Bighorn Canyon National Recreation Area	18.3 (26.1)	60.1 (15.5)	80.4 (47.4)	77.3 (22)	97 (5.7)	21.1 (25.5)
Biscayne National Park	76.8 (51.1)	87.6 (4.9)	76.9 (6.4)	45 (32.9)	66.3 (37.4)	NA (NA)
Black Canyon Of The Gunnison National Park	22.3 (36.5)	25.1 (21)	9.6 (19.5)	91.3 (18)	96.4 (10.7)	33.9 (25.2)
Blue Ridge Parkway	57.5 (14.1)	45.6 (38.1)	100 (0)	96.7 (2.4)	97.7 (5.8)	52.6 (27.2)
Bluestone National Scenic River	51.4 (10.4)	51.8 (54.1)	98.7 (2.9)	96 (4.5)	96.7 (8.7)	37.6 (16.8)
Booker T. Washington National Monument	75.6 (39.6)	39.3 (33.2)	99 (2.2)	97.1 (1.7)	95.7 (11.7)	59.1 (38.3)
Boston Harbor Islands National Recreation Area	30.5 (48.5)	44 (87.4)	74.8 (11.9)	64 (35)	82.7 (42.6)	80.3 (19.2)

Park	Cld1	Cld4	Vap18	Wet12	Wet18	Frs12
Bryce Canyon National Park	14.4 (24.8)	32.6 (40.4)	21.7 (34.9)	53.3 (58.1)	83.1 (38.6)	83 (37.7)
Buck Island Reef National Monument	95.4 (10.6)	93 (13.6)	15.5 (37.5)	98.1 (3.6)	100 (0)	NA (NA)
Buffalo National River	20.2 (24.8)	15.5 (28.7)	89.5 (25.8)	82.8 (26.6)	93.1 (9.8)	50.2 (10.5)
Cabrillo National Monument	54.5 (90.6)	18.3 (30.2)	64 (67)	19.8 (11.2)	46.1 (49.3)	4.1 (7.7)
Canaveral National Seashore	51 (58.5)	76.2 (25.3)	57.4 (18.3)	97.8 (2.5)	64.5 (51)	82.7 (31.6)
Canyon De Chelly National Monument	14.2 (26.8)	15.7 (18.3)	26.1 (58.8)	26.9 (30.5)	80.1 (35.5)	47.1 (56.4)
Canyonlands National Park	18.7 (28.2)	27.5 (17.9)	19.1 (31.8)	58.4 (50.6)	93.9 (18.4)	44.9 (41.7)
Cape Cod National Seashore	1.5 (1.4)	44.9 (82.4)	68.4 (39.4)	61.6 (31.9)	89.8 (26.1)	78.5 (49.5)
Cape Hatteras National Seashore	38.4 (45.1)	5.4 (9.5)	94.5 (16.5)	98.6 (1.2)	25.2 (3.6)	35.9 (68)
Cape Krusenstern National Monument	2.7 (4.7)	17.5 (20.4)	70.4 (24.9)	35.6 (67)	64.8 (38.5)	59.9 (71.4)
Cape Lookout National Seashore	48.4 (54.9)	5 (9.5)	87.3 (20.9)	97.1 (5.5)	22.8 (24.1)	55 (44.4)
Capitol Hill Parks	42.7 (50.1)	40.1 (29.3)	97.1 (8.7)	96 (5.6)	57.7 (80.7)	65.6 (31)
Capitol Reef National Park	10.8 (14.1)	31.2 (31.5)	29.2 (45.5)	61.1 (55.4)	84.5 (37.6)	23.4 (30)
Capulin Volcano National Monument	31.7 (52.2)	81.8 (40.6)	38.5 (56.1)	91.1 (22.3)	97.7 (4.9)	25.7 (22.9)
Carl Sandburg Home National Historic Site	44.8 (29.9)	50.7 (26.5)	93.3 (15.7)	97.8 (2.5)	99.3 (1.2)	26.6 (18.4)
Carlsbad Caverns National Park	70.3 (49.4)	86.7 (30.9)	34.6 (92.9)	64.6 (28)	58.6 (53.4)	38.7 (81.6)
Casa Grande Ruins National Monument	69.6 (52.3)	38.4 (40.1)	20.2 (4.2)	27.1 (46.4)	66.6 (48.9)	11.6 (15.3)
Castillo De San Marcos National Monument	22.7 (17.8)	17.9 (2.5)	69.2 (13.8)	97.8 (0.5)	72.2 (44.5)	98.9 (2.2)
Catoctin Mountain Park	43.7 (48.6)	47.1 (35.9)	97.4 (7.8)	96.7 (2.4)	72.1 (61.7)	56.6 (68.8)
Cedar Breaks National Monument	18.7 (25.8)	35.4 (38.7)	26.9 (40)	56.1 (54)	71.3 (48.1)	84.6 (9.9)
Chaco Culture National Historical Park	16 (32.3)	19.9 (25)	31.7 (74.4)	41.7 (53.4)	89.4 (23.1)	12.8 (14.7)
Channel Islands National Park	51.8 (85.8)	1.1 (0.2)	62.9 (74.3)	28.6 (17.4)	37 (38.1)	1.8 (1.4)
Charles Pinckney National Historic Site	29.5 (5.6)	6.5 (8.6)	80.3 (11.5)	77.8 (25.1)	64.6 (21.3)	62.5 (35.3)
Chattahoochee River National Recreation Area	62.7 (60.5)	57.8 (79.1)	97.8 (5.4)	90.4 (12.2)	65.8 (35.8)	35.1 (59.8)
Chesapeake and Ohio Canal National Historical Park	44 (49.6)	43.8 (32.9)	97.7 (6.8)	96.7 (2.4)	68.3 (69.2)	55.1 (56.7)
Chickamauga and Chattanooga National Military Park	71.4 (63.1)	56 (45.8)	98.9 (0.2)	91.1 (6.9)	95.7 (1.8)	28.6 (11.5)
Chickasaw National Recreation Area	27.7 (46.2)	31.1 (28.6)	71.4 (56.3)	97.6 (4.3)	62.8 (23.4)	30.6 (23.7)
Chiricahua National Monument	34.4 (19.3)	33.6 (28.5)	40.8 (89.6)	28.1 (62.9)	78.4 (36.7)	3.6 (3.4)
City Of Rocks National Reserve	35.6 (48.7)	25.3 (36.1)	30.2 (37.8)	87.6 (25.2)	78.9 (21.3)	42.5 (6.9)
Colonial National Historical Park	37.8 (19.4)	28.5 (29.1)	94.8 (15.5)	99.7 (1)	54.6 (52.6)	45.6 (80.7)
Colorado National Monument	15.5 (20)	21.8 (22.6)	17.6 (20.4)	83.5 (28.9)	96.1 (11.7)	52.7 (69.5)
Congaree National Park	17.3 (12)	16.5 (33.9)	95.1 (12.6)	99.7 (1)	97.6 (6)	32.5 (6)
Coronado National Memorial	34.5 (23.5)	29.8 (31)	50.3 (74.6)	27.4 (65.8)	82.1 (29.6)	31.8 (77.6)
Cowpens National Battlefield	38.2 (20.8)	46.2 (31.5)	97.6 (6)	98.1 (2.6)	99.7 (1)	15.3 (23.8)
Crater Lake National Park	57 (90.1)	44.3 (20.8)	57.2 (87)	92.5 (12.9)	70 (8.1)	31 (42.3)
Craters Of The Moon National Monument and Preserve	25.7 (47.1)	38.9 (37.7)	51.3 (47.1)	79.1 (45.6)	73.8 (15.2)	23.9 (17)
Cumberland Gap National Historical Park	67.5 (32.6)	68.4 (40.4)	99 (1.9)	95.2 (4.5)	99 (2.9)	62.5 (53.7)
Cumberland Island National Seashore	13.4 (16.3)	19.4 (15.4)	73.6 (27.6)	98.2 (0.9)	88.4 (23.4)	78.3 (36.6)
Curecanti National Recreation Area	25.8 (47.2)	28.6 (18.1)	9.5 (19.4)	92.8 (19.4)	96.4 (10.7)	35.8 (36.9)
Cuyahoga Valley National Park	21.9 (31.6)	36 (68.1)	98.1 (4.8)	86 (19.2)	80.3 (10.5)	60.9 (13.6)

Park	Cld1	Cld4	Vap18	Wet12	Wet18	Frs12
Death Valley National Park	65.7 (93.2)	20.3 (6.4)	65.3 (82.1)	43.6 (15.9)	51.1 (41.2)	51.6 (60.1)
Delaware Water Gap National Recreation Area	39.7 (67.8)	34.9 (92.9)	95.3 (0.6)	96.2 (5.3)	95.7 (11.7)	64.3 (14.2)
Denali National Park and Preserve	3.4 (5.7)	4 (7.5)	9.2 (12.2)	62.6 (21.8)	97.2 (4.9)	82.7 (26.8)
Devils Postpile National Monument	67.6 (87.1)	31.3 (3.4)	62.2 (96.6)	51.8 (16)	21 (37.2)	52.5 (83.6)
Devils Tower National Monument	37.9 (58.6)	37.6 (29.7)	74.8 (43.6)	96.5 (8.4)	73.4 (36.1)	38.2 (31.9)
Dinosaur National Monument	8.1 (15.1)	20.7 (31.1)	28.2 (17.2)	87 (37.9)	93.2 (20.4)	9.8 (13)
Dry Tortugas National Park	40.3 (16.1)	90.5 (15.5)	48.6 (11.5)	49.5 (34.3)	75.1 (33.8)	NA (NA)
Ebey's Landing National Historical Reserve	32.6 (6.4)	47.7 (21.7)	92.3 (5.9)	44.5 (61.1)	35.2 (47.9)	39.8 (35.7)
Effigy Mounds National Monument	40.1 (80.2)	17 (31.5)	90 (10.1)	96.4 (2.7)	99 (2.9)	64.1 (73.5)
Eisenhower National Historic Site	40.8 (46.5)	48.8 (38.8)	97.4 (7.8)	98.2 (1.3)	78.3 (53.7)	58 (67.1)
El Malpais National Monument	31.1 (28.7)	30.5 (37.4)	26.5 (71.3)	47.4 (73.2)	89.7 (22.1)	60.7 (64.4)
El Morro National Monument	26.3 (21)	26.5 (36)	24.5 (63)	42.4 (61.6)	86.4 (30.8)	66.9 (46)
Eugene O'Neill National Historic Site	43.7 (88.9)	26.5 (30.1)	60.7 (84.5)	81.6 (39.8)	40.1 (16.2)	14.3 (16.1)
Everglades National Park	72.9 (64.8)	88.8 (8.5)	79.9 (4.1)	48.2 (30.4)	75.3 (38.2)	33.2 (53.7)
Fire Island National Seashore	24.1 (31.2)	31.1 (81.1)	93.8 (7.8)	76 (30.8)	91.9 (24.3)	82 (13.2)
Florissant Fossil Beds National Monument	32.2 (56.1)	47.2 (26.9)	41.6 (51.4)	75.5 (29.1)	92.5 (19.4)	26.5 (28.7)
Fort Bowie National Historic Site	38.1 (21.7)	36.2 (30.9)	40.4 (86.2)	29.1 (62.9)	81.4 (40.5)	5.5 (4.6)
Fort Caroline National Memorial	15.6 (15.1)	17.3 (9.5)	58.9 (7.9)	97.8 (0.5)	83 (33.2)	91 (20.5)
Fort Davis National Historic Site	71.1 (47.5)	80.5 (24.7)	67.1 (67.7)	80.8 (30.4)	47.8 (45)	53.5 (43.6)
Fort Donelson National Battlefield	53.7 (68.6)	33.2 (54.2)	92.5 (10.4)	86.7 (10.8)	98.1 (2.6)	26.3 (25.1)
Fort Frederica National Monument	12.7 (14.4)	19.3 (24.6)	80.3 (28.6)	99 (1.9)	91 (21.7)	74.7 (36.3)
Fort Laramie National Historic Site	42.8 (36.9)	42.3 (30.3)	63.5 (58.1)	95.3 (13.3)	95.8 (12.6)	42.1 (54.7)
Fort Larned National Historic Site	20.8 (33.1)	55.9 (11.5)	54.8 (50.7)	82.6 (35.2)	96.6 (6.5)	45 (27.7)
Fort Matanzas National Monument	24.1 (13.8)	30.5 (20.3)	71.4 (17.2)	97.8 (0.5)	69.8 (51.7)	98.5 (2.4)
Fort Moultrie National Monument	30.6 (5.8)	6.8 (8.6)	79.9 (11.1)	77.8 (25.1)	64.6 (21.3)	63.9 (36)
Fort Necessity National Battlefield	58.6 (83.4)	26.6 (36.5)	99.6 (1.2)	96.8 (7.5)	84 (32.3)	69.4 (66.3)
Fort Point National Historic Site	32 (72.3)	22 (35.3)	55.3 (84.1)	83.4 (34.4)	55.4 (24.4)	4 (8.6)
Fort Pulaski National Monument	16.2 (5.2)	29.2 (48)	85.7 (13.7)	98 (4.9)	86.2 (36.4)	68.6 (36.5)
Fort Raleigh National Historic Site	33.1 (29.9)	7.7 (14.1)	92.2 (23.3)	99.7 (1)	38.2 (26.1)	30.8 (70.4)
Fort Sumter National Monument	29.2 (5.6)	6.8 (8.6)	81.5 (13.2)	77.8 (25.1)	66.3 (24.5)	63.9 (36)
Fort Union National Monument	22.9 (36)	83.4 (40.6)	33.3 (83.3)	83.8 (38.8)	96.7 (7.8)	66 (90.3)
Fort Union Trading Post National Historic Site	69.3 (39)	50.6 (42.9)	69.5 (56.7)	27.6 (35.3)	79.1 (14.2)	59.5 (70.2)
Fort Vancouver National Historic Site	64.4 (75.5)	41.7 (23.1)	62.6 (66.5)	73.1 (60.2)	95.8 (8.4)	31.2 (27.6)
Fort Washington Park	44.4 (54.5)	39.5 (26.4)	97.1 (8.7)	96 (5.6)	55.7 (80.7)	66.7 (31)
Fossil Butte National Monument	10.7 (22.8)	5.2 (7.6)	32.7 (37.3)	87.8 (15.7)	58 (69.1)	46.1 (26.2)
Frederick Douglass National Historic Site	44.9 (56.5)	40.1 (29.3)	97.1 (8.7)	96 (5.6)	56.1 (80.7)	66.2 (25.9)
Fredericksburg and Spotsylvania National Military Park	42 (42)	42.8 (30)	96.8 (9.7)	95.7 (6.7)	66.3 (72)	65 (74.3)
Friendship Hill National Historic Site	57.7 (88.4)	26.7 (41.1)	98.5 (3.2)	97.6 (5.4)	84.8 (25)	72.5 (61.9)
Gates Of The Arctic National Park and Preserve	4.8 (7.6)	3.3 (6.7)	18 (27.5)	44.2 (38.8)	87.2 (7.9)	56.1 (54.8)
Gateway National Recreation Area	19.4 (26.5)	30.3 (82.1)	97.4 (3.3)	87 (22.2)	92.5 (21.4)	90.1 (5.5)

Park	Cld1	Cld4	Vap18	Wet12	Wet18	Frs12
Gauley River National Recreation Area	38.7 (32.7)	45.2 (38.6)	99.4 (1.9)	96 (4.5)	94.4 (5.1)	35.3 (21.2)
George Washington Birthplace National Monument	42.1 (31.9)	38.7 (30.6)	97.1 (8.7)	96 (5.6)	53.6 (73.5)	65.7 (41.8)
George Washington Carver National Monument	21.9 (14)	19.8 (38.4)	69.7 (51.8)	41.8 (43.3)	99.6 (1.2)	47.7 (30.6)
George Washington Memorial Parkway	39.8 (41.5)	40.7 (29.2)	97.1 (8.7)	95.6 (5.6)	61.3 (79.7)	62.4 (50.4)
Gettysburg National Military Park	41.5 (48.7)	49.1 (39.8)	97.4 (7.8)	98.2 (1.3)	78.3 (53.7)	59.7 (56.9)
Gila Cliff Dwellings National Monument	40.2 (30.4)	43.2 (45.9)	40.3 (83.3)	41.7 (83.6)	86.8 (30.8)	63.3 (94.9)
Glacier Bay National Park and Preserve	8.3 (11.3)	44 (58.7)	34.2 (32.9)	37.2 (21.6)	78.4 (16.6)	23.5 (24.8)
Glacier National Park	7.5 (10.9)	60.4 (25.4)	69.4 (56.1)	54.6 (83.4)	61.6 (68.3)	61.6 (47.9)
Glen Canyon National Recreation Area	13.7 (18)	33.7 (29.5)	29.6 (54.9)	41.8 (44.9)	83.8 (38.6)	50.1 (22.8)
Golden Gate National Recreation Area	28.8 (73.7)	18.7 (35.2)	49.3 (84.3)	84.1 (31.2)	53.2 (21.9)	6.4 (12.4)
Golden Spike National Historic Site	38.6 (49.2)	16 (41.1)	8.7 (19.5)	90.7 (11.2)	78.4 (16.2)	43 (16.9)
Grand Canyon National Park	49.5 (52.4)	38.2 (38)	21.1 (37.3)	34.2 (15.2)	58.2 (28.6)	69.3 (26.8)
Grand Portage National Monument	15.7 (12.9)	33.5 (66.4)	87.2 (19.1)	98.9 (2.2)	85.4 (36.1)	78.3 (55)
Grand Teton National Park	27.4 (51.9)	34.9 (62.4)	51.6 (58.3)	26 (16.2)	80.8 (55.3)	35.5 (50.6)
Grant-Kohrs Ranch National Historic Site	35.1 (55.9)	58.4 (20.3)	71.2 (81.6)	8.6 (8.2)	83.4 (38.8)	54.8 (87.7)
Great Basin National Park	35.5 (55.3)	39.5 (47.3)	42.1 (44.7)	77.8 (31.9)	46.3 (81.7)	57.9 (50.1)
Great Sand Dunes National Park and Preserve	34.4 (59)	42.1 (25)	34.3 (45.7)	77.4 (49.3)	92.2 (21.4)	23.3 (40.5)
Great Smoky Mountains National Park	71.6 (48.3)	60.7 (31.2)	99.3 (1.1)	96 (3.4)	99.7 (1)	47.7 (26.2)
Greenbelt Park	42 (47.9)	39 (29.4)	97.1 (8.7)	95.6 (5.6)	57.6 (78.8)	62.8 (21.8)
Guadalupe Mountains National Park	69.2 (49.4)	84.5 (26.9)	34.6 (92.9)	62.6 (26.3)	53.3 (52.7)	36.9 (90.8)
Guilford Courthouse National Military Park	58.6 (5.7)	29.4 (29.3)	97.7 (4.9)	97.4 (1.7)	96.4 (9.7)	67.1 (51.5)
Gulf Islands National Seashore	42.9 (34.9)	34 (72.5)	76.8 (41.3)	89.1 (15.3)	85.4 (13.9)	65.5 (43.5)
Hagerman Fossil Beds National Monument	26.8 (43.2)	27.5 (37.9)	92.2 (11.5)	80.4 (43.7)	56.5 (17)	30.4 (27.6)
Haleakala National Park	97.7 (6.8)	96.8 (1.5)	20.3 (28.2)	31 (18)	49.1 (68.6)	NA (NA)
Harpers Ferry National Historical Park	44 (48.6)	46.6 (33.6)	97.4 (7.8)	96.7 (2.4)	67 (73.1)	59.8 (69.3)
Hawaii Volcanoes National Park	98.1 (5.8)	99 (2.9)	41.8 (47.2)	17.5 (15.6)	10.4 (20.2)	42.4 (18.4)
Herbert Hoover National Historic Site	40.9 (81.9)	18.2 (30.9)	95 (3.9)	97.5 (2.8)	97 (6.8)	67.7 (69.4)
Homestead National Monument of America	40.1 (43.9)	16.9 (25.7)	80.8 (26.3)	100 (0)	100 (0)	61 (37.7)
Hopewell Culture National Historical Park	17.6 (19.7)	44.4 (32.7)	98.7 (3.9)	96.2 (4.2)	91.3 (6.6)	76.1 (63.1)
Hopewell Furnace National Historic Site	32.2 (47)	31.4 (64.4)	91.9 (15.3)	97.4 (1.7)	88.9 (30)	48 (18.9)
Horseshoe Bend National Military Park	56.3 (62)	50.7 (91.7)	81.9 (34.9)	52 (70)	64.3 (24.4)	33.2 (64.6)
Hot Springs National Park	43.2 (57.9)	17.7 (36.4)	89.1 (26.9)	86.1 (15.8)	78.7 (32.2)	50.2 (34.7)
Hovenweep National Monument	15.8 (17.4)	13.9 (10.5)	23.6 (48.9)	33 (39.4)	93.8 (17.5)	45.3 (26.2)
Hubbell Trading Post National Historic Site	15.5 (30.7)	18.8 (24.1)	27.6 (60.9)	27.2 (28.3)	70.2 (29)	50.1 (72.2)
Indiana Dunes National Lakeshore	38.6 (85.8)	14.5 (27.8)	95.1 (12.6)	99 (1.9)	96.2 (4.6)	56.7 (22.1)
Isle Royale National Park	15 (12)	30.1 (62.8)	90.4 (19.1)	93.9 (17.2)	68.5 (58.8)	83.7 (45.6)
Jean Lafitte National Historical Park and Preserve	15.4 (19.7)	18.1 (26)	97.5 (6.5)	76.9 (44.5)	75.1 (25.6)	35.8 (53.3)
Jewel Cave National Monument	49.4 (46.4)	24.2 (15.9)	80.7 (48.2)	98.5 (2.4)	64.2 (69.9)	21.4 (32.3)
John Day Fossil Beds National Monument	50.5 (65.8)	48 (21.9)	86.8 (13.8)	73.7 (56.1)	95.4 (6.7)	44.4 (38.3)
John Muir National Historic Site	43.6 (86.4)	24.4 (35.5)	60 (83.3)	83.3 (35.5)	42.5 (21)	23 (29)

Park	Cld1	Cld4	Vap18	Wet12	Wet18	Frs12
Johnstown Flood National Memorial	38.3 (50.4)	42.5 (55.9)	100 (0)	87.7 (22.9)	78.1 (23)	46.3 (36.6)
Joshua Tree National Park	66.5 (96.1)	35 (50.9)	68.1 (79.6)	32.4 (15.3)	70.9 (49.5)	5.5 (13.5)
Kalaupapa National Historical Park	97.7 (6.8)	97.2 (2.7)	24.4 (37.8)	84.1 (24.5)	77.6 (33.8)	NA (NA)
Kaloko-Honokohau National Historical Park	98.1 (5.8)	97.3 (4.3)	32.4 (43.1)	12.4 (13.4)	10.8 (2.4)	42.4 (18.4)
Katmai National Park and Preserve	7.8 (11.5)	22 (24.8)	14.4 (28.9)	89.3 (32)	57.8 (10)	58.7 (31.1)
Kenai Fjords National Park	9 (17)	5.1 (9.6)	4.4 (8.5)	74.4 (44)	92.5 (11.7)	34.7 (53.5)
Kennesaw Mountain National Battlefield Park	60.7 (63.1)	57.2 (79.1)	98.5 (3.2)	83.3 (24.1)	51.9 (48)	37.4 (56.9)
Kings Mountain National Military Park	38.3 (22.6)	39.2 (35.9)	96 (4.9)	98.5 (1.4)	99.3 (1.2)	25 (19.8)
Klondike Gold Rush National Historical Park	3.8 (6.7)	32.9 (71.2)	47.3 (36)	60.1 (30.9)	82.2 (14.1)	45.9 (51.9)
Knife River Indian Villages National Historic Site	50.6 (33.6)	36.2 (61.1)	93.1 (9.5)	98.3 (1.8)	59.3 (64.3)	55.5 (78.5)
Kobuk Valley National Park	3.3 (6.7)	19.6 (8)	39.1 (58.3)	46.7 (12.2)	70.4 (25.7)	54 (57.7)
Lake Clark National Park and Preserve	22.5 (22.7)	15.8 (29.6)	9.2 (14.3)	66.6 (33.9)	66.9 (26.8)	66.9 (22.8)
Lake Mead National Recreation Area	80.6 (37.6)	30.4 (38.8)	27.1 (46.7)	41.1 (26.1)	58.1 (29.5)	44.6 (75.6)
Lake Meredith National Recreation Area	29.5 (44.5)	88 (26.2)	31.2 (51.1)	84.8 (24.6)	67.6 (4.8)	40.4 (59)
Lake Roosevelt National Recreation Area	15.7 (17.3)	48.8 (8.1)	70 (26.9)	18.4 (27.1)	78.8 (49)	13.6 (19.5)
Lassen Volcanic National Park	72.2 (73.6)	45.2 (30.3)	61.6 (79.4)	89.2 (23.7)	69.1 (37.2)	81.5 (35.3)
Lava Beds National Monument	61.5 (86.2)	28.1 (32.2)	60.8 (77)	94.8 (9.7)	72.8 (10.4)	39.3 (57.2)
Lewis and Clark National Historical Park	57.8 (81.3)	42 (31.6)	68.7 (37)	68.9 (32.8)	100 (0)	41.5 (36.2)
Lincoln Boyhood National Memorial	67.8 (92.2)	29.3 (44.6)	100 (0)	67.4 (45.2)	96.2 (7.5)	70.7 (43.6)
Little Bighorn Battlefield National Monument	19.4 (29.9)	59 (9.3)	81.9 (36.7)	85 (22.1)	89.8 (13.3)	33.2 (28.9)
Little River Canyon National Preserve	59.1 (68)	48 (68.5)	98.9 (0.2)	87.8 (9.5)	89 (12.9)	23.7 (9.8)
Lyndon B. Johnson National Historical Park	46.3 (38.5)	49 (13.9)	91.9 (21.5)	96.9 (4.6)	94.7 (7.7)	18.6 (20.2)
Mammoth Cave National Park	72.9 (74.8)	41.2 (53.5)	99.3 (2.2)	89.6 (15.6)	96.9 (4.9)	86.2 (24.7)
Manassas National Battlefield Park	44.1 (50.9)	44.9 (26)	97.1 (8.7)	95.6 (5.6)	63.3 (80.7)	68.7 (56.6)
Manzanar National Historic Site	66.1 (94.2)	31.9 (13.7)	63 (87.9)	48.8 (8.8)	58.9 (31.9)	61.8 (57.5)
Marsh-Billings-Rockefeller National Historical Park	21.7 (36.4)	55.5 (78.4)	36.4 (21.1)	78.9 (41)	92.2 (22.3)	90.4 (4.1)
Mary McLeod Bethune Council House National Historic Site	41.3 (45.8)	39.7 (29.3)	97.1 (8.7)	95.6 (5.6)	58.5 (80.7)	66.2 (36.5)
Mesa Verde National Park	14.9 (22.9)	14.1 (12.5)	20.2 (44.1)	40.9 (30.1)	95.8 (11.7)	44.6 (41.8)
Minute Man National Historical Park	35.6 (61.4)	43.3 (87)	72.6 (9.9)	60.9 (31.9)	82.3 (41.5)	91.2 (10.1)
Mississippi National River and Recreation Area	16.5 (41.2)	7.5 (9.8)	92.6 (11.6)	94.3 (4.5)	97.6 (7.2)	76.1 (71.8)
Missouri National Recreational River	65.8 (55.3)	32 (42.7)	95 (8.4)	99.4 (1.9)	99.6 (1.1)	71.4 (68.9)
Mojave National Preserve	69.6 (82.3)	18.5 (24.2)	54.2 (71.7)	34.7 (10.6)	58.5 (35.2)	35.6 (92)
Monocacy National Battlefield	42.7 (45.5)	47.9 (35)	97.4 (7.8)	96 (4.5)	68.1 (72.1)	60.2 (68.5)
Montezuma Castle National Monument	66 (40.3)	40.1 (29.5)	14 (20.4)	27.6 (19.4)	50 (20.6)	12.4 (24.5)
Moores Creek National Battlefield	40.2 (40.8)	3.3 (6.7)	76.7 (21.1)	96.4 (9.6)	36.6 (49.4)	40.3 (36.1)
Morristown National Historical Park	25 (44.2)	32.7 (89.3)	97.5 (3.1)	89.7 (16.1)	93.4 (18.4)	78.1 (19.2)
Mount Rainier National Park	52.6 (68.1)	55.8 (46.5)	90.8 (4.1)	46.9 (62)	74.5 (13.7)	10.4 (18.7)
Mount Rushmore National Memorial	53.3 (40.8)	22.2 (10.1)	80.7 (48.2)	98.7 (3.9)	63.3 (67.6)	41.7 (26.2)
Muir Woods National Monument	29.8 (71.8)	19.6 (35.2)	48.1 (82.9)	85.1 (30.1)	55.9 (19.8)	6 (13.4)
Natchez Trace Parkway	51.9 (28.9)	19.4 (36.5)	85.4 (40.9)	92.5 (1.8)	90.4 (2.2)	26.2 (50.3)

Park	Cld1	Cld4	Vap18	Wet12	Wet18	Frs12
National Capital Parks-East	41.6 (46.8)	39.7 (29.3)	97.1 (8.7)	95.6 (5.6)	58.5 (80.7)	66.2 (36.5)
National Park of American Samoa	97.4 (7.8)	100 (0)	97.6 (7.2)	5.1 (9.8)	100 (0)	NA (NA)
Natural Bridges National Monument	22.6 (37.9)	34 (27.3)	19.7 (39.3)	38.4 (34.4)	87.4 (28.7)	47.1 (6.1)
Navajo National Monument	10.7 (20.7)	32.1 (36.1)	36.3 (73)	24.1 (14.3)	68.8 (38.4)	67.4 (48.6)
New River Gorge National River	47.9 (18.3)	47.8 (47)	99 (1.9)	96 (4.5)	96.3 (7.7)	35.6 (20.8)
Nez Perce National Historical Park	27.4 (13.8)	60.4 (13.7)	87.8 (16.1)	26.5 (40)	87.1 (38.8)	9.1 (11.3)
Ninety Six National Historic Site	36 (18.6)	57 (62.8)	84.3 (27.2)	98.9 (0.2)	98.5 (1.4)	27 (6.2)
Niobrara National Scenic River	96.1 (11.7)	39 (38.5)	92.3 (15.7)	99.7 (1)	100 (0)	91.9 (19.4)
Noatak National Preserve	3.3 (6.7)	7.8 (3.3)	51.8 (46.1)	43.4 (9)	74 (24.3)	58.5 (61.5)
North Cascades National Park	29.5 (18.2)	48 (18.8)	91.1 (3.2)	52.5 (95.6)	54.4 (42.1)	39.1 (30.3)
Obed Wild and Scenic River	81.1 (51.5)	50.2 (48.5)	98.9 (0.2)	94.5 (6.7)	99.7 (1)	67.2 (39)
Ocmulgee National Monument	47 (60.7)	43.7 (88.1)	72.3 (33.4)	88.2 (20.7)	45 (45.6)	52.2 (24.8)
Olympic National Park	37.9 (27.5)	44 (38.6)	93.9 (10.7)	43 (1.5)	95 (8.9)	35.9 (31.2)
Oregon Caves National Monument	63.1 (90.1)	26.5 (28.7)	20.9 (54.5)	98.2 (5.4)	73.9 (1.9)	48.5 (41.9)
Organ Pipe Cactus National Monument	57.9 (57)	40.5 (50.3)	35.3 (36.2)	41.3 (62)	64.4 (55.8)	9.1 (16)
Ozark National Scenic Riverways	5.8 (2.9)	15.4 (29.9)	98.9 (2.4)	66.4 (54.9)	83 (23.6)	42.8 (25.4)
Padre Island National Seashore	72.8 (29.8)	65.8 (34.5)	23.2 (50.9)	86.9 (25.5)	98.2 (2.9)	17.3 (41.7)
Palo Alto Battlefield National Historical Park	68.8 (30.2)	61.8 (33.8)	5.5 (5.2)	85.9 (23.6)	98.9 (2.4)	17.3 (41.7)
Pea Ridge National Military Park	21.9 (21.9)	17.9 (31.6)	68.2 (53.9)	67.6 (32.7)	99.3 (1.2)	37.8 (5)
Pecos National Historical Park	23.2 (25.2)	67.6 (41)	30.8 (84.5)	77.3 (56.3)	94 (11.4)	68.5 (80.3)
Petersburg National Battlefield	46.9 (27.4)	30.8 (25.5)	94.5 (16.5)	91.5 (13.1)	54.9 (44.4)	48.8 (82.4)
Petrified Forest National Park	26.7 (25.1)	26.7 (25.8)	27.5 (57.3)	30.3 (35.3)	64.3 (32.6)	56.2 (63.3)
Petroglyph National Monument	31.1 (21.6)	52.2 (45.7)	37.5 (86.2)	69.8 (68)	89.7 (23.1)	35 (56.6)
Pictured Rocks National Lakeshore	12.2 (20)	12.2 (21.8)	78.3 (26.4)	74.7 (26)	61.3 (60.4)	83.7 (46.6)
Pinnacles National Monument	44.3 (96.6)	19 (30.8)	66.1 (84.2)	66.7 (43.2)	33 (31.4)	23.9 (37.3)
Pipe Spring National Monument	36.9 (44.6)	37.1 (41)	14.6 (16.8)	43.3 (38.1)	67.7 (49.3)	74.1 (12.9)
Pipestone National Monument	25.3 (48.7)	36.5 (82.1)	84 (25.4)	97.9 (1.8)	99.6 (1.1)	71.6 (81.6)
Piscataway Park	43.7 (52.3)	39.5 (26.4)	97.1 (8.7)	96 (5.6)	55.7 (80.7)	65.7 (33.9)
Point Reyes National Seashore	24.4 (60.5)	15.5 (32.7)	44.5 (80.2)	85.8 (28)	40.2 (17.7)	9.9 (24)
Port Chicago Naval Magazine National Memorial	46.2 (88.9)	22.8 (35.5)	59.7 (84.5)	83 (36.6)	43.4 (16.1)	33.1 (32.9)
Presidio of San Francisco	32.8 (74.7)	22 (35.3)	55.3 (84.1)	83.4 (34.4)	55.4 (24.4)	4 (8.6)
Prince William Forest Park	42.3 (49)	43.8 (30.8)	96.4 (10.7)	95.3 (6.7)	63.6 (78.8)	68.3 (54.4)
Pu'uhonua o Honaunau National Historical Park	98.1 (5.8)	98.7 (2.9)	36.3 (52.3)	11.9 (6.9)	10.1 (18)	42.4 (18.4)
Puukohola Heiau National Historic Site	98.1 (5.8)	98 (3.2)	34.8 (39.7)	10.4 (8.5)	7.3 (3.8)	42.4 (18.4)
Rainbow Bridge National Monument	10.8 (14.1)	35 (35.3)	36.4 (64.3)	33 (30.7)	78.5 (41)	62.5 (27.3)
Redwood National Park	62 (89.8)	27.9 (34.8)	15.1 (41.2)	96.8 (9.7)	67.2 (5.1)	93.3 (12.2)
Richmond National Battlefield Park	45.1 (27.2)	37.8 (22.5)	94.5 (16.5)	93.5 (13.1)	53.6 (58.3)	53.4 (64)
Rio Grande Wild and Scenic River	66.7 (37.7)	90.2 (17.7)	62 (65.6)	95.5 (7.5)	58.2 (32.6)	53 (37)
Rock Creek Park	40.9 (44.7)	39.7 (29.3)	97.1 (8.7)	95.6 (5.6)	59.7 (79.7)	65.9 (38.7)
Rocky Mountain National Park	23.1 (36.8)	43.2 (74.3)	45.4 (57.1)	84.2 (14.4)	89.9 (15.7)	33.8 (48.3)

Park	Cld1	Cld4	Vap18	Wet12	Wet18	Frs12
Roosevelt-Vanderbilt Headquarters	22.7 (41)	41.7 (84.7)	82.1 (31.3)	79.9 (10.7)	93.1 (19.4)	93.7 (15.4)
Russell Cave National Monument	65.3 (60.7)	46.8 (51.4)	98.9 (0.2)	91.1 (5.8)	95.4 (1.8)	24.5 (15.6)
Sagamore Hill National Historic Site	16 (21.2)	31.1 (84.5)	96.3 (2.9)	80.6 (22.1)	93.1 (19.4)	91.7 (2.9)
Saguaro National Park	52.5 (47.9)	43 (40.1)	51.2 (67.6)	33.8 (73.9)	83.8 (36.9)	6.4 (8.2)
Saint Croix National Scenic Riverway	11 (26.7)	4.7 (9.6)	84.8 (16.7)	94.7 (3.6)	96.8 (9.6)	73.8 (78.6)
Saint-Gaudens National Historic Site	24.2 (36.4)	52.3 (79.2)	37.7 (22.1)	72.5 (43)	91.8 (23.3)	96.9 (2.7)
Salinas Pueblo Missions National Monument	40.4 (12.3)	55.5 (30.7)	35.9 (80.7)	69 (65)	91.3 (18.2)	60 (84.7)
San Antonio Missions National Historical Park	53.4 (48.8)	60.5 (22)	88.1 (28)	95.9 (4.6)	94.9 (9.6)	8.2 (10.2)
San Juan Island National Historical Park	33.6 (6.4)	47.7 (25)	95.7 (9.7)	25.7 (28.7)	31.1 (41.6)	54.6 (44.2)
Sand Creek Massacre National Historic Site	36 (55.9)	79.6 (32.5)	53.4 (82.7)	96.4 (10.7)	97.9 (1.7)	7.5 (10.2)
Santa Monica Mountains National Recreation Area	61.2 (97.1)	3.7 (5.5)	57.3 (88.1)	21.8 (7.8)	74.1 (29.3)	19.4 (27.2)
Saratoga National Historical Park	42.2 (67.7)	62.3 (77.2)	41.2 (29.6)	55.1 (58.6)	90.8 (24.3)	72.5 (26.1)
Saugus Iron Works National Historic Site	33.8 (56)	44.3 (86.2)	73.8 (13)	64.5 (34.9)	83.3 (40.7)	86.3 (20.9)
Scotts Bluff National Monument	52.4 (39.1)	31.3 (37.2)	76.5 (36.1)	98.1 (4.8)	98.7 (3.9)	71.3 (40.8)
Sequoia and Kings Canyon National Parks	65.8 (95.1)	23.8 (6.2)	62.5 (93)	49.5 (8.9)	53.7 (27.4)	62.2 (71.7)
Shenandoah National Park	55.6 (61.8)	43.5 (31.9)	97.4 (7.8)	97.5 (1.3)	76 (50.1)	71.7 (50.8)
Shiloh National Military Park	51.6 (29.6)	19.4 (33.4)	89.8 (19.1)	88.1 (2.6)	95.7 (1.6)	22 (26.8)
Sitka National Historical Park	14.5 (10.7)	51.9 (48.9)	15.5 (19.6)	10.4 (23.4)	41.2 (29.9)	26.9 (49)
Sleeping Bear Dunes National Lakeshore	51.7 (83.4)	32.6 (82.1)	86.4 (13.3)	97.7 (4.8)	83.1 (28)	65.7 (63.8)
Stones River National Battlefield	66.2 (68)	41.1 (52.4)	95 (15.1)	92.5 (4.9)	97.2 (1.7)	57.7 (18.5)
Sunset Crater Volcano National Monument	55.8 (56.1)	43.8 (35.6)	17.2 (29.9)	26.7 (14.6)	52.5 (25.4)	76.2 (34.4)
Tallgrass Prairie National Preserve	26.4 (41.1)	36.6 (20.5)	80.7 (29.8)	84.8 (24.3)	100 (0)	62.9 (50)
Theodore Roosevelt National Park	63.4 (49.8)	52.2 (45.1)	88.2 (21.4)	54.7 (32.8)	65.7 (38.2)	50.8 (53.5)
Thomas Stone National Historic Site	41.7 (46.1)	39.1 (27.5)	97.1 (8.7)	95.7 (6.7)	54.6 (81.7)	65.6 (39.2)
Timpanogos Cave National Monument	24.1 (41.5)	6.8 (9.9)	12.3 (20.8)	88.7 (18.3)	63.3 (56.2)	45.1 (31.3)
Timucuan Ecological and Historic Preserve	13.4 (16.3)	20.6 (4.3)	61.5 (13.7)	97.8 (0.5)	83 (33.2)	84.9 (31.3)
Tonto National Monument	49.6 (14.1)	31.4 (27.3)	14.6 (8.4)	29.2 (44.5)	58.7 (30.6)	40.3 (44.3)
Tumacacori National Historical Park	42.8 (34.8)	39.2 (44)	38.4 (52.7)	37.1 (86)	84.2 (28.2)	12.2 (30.4)
Tuzigoot National Monument	73.5 (36.3)	44.6 (25.6)	9.1 (14.2)	28.9 (17.2)	52.1 (24.2)	32.2 (61.6)
Upper Delaware National Scenic and Recreational River	49.8 (76.4)	43 (93.3)	86.7 (19.7)	96.2 (7.4)	95.7 (11.7)	78.3 (19)
Valley Forge National Historical Park	30.7 (45.9)	24.3 (54.3)	94.7 (12.5)	97.1 (1.7)	88.6 (33)	65.4 (14.5)
Valor in the Pacific National Monument	86.2 (35)	68 (23.7)	34.3 (53.9)	100 (0)	86.8 (22.1)	28 (40.4)
Vicksburg National Military Park	50 (5.2)	17 (31.6)	85.1 (41.9)	88.5 (18.7)	74.5 (29.2)	21.1 (42.5)
Virgin Islands National Park	95.4 (10.6)	93.3 (13.6)	16 (41.1)	99 (1.9)	99.6 (1.2)	NA (NA)
Voyageurs National Park	3.8 (5.7)	19.8 (54.3)	69.6 (39.4)	89.8 (15.6)	71.2 (42.4)	49.4 (57.4)
Walnut Canyon National Monument	60.5 (53.5)	43.7 (34.7)	17.2 (29.9)	26 (14.6)	52.1 (24.2)	73.2 (37.3)
War In The Pacific National Historical Park	98.7 (3.9)	100 (0)	60.5 (14.4)	40.7 (24.4)	65.1 (48.8)	NA (NA)
Washita Battlefield National Historic Site	29.9 (42.3)	81.4 (43.7)	56.7 (54.4)	86.4 (21.5)	74.4 (7.3)	55.2 (49.2)
Weir Farm National Historic Site	14.2 (24.1)	34.2 (85)	93.1 (14.1)	76.1 (27.2)	89.9 (29.1)	89 (12.3)
Whiskeytown National Recreation Area	71.6 (76.5)	40.7 (42.3)	46.6 (69.1)	96.4 (10.8)	74.2 (10.8)	42.7 (24.4)

Park	Cld1	Cld4	Vap18	Wet12	Wet18	Frs12
White Sands National Monument	66.2 (41.9)	58.8 (21.3)	38.4 (93.5)	54.9 (36.2)	63.2 (37.6)	32.7 (93)
Whitman Mission National Historic Site	51.1 (35.7)	54.3 (6.4)	90.5 (12.3)	64.4 (67.8)	96.4 (10.7)	33.1 (34)
Wilson's Creek National Battlefield	6.9 (5.3)	17.2 (29.6)	71.3 (47.3)	38.5 (24.2)	99.6 (1.2)	44.1 (9.4)
Wind Cave National Park	51.6 (42.1)	23.3 (20.5)	81.1 (47)	99.4 (1.9)	63.6 (70.8)	46.9 (32.5)
Wolf Trap National Park for the Performing Arts	40 (42.3)	43.5 (27.8)	97.1 (8.7)	95.6 (5.6)	62.5 (79.7)	63.9 (51.8)
Wrangell - St Elias National Park and Preserve	7.1 (10.6)	10 (20.1)	29.8 (41.8)	56.4 (27.9)	78.2 (18.2)	24 (21.2)
Wright Brothers National Memorial	33.4 (29)	7.7 (14.1)	92.2 (23.3)	99.4 (1.9)	41 (24.2)	29.6 (65.6)
Wupatki National Monument	45.9 (58.9)	41.8 (39.4)	28.6 (58.8)	27.5 (16)	55.8 (31.4)	77.5 (37.1)
Yellowstone National Park	31.3 (72.4)	39.7 (57.9)	64.8 (70.1)	21.2 (31.7)	83.1 (49.5)	13.1 (14.4)
Yosemite National Park	67.6 (87.1)	24.3 (7.4)	62.6 (95.6)	60 (30.2)	16.7 (27.4)	53.4 (89.6)
Yucca House National Monument	13.9 (20)	13.2 (9.1)	23.6 (48.9)	34.2 (31.5)	95.4 (11.7)	45.9 (21.2)
Yukon-Charley Rivers National Preserve	12.6 (27.8)	16.9 (35.1)	23.9 (47.7)	83 (15.6)	83.1 (27.2)	44.3 (55.6)
Zion National Park	29.9 (31)	35 (36.6)	31.6 (50.8)	54 (56.6)	69.9 (49.3)	88.2 (12.8)
